# Iron-Catalyzed Coupling of Alkenes and Enones: Sakurai–Michael-type Conjugate Addition of Catalytic Allyliron Nucleophiles

**DOI:** 10.1021/acs.orglett.3c00139

**Published:** 2023-02-27

**Authors:** Sarah G. Scrivener, Yidong Wang, Yi-Ming Wang

**Affiliations:** † Department of Chemistry, University of Pittsburgh, Pittsburgh, Pennsylvania 15260, United States; ‡ School of Chemistry & Chemical Engineering, Yangzhou University, Yangzhou, Jiangsu 225002, China

## Abstract

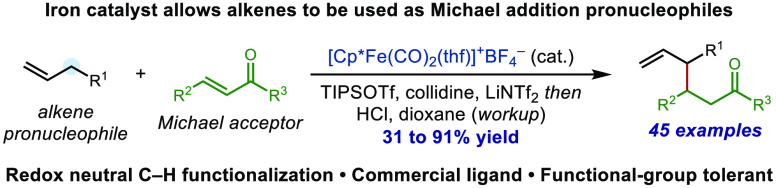

The iron-catalyzed coupling of alkenes and enones through allylic C(sp^3^)–H functionalization is reported. This redox-neutral process employs a cyclopentadienyliron(II) dicarbonyl catalyst and simple alkene substrates to generate catalytic allyliron intermediates for 1,4-addition to chalcones and other conjugated enones. The use of 2,4,6-collidine as the base and a combination of triisopropylsilyl triflate and LiNTf_2_ as Lewis acids was found to facilitate this transformation under mild, functional group-tolerant conditions. Both electronically unactivated alkenes as well as allylbenzene derivatives could be employed as pronucleophilic coupling partners, as could a range of enones bearing electronically varied substituents.

The 1,4-addition reaction to α,β-unsaturated carbonyl derivatives is a powerful synthetic tool that reliably forms C–C and C–heteroatom bonds with predictable regiochemical outcomes. Classically, C–C bond formation through Michael additions occurs via the 1,4-addition of a stabilized anionic carbon nucleophile that is generated *in situ* from an acidic pronucleophile and catalytic alkoxide base. However, without recourse to harsh bases, classical deprotonation strategies are largely limited to stabilized nucleophiles bearing moderate to strong electron-withdrawing groups (e.g., ketones and 1,3-dicarbonyl compounds).^1^ Lewis acid catalysis^2^ has been employed to expand the scope of Michael addition to less acidic pronucleophiles (e.g., simple esters and nitriles), while the use of preformed organometallic reagents^3^ and stable enol equivalents^4^ has further broadened the scope of 1,4-addition chemistry to nucleophiles that would be difficult to generate selectively by *in situ* deprotonation. However, the need for prefunctionalization steps reduces the attractiveness and practicality of protocols that call for preformed nucleophiles, particularly if the requisite organometallic reagent is complex or functional group-rich.

Recently developed catalytic strategies have allowed for the generation of carbon nucleophiles for 1,4-addition from unactivated or weakly activated C(sp^3^)–H bonds, allowing prefunctionalization to be avoided.^5^ One such strategy is catalytic metalation by transition metals enabled by the use of directing groups (Scheme 1A).^6^ This approach has allowed for the generation of alkylmetal and benzylmetal species using 2-aminopyridyl^7a^ and 8-quinolinyl^7b^ directing groups, respectively. In another approach, *N*-heterocyclic carbene (NHC) catalysis has been applied to β-C–H functionalization of carbonyl compounds (Scheme 1B).^8^ In this approach, formation of the NHC-bound intermediate promotes consecutive deprotonation of the α- and β-C(sp^3^)–H bonds to generate the nucleophilic Michael donor. Finally, nucleophilic alkyl radicals generated from C(sp^3^)–H bonds have also been employed in 1,4-addition chemistry (Scheme 1C).^9^ These protocols leverage photoredox catalysis to perform the requisite hydrogen-atom transfer (HAT) and single-electron transfer steps.

**Scheme 1:**
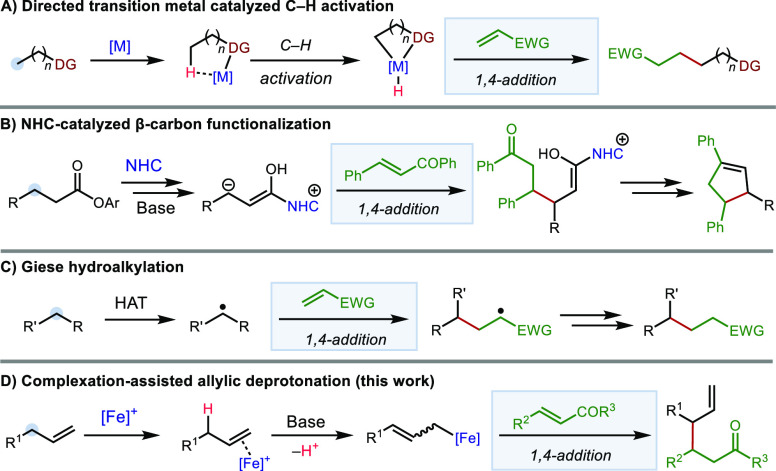
Catalytic Strategies for the Formal 1,4-Addition of C(sp^3^)–H Bonds to Electron-Deficient Alkenes

These strategies have greatly expanded the range of C(sp^3^)–H bonds that can serve as precursors to nontraditional Michael donors, but most reported processes require a directing group or the use of highly symmetrical substrates in order to avoid the formation of regioisomeric mixtures. The use of more general and synthetically versatile classes of substrates with weakly activated C(sp^3^)–H bonds as pronucleophiles for 1,4-addition remains largely undeveloped.^6^ In this context, the application of alkenes as precursors to allyl nucleophiles for 1,4-addition chemistry represents a potentially valuable yet unreported coupling strategy. Such a process would constitute a streamlined alternative to the Sakurai–Michael^10^ addition, wherein preformed allylmetal reagents (e.g., allylsilanes and -stannanes) are employed as allyl anion equivalents for 1,4-addition to α,β-unsaturated carbonyl derivatives. The Sakurai–Michael reaction has been applied as a key step in the synthesis of several biologically relevant molecules, including (−)-xialenon A,^11a^ (±)-hirsutene and (±)-capnellene,^11b^ (−)-acutumine and (−)-dechloroacutumine,^11c^*ent*-callilongisin B,^11d^ and 3-*epi*-hypatulin B.^11e^ It has also been used as a tool for the construction of useful early building blocks for natural product synthesis.^12^ In this Communication, we report the development of an iron-catalyzed coupling of alkenes and enones that realizes this novel C–C bond-forming strategy, delivering δ,ε-unsaturated ketones through the generation of catalytic allyliron intermediates that undergo subsequent 1,4-addition.

Our group has previously demonstrated that cyclopentadienyliron(II) dicarbonyl complexes are capable of coordinating to and enhancing the C–H acidity of a range of unsaturated substrates, to facilitate the removal of protons at the allylic, propargylic, or allenic positions.^13^ As a result, functional group-tolerant amine and pyridine bases could be used for abstraction of the α-proton to deliver a nucleophilic organoiron species that could undergo subsequent electrophilic functionalization with predictable S_E_2′ selectivity. We hypothesized that an allyliron intermediate arising from an alkene substrate could undergo 1,4-addition to an enone substrate activated by Lewis acid (Scheme 1D). We further hypothesized that in the presence of a silyl triflate, the resultant adduct would be trapped as a silyl enol ether (Scheme 2, **3-Si**) that could subsequently be desilylated in an acidic workup step to afford the desired ketone (**3**).

**Scheme 2:**
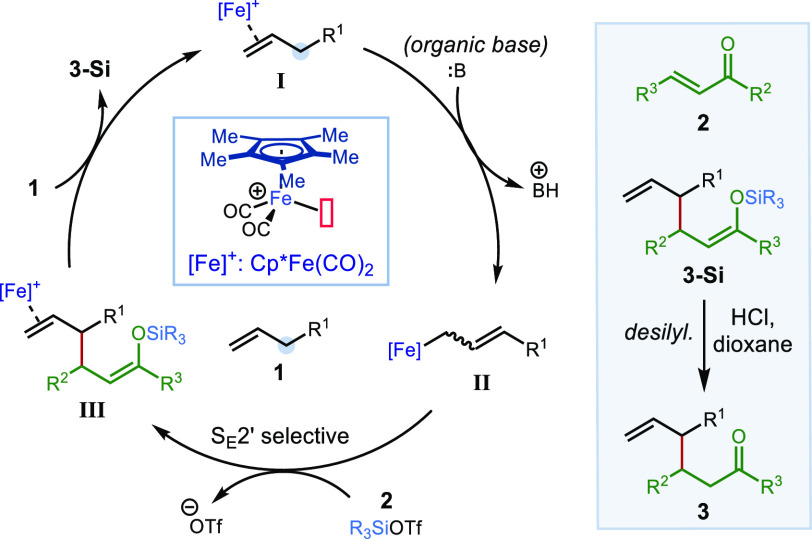
[Cp*Fe(CO)_2_]^+^ for the Catalytic Coupling of Alkenes and Enones

We commenced reaction development using our previously reported combination of base (2,2,6,6-tetramethylpiperidine, TMPH) and iron catalyst ([Cp*Fe(CO)_2_(thf)]^+^BF_4_^–^) as the starting point. We selected chalcone^14^ (**1a**) and allylbenzene (**2a**) as the Michael acceptor and pronucleophile, respectively, for the initial investigations. With TMSOTf chosen as the silylating agent, the initially formed silyl enol ether could be observed by ^1^H NMR spectroscopy of the crude material. After silyl deprotection (4 N HCl in dioxane), desired ketone **3aa** was detected in modest yield as a mixture of diastereomers (Table 1, entry 1). Switching the base to 2,4,6-collidine resulted in a substantial improvement in yield (entry 3), as did switching the solvent from chlorobenzene to toluene (entry 4). Extensive investigations into reaction stoichiometry showed that yields could be maintained when the amounts of TMSOTf and collidine were simultaneously reduced (entry 4 vs 7). With this new stoichiometry, the choice of silyl triflate was scrutinized. More hindered reagents gave significant improvements in the yield (entries 8–10). Finally, with TIPSOTf selected as the silylating reagent, the incorporation of substoichiometric LiNTf_2_ as an additive^15^ was found to further improve the reactivity, delivering **3aa** in 82% isolated yield (entry 11).

**Table 1:**
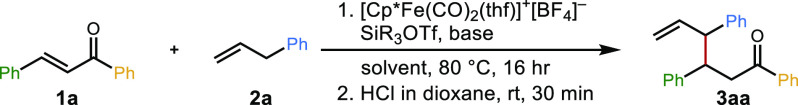
Optimization of Iron-Catalyzed Alkene–Enone Coupling

entry	base	solvent	ratio (**2a**/LA/base)^a^	Lewis acid	yield (%)^b^
1	TMPH	PhCl	3/2.5/4	TMSOTf	29
2	lutidine	PhCl	3/2.5/4	TMSOTf	20
3	collidine	PhCl	3/2.5/4	TMSOTf	46
4	collidine	PhCH_3_	3/2.5/4	TMSOTf	52
5	collidine	PhCH_3_	3/2.5/2	TMSOTf	44
6	collidine	PhCH_3_	3/1.5/4	TMSOTf	45
7	collidine	PhCH_3_	3/1.5/2	TMSOTf	51
8	collidine	PhCH_3_	3/1.5/2	TESOTf	61
9	collidine	PhCH_3_	3/1.5/2	TBDPSOTf	65
10	collidine	PhCH_3_	3/1.5/2	TIPSOTf	76
**11**^c^	**collidine**	**PhCH_3_**	**3/1.5/2**	**TIPSOTf**	**82**^d^

a Molar ratio relative to 1 equiv of **1**, using [Cp*Fe(CO)_2_(thf)]^+^[BF_4_]^−^ (20 mol %) and solvent at 1.5 M. LA = Lewis acid.

b Yields were determined by ^1^H NMR using 1,3-dinitrobenzene as the internal standard.

c LiNTf_2_ (0.35 equiv) was included as an additive.

d Isolated yield: 82% (0.3 mmol scale). DCE = 1,2-dichloroethane. TMPH = 2,2,6,6-tetramethylpiperidine. Lutidine = 2,6-dimethylpyridine. Collidine = 2,4,6-trimethylpyridine.

With these reaction conditions in hand, we sought to understand the scope of the Michael acceptor. With allylbenzene as the alkene coupling partner, a variety of substituted chalcone substrates were found to give the desired coupling products in moderate to excellent yields. Chalcones bearing aryl substituents that range from moderately electron-rich (e.g., **1b**, **1k**, **1l**, **1r**) to highly electron-deficient (e.g., **1f, 1m**, **1p**) on either aryl ring were found to be competent substrates. Moreover, products containing a variety of functional groups and heterocycles, including a nitro group (**3fa**), a boronic acid pinacol ester (**3ha**), a methylenedioxy group (**3ia**), a thiophene (**3ka**), a furan (**3la**), a trifluoromethylated pyridine (**3ma**), and a tosyl-protected amine (**3ra**), could all be prepared using the current protocol.

We then investigated the scope of the alkene pronucleophile. Allylbenzene derivatives spanning a range of electronic properties afforded the coupling products in good to excellent yields (**3ab**–**3ae**) under the optimized conditions. Under slightly modified conditions, phthalimide-protected allylic and homoallylic amines (**2j** and **2k**) were also found to be competent alkene coupling partners, as was 1,2-dihydronaphthalene (**2l**). In addition, a variety of electronically unactivated alkenes^16^ gave good yields, including functionalized derivatives containing an alkyl *p*-toluenesulfonate ester (**2n**) and an internal alkyne (**2r**). In the case of the latter, it is noteworthy that functionalization occurred exclusively at the allylic (and not the propargylic) position. Finally, products incorporating a pharmaceutical fragment (**3aq**) or five-membered heterocycles (**3ao** and **3ap**) could also be obtained.

**Table 2:**
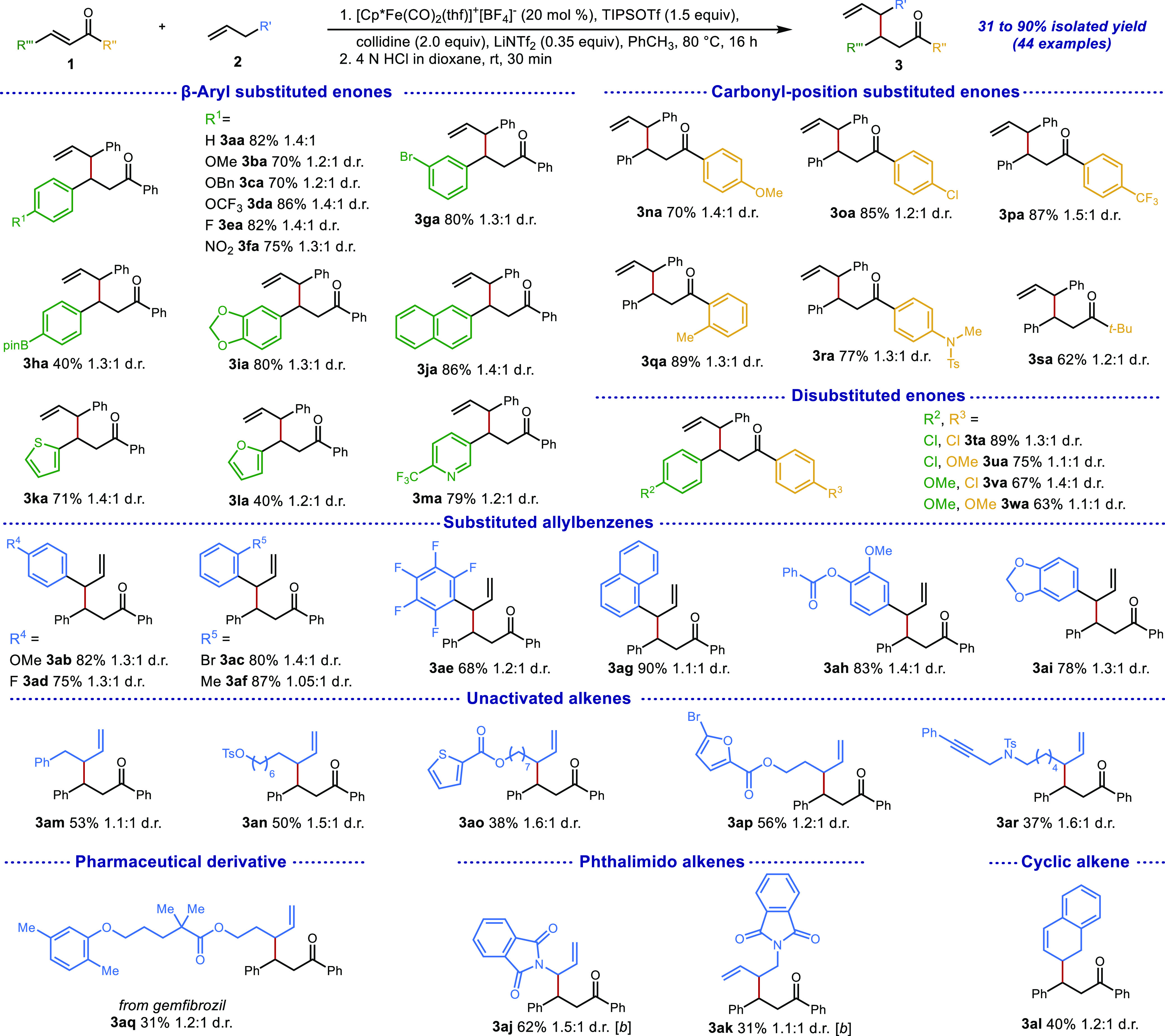
Enone and Alkene Scope^a^

a Conditions: 1. **1** (0.3 mmol), allylbenzene (3.0 equiv), [Cp*Fe(CO)_2_(thf)]^+^[BF_4_]^−^ (20 mol %), TIPSOTf (1.5 equiv), collidine (2.0 equiv), LiNTf_2_ (0.35 equiv), toluene [1.5 M], 80 **°**C, 16 h. 2. 4 N HCl in dioxane.

b Conditions: 1. **1** (0.3 mmol), allylbenzene (3.0 equiv), [Cp*Fe(CO)_2_(thf)]^+^[BF_4_]^−^ (15 mol %), TIPSOTf (1.5 equiv), collidine (2.0 equiv), LiNTf_2_ (0.8 equiv), toluene [1.5 M], 80 **°**C, 16 h. 2. 4 N HCl in dioxane. Isolated yields.

To further demonstrate the utility of this protocol, the synthesis of **3ob** was carried out on a 5.0 mmol scale to deliver 1.78 g of the product (91% isolated yield) using 15 mol % loading of the iron catalyst (Scheme 3). In addition, the products could be further elaborated using methods reported in the literature. In particular, a cyclopentene (**4**) could be prepared in 77% overall yield from **3ob** through a Wittig/ring-closing metathesis sequence, while Buchwald–Hartwig cross-coupling of **3ob** with piperidine afforded **5** in 80% yield. Finally, the ozonolysis of **3aa** followed by acid-catalyzed cyclization gave 4*H*-pyran **6** in 45% yield over two steps.

**Scheme 3:**
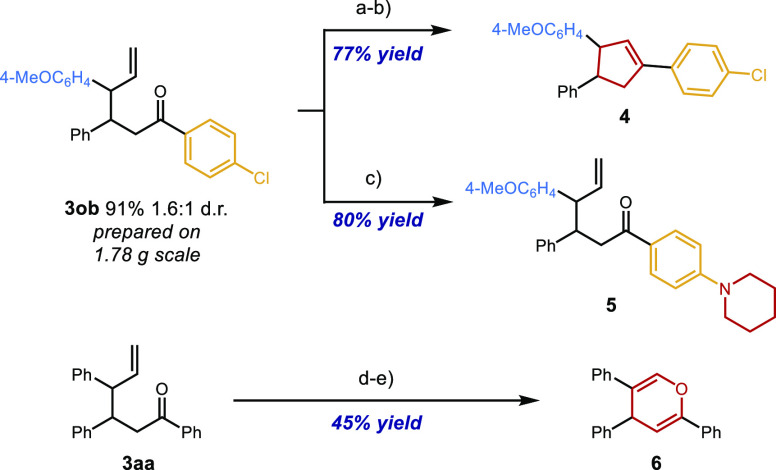
Divergent Transformations of Products **3** Conditions: (a) Ph_3_PMeBr (1.2 equiv), *t*-BuOK (1.2 equiv), THF, rt. (b) Grubbs II (10 mol %), DCE, 80 °C. (c) Pd(OAc)_2_ (4 mol %), XPhos (10 mol %), NaO^*t*^Bu (1.4 equiv), piperidine (1.2 equiv), THF, 100 °C. (d) O_3_, CH_2_Cl_2_, −78 °C. (e) H_3_PO_4_ (aq., cat.), MgSO_4_, toluene, 110 °C.

In summary, we have developed an iron-catalyzed coupling of alkenes and enones to give a variety of δ,ε-unsaturated ketones through a Michael addition facilitated by the deprotonative generation of catalytic allyliron intermediates. Further explorations of this strategy toward the use of a broader range of Michael acceptors and improved stereocontrol are ongoing in our laboratory and will be reported in due course.

## Data Availability

The data underlying this study are available in the published article and its Supporting Information.

## Supporting Information Available

Experimental procedures and spectroscopic data for the substrates and products (PDF)

## References

[ref1] aBako P.; Nemcsok T.; Rapi Z.; Keglevich G. Enantioselective Michael Addition of Malonates to Enones. Curr. Org. Chem. 2020, 24, 746–773. 10.2174/1385272824666200316122221.

[ref2] aKanemasa S.; Hasegawa M.; Ono F. Catalytic activation through metal enolization of nucleophile precursors and synthetic applications to enantioselective Michael additions. Chem. Rec. 2007, 7, 137–149. 10.1002/tcr.20105. 17549687

[ref3] aBurns A. R.; Lam H. W.; Roy I. D. Enantioselective, Rhodium-Catalyzed 1,4-Addition of Organoboron Reagents to Electron-Deficient Alkenes. In Organic Reactions; Denmark S. E., Ed.; Wiley, 2017; Vol. 93, pp 1–415.

[ref4] Mukaiyama T.; Banno K.; Narasaka K. New cross-aldol reactions. Reactions of silyl enol ethers with carbonyl compounds activated by titanium tetrachloride. J. Am. Chem. Soc. 1974, 96, 7503–7509. 10.1021/ja00831a019.

[ref5] aMandal D.; Roychowdhury S.; Biswas J. P.; Maiti S.; Maiti D. Transition-metal-catalyzed C–H bond alkylation using olefins: recent advances and mechanistic aspects. Chem. Soc. Rev. 2022, 51, 7358–7426. 10.1039/D1CS00923K. 35912472

[ref6] Yang L.; Huang H. Transition-Metal-Catalyzed Direct Addition of Unactivated C–H Bonds to Polar Unsaturated Bonds. Chem. Rev. 2015, 115, 3468–3517. 10.1021/cr500610p. 25749375

[ref7] aPan S.; Matsuo Y.; Endo K.; Shibata T. Cationic iridium-catalyzed enantioselective activation of secondary sp^3^ C–H bond adjacent to nitrogen atom. Tetrahedron 2012, 68, 9009–9015. 10.1016/j.tet.2012.08.071.

[ref8] aFu Z.; Jiang K.; Zhu T.; Torres J.; Chi Y. R. Access to Oxoquinoline Heterocycles by N-Heterocyclic Carbene Catalyzed Ester Activation for Selective Reaction with an Enone. Angew. Chem., Int. Ed. 2014, 53, 6506–6510. 10.1002/anie.201402620. 24839111

[ref9] aGant Kanegusuku A. L.; Roizen J. L. Recent Advances in Photoredox-Mediated Radical Conjugate Addition Reactions: An Expanding Toolkit for the Giese Reaction. Angew. Chem., Int. Ed. 2021, 60, 21116–21149. 10.1002/anie.202016666. PMC8382814 33629454

[ref10] Hosomi A.; Sakurai H. Conjugate Addition of Allylsilanes to α,β-Enones. A New Method of Stereoselective Introduction of the Angular Allyl Group in Fused Cyclic α,β-Enones. J. Am. Chem. Soc. 1977, 99, 1673–1675. 10.1021/ja00447a080.

[ref11] aHodgson D. M.; Galano J.-M.; Christlieb M. Synthesis of (−)-xialenon A by enantioselective α-deprotonation-rearrangement of a *meso*-epoxide. Tetrahedron 2003, 59, 9719–9728. 10.1016/j.tet.2003.09.023.

[ref12] aXu K.; Cheng B.; Li Y.; Xu T.; Yu C.; Zhang J.; Ma Z.; Zhai H. Stereocontrolled Total Syntheses of (±)-Fawcettimine, (±)-Lycoflexine, and (±)-Lycoflexine *N*-Oxide. Org. Lett. 2014, 16, 196–199. 10.1021/ol403185g. 24295384

[ref13] aWang Y.; Scrivener S. G.; Zuo X.-D.; Wang R.; Palermo P. N.; Murphy E.; Durham A. C.; Wang Y.-M. Iron-Catalyzed Contrasteric Functionalization of Allenic C(sp^2^)–H Bonds: Synthesis of α-Aminoalkyl 1,1-Disubstituted Allenes. J. Am. Chem. Soc. 2021, 143, 14998–15004. 10.1021/jacs.1c07512. 34491051 PMC8458257

[ref14] Zhuang C.; Zhang W.; Sheng C.; Zhang W.; Xing C.; Miao Z. Chalcone: A Privileged Structure in Medicinal Chemistry. Chem. Rev. 2017, 117, 7762–7810. 10.1021/acs.chemrev.7b00020. 28488435 PMC6131713

[ref15] Ding R.; Wang Y.; Wang Y.-M. Synthesis of 1,1-Disubstituted Allenylic Silyl Ethers Through Iron-Catalyzed Regioselective C(sp^2^)–H Functionalization of Allenes. Synthesis 2023, 55, 733–743. 10.1055/a-2004-0951. PMC10237284 37274078

[ref16] Preliminary experiments showed that propene is also an acceptable alkene coupling partner, affording allylation products in modest yields. See the Supporting Information (S22 and S23) for results.

