# Supplementary material for: Iron-Catalyzed Coupling of Alkenes and Enones: Sakurai–Michael-type Conjugate Addition of Catalytic Allyliron Nucleophiles
Source: Org Lett. 2023 Feb 27;25(9):1420–4. doi: 10.1021/acs.orglett.3c00139 (PMC10006348; doi:10.1021/acs.orglett.3c00139)
Supplement: Supplementary file 1 — ol3c00139_si_001.pdf [file ol3c00139_si_001.pdf]

SUPPORTING INFORMATION *for*

Iron-Catalyzed Coupling of Alkenes and Enones: Sakurai–Michael-type Conjugate Addition of Catalytic Allyliron Nucleophiles

Sarah G. Scrivener,[a] Yidong Wang,[a,b] and Yi-Ming Wang[a]*

[a]*Department of Chemistry, University of Pittsburgh, Pittsburgh, PA, 15260*
[b]*School of Chemistry & Chemical Engineering, Yangzhou University, Yangzhou, Jiangsu 225002, China*

*ym.wang@pitt.edu (*Corresponding Author*)

Contents

General Information 2

1. General procedures 3

2. Characterization data 4

   2.1 Synthesis of Michael adducts from Fe-catalyzed Michael addition – Michael Acceptor Scope 4

   2.2 Synthesis of Michael adducts from Fe-catalyzed Michael addition – Alkene Scope 14

   2.3 Synthesis of Michael acceptor substrates 23

   2.4 Synthesis of alkene substrates 24

3. Large scale synthesis and synthetic applications of products 24

4. X-Ray determination of diastereomer assignments 28

5. Reaction optimization 30

6. Unsuccessful substrates 32

7. Copies of NMR spectra of products and substrates 33

   Copies of NMR spectra of products: Michael acceptor scope 33

   Copies of NMR spectra of products: Nucleophile scope 59

   Copies of NMR spectra of products: Large scale reaction and synthetic applications of products 81

   Copies of NMR spectra of starting materials 87

8. References 91

General Information

General Reagent Information: Anhydrous tetrahydrofuran (THF), 1,2-dichloroethane (DCE), toluene and trifluorotoluene were purchased from Acros (AcroSeal packaging), Sigma Aldrich (Sure/Seal packaging), and Frontier Scientific (J&KSeal packaging), respectively, and were sparged with nitrogen before transferring into an argon-filled glovebox and used without further purification. Other dry solvents were obtained by distillation and storage over 3Å or 4Å molecular sieves. All other reagents were purchased from Oakwood, Acros, TCI, Strem, Alfa Aesar, or Sigma Aldrich and used as received. Compounds were purified by flash column chromatography using SiliCycle SiliaFlash® F60 silica gel, unless otherwise indicated.

General Analytical Information: New compounds were characterized by ^{1}H NMR, ^{13}C NMR and HRMS. Structural assignments were made with additional information from gCOSY experiments. Copies of the ^{1}H NMR and ^{13}C NMR spectra can be found at the end of the Supporting Information. ^{1}H and ^{13}C NMR spectra were recorded on Bruker 400 MHz, or 500 MHz instruments. All ^{1}H NMR data are reported in δ units, parts per million (ppm), and were measured relative to the residual proton signal in the deuterated solvent at 7.26 ppm ($CDCl_3$). All ^{13}C NMR spectra are ^{1}H decoupled and reported in ppm relative to the solvent signal at 77.16 ppm ($CDCl_3$). Thin-layer chromatography (TLC) was performed on Silicycle 250 μm (analytical) or 1000 μm (preparative) silica gel plates. Compounds were visualized by irradiation with UV light, or by staining with potassium permanganate. Yields refer to isolated compounds, unless otherwise indicated. High resolution mass spectra were recorded on a Thermo Scientific Q-Exactive mass spectrometer. NMR yields were determined by using 2,4-dinitrotoluene as the internal standard for ^{1}H NMR spectroscopy. High resolution mass spectra were obtained by quadrupole mass analyzer on a Bruker Daltonics, Inc. APEXIII 7.0 TESLA FTMS instrument (ESI). X-ray data were collected on a Bruker X8 Prospector Ultra single crystal diffractometer with Cu Kα radiation and an Apex II CCD detector.

1. General procedures

General procedure A for the synthesis of ketones **3** through the coupling of alkenes and enones

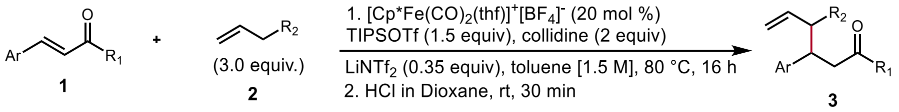

A reaction tube (13 mm × 100 mm, Fisherbrand, part # 14-959-35C) equipped with an olive shaped magnetic stir bar (10 mm) was capped with a Teflon/silicone septum (Thermo/National part # C4015-66A) screw cap, flame dried under vacuum, and transferred into an argon-filled glovebox. In the glovebox were added Michael acceptor (**1**, 0.3 mmol, 1.0 equiv), dry toluene (0.2 mL, 1.5 M), and alkene (**2**, 0.9 mmol, 3.0 equiv). The solution was briefly stirred. Then collidine (0.6 mmol, 80 µL, 2.0 equiv) and TIPSOTf (0.45 mmol, 139 µL, 1.5 equiv) were sequentially added with brief stirring after each addition. $LiNTf_2$ (0.11 mmol, 30.1 mg, 0.35 equiv) was added and the reaction vial was capped and shaken to ensure complete mixing. Finally, $[Cp^*Fe(CO)_2(thf)]^+[BF_4]^-$ (20 mol %, 24.6 mg) was added and the reaction tube was again capped and shaken. The reaction tube was then removed from the glovebox and placed in an oil bath at 80 °C for 16 h at 400 rpm. After completion of the reaction, the reaction mixture was cooled to room temperature and filtered through a short plug of silica gel using ethyl acetate. A 4 N solution of HCl in dioxane (0.6 mmol, 150 µL, 2.0 equiv) was added to the crude solution at room temperature and the mixture was stirred for 30 min at room temperature. The crude mixture was concentrated *in vacuo*. The diastereomeric ratio (dr) was then determined by ^1H NMR analysis of a portion of the crude material. After concentration *in vacuo*, the crude mixture was purified by flash column chromatography to provide the desired product.

General procedure B for the synthesis of enones **1**[1]

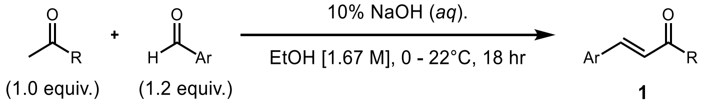

To a round bottom flask containing the ketone (2 mmol, 1.0 equiv) in ethanol (1.67 M) was added a 10% aqueous solution of NaOH (2 mL, 1.3 equiv). The corresponding aldehyde (2.4 mmol, 1.2 equiv) was added dropwise at 0 °C with stirring. The reaction mixture was allowed to warm to room temperature overnight. Water (4 mL) was added and the crude solid product was collected via vacuum filtration and recrystallized from hexanes and ether to afford the title compounds **1**.

2. Characterization data

2.1 Synthesis of Michael adducts from Fe-catalyzed Michael addition – Michael Acceptor Scope

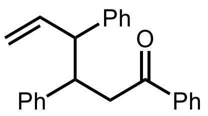

3aa

1,3,4-Triphenyl-5-henxen-1-one[2] (**3aa**): (SGS-3-185-8) Prepared following **General Procedure A** using chalcone (**1a**, 62.4 mg, 0.3 mmol, 1.0 equiv) and allylbenzene (**2a**, 119 µL, 0.9 mmol, 3.0 equiv). The reaction mixture was heated at 80 °C for 16 h. The crude reaction mixture was purified by flash column chromatography (hexanes/ethyl acetate = 100:1) to afford the title compound as a white solid (81.2 mg, 82%). The diastereomeric ratio (dr 1.4:1) was determined by NMR analysis of the crude product. The relative configuration was assigned by diastereomeric enrichment through crystallization followed by X-ray crystallography.

Throughout, the major diastereomer is indicated by an H* designation, and the minor diastereomer is indicated by an H' designation. See page S27 for relative configuration assignment of the major diastereomer.

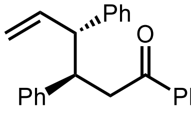

3aa (major)

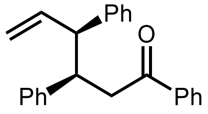

3aa (minor)

^{1}H NMR (500 MHz, $CDCl_3$) δ 7.86 (d, *J* = 7.5 Hz, 2H'), 7.72 (d, *J* = 7.5 Hz, 2H*), 7.52 (t, *J* = 7.3 Hz, 1H, Minor), 7.47 (t, *J* = 7.4 Hz, 1H*), 7.43 – 7.31 (m, 3H* + 3H'), 7.25 – 6.96 (m, 9H*+ 9H'), 6.10 (dt, *J* = 17.0, 9.8 Hz, 1H'), 5.91 – 5.78 (m, 1H*), 5.13 (dd, *J* = 60.2, 13.5 Hz, 2H'), 4.84 (dd, *J* = 48.1, 13.6 Hz, 2H*), 3.84 (td, *J* = 9.1, 5.1 Hz, 1H'), 3.78 (td, *J* = 9.4, 4.4 Hz, 1H*), 3.63 (t, *J* = 9.0 Hz, 1H*), 3.59 – 3.48 (m, 2H'), 3.39 – 3.27 (m, 1H' + 1H*), 3.10 (dd, *J* = 16.8, 4.3 Hz, 1H*).

^{13}C NMR (126 MHz, $CDCl_3$) δ 199.0, 198.9, 142.8, 142.7, 142.5, 142.4, 140.8, 139.7, 137.5, 137.4, 132.9, 132.9, 128.8, 128.6, 128.5, 128.5, 128.4, 128.27, 128.25, 128.2, 128.1, 128.03, 128.00, 126.8, 126.6, 126.2, 116.3, 57.5, 55.8, 46.6, 45.9, 43.9, 43.5 (three carbons missing due to overlap).

HRMS (ESI) calcd for $C_{24}H_{23}O$ $[M+H]^+$: 327.1743, found: 327.1741.

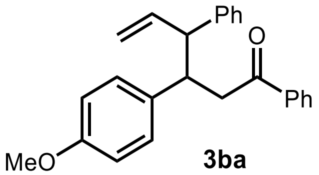

3ba

3-(4-Methoxyphenyl)-1,4-diphenylhex-5-en-1-one (**3ba**): (SGS-4-2) Prepared following **General Procedure A** using 1-(4-methoxyphenyl)-3-phenylprop-2-en-1-one (**1b**, 71.5 mg, 0.3 mmol, 1.0 equiv) and allylbenzene (**2a**, 119 µL, 0.9 mmol, 3.0 equiv). The reaction mixture was heated at 80 °C for 16 h. The crude reaction mixture was purified by flash column chromatography (hexanes/ethyl acetate = 100:1) to afford the title compound as a white solid (74.5 mg, 70%). The diastereomeric ratio (dr 1.2:1) was determined by NMR analysis of the crude product.

^{1}H NMR (500 MHz, $CDCl_3$) δ 7.86 (d, *J* = 7.5 Hz, 2H'), 7.73 (d, *J* = 7.5 Hz, 2H*), 7.52 (t, *J* = 7.4 Hz, 1H'), 7.48 (t, *J* = 7.4 Hz, 1H*), 7.41 (t, *J* = 7.7 Hz, 2H'), 7.39 – 7.29 (m, 4H*), 7.25-7.21 (m, 2H*+3H'), 7.15 (t, *J* = 7.5 Hz, 2H'), 7.11 – 7.00 (m, 3H*+2H'), 6.95 (d, *J* = 8.5 Hz, 2H'), 6.79 (d, *J* = 8.5 Hz, 2H*), 6.64 (d, *J* = 8.5 Hz, 2H'), 6.15 – 6.03 (m, 1H'), 5.93 – 5.78 (m, 1H*), 5.12 (dd, *J* = 57.5, 13.5 Hz, 2H'), 4.85 (dd, *J* = 50.8, 13.6 Hz, 2H*), 3.84 – 3.69 (m, 4H*+1H'), 3.68 (s, 3H'), 3.60 (t, *J* = 8.8 Hz, 1H*), 3.56 – 3.46 (m, 2H'), 3.39 – 3.20 (m, 1H*+1H'), 3.08 (dd, *J* = 16.6, 4.4 Hz, 1H*).

^{13}C NMR (126 MHz, CDCl$_3$) δ 199.3, 199.2, 158.2, 157.9, 143.0, 142.6, 140.9, 139.8, 137.6, 137.4, 134.7, 134.4, 132.93, 132.87, 129.7, 129.4, 128.8, 128.6, 128.5, 128.4, 128.30, 128.26, 128.10, 128.05, 126.8, 126.2, 116.3, 116.2, 113.7, 113.5, 57.5, 55.9, 55.3, 55.2, 45.9, 45.1, 43.74, 43.70.

HRMS (ESI) calcd for $C_{25}H_{25}O_2$ [M+H]$^+$: 357.1849, found: 357.1857.

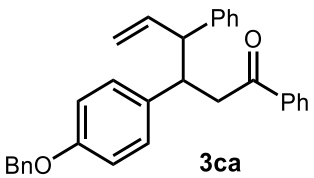

3ca

3-(4-(Benzyloxy)phenyl)-1,4-diphenylhex-5-en-1-one (**3ca**): (SGS-4-32) Prepared following **General Procedure A** using 1-(4-(benzyloxy)phenyl)-3-phenylprop-2-en-1-one (**1c**, 94.3 mg, 0.3 mmol, 1.0 equiv) and allylbenzene (**2a**, 119 μL, 0.9 mmol, 3.0 equiv). The reaction mixture was heated at 80 °C for 16 h. The crude reaction mixture was purified by flash column chromatography (hexanes/ethyl acetate = 100:1) to afford the title compound as a white solid (87.9 mg, 70%). The diastereomeric ratio (dr 1.2:1) was determined by NMR analysis of the crude product.

^{1}H NMR (500 MHz, CDCl$_3$) δ 7.84 (d, *J* = 8.8 Hz, 2H'), 7.71 (d, *J* = 8.8 Hz, 2H*), 7.44 – 6.97 (m, 15H), 6.95 (d, *J* = 8.8 Hz, 2H'), 6.89 (d, *J* = 8.8 Hz, 2H*), 6.12 – 6.05 (m, 1H'), 5.89 – 5.77 (m, 1H*), 5.22 – 5.02 (m, 2H* + 4H'), 4.82 (dd, *J* = 47.5, 13.6 Hz, 2H*), 3.82 (td, *J* = 9.1, 5.0 Hz, 1H), 3.76 (td, *J* = 9.3, 4.4 Hz, 1H'), 3.61 (t, *J* = 8.9 Hz, 1H*), 3.54 (t, *J* = 9.7 Hz, 1H'), 3.45 (dd, *J* = 16.9, 4.9 Hz, 1H'), 3.33 – 3.20 (m, 1H* + 1H'), 3.03 (dd, *J* = 16.6, 4.4 Hz, 1H*).

^{13}C NMR (126 MHz, CDCl$_3$) δ 197.5, 197.4, 162.6, 162.5, 142.9, 142.8, 142.6, 142.5, 140.9, 139.7, 136.4, 136.3, 130.8, 130.7, 130.4, 130.3, 128.83, 128.81, 128.78, 128.5, 128.4, 128.4, 128.4, 128.3, 128.2, 128.0, 127.6, 127.6, 126.8, 126.6, 126.2, 126.2, 116.3, 114.6, 114.5, 70.3, 70.2, 57.5, 55.8, 46.6, 45.9, 43.1, 43.1 (three carbons missing due to overlap).

HRMS (ESI) calcd for $C_{31}H_{29}O_2$ [M+H]$^+$: 433.2162, found: 433.2150.

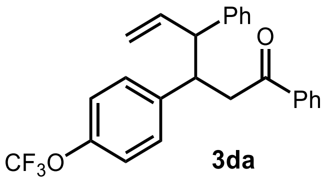

3da

1,4-Diphenyl-3-(4-(trifluoromethoxy)phenyl)hex-5-en-1-one (**3da**): (SGS-4-26) Prepared following **General Procedure A** using 3-phenyl-1-(4-(trifluoromethoxy)phenyl)prop-2-en-1-one (**1d**, 87.7 mg, 0.3 mmol, 1.0 equiv) and allylbenzene (**2a**, 119 μL, 0.9 mmol, 3.0 equiv). The reaction mixture was heated at 80 °C for 16 h. The crude reaction mixture was purified by flash column chromatography (hexanes/ethyl ether = 100:1) to afford the title compound as a white solid (106.0 mg, 86%). The diastereomeric ratio (dr 1.4:1) was determined by NMR analysis of the crude product.

^{1}H NMR (500 MHz, CDCl$_3$) δ 7.84 (d, *J* = 7.4 Hz, 1H), 7.70 (d, *J* = 7.4 Hz, 1H), 7.50 (t, *J* = 7.4 Hz, 1H), 7.45 (t, *J* = 7.4 Hz, 1H), 7.42 – 7.01 (m, 12H), 6.98 (d, *J* = 7.2 Hz, 1H), 6.91 (d, *J* = 8.2 Hz, 1H), 6.13 – 6.02 (m, 1H), 5.80 (ddd, *J* = 17.2, 10.0, 8.6 Hz, 1H), 5.13 (dd, *J* = 53.2, 13.5 Hz, 1H), 4.83 (dd, *J* = 50.4, 13.6 Hz, 1H), 3.88 – 3.74 (m, 1H), 3.66 – 3.46 (m, 2H), 3.38 – 3.20 (m, 1H), 3.10 (dd, *J* = 17.0, 4.3 Hz, 1H).

^{13}C NMR (126 MHz, CDCl$_3$) δ 198.7, 198.6, 147.86, 147.85, 147.5, 142.4, 142.1, 141.5, 141.2, 140.3, 139.2, 137.3, 137.2, 133.13, 133.07, 130.0, 129.7, 128.9, 128.7, 128.6, 128.4, 128.3, 128.1, 128.04, 127.98, 127.0, 126.5, 120.7, 120.6 (q, *J* = 256.8 Hz), 120.5 (q, *J* = 256.7 Hz), 120.5, 116.7, 57.4, 55.7, 45.9, 45.3, 43.4, 43.3.

^{19}F NMR (471 MHz, CDCl$_3$) δ -57.78, -57.86.

HRMS (ESI) calcd for $C_{25}H_{22}O_2F_3$ [M+H]$^+$: 411.1566, found: 411.1566.

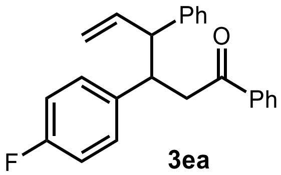

3ea

3-(4-Fluorophenyl)-1,4-diphenylhex-5-en-1-one (**3ea**): (SGS-4-1) Prepared following **General Procedure A** using 1-(4-fluorophenyl)-3-phenylprop-2-en-1-one (**1e**, 67.9 mg, 0.3 mmol, 1.0 equiv) and allylbenzene (**2a**, 119 μL, 0.9 mmol, 3.0 equiv). The reaction mixture was heated at 80 °C for 16 h. The crude reaction mixture was purified by flash column chromatography (hexanes/ethyl ether = 100:1) to afford the title compound as a white solid (84.6 mg, 82%). The diastereomeric ratio (dr 1.4:1) was determined by NMR analysis of the crude product.

^{1}H NMR (500 MHz, $CDCl_3$) δ 7.86 (d, *J* = 7.4 Hz, 2H'), 7.73 (d, *J* = 7.4 Hz, 2H*), 7.53 (t, *J* = 7.4 Hz, 1H'), 7.49 (t, *J* = 7.4 Hz, 1H*), 7.43 (t, *J* = 7.7 Hz, 2H'), 7.37 (t, *J* = 7.7 Hz, 2H*), 7.35 – 7.29 (m, 1H*), 7.25-7.22 (m, 4H*), 7.17-7.11 (m, 2H*+2H'), 7.07 (t, *J* = 7.3 Hz, 1H'), 7.04 – 6.97 (m, 4H'), 6.93 (t, *J* = 8.7 Hz, 2H*), 6.78 (t, *J* = 8.7 Hz, 2H'), 6.09 (ddd, *J* = 16.99, 9.8, 9.8, Hz, 1H'), 5.83 (ddd, *J* = 17.2, 10.1, 8.6 Hz, 1H*), 5.15 (dd, *J* = 53.5, 13.4 Hz, 2H'), 4.86 (dd, *J* = 50.8, 13.6 Hz, 2H*), 3.85 – 3.79 (m, 1H'), 3.79 – 3.73 (m, 1H*), 3.59 (t, *J* = 9.0 Hz, 1H*), 3.56 – 3.48 (m, 2H'), 3.34 (dd, *J* = 17.1, 8.8 Hz, 1H'), 3.26 (dd, *J* = 16.8, 9.4 Hz, 1H*), 3.10 (dd, *J* = 16.8, 4.3 Hz, 1H*).

^{13}C NMR (126 MHz, $CDCl_3$) δ 198.9, 198.8, 162.4 (d, *J* = 38.9 Hz), 160.5 (d, *J* = 38.6 Hz), 142.6, 142.3, 140.6, 139.4, 138.4 (d, *J* = 3.2 Hz), 138.1 (d, *J* = 3.2 Hz), 137.4, 137.3, 133.1, 133.0, 130.1 (d, *J* = 7.8 Hz), 129.9 (d, *J* = 7.9 Hz), 128.9, 128.64, 128.55, 128.38, 128.30, 128.1, 128.04, 127.98, 126.9, 126.4, 116.5, 115.1 (d, *J* = 21.1 Hz), 114.8 (d, *J* = 21.1 Hz), 57.5, 55.9, 45.9, 45.2, 43.6, 43.5 (one carbon missing due to overlap).

^{19}F NMR (471 MHz, $CDCl_3$) δ -116.55, -116.92.

HRMS (ESI) calcd for $C_{24}H_{22}OF$ $[M+H]^+$: 345.1649, found: 345.1651.

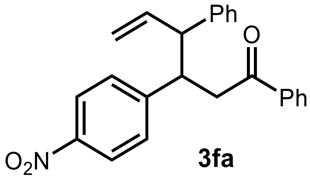

3fa

3-(4-Nitrophenyl)-1,4-diphenylhex-5-en-1-one (**3fa**): (SGS-4-3) Prepared following **General Procedure A** using 1-(4-nitrophenyl)-3-phenylprop-2-en-1-one (**1f**, 75.98 mg, 0.3 mmol, 1.0 equiv) and allylbenzene (**2a**, 119 μL, 0.9 mmol, 3.0 equiv). The reaction mixture was heated at 80 °C for 16 h. The crude reaction mixture was purified by flash column chromatography (hexanes/ethyl acetate = 100:1 to 50:1) to afford the title compound as a white solid (84.1 mg, 75%). The diastereomeric ratio (dr 1.3:1) was determined by NMR analysis of the crude product.

^{1}H NMR (500 MHz, $CDCl_3$) δ 8.12 (d, *J* = 8.7 Hz, 2H*), 7.95 (d, *J* = 8.7 Hz, 2H'), 7.87 (d, *J* = 7.5 Hz, 2H'), 7.73 (d, *J* = 7.4 Hz, 2H*), 7.55 (t, *J* = 7.4 Hz, 1H'), 7.50 (t, *J* = 7.4 Hz, 1H*), 7.45 – 7.01 (m, 13H* +13H'), 6.12 – 6.05 (m, 1H'), 5.80 (ddd, *J* = 17.1, 10.0, 9.0 Hz, 1H*), 5.20 (dd, *J* = 48.4, 13.5 Hz, 2H'), 4.86 (dd, *J* = 41.8, 13.6 Hz, 2H*), 3.99 – 3.87 (m, 1H*+1H'), 3.68 – 3.59 (m, 1H*+1H'), 3.57 – 3.52 (m, 1H'), 3.44 – 3.31 (m, 1H*+1H'), 3.16 (dd, *J* = 17.3, 4.0 Hz, 1H*).

^{13}C NMR (126 MHz, $CDCl_3$) δ 198.1, 198.0, 151.0, 150.7, 146.8, 146.4, 141.9, 141.6, 139.9, 138.8, 137.0, 136.9, 133.4, 133.4, 129.6, 129.4, 129.1, 128.8, 128.70, 128.67, 128.2, 128.04, 127.98, 127.9, 127.3, 126.8, 123.6, 123.4, 117.2, 117.1, 57.1, 55.7, 46.4, 45.8, 43.2, 43.0.

HRMS (ESI) calcd for $C_{24}H_{22}O_3N$ $[M+H]^+$: 372.1594, found: 372.1593.

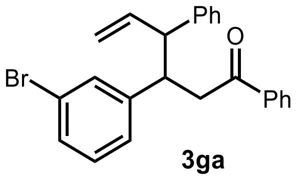

3ga

3-(3-Bromophenyl)-1,4-diphenylhex-5-en-1-one (**3ga**): (SGS-4-20) Prepared following **General Procedure A** using 1-(3-bromophenyl)-3-phenylprop-2-en-1-one (**1g**, 86.1 mg, 0.3 mmol, 1.0 equiv) and allylbenzene (**2a**, 119 μL, 0.9 mmol, 3.0 equiv). The reaction mixture was heated at 80 °C for 16 h. The crude reaction mixture was purified by flash column chromatography (hexanes/ethyl ether = 100:1) to afford the title compound as a white solid (97.2 mg, 80%). The diastereomeric ratio (dr 1.3:1) was determined by NMR analysis of the crude product.

^{1}H NMR (500 MHz, $CDCl_3$) δ 7.90 – 7.85 (m, 2H'), 7.75 – 7.70 (m, 2H*), 7.54 (t, J = 7.4 Hz, 1H'), 7.49 (t, J = 7.4 Hz, 1H*), 7.46 – 6.91 (m, 11H* + 11H'), 6.12 – 6.01 (m, 1H'), 5.82 (ddd, J = 17.1, 10.1, 8.5 Hz, 1H*), 5.14 (dd, J = 55.0, 13.5 Hz, 2H'), 4.86 (dd, J = 52.7, 13.6 Hz, 2H*), 3.85 – 3.72 (m, 1H* + 1H'), 3.62 – 3.47 (m, 1H* + 2H'), 3.37 – 3.22 (m, 1H* + 1H'), 3.10 (dd, J = 17.1, 4.3 Hz, 1H*).

^{13}C NMR (126 MHz, $CDCl_3$) δ 198.5, 198.4, 145.3, 145.0, 142.3, 142.0, 140.3, 139.2, 137.3, 137.2, 133.1, 133.1, 131.6, 131.3, 129.8, 129.8, 129.6, 129.4, 128.9, 128.7, 128.6, 128.5, 128.3, 128.1, 128.1, 128.0, 127.7, 127.5, 127.0, 126.5, 122.4, 122.2, 116.8, 116.7, 57.3, 55.6, 46.1, 45.5, 43.3, 43.2.

HRMS (ESI) calcd for $C_{24}H_{22}OBr$ $[M+H]^+$: 405.0849, found: 405.0852.

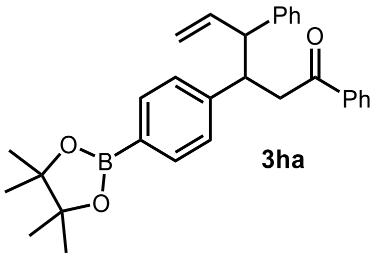

3ha

1,4-Diphenyl-3-(4-(3,3,4,4-tetramethyl-1l3,2,5-bromadioxolan-1-yl)phenyl)hex-5-en-1-one (**3ha**): (SGS-4-28) Prepared following **General Procedure A** using 1-phenyl-3-(4-(4,4,5,5-tetramethyl-1,3,2-dioxaborolan-2-yl)phenyl)prop-2-en-1-one (**1h**, 100.3 mg, 0.3 mmol, 1.0 equiv) and allylbenzene (**2a**, 119 μL, 0.9 mmol, 3.0 equiv). The reaction mixture was heated at 80 °C for 16 h. The crude reaction mixture was purified by flash column chromatography (hexanes/ethyl acetate = 100:1 to 20:1) to afford the title compound as a white solid (51.5 mg, 40%). The diastereomeric ratio (dr 1.3:1) was determined by NMR analysis of the crude product.

^{1}H NMR (500 MHz, $CDCl_3$) δ 7.87 – 7.03 (m, 18H* + 18H'), 6.18 – 5.99 (m, 1H'), 5.96 – 5.76 (m, 1H*), 5.12 (dd, *J* = 37.5, 13.4 Hz, 2H'), 4.83 (dd, *J* = 26.5, 13.6 Hz, 2H*), 3.96 – 3.73 (m, 1H* +1H'), 3.71 – 3.48 (m, 1H* + 2H'), 3.41 – 3.24 (m, 1H* + 1H'), 3.10 (dd, *J* = 16.8, 4.3 Hz, 1H*), 1.33 (s, 12H*), 1.29 (s, 12H').

^{13}C NMR (126 MHz, $CDCl_3$) δ 198.9, 198.8, 146.2, 145.8, 142.7, 142.4, 140.8, 139.54, 137.49, 137.3, 134.8, 134.6, 133.0, 132.9, 128.9, 128.6, 128.5, 128.4, 128.4, 128.3, 128.2, 128.10, 128.05, 128.0, 126.9, 126.3, 116.44, 116.35, 83.8, 83.7, 57.2, 55.7, 46.7, 45.9, 43.6, 43.4, 25.04, 25.02, 25.01 (five carbons missing due to overlap).

HRMS (ESI) calcd for $C_{30}H_{34}O_3B$ $[M+H]^+$: 453.2596, found: 453.2596.

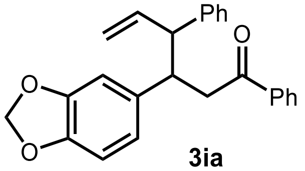

3ia

3-(Benzo[*d*][1,3]dioxol-5-yl)-1,4-diphenylhex-5-en-1-one (**3ia**): (SGS-4-6) Prepared following **General Procedure A** using 1-(benzo[d][1,3]dioxol-5-yl)-3-phenylprop-2-en-1-one (**1i**, 75.7 mg, 0.3 mmol, 1.0 equiv) and allylbenzene (**2a**, 119 μL, 0.9 mmol, 3.0 equiv). The reaction mixture was heated at 80 °C for 16 h. The crude reaction mixture was purified by flash column chromatography (hexanes/ethyl ether = 100:1) to afford the title compound as a white solid (89.3 mg, 80%). The diastereomeric ratio (dr 1.3:1) was determined by NMR analysis of the crude product.

^{1}H NMR (500 MHz, $CDCl_3$) δ 7.85 (d, *J* = 7.0 Hz, 1H'), 7.71 (d, *J* = 7.1 Hz, 1H*), 7.61 – 6.97 (m, 6H), 6.77 – 6.44 (m, 2H), 6.17 – 5.98 (m, 1H'), 5.96 – 5.70 (m, 3H*+2H'), 5.11 (dd, *J* = 57.6, 13.5 Hz, 2H'), 4.85 (dd, *J* = 51.3, 13.6 Hz, 2H*), 3.82 – 3.63 (m, 1H*+1H'), 3.59 – 3.40 (m, 1H* = 2H'), 3.34 – 3.15 (m, 1H* = 1H'), 3.02 (d, *J* = 16.6 Hz, 1H*).

^{13}C NMR (126 MHz, $CDCl_3$) δ 199.01, 198.96, 147.6, 147.3, 146.1, 145.8, 142.8, 142.5, 140.8, 139.7, 137.5, 137.4, 136.6, 136.3, 133.0, 132.9, 128.9, 128.6, 128.5, 128.4, 128.2, 128.11, 128.06, 126.9, 126.3, 122.0, 121.8, 116.3, 108.8, 108.7, 108.1, 107.9, 100.9, 100.8, 57.6, 56.0, 46.4, 45.6, 43.8, 43.7 (two carbons missing due to overlap).

HRMS (ESI) calcd for $C_{25}H_{23}O_3$ [M+H]$^+$: 371.1642, found: 371.1638.

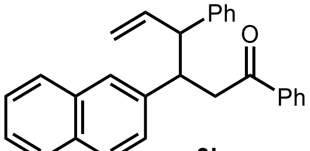

3ja

3-(Naphthalen-2-yl)-1,4-diphenylhex-5-en-1-one (**3ja**): (SGS-4-31) Prepared following **General Procedure A** using 1-(naphthalen-2-yl)-3-phenylprop-2-en-1-one (**1j**, 77.5 mg, 0.3 mmol, 1.0 equiv) and allylbenzene (**2a**, 119 μL, 0.9 mmol, 3.0 equiv). The reaction mixture was heated at 80 °C for 16 h. The crude reaction mixture was purified by flash column chromatography (hexanes/ethyl acetate = 100:1 to 50:1) to afford the title compound as a white solid (98.4 mg, 87%). The diastereomeric ratio (dr 1.4:1) was determined by NMR analysis of the crude product.

^{1}H NMR (500 MHz, $CDCl_3$) δ 7.90 – 6.98 (m, 17H* + 17H'), 6.19 – 6.08 (m, 1H*), 5.93 – 5.82 (m, 1H'), 5.27 – 5.06 (m, 2H*), 4.82 (dd, *J* = 24.1, 13.6 Hz, 2H'), 4.04 (td, *J* = 9.1, 5.0 Hz, 1H*), 3.97 (td, *J* = 9.5, 4.2 Hz, 1H'), 3.79 – 3.66 (m, 1H* + 1H'), 3.61 (dd, *J* = 17.2, 4.9 Hz, 1H*), 3.50 – 3.37 (m, 1H* + 1H'), 3.18 (dd, *J* = 16.9, 4.2 Hz, 1H').

^{13}C NMR (126 MHz, $CDCl_3$) δ 199.0, 198.9, 142.8, 142.4, 140.8, 140.4, 140.0, 139.7, 137.5, 137.3, 133.5, 133.4, 133.0, 132.9, 132.5, 132.2, 128.8, 128.6, 128.51, 128.46, 128.4, 128.2, 128.1, 128.0, 127.9, 127.9, 127.8, 127.70, 127.65, 127.6, 127.3, 126.9, 126.3, 125.9, 125.7, 125.5, 125.3, 116.4, 57.3, 55.8, 46.6, 45.8, 43.7, 43.6 (four carbons missing due to overlap).

HRMS (ESI) calcd for $C_{28}H_{25}O$ [M+H]$^+$: 377.1900, found: 377.1896.

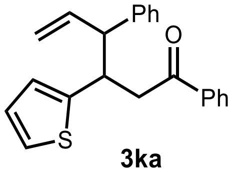

3ka

1,4-Diphenyl-3-(thiophen-2-yl)hex-5-en-1-one (**3ka**): (SGS-4-21) Prepared following **General Procedure A** using 1-phenyl-3-(thiophen-2-yl)prop-2-en-1-one (**1k**, 64.3 mg, 0.3 mmol, 1.0 equiv) and allylbenzene (**2a**, 119 μL, 0.9 mmol, 3.0 equiv). The reaction mixture was heated at 80 °C for 16 h. The crude reaction mixture was purified by flash column chromatography (hexanes/ethyl ether = 100:1) to afford the title compound as a tan solid (71.2 mg, 71%). The diastereomeric ratio (dr 1.4:1) was determined by NMR analysis of the crude product.

^{1}H NMR (500 MHz, $CDCl_3$) δ 7.89 (d, *J* = 7.6 Hz, 2H'), 7.78 (d, *J* = 7.6 Hz, 2H*), 7.60 – 7.07 (m, 13H), 6.91 – 6.75 (m, 2H*), 6.72 – 6.55 (m, 2H'), 6.13 – 6.04 (m, 1H'), 6.03 – 5.93 (m, 1H*), 5.14 (dd, *J* = 57.2, 13.5 Hz, 2H'), 4.95 (dd, *J* = 43.3, 13.6 Hz, 2H*), 4.24 – 4.11 (m, 1H* + 1H'), 3.63 (t, *J* = 8.7 Hz, 1H*), 3.57 (t, *J* = 9.5 Hz, 1H'), 3.50 (dd, *J* = 17.1, 4.8 Hz, 1H'), 3.44 – 3.27 (m, 1H* + 1H'), 3.12 (dd, *J* = 16.9, 4.5 Hz, 1H*).
^{13}C NMR (126 MHz, $CDCl_3$) δ 198.5, 198.4, 146.3, 145.9, 142.5, 142.3, 140.3, 139.0, 137.3, 137.2, 133.1, 133.0, 128.8, 128.6, 128.5, 128.4, 128.2, 128.12, 128.09, 128.0, 126.9, 126.5, 126.5, 126.3, 125.7, 125.3, 123.5, 123.1, 116.9, 116.8, 58.1, 56.2, 44.4, 44.3, 42.0, 41.3.
HRMS (ESI) calcd for $C_{22}H_{21}OS$ $[M+H]^+$: 333.1308, found: 333.1318.

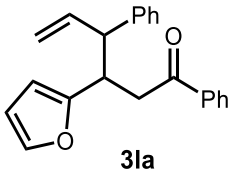

3la

3-(Furan-2-yl)-1,4-diphenylhex-5-en-1-one (**3la**): (SGS-4-29) Prepared following **General Procedure A** using (E)-3-(furan-2-yl)-1-phenylprop-2-en-1-one (**1l**, 59.4 mg, 0.3 mmol, 1.0 equiv) and allylbenzene (**2a**, 119 μL, 0.9 mmol, 3.0 equiv). The reaction mixture was heated at 80 °C for 16 h. The crude reaction mixture was purified by flash column chromatography (hexanes/ethyl ether = 50:1) to afford the title compound as a tan solid (37.7 mg, 40%). The diastereomeric ratio (dr 1.2:1) was determined by NMR analysis of the crude product.
1H NMR (500 MHz, CDCl3) δ 7.84 – 7.79 (m, 2H'), 7.74 – 7.68 (m, 2H*), 7.49 – 6.97 (m, 9H* + 9H'), 6.13 (dd, *J* = 3.0, 1.8 Hz, 1H*), 6.06 – 5.96 (m, 2H'), 5.94 – 5.85 (m, 2H*), 5.68 (d, *J* = 3.1 Hz, 1H'), 5.15 – 4.99 (m, 2H'), 4.87 (dd, J = 31.1, 13.6 Hz, 2H*), 3.91 – 3.81 (m, 1H* + 1H'), 3.62 (dd, J = 19.5, 9.3 Hz, 1H* + 1H'), 3.42 – 3.22 (m, 1H* + 2H'), 2.93 (dd, J = 16.8, 4.4 Hz, 1H*).
^{13}C NMR (126 MHz, $CDCl_3$) δ 198.8, 198.7, 155.4, 142.4, 142.4, 141.2, 140.9, 140.0, 139.4, 137.3, 137.2, 133.1, 133.1, 128.8, 128.7, 128.6, 128.4, 128.2, 128.2, 128.1, 128.0, 126.9, 126.5, 116.8, 116.3, 110.11, 110.06, 107.4, 107.0, 55.0, 54.1, 41.0, 40.7, 40.1, 39.9 (one carbon missing due to overlap).
HRMS (ESI) calcd for $C_{22}H_{21}O_2$ $[M+H]^+$: 317.1536, found: 317.1537.

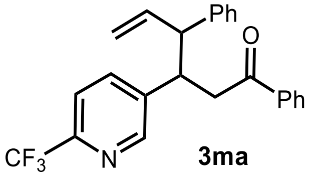

3ma

1,4-Diphenyl-3-(6-(trifluoromethyl)pyridin-3-yl)hex-5-en-1-one (**3ma**): (SGS-4-51) Prepared following **General Procedure A** using 1-phenyl-3-(6-(trifluoromethyl)pyridin-3-yl)prop-2-en-1-one (**1m**, 83.2 mg, 0.3 mmol, 1.0 equiv) and allylbenzene (**2a**, 119 μL, 0.9 mmol, 3.0 equiv). The reaction mixture was heated at 80 °C for 16 h. The crude reaction mixture was purified by flash column chromatography (hexanes/ethyl acetate = 100:1 to 20:1) to afford the title compound as a white solid (93.7 mg, 79%). The diastereomeric ratio (dr 1.2:1) was determined by NMR analysis of the crude product.
^{1}H NMR (500 MHz, $CDCl_3$) δ 8.58 (d, *J* = 1.5 Hz, 1H), 8.47 (d, *J* = 1.6 Hz, 1H), 7.89 – 7.85 (m, 2H), 7.76 – 7.71 (m, 2H), 7.69 (dd, *J* = 8.0, 1.8 Hz, 1H), 6.13 – 6.04 (m, 1H), 5.79 (ddd, *J* = 17.0, 10.1, 9.0 Hz, 1H), 5.29 – 5.14 (m, 1H), 4.90 (dd, *J* = 42.4, 13.6 Hz, 2H), 3.97 – 3.87 (m, 2H), 3.69 – 3.55 (m, 3H), 3.47 – 3.29 (m, 2H), 3.21 (dd, *J* = 17.5, 4.1 Hz, 1H).
^{13}C NMR (126 MHz, CH_2Cl_2) δ 197.8, 197.7, 150.5, 150.1, 146.5 (q, *J* = 34.7 Hz), 141.9, 141.7, 141.6, 141.3, 139.6, 138.5, 137.6, 137.4, 136.9, 136.7, 133.5, 133.4, 129.2, 128.8, 128.7, 128.1, 128.04, 127.98, 127.9, 127.4, 127.0, 124.6 (q, *J* = 445.7 Hz), 124.5 (q, *J* = 441.2 Hz), 120.0, 119.8, 117.6, 117.5, 56.7, 55.4, 43.9, 43.4, 42.8, 42.7 (two carbons missing due to overlap).

^{19}**F NMR** (471 MHz, $CDCl_3$) δ -67.67, -67.75.
HRMS (ESI) calcd for $C_{24}H_{21}ONF_3$ $[M+H]^+$: 396.1570, found: 396.1572.

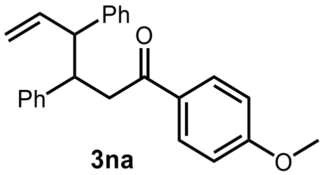

3na

1-(4-Methoxyphenyl)-3,4-diphenylhex-5-en-1-one (**3na**): (SGS-4-23) Prepared following **General Procedure A** using 1-(4-methoxyphenyl)-3-phenylprop-2-en-1-one (**1n**, 71.5 mg, 0.3 mmol, 1.0 equiv) and allylbenzene (**2a**, 119 µL, 0.9 mmol, 3.0 equiv). The reaction mixture was heated at 80 °C for 16 h. The crude reaction mixture was purified by flash column chromatography (hexanes/ethyl acetate = 100:1) to afford the title compound as an off white solid (74.7 mg, 70%). The diastereomeric ratio (dr 1.4:1) was determined by NMR analysis of the crude product.
^{1}**H NMR** (500 MHz, $CDCl_3$) δ 7.91 – 7.81 (m, 2H'), 7.75 – 7.69 (m, 2H*), 7.52 (t, *J* = 7.4 Hz, 1H'), 7.48 (t, *J* = 7.4 Hz, 1H*), 7.44 – 6.99 (m, 9H*+ 7H'), 6.97 – 6.92 (m, 2H'), 6.82 – 6.73 (m, 2H*), 6.66 – 6.60 (m, 2H'), 6.13 – 6.04 (m, 1H'), 5.85 (ddd, *J* = 17.1, 10.2, 8.3 Hz, 1H*), 5.12 (ddd, *J* = 13.5, 11.6, 1.3 Hz, 2H'), 4.95 – 4.75 (m, 2H*), 3.82 – 3.65 (m, 4H* + 4H'), 3.62 – 3.45 (m, 1H* + 2H'), 3.35 – 3.19 (m, 1H* + 1H'), 3.08 (dd, *J* = 16.7, 4.5 Hz, 1H*).
^{13}**C NMR** (126 MHz, $CDCl_3$) δ 199.2, 199.1, 158.2, 157.8, 142.9, 142.6, 140.9, 139.7, 137.5, 137.4, 134.7, 134.3, 132.9, 132.8, 129.6, 129.4, 128.8, 128.6, 128.5, 128.4, 128.3, 128.2, 128.1, 128.0, 126.7, 126.2, 116.3, 116.2, 113.6, 113.4, 57.5, 55.8, 55.2, 55.1, 45.8, 45.1, 43.70, 43.65.
HRMS (ESI) calcd for $C_{25}H_{25}O_2$ $[M+H]^+$: 357.1849, found: 357.1854.

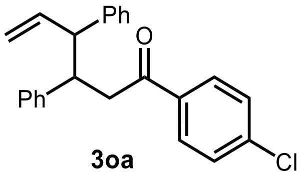

3oa

1-(4-Chlorophenyl)-3,4-diphenylhex-5-en-1-one (**3oa**): (SGS-4-4) Prepared following **General Procedure A** using 1-(4-chlorophenyl)-3-phenylprop-2-en-1-one (**1o**, 72.8 mg, 0.3 mmol, 1.0 equiv) and allylbenzene (**2a**, 119 µL, 0.9 mmol, 3.0 equiv). The reaction mixture was heated at 80 °C for 16 h. The crude reaction mixture was purified by flash column chromatography (hexanes/ethyl ether = 100:1) to afford the title compound as a white solid (92.0 mg, 85%). The diastereomeric ratio (dr 1.4:1) was determined by NMR analysis of the crude product.
^{1}**H NMR** (500 MHz, $CDCl_3$) δ 7.75 (d, *J* = 8.4 Hz, 2H'), 7.60 (d, *J* = 8.4 Hz, 2H*), 7.37 – 6.91 (m, 12H*+12H'), 6.12 – 6.01 (m, 1H'), 5.87 – 5.76 (m, 1H*), 5.10 (dd, *J* = 59.9, 13.4 Hz, 2H'), 4.81 (dd, *J* = 48.5, 13.6 Hz, 2H*), 3.83 – 3.76 (m, 1H'), 3.76 – 3.68 (m, 1H*), 3.60 (t, *J* = 8.9 Hz, 1H*), 3.53 (t, *J* = 9.8 Hz, 1H'), 3.46 (dd, *J* = 16.9, 4.9 Hz, 1H'), 3.32– 3.18 (m, 1H*+1H'), 3.03 (dd, *J* = 16.6, 4.3 Hz, 1H*).
^{13}**C NMR** (126 MHz, $CDCl_3$) δ 197.9, 197.8, 142.7, 142.5, 142.4, 142.2, 140.7, 139.6, 139.34, 139.26, 135.8, 135.6, 129.5, 129.4, 128.9, 128.82, 128.77, 128.7, 128.44, 128.38, 128.31, 128.30, 128.14, 128.08, 126.9, 126.7, 126.31, 126.27, 116.40, 116.37, 57.4, 55.8, 46.7, 46.0, 43.53, 43.51.
HRMS (ESI) calcd for $C_{24}H_{22}OCl$ $[M+H]^+$: 361.1354, found: 361.1354.

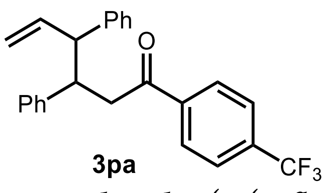

3pa

3,4-Diphenyl-1-(4-(trifluoromethyl)phenyl)hex-5-en-1-one (**3pa**): (SGS-4-5) Prepared following **General Procedure A** using 3-phenyl-1-(4-(trifluoromethyl)phenyl)prop-2-en-1-one (**1p**, 82.9 mg, 0.3 mmol, 1.0 equiv) and allylbenzene (**2a**, 119 µL, 0.9 mmol, 3.0 equiv). The reaction mixture was heated at 80 °C for 16 h. The crude reaction mixture was purified by flash column chromatography (hexanes/ethyl acetate = 100:1) to afford the title compound as a white solid (103.0 mg, 87%). The diastereomeric ratio (dr 1.5:1) was determined by NMR analysis of the crude product.

^{1}H NMR (500 MHz, $CDCl_3$) δ 7.94 (d, *J* = 8.2 Hz, 2H'), 7.78 (d, *J* = 8.2 Hz, 2H*), 7.68 (d, *J* = 8.2 Hz, 2H'), 7.62 (d, *J* = 8.3 Hz, 2H*), 7.38 – 7.00 (m, 10H*+10H'), 6.15 – 6.06 (m, 1H'), 5.85 (ddd, *J* = 17.2, 10.2, 8.2 Hz, 1H*), 5.15 (dd, *J* = 59.5, 13.5 Hz, 2H'), 4.85 (dd, *J* = 51.0, 13.6 Hz, 2H*), 3.88 – 3.80 (m, 1H'), 3.79 – 3.73 (m, 1H*), 3.67 – 3.51 (m, 1H*+2H'), 3.42 – 3.25 (m, 1H*+1H'), 3.12 (dd, *J* = 16.7, 4.4 Hz, 1H*).

^{13}C NMR (126 MHz, $CDCl_3$) δ 198.3, 198.2, 142.6, 142.4, 142.3, 142.1, 140.7, 140.2, 140.0, 139.5, 134.3 (q, *J* = 32.7 Hz) 134.2 (q, *J* = 32.5 Hz) 128.9, 128.7, 128.5, 128.4, 128.40, 128.37, 128.3, 128.2, 128.2, 127.0, 126.8, 126.44, 126.36, 125.7 (q, *J* = 3.7 Hz), 125.6 (q, *J* = 3.7 Hz), 123.8 (q, *J* = 272.5 Hz), 123.7 (q, *J* = 272.7 Hz), 116.53, 116.46, 57.5, 55.8, 46.7, 46.0, 44.00, 43.98 (one carbon missing due to overlap).

19F NMR (471 MHz, CDCl3) δ -63.09, -63.12.

HRMS (ESI) calcd for $C_{25}H_{22}OF_3$ $[M+H]^+$: 395.1617, found: 395.1613.

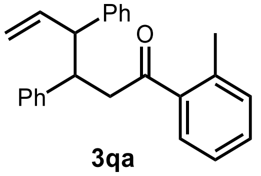

3qa

3,4-Diphenyl-1-(o-tolyl)hex-5-en-1-one (**3qa**): (SGS-4-33) Prepared following **General Procedure A** using 3-phenyl-1-(o-tolyl)prop-2-en-1-one (**1q**, 66.7 mg, 0.3 mmol, 1.0 equiv) and allylbenzene (**2a**, 119 µL, 0.9 mmol, 3.0 equiv). The reaction mixture was heated at 80 °C for 16 h. The crude reaction mixture was purified by flash column chromatography (hexanes/ethyl ether = 100:1) to afford the title compound as a white solid (90.8 mg, 89%). The diastereomeric ratio (dr 1.3:1) was determined by NMR analysis of the crude product.

^{1}H NMR (500 MHz, $CDCl_3$) δ 7.49 – 6.98 (m, 14H* + 14H'), 6.19 – 6.09 (m, 1H'), 5.85 (ddd, *J* = 17.2, 10.2, 8.2 Hz, 1H*), 5.26 – 5.09 (m, 2H'), 4.84 (dd, *J* = 49.4, 13.6 Hz, 2H*), 3.76 (td, *J* = 9.5, 5.0 Hz, 1H'), 3.69 (td, *J* = 9.8, 4.5 Hz, 1H*), 3.59 (t, *J* = 9.0 Hz, 1H*), 3.56 – 3.45 (m, 2H'), 3.27 (dd, *J* = 16.7, 9.1 Hz, 1H'), 3.15 (dd, *J* = 16.4, 9.8 Hz, 1H*), 3.05 (dd, *J* = 16.4, 4.5 Hz, 1H*), 2.20 (s, 3H'), 2.09 (s, 3H*).

^{13}C NMR (126 MHz, $CDCl_3$) δ 203.72, 203.67, 142.7, 142.5, 142.3, 142.1, 140.7, 139.7, 138.9, 138.7, 137.7, 137.7, 131.8, 131.7, 130.9, 130.9, 128.83, 128.80, 128.5, 128.33, 128.29, 128.27, 128.1, 128.0, 127.9, 127.8, 126.8, 126.7, 126.3, 126.2, 125.5, 125.4, 116.4, 116.2, 57.6, 56.0, 47.1, 47.0, 46.9, 46.4, 20.6, 20.4.

HRMS (ESI) calcd for $C_{25}H_{25}O$ $[M+H]^+$: 341.1899, found: 341.1899.

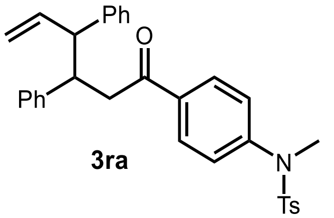

3ra

N-(4-(3,4-diphenylhex-5-enoyl)phenyl)-N,4-dimethylbenzenesulfonamide (**3ra**): (SGS-4-34) Prepared following **General Procedure A** using N,4-dimethyl-N-(4-(3-phenylacryloyl)phenyl)benzenesulfonamide (**1r**,

117.4 mg, 0.3 mmol, 1.0 equiv) and allylbenzene (**2a**, 119 µL, 0.9 mmol, 3.0 equiv). The reaction mixture was heated at 80 °C for 16 h. The crude reaction mixture was purified by flash column chromatography (hexanes/ethyl acetate = 20:1) to afford the title compound as a white foam (117.5 mg, 77%). The diastereomeric ratio (dr 1.3:1) was determined by NMR analysis of the crude product.
^{1}H NMR (500 MHz, $CDCl_3$) δ 7.79 (d, *J* = 8.6 Hz, 2H'), 7.65 (d, *J* = 8.6 Hz, 2H*), 7.42 – 6.99 (m, 16H* + 16H'), 6.14 – 6.04 (m, 1H'), 5.84 (ddd, *J* = 17.3, 10.1, 8.4 Hz, 1H*), 5.24 – 5.04 (m, 2H'), 4.83 (dd, *J* = 50.2, 13.7 Hz, 2H*), 3.86 – 3.73 (m, 1H'+1H*), 3.62 (t, *J* = 9.0 Hz, 1H'), 3.55 (t, *J* = 9.8 Hz, 1H*), 3.49 (dd, *J* = 17.1, 4.8 Hz, 1H'), 3.39 – 3.24 (m, 1H* + 1H'), 3.16 (s, 3H*), 3.13 (s, 3H'), 3.05 (dd, *J* = 16.8, 4.2 Hz, 1H*), 2.41 (s, 3H*), 2.40 (s, 3H').
^{13}C NMR (126 MHz, $CDCl_3$) δ 198.1, 198.0, 145.7, 145.7, 144.08, 144.06, 142.7, 142.6, 142.43, 142.39, 140.7, 139.7, 135.5, 135.4, 133.38, 133.35, 129.7, 129.6, 128.9, 128.79, 128.77, 128.73, 128.5, 128.4, 128.3, 128.2, 128.1, 127.84, 127.82, 126.9, 126.7, 126.29, 126.28, 125.7, 125.6, 116.44, 116.35, 57.4, 55.8, 46.6, 45.9, 43.59, 43.55, 37.69, 37.66, 21.7 (two carbons missing due to overlap).
HRMS (ESI) calcd for $C_{32}H_{32}O_3NS$ [M+H]$^+$: 510.2097, found: 510.2100.

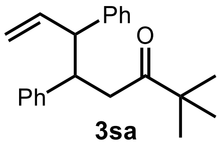

3sa

2,2-Dimethyl-5,6-diphenyloct-7-en-3-one (**3sa**): (SGS-4-52) Prepared following **General Procedure A** using 4,4-dimethyl-1-phenylpent-1-en-3-one (**1s**, 56.5 mg, 0.3 mmol, 1.0 equiv) and allylbenzene (**2a**, 119 µL, 0.9 mmol, 3.0 equiv). The reaction mixture was heated at 80 °C for 16 h. The crude reaction mixture was purified by flash column chromatography (hexanes/ethyl ether = 100:1) to afford the title compound as a white solid (56.7 mg, 62%). The diastereomeric ratio (dr 1.2:1) was determined by NMR analysis of the crude product.
^{1}H NMR (500 MHz, $CDCl_3$) δ p7.33 – 6.96 (m, 10H* +10H'), 6.08 – 6.00 (m, 1H'), 5.80 (ddd, *J* = 17.1, 10.1, 8.3 Hz, 1H*), 5.21 – 5.03 (m, 2H'), 4.88 – 4.75 (m, 2H*), 3.70 – 3.62 (m, 1H* + 1H'), 3.56 (t, *J* = 9.0 Hz, 1H*), 3.48 (t, *J* = 10.0 Hz, 1H'), 3.00 – 2.83 (m, 2H'+ 1H*), 2.50 (dd, *J* = 17.2, 4.3 Hz, 1H*), 0.97 (s, 9H'), 0.85 (s, 9H*).
^{13}C NMR (126 MHz, $CDCl_3$) δ 214.3, 214.2, 143.1, 142.92, 142.85, 142.8, 141.2, 140.0, 128.9, 128.7, 128.6, 128.4, 128.3, 128.2, 128.1, 128.0, 126.7, 126.5, 126.14, 126.09, 116.05, 116.0, 57.1, 55.3, 45.8, 45.2, 44.3, 44.2, 42.1, 41.8, 26.3, 26.0.
HRMS (ESI) calcd for $C_{22}H_{27}O$ [M+H]$^+$: 307.2056, found: 307.2054.

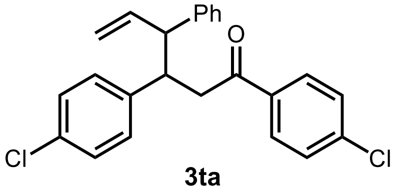

3ta

1,3-Bis(4-chlorophenyl)-4-phenylhex-5-en-1-one (**3ta**): (SGS-4-55) Prepared following **General Procedure A** using 1,3-bis(4-chlorophenyl)prop-2-en-1-one (**1t**, 83.1 mg, 0.3 mmol, 1.0 equiv) and allylbenzene (**2a**, 119 µL, 0.9 mmol, 3.0 equiv). The reaction mixture was heated at 80 °C for 16 h. The crude reaction mixture was purified by flash column chromatography (hexanes/ethyl ethyl acetate = 100:1) to afford the title compound as a white solid (103.9 mg, 87%). The diastereomeric ratio (dr 1.3:1) was determined by NMR analysis of the crude product.
^{1}H NMR (500 MHz, $CDCl_3$) δ 7.97 (d, *J* = 8.5 Hz, 2H'), 7.80 – 7.76 (m, 2H'), 7.65 (d, *J* = 8.6 Hz, 2H*), 7.58 (d, *J* = 8.5 Hz, 2H'), 7.52 – 6.97 (m, 11H* + 7H'), 6.11 – 6.02 (m, 1H'), 5.82 (ddd, *J* = 17.2, 10.1, 8.5 Hz, 1H*), 5.22

– 5.07 (m, 2H'), 4.86 (dd, J = 52.6, 13.6 Hz, 2H*), 3.80 (td, J = 9.4, 4.7 Hz, 1H'), 3.74 (td, J = 9.5, 4.3 Hz, 1H*), 3.62 – 3.46 (m, 1H* + 2H'), 3.33 – 3.17 (m, 1H* + 1H'), 3.06 (dd, J = 16.8, 4.3 Hz, 1H*).
^{13}C NMR (126 MHz, $CDCl_3$) δ 197.5, 197.42, 142.36, 142.0, 141.1, 140.8, 140.4, 139.6, 139.5, 139.2, 135.6, 135.44, 132.36, 132.0, 130.0, 129.8, 129.5, 129.4, 129.0, 128.9, 128.9, 128.5, 128.3, 128.1, 127.0, 126.5, 116.7, 116.7, 57.3, 55.7, 46.0, 45.3, 43.4, 43.3 (two carbons missing due to overlap).
HRMS (ESI) calcd for $C_{24}H_{21}OCl_2$ $[M+H]^+$: 395.0964, found: 395.0964.

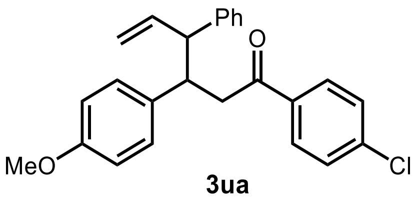
3ua

3-(4-Chlorophenyl)-1-(4-methoxyphenyl)-4-phenylhex-5-en-1-one (3ua): (SGS-4-56) Prepared following **General Procedure A** using 1-(4-chlorophenyl)-3-(4-methoxyphenyl)prop-2-en-1-one (**1u**, 81.8 mg, 0.3 mmol, 1.0 equiv) and allylbenzene (**2a**, 119 µL, 0.9 mmol, 3.0 equiv). The reaction mixture was heated at 80 °C for 16 h. The crude reaction mixture was purified by flash column chromatography (hexanes/ethyl acetate = 50:1) to afford the title compound as a white solid (86.9 mg, 75%). The diastereomeric ratio (dr 1.2:1) was determined by NMR analysis of the crude product.
^{1}H NMR (500 MHz, $CDCl_3$) δ 7.79 (d, J = 8.4 Hz, 2H'), 7.65 (d, J = 8.5 Hz, 2H*), 7.38 (d, J = 8.5 Hz, 2H'), 7.36 – 7.01 (m, 9fH* + 5H'), 6.95 (d, J = 8.2 Hz, 2H'), 6.80 (d, J = 8.3 Hz, 2H*), 6.64 (d, J = 8.4 Hz, 2H'), 6.14 – 6.04 (m, 1H'), 5.86 (dt, J = 18.2, 9.2 Hz, 1H*), 5.13 (dd, J = 56.9, 13.5 Hz, 2H'), 4.86 (dd, J = 52.1, 13.6 Hz, 2H*), 3.82 – 3.65 (m, 4H* + 4H'), 3.63 – 3.42 (m, 1H* + 2H'), 3.33 – 3.14 (m, 1H* + 1H'), 3.05 (dd, J = 16.5, 4.3 Hz, 1H*).
^{13}C NMR (126 MHz, $CDCl_3$) δ 198.1, 198.0, 158.2, 157.9, 142.8, 142.5, 140.8, 139.6, 139.32, 139.25, 135.8, 135.7, 134.4, 134.1, 129.6, 129.5, 129.5, 129.3, 128.9, 128.8, 128.8, 128.4, 128.3, 128.2, 126.8, 126.2, 116.3, 116.3, 113.7, 113.5, 57.5, 55.9, 55.2, 55.1, 46.0, 45.2, 43.7 (one carbon missing due to overlap).
HRMS (ESI) calcd for $C_{25}H_{24}O_2Cl$ $[M+H]^+$: 391.1459, found: 391.1455.

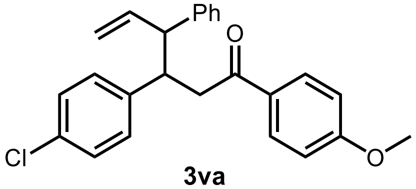
3va

1-(4-Chlorophenyl)-3-(4-methoxyphenyl)-4-phenylhex-5-en-1-one (3va): (SGS-4-57) Prepared following **General Procedure A** using 3-(4-chlorophenyl)-1-(4-methoxyphenyl)prop-2-en-1-one (**1v**, 81.8 mg, 0.3 mmol, 1.0 equiv) and allylbenzene (**2a**, 119 µL, 0.9 mmol, 3.0 equiv). The reaction mixture was heated at 80 °C for 16 h. The crude reaction mixture was purified by flash column chromatography (hexanes/ethyl acetate = 50:1) to afford the title compound as a white solid (78.9 mg, 67%). The diastereomeric ratio (dr 1.3:1) was determined by NMR analysis of the crude product.
^{1}H NMR (400 MHz, $CDCl_3$) δ 7.86 – 7.81 (m, 2H'), 7.74 – 7.67 (m, 2H*), 7.35 – 6.93 (m, 23H), 6.91 – 6.85 (m, 2H'), 6.85 – 6.79 (m, 2H*), 6.12 – 6.00 (m, 1H'), 5.80 (ddd, J = 17.0, 10.2, 8.5 Hz, 1H*), 5.12 (ddd, J = 13.5, 11.5, 1.2 Hz, 2H'), 4.94 – 4.73 (m, H2*), 3.87 – 3.70 (m, 4H* + 4H'), 3.61 – 3.39 (m, 1H* + 2H'), 3.32 – 3.14 (m, 1H* + 1H'), 3.03 (dd, J = 16.7, 4.4 Hz, 1H*).
^{13}C NMR (126 MHz, $CDCl_3$) δ 197.2, 197.1, 163.52, 163.47, 142.5, 142.2, 141.4, 141.1, 140.5, 139.3, 132.1, 131.8, 130.4, 130.32, 130.27, 130.1, 129.83, 128.84, 128.41, 128.36, 128.3, 128.2, 128.1, 126.9, 126.4, 116.6, 116.5, 113.8, 113.7, 57.3, 55.7, 55.6, 55.5, 46.1, 45.4, 43.0, 42.8 (one carbon missing due to overlap).
HRMS (ESI) calcd for $C_{25}H_{24}O_2Cl$ $[M+H]^+$: 391.1459, found: 391.1448.

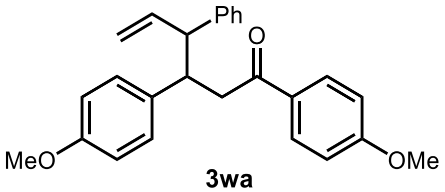

3wa

1,3-Bis(4-methoxyphenyl)-4-phenylhex-5-en-1-one (3wa): (SGS-4-27) Prepared following **General Procedure A** using 1,3-bis(4-methoxyphenyl)prop-2-en-1-one (**1w**, 80.5 mg, 0.3 mmol, 1.0 equiv) and allylbenzene (**2a**, 119 µL, 0.9 mmol, 3.0 equiv). The reaction mixture was heated at 80 °C for 16 h. The crude reaction mixture was purified by flash column chromatography (hexanes/ethyl acetate = 50:1 to 20:1) to afford the title compound as a white solid (73.0 mg, 63%). The diastereomeric ratio (dr 1.1:1) was determined by NMR analysis of the crude product.

^{1}H NMR (500 MHz, $CDCl_3$) δ 7.85 (d, *J* = 8.9 Hz, 2H'), 7.72 (d, *J* = 8.9 Hz, 2H*), 7.35 – 6.99 (m, 7H* + 5H'), 6.94 (d, *J* = 8.7 Hz, 2H'), 6.88 (d, *J* = 8.9 Hz, 2H'), 6.83 (d, *J* = 8.9 Hz, 2H*), 6.77 (d, *J* = 8.6 Hz, 2H*), 6.63 (d, *J* = 8.7 Hz, 2H'), 6.13 – 6.04 (m, 1H'), 5.85 (ddd, *J* = 17.1, 10.2, 8.4 Hz, 1H*), 5.11 (ddd, *J* = 13.5, 11.5, 1.2 Hz, 2H'), 4.93 – 4.76 (m, 2H*), 3.85 (s, 3H'), 3.82 (s, 3H*), 3.79 – 3.75 (m, 4H*), 3.73 – 3.67 (m, 4H'), 3.58 (t, *J* = 8.9 Hz, 1H*), 3.52 (t, *J* = 9.6 Hz, 1H'), 3.42 (dd, *J* = 16.7, 4.9 Hz, 1H'), 3.29 – 3.15 (m, 1H* + 1H'), 3.02 (dd, *J* = 16.4, 4.5 Hz, 1H*).

^{13}C NMR (126 MHz, $CDCl_3$) δ 197.8, 197.7, 163.4, 163.4, 158.2, 157.8, 143.1, 142.7, 141.0, 139.8, 134.8, 134.5, 130.7, 130.5, 130.4, 130.3, 129.7, 129.4, 128.8, 128.4, 128.30, 128.28, 126.7, 126.2, 116.3, 116.2, 113.74, 113.66, 113.6, 113.4, 57.5, 55.9, 55.57, 55.55, 55.3, 55.2, 46.0, 45.2, 43.3, 43.2.

HRMS (ESI) calcd for $C_{26}H_{27}O_3$ $[M+H]^+$: 387.1955, found: 387.1968.

2.2 Synthesis of Michael adducts from Fe-catalyzed Michael addition – Alkene Scope

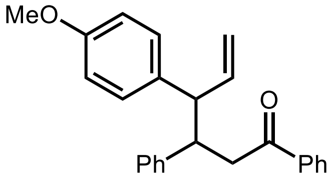

3ab

4-(4-Methoxyphenyl)-1,3-diphenylhex-5-en-1-one (3ab): (SGS-4-7) Prepared following **General Procedure A** using chalcone (**1a**, 62.4 mg, 0.3 mmol, 1.0 equiv) and 4-allylanisole (**2b**, 138 µL, 0.9 mmol, 3.0 equiv). The reaction mixture was heated at 80 °C for 16 h. The crude reaction mixture was purified by flash column chromatography (hexanes/ethyl acetate = 100:1) to afford the title compound as a white solid (87.7 mg, 82%). The diastereomeric ratio (dr 1.3:1) was determined by NMR analysis of the crude product.

^{1}H NMR (500 MHz, $CDCl_3$) δ 7.88 (d, *J* = 7.5 Hz, 2H'), 7.75 (d, *J* = 7.5 Hz, 2H*), 7.53 (t, *J* = 7.4 Hz, 1H'), 7.49 (t, *J* = 7.4 Hz, 1H*), 7.43 (t, *J* = 7.7 Hz, 2H'), 7.37 (t, *J* = 7.7 Hz, 2H*), 7.30 – 7.02 (m, 7H*+5H'), 6.96 (d, *J* = 8.6 Hz, 2H'), 6.88 (d, *J* = 8.6 Hz, 2H*), 6.71 (d, *J* = 8.6 Hz, 2H'), 6.15 – 6.03 (m, 1H'), 5.90 – 5.79 (m, 1H*), 5.12 (dd, *J* = 56.5, 13.4 Hz, 2H'), 4.84 (dd, *J* = 48.1, 13.6 Hz, 2H*), 3.87 – 3.70 (m, 4H'+4H*), 3.65 – 3.49 (m, 1H*+2H'), 3.41 – 3.26 (m, 1H*+1H'), 3.15 (dd, *J* = 16.7, 4.4 Hz, 1H*).

^{13}C NMR (126 MHz, $CDCl_3$) δ 199.1, 158.4, 157.9, 142.8, 142.6, 141.1, 140.0, 137.5, 137.4, 134.8, 134.6, 132.9, 132.9, 129.3, 129.1, 128.8, 128.59, 128.56, 128.5, 128.3, 128.08, 128.05, 128.0, 126.6, 126.2, 116.0, 115.9, 114.2, 113.7, 56.4, 55.4, 55.2, 54.9, 46.7, 45.9, 43.6, 43.6 (one carbon missing due to overlap).

HRMS (ESI) calcd for $C_{25}H_{25}O_2$ $[M+H]^+$: 357.1849, found: 357.1849.

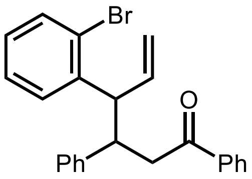

3ac

4-(2-Bromophenyl)-1,3-diphenylhex-5-en-1-one (3ac): (SGS-4-13) Prepared following **General Procedure A** using chalcone (**1a**, 62.4 mg, 0.3 mmol, 1.0 equiv) and 1-allyl-2-bromobenzene (**2c**, 135 μL, 0.9 mmol, 3.0 equiv). The reaction mixture was heated at 80 °C for 16 h. The crude reaction mixture was purified by flash column chromatography (hexanes/ethyl ether = 100:1) to afford the title compound as a white solid (97.6 mg, 80%). The diastereomeric ratio (dr 1.4:1) was determined by NMR analysis of the crude product.

^{1}H NMR (500 MHz, $CDCl_3$) δ 7.81 – 7.74 (m, 2H'), 7.67 – 7.61 (m, 2H*), 7.50 (d, *J* = 7.9 Hz, 1H*), 7.43 (t, *J* = 7.4 Hz, 1H'), 7.38 (t, *J* = 7.4 Hz, 1H*), 7.32 – 6.95 (m, 10H* + 9H'), 6.91 (t, *J* = 7.3 Hz, 1H'), 6.85 – 6.77 (m, 1H'), 5.91 – 5.81 (m, 1H'), 5.77 – 5.65 (m, 1H*), 5.12 (dd, *J* = 106.1, 13.5 Hz, 2H'), 4.79 (dd, *J* = 30.7, 13.6 Hz, 2H*), 4.28 – 4.21 (m, 1H* + 1H'), 3.93 – 3.85 (m, 1H'), 3.73 (td, *J* = 10.1, 3.8 Hz, 1H*), 3.47 (dd, *J* = 17.1, 4.5 Hz, 1H'), 3.38 – 3.31 (m, 1H* + 1H'), 2.91 (dd, *J* = 16.7, 3.9 Hz, 1H*).

^{13}C NMR (126 MHz, $CDCl_3$) δ 199.0, 198.7, 142.39, 142.36, 141.8, 141.5, 139.7, 138.6, 137.5, 137.2, 133.2, 133.0, 132.93, 132.92, 129.4, 129.2, 128.8, 128.6, 128.5, 128.4, 128.4, 128.2, 128.09, 128.05, 128.0, 127.6, 127.5, 126.7, 126.4, 125.6, 124.9, 117.3, 117.1, 54.4, 53.4, 46.4, 44.8, 44.1, 43.2 (one carbon missing due to overlap).

HRMS (ESI) calcd for $C_{24}H_{22}OBr$ $[M+H]^+$: 405.0849, found: 405.0851.

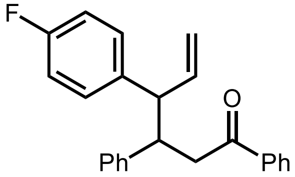

3ad

4-(4-Fluorophenyl)-1,3-diphenylhex-5-en-1-one (3ad): (SGS-4-11) Prepared following **General Procedure A** using chalcone (**1a**, 62.4 mg, 0.3 mmol, 1.0 equiv) and 1-allyl-4-fluorobenzene (**2d**, 121 μL, 0.9 mmol, 3.0 equiv). The reaction mixture was heated at 80 °C for 16 h. The crude reaction mixture was purified by flash column chromatography (hexanes/ethyl acetate = 100:1) to afford the title compound as a white solid (77.4 mg, 75%). The diastereomeric ratio (dr 1.3:1) was determined by NMR analysis of the crude product.

^{1}H NMR (500 MHz, $CDCl_3$) δ 7.82 (d, *J* = 7.6 Hz, 2H'), 7.71 (d, *J* = 7.6 Hz, 2H*), 7.50 – 6.89 (m, 12H* + 10H'), 6.77 (t, *J* = 8.5 Hz, 2H'), 6.07 – 5.97 (m, 1H'), 5.84 – 5.74 (m, 1H*), 5.08 (dd, *J* = 59.4, 13.4 Hz, 2H'), 4.83 (dd, *J* = 57.1, 13.6 Hz, 2H*), 3.82 – 3.68 (m, 1H* + 1H'), 3.62 (t, *J* = 8.6 Hz, 1H*), 3.56 – 3.43 (m, 1H* + 1H'), 3.38 – 3.20 (m, 1H* + 1H'), 3.10 (dd, *J* = 16.9, 4.8 Hz, 1H').

^{13}C NMR (126 MHz, $CDCl_3$) δ 198.9, 198.7, 162.4 (d, *J* = 52.4 Hz), 160.5 (d, *J* = 51.7 Hz), 142.5, 142.0, 140.6, 139.2, 138.4 (d, *J* = 3.2 Hz), 138.2 (d, *J* = 3.2 Hz), 137.4, 137.3, 132.94, 132.92, 129.8 (d, *J* = 7.8 Hz), 129.5 (d, *J* = 7.8 Hz), 128.8, 128.6, 128.5, 128.4, 128.2, 128.1, 128.0, 127.9, 126.6, 126.3, 116.6, 116.4, 115.5 (d, *J* = 21.1 Hz), 115.0 (d, *J* = 21.2 Hz), 56.6, 54.6, 46.6, 45.9, 43.6, 43.2.

^{19}F NMR (471 MHz, $CDCl_3$) δ -116.13, -116.75.

HRMS (ESI) calcd for $C_{24}H_{22}OF$ $[M+H]^+$: 345.1649, found: 345.1648.

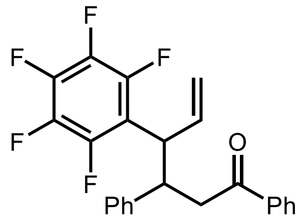

3ae

4-(Perfluorophenyl)-1,3-diphenylhex-5-en-1-one (3ae): (SGS-4-15) Prepared following **General Procedure A** using chalcone (**1a**, 62.4 mg, 0.3 mmol, 1.0 equiv) and 1-allyl-2,3,4,5,6-pentafluorobenzene (**2e**, 138 μL, 0.9 mmol, 3.0 equiv). The reaction mixture was heated at 80 °C for 16 h. The crude reaction mixture was purified by flash column chromatography (hexanes/ethyl acetate = 100:1) to afford the title compound as a white solid (84.9 mg, 68%). The diastereomeric ratio (dr 1.2:1) was determined by NMR analysis of the crude product.

^{1}H NMR (300 MHz, $CDCl_3$) δ 7.88 – 7.80 (m, 2H*), 7.74 – 7.68 (m, 2H'), 7.55 – 6.98 (m, 8H* + 8H'), 6.27 – 6.08 (m, 1H*), 6.01 – 5.84 (m, 1H'), 5.28 (dd, *J* = 38.5, 13.4 Hz, 2H*), 4.87 (dd, *J* = 21.4, 13.6 Hz, 1H'), 4.16 – 3.91 (m, 2H* + 2H'), 3.56 – 3.26 (m, 2H* + 1H'), 3.07 (dd, *J* = 16.6, 5.3 Hz, 1H').

^{13}C NMR (126 MHz, $CDCl_3$) δ 198.2, 197.7, 146.3 – 146.0 (m), 145.6 – 145.3 (m), 144.3 – 144.0 (m), 143.6 – 143.4 (m), 141.9, 138.9 – 138.2 (m), 137.3, 136.9, 136.5, 136.4 – 136.2 (m), 135.7, 133.3, 133.1, 128.70, 128.66, 128.64, 128.57, 128.4, 128.1, 127.9, 127.5, 127.2, 127.1, 119.6, 118.4, 116.5 – 115.9 (m), 47.4, 46.1, 44.4, 44.1, 43.4, 43.2 (carbons missing due to complex C-F coupling).

^{19}F NMR (471 MHz, $CDCl_3$) δ -140.62 (s), -141.66 (s), -156.07 (t, *J* = 20.9 Hz), -156.80 (t, *J* = 21.0 Hz), -161.63 (td, *J* = 22.0, 7.9 Hz), -162.52 (td, *J* = 22.1, 7.6 Hz).

HRMS (ESI) calcd for $C_{24}H_{18}OF_5$ $[M+H]^+$: 417.1272, found: 417.1269.

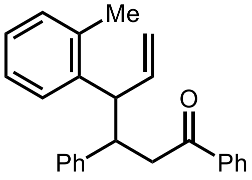

3af

4-(2-Methylphenyl)-1,3-diphenylhex-5-en-1-one (3af): (SGS-4-8) Prepared following **General Procedure A** using chalcone (**1a**, 62.4 mg, 0.3 mmol, 1.0 equiv) and 1-allyl-2-methylbenzene (**2f**, 132 μL, 0.9 mmol, 3.0 equiv). The reaction mixture was heated at 80 °C for 16 h. The crude reaction mixture was purified by flash column chromatography (hexanes/ethyl ether = 100:1) to afford the title compound as a white solid (89.4 mg, 87%). The diastereomeric ratio (dr 1.1:1) was determined by NMR analysis of the crude product.

1H NMR (500 MHz, CDCl3) δ 7.82 – 7.75 (m, 2H'), 7.66 – 7.59 (m, 2H*), 7.42 (t, J = 7.4 Hz, 1H'), 7.37 (t, J = 7.4 Hz, 1H*), 7.35 – 6.79 (m, 11H* + 11H'), 5.95 – 5.84 (m, 1H'), 5.70 (ddd, J = 17.2, 10.2, 8.0 Hz, 1H*), 5.00 (ddd, J = 13.4, 11.5, 1.3 Hz, 1H'), 4.70 (dd, J = 60.2, 13.6 Hz, 1H*), 3.91 – 3.70 (m, 2H* + 2H'), 3.51 (dd, J = 17.0, 4.8 Hz, 1H'), 3.34 – 3.16 (m, 1H* + 1H'), 3.00 (dd, J = 16.5, 4.1 Hz, 1H*), 2.36 (s, 3H'), 2.02 (s, 3H*).

^{13}C NMR (126 MHz, $CDCl_3$) δ 199.1, 199.0, 143.1, 142.6, 140.9, 140.8, 140.6, 139.5, 137.6, 137.3, 136.3, 135.5, 132.9, 132.9, 130.8, 130.3, 128.8, 128.6, 128.5, 128.3, 128.2, 128.1, 128.0, 127.3, 127.2, 126.6, 126.5, 126.4, 126.2, 126.1, 125.9, 116.1, 52.5, 50.5, 46.1, 45.1, 43.9, 43.2, 20.1, 19.7 (two carbons missing due to overlap).

HRMS (ESI) calcd for $C_{25}H_{25}O$ $[M+H]^+$: 341.1900, found: 341.1901.

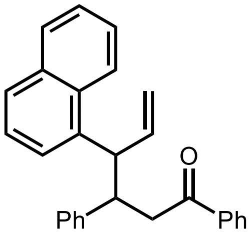

3ag

4-(Naphthalen-1-yl)-1,3-diphenylhex-5-en-1-one (3ag): (SGS-4-14) Prepared following **General Procedure A** using chalcone (**1a**, 62.4 mg, 0.3 mmol, 1.0 equiv) and 1-allylnaphthalene (**2g**, 151 µL, 0.9 mmol, 3.0 equiv). The reaction mixture was heated at 80 °C for 16 h. The crude reaction mixture was purified by flash column chromatography (hexanes/ethyl acetate = 100:1) to afford the title compound as a white solid (109.5 mg, 90%). The diastereomeric ratio (dr 1.1:1) was determined by NMR analysis of the crude product.

^{1}H NMR (500 MHz, $CDCl_3$) δ 8.42 (d, *J* = 8.6 Hz, 1H'), 8.16 (d, *J* = 8.5 Hz, 1H*), 7.92 – 6.89 (m, 16H* + 16H'), 6.25 – 6.13 (m, 1H'), 6.01 (ddd, *J* = 17.1, 10.2, 8.4 Hz, 1H*), 5.17 (ddd, *J* = 13.4, 11.4, 1.1 Hz, 2H'), 4.98 – 4.86 (m, 2H*), 4.58 (t, *J* = 8.7 Hz, 1H*), 4.52 (t, *J* = 9.5 Hz, 1H'), 4.17 (td, *J* = 8.9, 4.8 Hz, 1H'), 4.04 (td, *J* = 8.6, 5.4 Hz, 1H*), 3.63 (dd, *J* = 16.9, 4.8 Hz, 1H'), 3.45 (dd, *J* = 16.9, 8.4 Hz, 1H'), 3.35 (dd, *J* = 16.9, 8.4 Hz, 1H*), 3.23 (dd, *J* = 16.9, 5.4 Hz, 1H*).

^{13}C NMR (126 MHz, $CDCl_3$) δ 199.2, 199.1, 143.0, 142.4, 140.3, 138.9, 138.7, 138.6, 137.5, 137.3, 134.3, 134.1, 133.0, 132.9, 132.0, 131.6, 129.2, 129.1, 128.9, 128.6, 128.5, 128.2, 128.1, 128.1, 128.1, 127.3, 126.9, 126.7, 126.3, 126.2, 125.9, 125.7, 125.6, 125.4, 125.4, 125.3, 123.5, 123.1, 116.9, 116.8, 51.1, 49.4, 45.8, 45.1, 43.9, 43.3 (two carbons missing due to overlap).

HRMS (ESI) calcd for $C_{28}H_{25}O$ $[M+H]^+$: 377.1900, found: 377.1903.

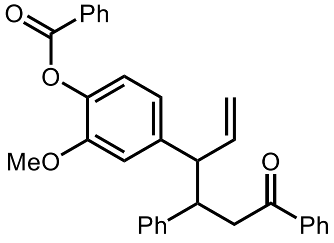

3ah

2-Methoxy-4-(6-oxo-4,6-diphenylhex-1-en-3-yl)phenyl benzoate (3ah): (SGS-4-30) Prepared following **General Procedure A** using chalcone (**1a**, 62.4 mg, 0.3 mmol, 1.0 equiv) and 2-methoxy-4-(6-oxo-4,6-diphenylhex-1-en-3-yl)phenyl benzoate (**2h**, 241 mg, 0.9 mmol, 3.0 equiv). The reaction mixture was heated at 80 °C for 16 h. The crude reaction mixture was purified by flash column chromatography (hexanes/ethyl acetate = 20:1) to afford the title compound as a white solid (119.2 mg, 83%). The diastereomeric ratio (dr 1.4:1) was determined by NMR analysis of the crude product.

^{1}H NMR (500 MHz, $CDCl_3$) δ 8.24 – 8.19 (m, 2H*), 8.19 – 8.13 (m, 2H'), 7.88 (d, *J* = 7.2 Hz, 2H'), 7.77 (d, *J* = 7.2 Hz, 2H*), 7.67 – 7.03 (m, 12H* +11H'), 6.94 (d, *J* = 8.1 Hz, 1H'), 6.87 (dd, *J* = 8.1, 1.7 Hz, 1H*), 6.80 (d, *J* = 1.7 Hz, 1H*), 6.67 (dd, *J* = 8.1, 1.8 Hz, 1H'), 6.55 (d, *J* = 1.7 Hz, 1H'), 6.14 – 6.04 (m, 1H'), 5.85 (ddd, *J* = 17.1, 10.2, 8.4 Hz, 1H*), 5.24 – 5.06 (m, 2H'), 4.90 (dd, *J* = 43.7, 13.6 Hz, 2H*), 3.87 – 3.74 (m, 4H* +1H'), 3.67 (t, *J* = 8.7 Hz, 1H*), 3.63 (s, 3H'), 3.61 – 3.49 (m, 2H'), 3.35 (ddd, *J* = 21.3, 17.0, 8.3 Hz, 1H* + 1H'), 3.22 (dd, *J* = 16.9, 4.9 Hz, 1H*).

^{13}C NMR (126 MHz, $CDCl_3$) δ 199.1, 199.0, 164.9, 164.8, 151.4, 150.7, 142.5, 142.2, 141.7, 141.3, 140.4, 139.2, 138.7, 138.3, 137.5, 137.4, 133.6, 133.5, 133.1, 133.0, 130.4, 130.4, 129.7, 129.6, 128.9, 128.7, 128.6, 128.6, 128.3, 128.2, 128.1, 128.1, 126.7, 126.5, 123.0, 122.5, 120.6, 120.5, 116.7, 116.6, 112.8, 112.6, 57.0, 56.1, 56.0, 55.4, 46.5, 46.0, 44.0, 43.3 (two carbons missing due to overlap).

HRMS (ESI) calcd for $C_{32}H_{29}O_4$ $[M+H]^+$: 477.2060, found: 477.2047.

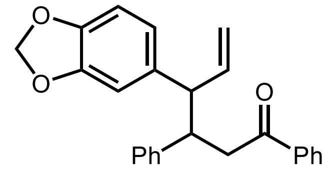

3ai

4-(Benzo[*d*][1,3]dioxol-5-yl)-1,3-diphenylhex-5-en-1-one (3ai): (SGS-4-10) Prepared following **General Procedure A** using chalcone (**1a**, 62.4 mg, 0.3 mmol, 1.0 equiv) and safrole (**2i**, 133 μL, 0.9 mmol, 3.0 equiv). The reaction mixture was heated at 80 °C for 16 h. The crude reaction mixture was purified by flash column chromatography (hexanes/ethyl acetate = 100:1) to afford the title compound as a white solid (86.9 mg, 78%). The diastereomeric ratio (dr 1.3:1) was determined by NMR analysis of the crude product.

^{1}H NMR (500 MHz, $CDCl_3$) δ 7.84 (d, *J* = 7.4 Hz, 2H'), 7.73 (d, *J* = 7.4 Hz, 2H*), 7.53 – 7.43 (m, 1H* +1H'), 7.39 (t, *J* = 7.7 Hz, 2H'), 7.35 (t, *J* = 7.7 Hz, 2H*), 7.26 – 7.00 (m, 12H), 6.74 (d, *J* = 7.6 Hz, 2H*), 6.69 (d, *J* = 8.7 Hz, 1H*), 6.56 (d, *J* = 7.6 Hz, 2H'), 6.45 (d, *J* = 8.1 Hz, 1H'), 6.06 – 5.97 (m, 1H'), 5.91 (s, 2H*), 5.86 – 5.71 (m, 1H* + 2H'), 5.09 (dd, *J* = 56.8, 13.5 Hz, 2H'), 4.81 (dd, *J* = 45.6, 13.6 Hz, 2H*), 3.76 (td, *J* = 9.0, 5.1 Hz, 1H'), 3.69 (td, *J* = 9.4, 4.4 Hz, 1H*), 3.56 – 3.45 (m, 1H* + 2H'), 3.36 – 3.22 (m, 1H* +1H'), 3.12 (dd, *J* = 16.8, 4.4 Hz, 1H*).

^{13}C NMR (126 MHz, $CDCl_3$) δ 199.0, 198.9, 148.0, 147.5, 146.4, 145.8, 142.8, 142.5, 140.9, 139.7, 137.5, 137.4, 136.7, 136.5, 133.0, 132.9, 128.7, 128.6, 128.5, 128.3, 128.11, 128.06, 128.0, 126.6, 126.3, 121.5, 121.3, 116.2, 116.1, 108.6, 108.5, 108.4, 108.1, 101.1, 100.8, 57.0, 55.4, 46.7, 45.8, 43.7, 43.5 (one carbon missing due to overlap).

HRMS (ESI) calcd for $C_{25}H_{23}O_3$ $[M+H]^+$: 371.1642, found: 371.1644.

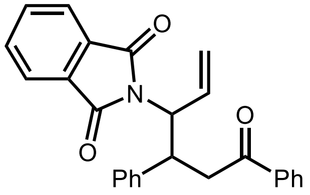

3aj

2-(6-Oxo-4,6-diphenylhex-1-en-3-yl)isoindoline-1,3-dione (3aj): (SGS-3-184) Prepared following **General Procedure A** using $LiNTf_2$ (68.9 mg, 0.24 mmol, 0.8 equiv), chalcone (**1a**, 62.4 mg, 0.3 mmol, 1.0 equiv) and 2-allylisoindoline-1,3-dione (**2j**, 187.2 mg, 0.9 mmol, 3.0 equiv). The reaction mixture was heated at 80 °C for 16 h. The crude reaction mixture was purified by flash column chromatography (hexanes/ethyl acetate = 100:1) to afford the title compound as a white solid (73.9 mg, 62%). The diastereomeric ratio (dr 1.5:1) was determined by NMR analysis of the crude product.

^{1}H NMR (500 MHz, $CDCl_3$) δ 7.88 – 7.15 (m, 14H* + 11H'), 7.08 (t, *J* = 7.5 Hz, 2H'), 6.98 (t, *J* = 7.3 Hz, 1H'), 6.50 – 6.41 (m, 1H'), 6.21 – 6.10 (m, 1H*), 5.41 (dd, *J* = 73.2, 13.6 Hz, 2H'), 5.06 – 4.91 (m, 3H* + 1H'), 4.45 – 4.31 (m, 1H* + 1H'), 3.46 – 3.24 (m, 2H* + 2H').

^{13}C NMR (126 MHz, $CDCl_3$) δ 197.80, 197.77, 168.2, 167.7, 141.2, 140.5, 137.3, 136.8, 134.6, 134.1, 134.0, 133.8, 133.1, 133.0, 131.9, 131.6, 128.8, 128.7, 128.5, 128.4, 128.2, 128.1, 128.1, 127.2, 127.0, 123.5, 123.1, 120.9, 119.2, 59.2, 58.8, 43.5, 43.3, 42.7, 42.3 (one carbon missing due to overlap).

HRMS (ESI) calcd for $C_{26}H_{22}O_3N$ $[M+H]^+$: 396.1594, found: 396.1596.

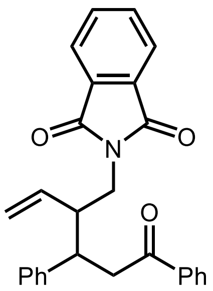

3ak

2-(5-Oxo-3,5-diphenyl-2-vinylpentyl)isoindoline-1,3-dione (3ak): (SGS-4-24) Prepared following **General Procedure A** using $LiNTf_2$ (68.9 mg, 0.24 mmol, 0.8 equiv), chalcone (**1a**, 62.4 mg, 0.3 mmol, 1.0 equiv) and 2-(but-3-en-1-yl)isoindoline-1,3-dione (**2k**, 181.1 mg, 0.9 mmol, 3.0 equiv). The reaction mixture was heated at 80 °C for 16 h. The crude reaction mixture was purified by flash column chromatography (hexanes/ethyl acetate = 100:1) to afford the title compound as a white solid (38.4 mg, 31%). The diastereomeric ratio (dr 1.1:1) was determined by NMR analysis of the crude product.

^{1}H NMR (500 MHz, $CDCl_3$) δ 7.90 – 7.63 (m, 6H* + 6H'), 7.51 – 7.20 (m, 7H* + 7H'), 7.17 (t, *J* = 7.1 Hz, 1H*), 7.12 (t, *J* = 7.3 Hz, 1H'), 5.71 – 5.59 (m, 1H'), 5.58 – 5.48 (m, 1H*), 5.04 – 4.83 (m, 2H* + 2H'), 3.73 (dd, *J* = 13.5, 4.9 Hz, 1H*), 3.69 – 3.51 (m, 1H* + 2H'), 3.48 – 3.22 (m, 3H* + 3H'), 3.06 – 2.98 (m, 1H'), 2.97 – 2.91 (m, 1H*).

^{13}C NMR (126 MHz, $CDCl_3$) δ 198.6, 198.3, 168.4, 168.4, 142.3, 140.8, 138.6, 137.4, 137.3, 136.7, 133.9, 133.9, 133.1, 133.0, 132.2, 132.1, 129.0, 128.8, 128.7, 128.6, 128.4, 128.2, 128.2, 128.1, 126.9, 123.3, 123.2, 119.5, 119.1, 45.0, 48.0, 44.6, 43.7, 42.6, 42.5, 41.3, 41.2 (one carbon missing due to overlap).

HRMS (ESI) calcd for $C_{27}H_{24}O_3N$ $[M+H]^+$: 410.1751, found: 410.1748.

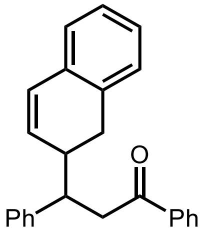

3al

3-(1,2-Dihydronaphthalen-2-yl)-1,3-diphenylpropan-1-one (3al): (SGS-4-18) Prepared following **General Procedure A** using chalcone (**1a**, 62.4 mg, 0.3 mmol, 1.0 equiv) and 1,2-dihydronaphthalene (**2l**, 117.5 μL, 0.9 mmol, 3.0 equiv). The reaction mixture was heated at 80 °C for 16 h. The crude reaction mixture was purified by flash column chromatography (hexanes/ethyl acetate = 100:1) to afford the title compound as a yellow oil (48.6 mg, 47%). The diastereomeric ratio (dr 1.2:1) was determined by NMR analysis of the crude product.

^{1}H NMR (300 MHz, $CDCl_3$) δ 8.11 – 6.87 (m, 14H* + 14H'), 6.56 (d, *J* = 9.7 Hz, 1H*), 6.43 (d, *J* = 9.6 Hz, 1H'), 6.15 (dd, *J* = 9.7, 4.3 Hz, 1H*), 5.79 (dd, *J* = 9.6, 2.7 Hz, 1H'), 3.67 – 3.27 (m, 3H* + 3H'), 2.98 – 2.61 (m, 2H* + 3H'), 2.48 (dd, *J* = 17.7, 9.0 Hz, 1H*).

^{13}C NMR (126 MHz, $CDCl_3$) δ 199.1, 198.9, 143.3, 142.9, 137.4, 137.3, 134.7, 134.5, 134.0, 133.8, 133.0, 132.9, 131.4, 130.6, 128.9, 128.7, 128.6, 128.5, 128.5, 128.4, 128.2, 128.2, 128.2, 128.1, 127.9, 127.4, 127.3, 126.7, 126.6, 126.6, 126.6, 126.0, 126.0, 44.6, 44.3, 42.5, 41.8, 39.8, 39.0, 32.2, 31.9 (one carbon missing due to overlap).

HRMS (ESI) calcd for $C_{25}H_{23}O$ $[M+H]^+$: 339.1743, found: 339.1752.

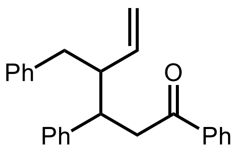

3am

4-Benzyl-1,3-diphenylhex-5-en-1-one (3am): (SGS-4-16) Prepared following **General Procedure A** using chalcone (**1a**, 62.4 mg, 0.3 mmol, 1.0 equiv) and 4-phenyl-1-butene (**2m**, 104.7 µL, 0.9 mmol, 3.0 equiv). The reaction mixture was heated at 80 °C for 16 h. The crude reaction mixture was purified by flash column chromatography (hexanes/ethyl ether = 100:1) to afford the title compound as a yellow oil (54.6 mg, 53%). The diastereomeric ratio (dr 1:1) was determined by NMR analysis of the crude product.

^{1}H NMR (500 MHz, $CDCl_3$) δ 7.93 (d, *J* = 7.9 Hz, 2H), 7.83 (d, *J* = 7.9 Hz, 2H), 7.57 – 6.95 (m, 13H+ 13H), 5.70 – 5.50 (m, 1H +1H), 5.03 – 4.80 (m, 2H + 2H), 3.74 – 3.60 (m, 1H), 3.55 – 3.24 (m, 3H + 2H), 2.83 – 2.74 (m, 2H), 2.67 – 2.59 (m, 2H), 2.43 (dd, *J* = 13.1, 9.1 Hz, 1H), 2.38 – 2.28 (m, 1H).

^{13}C NMR (126 MHz, $CDCl_3$) δ 199.1, 198.9, 143.6, 141.3, 140.6, 140.3, 138.5, 137.4, 137.4, 133.0, 132.8, 129.2, 128.6, 128.5, 128.5, 128.4, 128.1, 128.0, 128.0, 126.6, 126.5, 125.8, 125.7, 117.5, 117.5, 52.7, 49.8, 45.1, 44.0, 44.0, 42.5, 39.7, 39.3 (five carbons missing due to overlap).

HRMS (ESI) calcd for $C_{25}H_{25}O$ [M+H]$^+$: 341.1900, found: 341.1909.

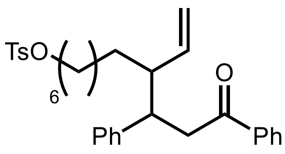

3an

11-Oxo-9,11-diphenyl-8-vinylundecyl 4-methylbenzenesulfonate (3an): (SGS-4-42) Prepared following **General Procedure A** using chalcone (**1a**, 62.4 mg, 0.3 mmol, 1.0 equiv) and dec-9-en-1-yl 4-methylbenzenesulfonate (**2n**, 279.4 mg, 0.9 mmol, 3.0 equiv). The reaction mixture was heated at 80 °C for 16 h. The crude reaction mixture was purified by flash column chromatography (hexanes/ethyl ether = 100:1) to afford the title compound as a yellow oil (74.9 mg, 50%). The diastereomeric ratio (dr 1.5:1) was determined by NMR analysis of the crude product.

^{1}H NMR (500 MHz, $CDCl_3$) δ 7.93 (d, *J* = 7.3 Hz, 2H*), 7.82 (d, *J* = 7.3 Hz, 2H'), 7.78 (dd, *J* = 8.2, 4.4 Hz, 2H* + 2H'), 7.54 (t, *J* = 7.4 Hz, 1H*), 7.49 (t, *J* = 7.4 Hz, 1H'), 7.44 (t, *J* = 7.7 Hz, 2H*), 7.39 (t, *J* = 7.7 Hz, 2H'), 7.36 – 7.11 (m, 7H* + 7H'), 5.62 – 5.52 (m, 1H'), 5.48 – 5.37 (m, 1H*), 5.12 – 4.89 (m, 2H* + 2H'), 4.01 – 3.96 (m, 2H* + 2H'), 3.54 – 3.18 (m, 3H* + 3H'), 2.44 (s, 3H* + 3H'), 2.34 – 2.19 (m, 1H* + 1H'), 1.67 – 1.50 (m, 3H* + 3H'), 1.47 – 1.35 (m, 1H* + 1H'), 1.31 – 0.97 (m, 8H* + 8H').

^{13}C NMR (126 MHz, $CDCl_3$) δ 199.4, 199.2, 144.7, 143.9, 141.8, 141.7, 139.8, 137.6, 137.5, 133.4, 133.0, 132.8, 129.9, 129.1, 128.7, 128.5, 128.4, 128.4, 128.1, 128.0, 128.0, 126.4, 126.4, 116.9, 116.9, 70.8, 50.5, 48.6, 45.3, 44.5, 44.2, 42.5, 33.0, 32.3, 29.4, 29.2, 29.0, 28.9, 28.9, 28.8, 27.5, 27.1, 25.4, 25.3, 21.7 (five carbons missing due to overlap).

HRMS (ESI) calcd for $C_{32}H_{39}O_4S$ [M+H]$^+$: 519.2564, found: 519.2551.

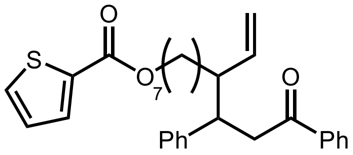

3ao

11-Oxo-9,11-diphenyl-8-vinylundecyl thiophene-2-carboxylate (3ao): (SGS-4-40) Prepared following **General Procedure A** using chalcone (**1a**, 62.4 mg, 0.3 mmol, 1.0 equiv) and dec-9-en-1-yl thiophene-2-carboxylate (**2o**, 239.7 mg, 0.9 mmol, 3.0 equiv). The reaction mixture was heated at 80 °C for 16 h. The crude

reaction mixture was purified by flash column chromatography (hexanes/ethyl acetate = 100:1) to afford the title compound as a yellow oil (53.9 mg, 38%). The diastereomeric ratio (dr 1.6:1) was determined by NMR analysis of the crude product.

^{1}H NMR (500 MHz, $CDCl_3$) δ 7.96 – 7.90 (m, 2H*), 7.84 – 7.80 (m, 2H'), 7.80 – 7.76 (m, 1H* + 1H'), 7.57 – 7.13 (m, 10H* + 10H'), 7.11 – 7.07 (m, 1H* + 1H'), 5.63 – 5.53 (m, 1H'), 5.48 – 5.38 (m, 1H'), 5.16 – 4.82 (m, 2H* + 2H'), 4.34 – 4.16 (m, 2H* + 2H'), 3.55 – 3.18 (m, 3H* + 3H'), 2.35 – 2.20 (m, 1H* + 1H'), 1.74 – 1.64 (m, 2H * + 2H'), 1.51 – 0.96 (m, 10H* + 10H').

^{13}C NMR (126 MHz, $CDCl_3$) δ 199.5, 199.2, 162.5, 143.9, 141.9, 141.7, 139.9, 137.6, 137.6, 134.3, 133.3, 133.0, 132.8, 132.3, 129.2, 128.7, 128.5, 128.4, 128.4, 128.13, 128.05, 128.0, 127.8, 126.43, 126.38, 116.9, 116.8, 65.4, 65.4, 50.6, 48.7, 45.4, 44.6, 44.2, 42.5, 33.1, 32.3, 29.6, 29.3, 29.2, 28.8, 28.8, 27.6, 27.2, 26.0, 26.0 (six carbons missing due to overlap).

HRMS (ESI) calcd for $C_{30}H_{35}O_3S$ $[M+H]^+$: 475.2301, found: 475.2304.

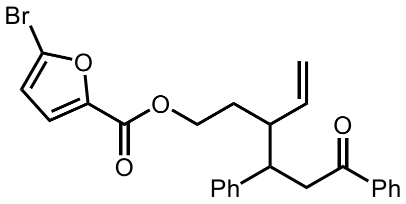

3ap

6-Oxo-4,6-diphenyl-3-vinylhexyl 5-bromofuran-2-carboxylate (3ap): (SGS-4-41) Prepared following **General Procedure A** using chalcone (**1a**, 62.4 mg, 0.3 mmol, 1.0 equiv) and pent-4-en-1-yl 5-bromofuran-2-carboxylate (**2p**, 233.2 mg, 0.9 mmol, 3.0 equiv). The reaction mixture was heated at 80 °C for 16 h. The crude reaction mixture was purified by flash column chromatography (hexanes/ethyl ether = 100:1) to afford the title compound as a yellow oil (78.2 mg, 56%). The diastereomeric ratio (dr 1.2:1) was determined by NMR analysis of the crude product.

^{1}H NMR (500 MHz, $CDCl_3$) δ 7.87 – 7.82 (m, 2H*), 7.78 – 7.72 (m, 2H'), 7.47 (t, = 7.4 Hz, 1H*), 7.43 (t, *J* = 7.4 Hz, 1H'), 7.40 – 7.29 (m, 2H* + 2H'), 7.23 – 7.06 (m, 6H* + 6H'), 7.02 (d, *J* = 3.5 Hz, 1H*), 6.97 (d, *J* = 3.5 Hz, 1H'), 6.37 – 6.36 (m, 1H* + 1H'), 5.60 – 5.51 (m, 1H'), 5.46 – 5.38 (m, 1H*), 5.19 – 4.90 (m, 2H* + 2H'), 4.22 – 4.04 (m, 2H* +2H'), 3.48 – 3.17 (m, 3H* + 3H'), 2.50 – 2.35 (m, 1H* + 1H'), 1.93 – 1.86 (m, 1H*), 1.65 – 1.59 (m, 1H'), 1.48 – 1.34 (m, 1H* + 1H').

^{13}C NMR (126 MHz, $CDCl_3$) δ 199.0, 198.8, 157.7, 157.6, 146.6, 143.2, 141.1, 140.3, 138.0, 137.5, 137.4, 133.2, 133.0, 129.1, 128.7, 128.7, 128.6, 128.4, 128.2, 128.12, 128.05, 127.54, 127.48, 126.78, 126.75, 120.1, 120.0, 118.3, 118.2, 114.0, 114.0, 63.8, 63.7, 47.5, 45.3, 45.2, 44.6, 44.3, 42.3, 32.0, 31.3 (one carbon missing due to overlap).

HRMS (ESI) calcd for $C_{25}H_{24}O_4Br$ $[M+H]^+$: 467.0853, found: 467.0866.

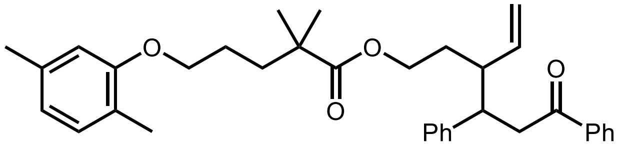

3aq

6-Oxo-4,6-diphenyl-3-vinylhexyl 5-(2,5-dimethylphenoxy)-2,2-dimethylpentanoate (3aq): (SGS-4-39) Prepared following **General Procedure A** using chalcone (**1a**, 62.4 mg, 0.3 mmol, 1.0 equiv) and pent-4-en-1-yl 5-(2,5-dimethylphenoxy)-2,2-dimethylpentanoate (**2q**, 273.9 mg, 0.9 mmol, 3.0 equiv). The reaction mixture was heated at 80 °C for 16 h. The crude reaction mixture was purified by flash column chromatography (hexanes/ethyl acetate = 100:1) to afford the title compound as a clear oil (48.2 mg, 31%). The diastereomeric ratio (dr 1.2:1) was determined by NMR analysis of the crude product.

^{1}H NMR (500 MHz, $CDCl_3$) δ 7.92 (dd, *J* = 8.3, 1.1 Hz, 2H*), 7.84 – 7.79 (m, 2H'), 7.56 – 7.47 (m, 1H* + 1H'), 7.46 – 7.36 (m, 2H* + 2H'), 7.31 – 7.12 (m, 5H* + 5H'), 7.00 (t, *J* = 8.0 Hz, 1H* + 1H'), 6.69 – 6.56 (m, 2H* + 2H'), 5.64 – 5.55 (m, 1H'), 5.50 – 5.42 (m, 1H*), 5.18 – 4.96 (m, 2H* + 2H'), 4.11 – 3.86 (m, 4H* + 4H'), 3.55 – 3.21 (m, 3H* + 3H'), 2.56 – 2.39 (m, 1H* + 1H'), 2.32 (s, 3H'), 2.30 (s, 3H*), 2.18 (s, 3H'), 2.17 (s, 3H*), 1.92 – 1.55 (m, 5H* + 5H'), 1.44 – 1.30 (m, 1H* + 1H'), 1.20 (s, 6H*), 1.18 (d, *J* = 1.4 Hz, 6H').
^{13}C NMR (126 MHz, $CDCl_3$) δ 199.0, 198.8, 177.9, 177.7, 157.1, 143.3, 141.2, 140.4, 138.2, 137.5, 137.4, 136.6, 136.6, 133.1, 132.9, 130.4, 130.4, 129.1, 128.7, 128.6, 128.6, 128.4, 128.2, 128.1, 128.0, 126.7, 126.7, 123.7, 120.9, 120.8, 118.2, 112.1, 68.1, 68.1, 63.9, 62.8, 62.6, 47.6, 45.3, 45.2, 44.6, 44.2, 42.3, 42.2, 42.1, 37.2, 32.1, 31.4, 25.3, 25.3, 25.3, 21.5, 15.9, 15.9 (eight carbons missing due to overlap).
HRMS (ESI) calcd for $C_{35}H_{43}O_4$ $[M+H]^+$: 527.3156, found: 527.3182.

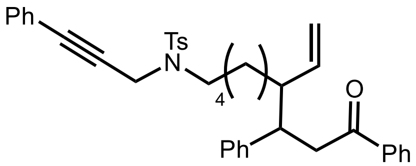

3ar

4-Methyl-*N*-(9-oxo-7,9-diphenyl-6-vinylnonyl)-*N*-(3-phenylprop-2-yn-1-yl)benzenesulfonamide (3ar): (SGS-4-36) Prepared following **General Procedure A** using chalcone (**1a**, 62.4 mg, 0.3 mmol, 1.0 equiv) and 4-methyl-N-(oct-7-en-1-yl)-N-(3-phenylprop-2-yn-1-yl)benzenesulfonamide (**2r**, 395.6 mg, 0.9 mmol, 3.0 equiv). The reaction mixture was heated at 80 °C for 16 h. The crude reaction mixture was purified by flash column chromatography (hexanes/ethyl acetate = 100:1) to afford the title compound as a yellow oil (66.2 mg, 37%). The diastereomeric ratio (dr 1.6:1) was determined by NMR analysis of the crude product.
^{1}H NMR (500 MHz, $CDCl_3$) δ 7.95 – 7.91 (m, 2H*), 7.83 – 7.80 (m, 2H'), 7.77 – 7.72 (m, 2H* + 2H'), 7.54 (t, *J* = 7.4 Hz, 1H*), 7.49 (t, *J* = 7.4 Hz, 1H'), 7.44 (t, *J* = 7.7 Hz, 2H*), 7.39 (t, *J* = 7.7 Hz, 2H'), 7.29 – 7.02 (m, 11H* + 11H'), 5.61 – 5.52 (m, 1H'), 5.46 – 5.37 (m, 1H*), 5.09 – 4.91 (m, 2H* + 2H'), 4.31 (s, 2H*), 4.27 (s, 2H'), 3.52 – 3.12 (m, 5H* + 5H'), 2.37 – 2.19 (m, 4H* + 4H'), 1.61 – 1.43 (m, 3H* + 3H'), 1.37 – 1.04 (m, 5H* + 5H').
^{13}C NMR (126 MHz, $CDCl_3$) δ 199.5, 199.3, 143.9, 143.5, 142.0, 141.7, 139.8, 137.8, 137.7, 136.3, 133.2, 132.9, 131.8, 131.7, 129.9, 129.7, 129.7, 129.3, 128.8, 128.7, 128.6, 128.6, 128.5, 128.4, 128.3, 128.19, 128.17, 128.1, 128.0, 127.4, 126.6, 126.6, 122.5, 117.2, 117.1, 85.8, 85.72, 82.2, 50.6, 48.7, 46.7, 46.6, 45.5, 44.7, 44.4, 42.6, 37.21, 37.17, 33.1, 32.3, 27.8, 27.6, 27.4, 27.1, 26.8, 26.6, 26.6, 21.7 (four carbons missing due to overlap).
HRMS (ESI) calcd for $C_{39}H_{42}O_3NS$ $[M+H]^+$: 604.2880, found: 604.2880.

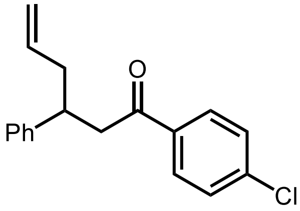

1-(4-chlorophenyl)-3-phenylpent-4-en-1-one (S1): (SGS-4-76-1) Prepared following an adapted literature procedure.[3] To an oven-dried pressure tube (102 mm × 13 mm, Ace glass, part# 8648-61) charged with a magnetic stir bar, $[Cp^*Fe(CO)_2(thf)]^+[BF_4]^-$ (26.4 mg, 20 mol %), 4'-chlorochalcone (**1o**, 72.8 mg, 0.3 mmol, 1.0 equiv), $LiNTf_2$ (0.11 mmol, 30.1 mg, 0.35 equiv) dry toluene (0.2 mL, 1.5 M), collidine (0.6 mmol, 80 μL, 2.0 equiv) and TIPSOTf (0.45 mmol, 139 μL, 1.5 equiv) were added in succession in an argon-filled glovebox. The tube was capped with a PTFE bushing with plunger valve (#7 Ace-Thred) and was cooled in liquid nitrogen. After the entire reaction mixture was frozen, the tube was placed under vacuum. Propylene (27 mL, 5.5 equiv, 8 atm at 80 °C, initial pressure) was condensed into the vacuum tube through the plunger valve, then the tube was resealed and removed from the liquid nitrogen. After warming up to room temperature, the tube was heated at

80 °C for 16 h with vigorous stirring. The crude mixture was concentrated in vacuo and purified by flash column chromatography on silica gel (hexanes/ethyl acetate = 100:1) to afford the title compound as a yellow oil (26.5 mg, 31%).
^{1}H NMR (500 MHz, $CDCl_3$) δ 7.85 – 7.78 (m, 2H), 7.42 – 7.37 (m, 2H), 7.30 – 7.26 (m, 2H), 7.24 – 7.20 (m, 2H), 7.20 – 7.16 (m, 1H), 5.68 (ddt, *J* = 17.2, 10.1, 7.0 Hz, 1H), 5.04 – 4.94 (m, 2H), 3.45 (p, *J* = 7.1 Hz, 1H), 3.27 – 3.23 (m, 2H), 2.45 (t, *J* = 7.1 Hz, 2H).
^{13}C NMR (126 MHz, $CDCl_3$) δ 197.9, 144.3, 139.5, 136.3, 135.7, 129.6, 129.0, 128.6, 127.7, 126.6, 117.0, 44.7, 41.0, 40.8.
HRMS (ESI) calcd for $C_{18}H_{18}OCl$ $[M+H]^+$: 285.1041, found: 285.1039.

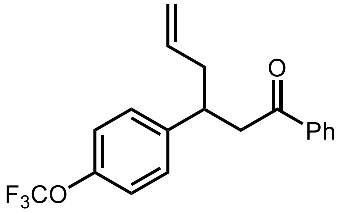

1-phenyl-3-(4-(trifluoromethoxy)phenyl)pent-4-en-1-one (**S2**): (SGS-4-76-2) Prepared in analogy to SGS-4-76-2 using 3-phenyl-1-(4-(trifluoromethoxy)phenyl)prop-2-en-1-one (**1d**, 87.7 mg, 0.3 mmol, 1.0 equiv). The reaction mixture was heated at 80 °C for 16 h. The crude reaction mixture was purified by flash column chromatography (hexanes/ethyl acetate = 100:1) to afford the title compound as a yellow oil (22.1 mg, 22%).
^{1}H NMR (500 MHz, $CDCl_3$) δ 7.92 – 7.86 (m, 2H), 7.58 – 7.51 (m, 1H), 7.47 – 7.41 (m, 2H), 7.28 – 7.22 (m, 3H), 7.12 (d, *J* = 8.0 Hz, 2H), 5.67 (ddt, *J* = 17.1, 10.2, 7.0 Hz, 1H), 5.07 – 4.95 (m, 2H), 3.54 – 3.46 (m, 1H), 3.35 – 3.22 (m, 2H), 2.51 – 2.38 (m, 2H).
^{13}C NMR (126 MHz, $CDCl_3$) δ 198.7, 147.8, 143.2, 137.2, 135.9, 133.2, 129.0, 128.7, 128.1, 121.1, 120.6 (q, *J* = 256.6 Hz), 117.4, 44.6, 40.8, 40.2.
^{19}F NMR (471 MHz, $CDCl_3$) δ -57.87.
HRMS (ESI) calcd for $C_{19}H_{18}O_2F_3$ $[M+H]^+$: 335.1253, found: 335.1249.

2.3 Synthesis of Michael acceptor substrates

The Michael Acceptors **1a**, **1b**, **1e**, **1f**, **1i**, and **1o** are commercial, and were used directly without purification.
The Michael Acceptors (**1c**, **1k**, **1l**, **1j**, **1p**, **1s**),[1] **1d**,[4] **1g**,[5] **1n**,[6] **1q**,[7] **1t**,[8] **1u**,[9] **1v**,[10] **1w**[11] were synthesized according to known literature procedures and have been previously characterized.

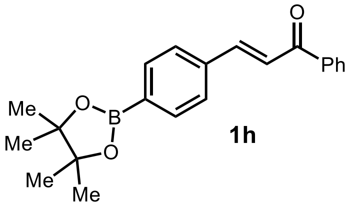

1h

1-Phenyl-3-(4-(4,4,5,5-tetramethyl-1,3,2-dioxaborolan-2-yl)phenyl)prop-2-en-1-one (**1h**): (SGS-3-187-8) Prepared following **General Procedure B** using 4-formylbenzeneboronic acid pinacol ester (557.0 mg, 2.4 mmol) and acetophenone (233.0 μL, 2.0 mmol) to afford the title compound as a white solid (318.8 mg, 48%).
^{1}H NMR (300 MHz, $CDCl_3$) δ 8.10 – 7.40 (m, 12H), 1.36 (s, 12H).
^{13}C NMR (126 MHz, $CDCl_3$) δ 190.7, 144.8, 138.4, 137.6, 135.6, 133.0, 128.8, 128.7, 128.3, 127.8, 123.1, 84.2, 25.0.
HRMS (ESI) calcd for $C_{21}H_{24}O_3B$ $[M+H]^+$: 335.1813, found: 335.1825.

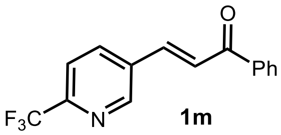

1m

1-Phenyl-3-(6-(trifluoromethyl)pyridin-3-yl)prop-2-en-1-one (1m): (SGS-3-187-12) Prepared following **General Procedure B** using 6-(trifluoromethyl)nicotinaldehyde (420.3 mg, 2.4 mmol) and acetophenone (233.0 μL, 2.0 mmol) to afford the title compound as an off white solid (277.25 mg, 51%).

^{1}H NMR (500 MHz, $CDCl_3$) δ 8.92 (s, 1H), 8.09 (d, *J* = 7.8 Hz, 1H), 8.01 (d, *J* = 7.4 Hz, 2H), 7.82 – 7.64 (m, 3H), 7.60 (t, *J* = 7.4 Hz, 1H), 7.51 (t, *J* = 7.6 Hz, 2H).

^{13}C NMR (126 MHz, $CDCl_3$) δ 189.4, 149.8, 148.9 (q, *J* = 35.1 Hz), 139.0, 137.5, 136.0, 133.6, 133.5, 128.9, 128.7, 126.1, 121.4 (q, *J* = 274.2 Hz), 120.7 (q, *J* = 2.64 Hz).

^{19}F NMR (471 MHz, $CDCl_3$) δ -67.67, -67.68, -67.69.

HRMS (ESI) calcd for $C_{15}H_{11}ONF_3$ [M+H]$^+$: 278.0787, found: 278.0789.

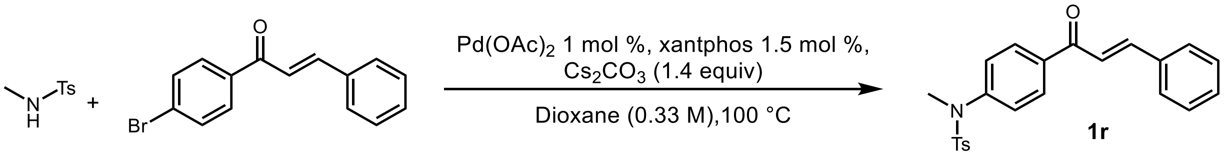

***N*-(4-Cinnamoylphenyl)-*N*,4-dimethylbenzenesulfonamide (1r)**: (**SGS-3-191**)

Step 1. 4'-bromochalcone was synthesized according to a known literature procedure and has been previously characterized.[1]

Step 2. This step was based on a reported protocol.[12]

A solution of *N*,4-dimethylbenzenesulfonamide (3.71 g, 20 mmol, 1.0 equiv), 4'-bromochalcone (344 mg, 1.2 mmol, 1.2 equiv), palladium(II) acetate (22.5 mg, 1 mol %), Xantphos (86.8 mg, 1.5 mol %), and Cs_2CO_3 (9.12 g, 28 mmol, 1.4 equiv) in dioxane (60 mL) under an N_2 atmosphere was stirred for 4 h at 100 °C. The solution was then cooled to room temperature, diluted with CH_2Cl_2(60 mL), filtered through a plug of Celite, and concentrated under reduced pressure. The crude residue was purified by flash column chromatography (hexanes/EtOAc = 4:1 to 1:1) to give pure **1r** (222.1 mg, 0.567 mmol, 47%) as an off white solid.

^{1}H NMR (500 MHz, $CDCl_3$) δ 7.81 (d, *J* = 8.6 Hz, 2H), 7.66 (d, *J* = 15.7 Hz, 1H), 7.50 – 7.48 (m, 2H), 7.36 (d, *J* = 15.7 Hz, 1H), 7.29 – 7.25 (m, 5H), 7.17 – 7.05 (m, 4H), 3.05 (s, 3H), 2.25 (s, 3H).

^{13}C NMR (126 MHz, $CDCl_3$) δ 189.4, 145.7, 145.2, 144.1, 136.4, 134.9, 133.4, 130.8, 129.7, 129.3, 129.1, 128.6, 127.9, 125.8, 121.8, 37.7, 21.7.

HRMS (ESI) calcd for $C_{23}H_{22}O_3NS$ [M+H]$^+$: 392.1315, found: 392.1334.

2.4 Synthesis of alkene substrates

The alkenes **2a**-**2g**, **2i**, **2l**, and **2m** are commercial, and were used directly without purification.

The alkenes **2h**,[13] **2j**,[14] **2k**,[14] **2n**,[15] **2o**,[16] **2p**,[17] **2q**,[18] **2r**[14] were synthesized according to known literature procedures and have been previously characterized.

3. Large scale synthesis and synthetic applications of products

Large scale synthesis of 1-(4-chlorophenyl)-4-(4-methoxyphenyl)-3-phenylhex-5-en-1-one (3ob)

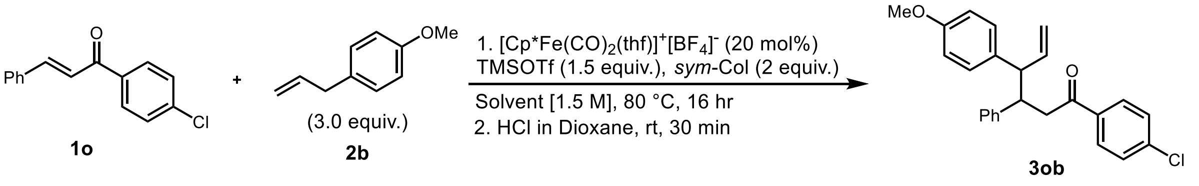

5 mmol scale reaction: A 25 mL round bottom flask equipped with a magnetic stir bar and condenser was flame dried under vacuum and transferred into an argon-filled glovebox. In the glovebox were added 4'-chlorochalcone (**1o**, 1.21 g, 5 mmol, 1.0 equiv), dry toluene (3.3 mL, 1.5 M), and 4-allylanisole (**2b**, 2.3 mL, 15 mmol, 3.0 equiv) and solution was briefly stirred. Then collidine (10 mmol, 1.33 mL, 2.0 equiv), TIPSOTf (7.5 mmol, 2.32 mL, 1.5 equiv), $LiNTf_2$ (1.75 mmol, 501.8 mg, 0.35 equiv), and $[Cp^*Fe(CO)_2(thf)]^+[BF_4]^-$ (15 mol %, 308.4 mg), were sequentially added with brief stirring after each addition. The flask was then removed from the glovebox and placed in an oil bath at 80 °C with water cooled condenser for 16 h at 400 rpm. After completion of the reaction, the reaction mixture was cooled to room temperature and filtered through a silica plug using ethyl acetate. A 4 N solution of HCl in dioxane (10 mmol, 2.5 mL, 2.0 equiv) was added at room temperature and the mixture was stirred for 30 min at room temperature. The crude mixture was concentrated *in vacuo*. The diastereomeric ratio (dr) was then determined by ^{1}H NMR analysis of a portion of the filtrate. After concentration *in vacuo*, the crude mixture was purified by flash column chromatography (hexanes/ethyl acetate = 100:1) to afford the title compound as an off white solid (1.78 g, 91%). The diastereomeric ratio (dr 1.6:1) was determined by NMR analysis of the crude product.

^{1}H NMR (500 MHz, $CDCl_3$) δ 7.76 (d, *J* = 8.4 Hz, 2H'), 7.62 (d, *J* = 8.5 Hz, 2H*), 7.38 – 7.34 (m, 1H'), 7.32 – 7.28 (m, 1H*), 7.26 – 6.97 (m, 7H), 6.91 (d, *J* = 7.9 Hz, 1H'), 6.84 (d, *J* = 8.2 Hz, 1H*), 6.66 (d, *J* = 8.4 Hz, 1H'), 6.07 – 5.99 (m, 1H'), 5.83 – 5.76 (m, 1H*), 5.08 (dd, *J* = 55.8, 13.5 Hz, 2H'), 4.88 – 4.71 (m, 2H*), 3.78 – 3.64 (m, 4H* + 4H'), 3.59 – 3.41 (m, 2H* + 1H'), 3.32 – 3.16 (m, 1H* + 1H'), 3.06 (dd, *J* = 16.6, 4.4 Hz, 1H*).

^{13}C NMR (126 MHz, $CDCl_3$) δ 198.0, 197.9, 158.5, 158.0, 142.6, 142.4, 141.0, 139.9, 139.4, 139.3, 135.8, 135.7, 134.6, 134.4, 129.5, 129.5, 129.3, 129.1, 128.9, 128.8, 128.7, 128.5, 128.3, 128.1, 126.6, 126.3, 116.0, 116.0, 114.2, 113.7, 56.4, 55.3, 55.2, 54.9, 46.9, 46.0, 43.6 (seven carbons missing due to overlap).

HRMS (ESI) calcd for $C_{25}H_{23}O_2Cl$ $[M+H]^+$: 391.1459, found: 391.1458.

Synthesis of 1-chloro-4-(3-(4-methoxyphenyl)-4-phenylcyclopent-1-en-1-yl)benzene (4)

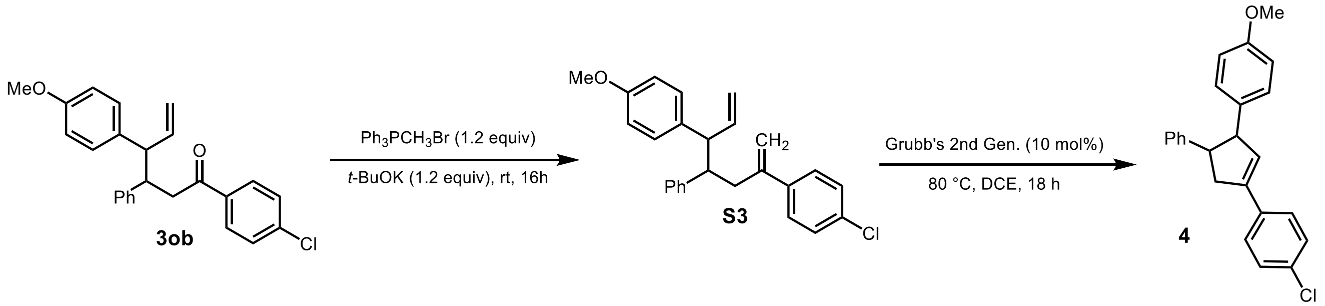

A flame dried round bottom flask with stir bar was transferred into the glove box. Methyltriphenylphosphonium bromide (267.9 mg, 0.75 mmol, 1.5 equiv) and potassium *tert*-butoxide (84.2 mg, 0.75 mmol, 1.5 equiv) were transferred into the flask and subsequently dissolved in anhydrous THF (2.5 mL) at room temperature. The resulting yellow solution was stirred at room temperature for 1 h. Ketone **3ob** (195.4 mg, 0.5 mmol, 1.0 equiv) was added to the reaction mixture. The flask was sealed, removed from the glove box, and stirred at rt for 16 h. The reaction is quenched by the addition of water and ethyl acetate. The crude reaction mixture is extracted with ethyl acetate. The combined organic layers were washed with water and brine, dried over $MgSO_4$, and evaporated under reduced pressure. The crude product was purified by flash chromatography (hexanes/ethyl ether = 100:3) to give the pure product **S3** as a yellow oil (167.8 mg, 86% yield).

^{1}H NMR (400 MHz, $CDCl_3$) δ 7.28 – 6.76 (m, 13H* + 11H'), 6.65 – 6.59 (m, 2H'), 6.11 – 5.99 (m, 1H'), 5.68 (ddd, *J* = 17.1, 10.2, 8.1 Hz, 1H*), 5.16 – 5.10 (m, 2H'), 4.98 (d, *J* = 0.7 Hz, 1H'), 4.92 (d, *J* = 1.2 Hz, 1H*), 4.82 – 4.73 (m, 1H* + 1H'), 4.72 – 4.63 (m, 2H*), 3.82 (s, 3H*), 3.66 (s, 3H'), 3.53 – 3.44 (m, 1H* + 1H'), 3.24 (dd, *J* = 14.3, 3.2 Hz, 1H'), 2.94 – 2.77 (m, 2H* + 1H'), 2.58 (dd, *J* = 14.3, 11.0 Hz, 1H'), 2.51 – 2.41 (m, 1H*).

^{13}C NMR (101 MHz, $CDCl_3$) δ 158.4, 157.8, 145.9, 145.7, 142.5, 142.1, 141.1, 140.4, 139.9, 139.5, 135.3, 135.0, 133.1, 129.3, 129.0, 128.8, 128.8, 128.5, 128.4, 128.0, 127.9, 127.9, 127.7, 126.3, 126.0, 115.9, 115.5, 115.3, 115.2, 114.1, 113.6, 56.1, 55.4, 55.2, 55.1, 49.9, 49.4, 40.7, 40.2 (one carbon missing due to overlap).

HRMS (ESI) calcd for $C_{26}H_{26}OCl$ $[M+H]^+$: 389.1667, found: 389.1676.

The following reaction was based on a literature procedure.[19] A reaction tube equipped with a magnetic stir bar was capped with a Teflon/silicone septum screw cap and flame dried under vacuum. **S3** (50.3 mg, 0.46 mmol, 1.0 equiv), Grubb's second generation catalyst (39.1 mg, 0.046 mmol, 10 mol %), and anhydrous DCE (5.0 mL) were added into the reaction tube in the glovebox. The reaction tube was sealed, removed from the glove box, and was stirred for 18 h at 80 °C. The reaction mixture was cooled to room temperature and filtered through a plug of silica gel and washed with CH_2Cl_2 (ca. 30 mL). The crude product was purified by flash chromatography (hexanes/ethyl ether = 100:1) to give the pure product **4** as a yellow oil (57.5 mg, 90% yield).
^{1}H NMR (300 MHz, $CDCl_3$) δ 7.56 – 6.86 (m, 9H* + 11H'), 6.85 – 6.78 (m, 2H'), 6.75 – 6.70 (m, 2H*), 6.63 – 6.56 (m, 2H*), 6.35 – 6.29 (m, 1H*), 6.22 (d, *J* = 1.5 Hz, 1H'), 4.33 – 4.25 (m, 1H*), 4.12 – 4.05 (m, 1H'), 4.00 (q, *J* = 8.4 Hz, 1H*), 3.79 (s, 3H'), 3.69 (s, 3H*), 3.41 (dd, *J* = 15.7, 7.4 Hz, 1H'), 3.36 – 3.23 (m, 1H'), 3.18 – 2.90 (m, 2H* + 1H').
^{13}C NMR (101 MHz, $CDCl_3$) δ 158.4, 158.2, 145.3, 142.4, 141.6, 140.9, 136.9, 134.8, 134.7, 133.30, 133.25, 132.5, 129.8, 129.3, 129.3, 128.8, 128.7, 128.6, 128.6, 128.5, 128.1, 127.9, 127.8, 127.5, 127.2, 126.5, 126.0, 114.0, 113.3, 60.2, 56.2, 55.4, 55.3, 54.8, 50.2, 42.0, 38.1 (one carbon missing due to overlap).
HRMS (ESI) calcd for $C_{24}H_{22}OCl$ $[M+H]^+$: 361.1354, found: 361.1350.

Synthesis of 4-(4-methoxyphenyl)-3-phenyl-1-(4-(piperidin-1-yl)phenyl)hex-5-en-1-one (5)

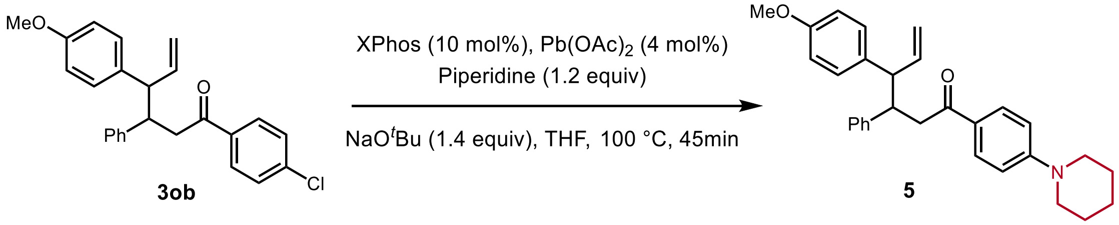

A reaction tube equipped with a magnetic stir bar was capped with a Teflon/silicone septum screw cap and flame dried under vacuum. Ketone **3ob** (117.3 mg, 0.3 mmol, 1.0 equiv), $Pd(OAc)_2$ (2.7 mg, 0.012 mmol, 4 mol %), XPhos (14.3 mg, 0.03 mmol, 10 mol %), sodium *tert*-butoxide (40.4 mg, 0.42 mmol, 1.4 equiv), piperidine (36 μL, 0.36 mmol, 1.2 equiv), anhydrous THF (0.3 mL) were added into the reaction tube in the glovebox. The reaction tube was removed from the glove box and stirred at 100 °C for 45 min. The reaction mixture was then cooled to room temperature and filtered through a plug of silica gel and washed with ethyl acetate (ca. 15 mL). The eluate was concentrated under reduced pressure and the crude material was purified by flash chromatography (hexanes/ethyl acetate = 4:1) to give the pure product **5** as a yellow oil (114.3 mg, 87% yield).
^{1}H NMR (500 MHz, $CDCl_3$) δ 7.78 (d, *J* = 9.0 Hz, 2H'), 7.67 (d, *J* = 9.0 Hz, 2H*), 7.31 – 6.91 (m, 8H* + 8H'), 6.85 (d, *J* = 8.7 Hz, 2H*), 6.80 (d, *J* = 9.0 Hz, 2H'), 6.75 (d, *J* = 9.0 Hz, 2H*), 6.68 (d, *J* = 8.7 Hz, 2H'), 6.12 – 6.02 (m, 1H'), 5.82 (ddd, *J* = 17.1, 10.2, 8.3 Hz, 1H*), 5.09 (ddd, *J* = 13.5, 11.6, 1.3 Hz, 2H'), 4.91 – 4.73 (m, 2H*), 3.84 – 3.69 (m, 4H* + 4H'), 3.59 (t, *J* = 8.8 Hz, 1H*), 3.53 (t, *J* = 9.5 Hz, 1H'), 3.40 (dd, *J* = 16.7, 5.2 Hz, 1H'), 3.36 – 3.15 (m, 3H* + 3H'), 3.04 (dd, *J* = 16.4, 4.6 Hz, 1H*), 1.63 (t, *J* = 11.8 Hz, 2H* + 2H').
^{13}C NMR (126 MHz, $CDCl_3$) δ 197.1, 197.0, 158.3, 157.9, 154.4, 154.4, 143.1, 142.9, 141.2, 140.1, 135.1, 134.8, 130.2, 130.2, 129.4, 129.2, 128.9, 128.7, 128.1, 127.9, 127.0, 126.9, 126.4, 126.0, 115.9, 115.7, 114.1, 113.6, 113.4, 113.3, 56.3, 55.4, 55.2, 54.8, 48.7, 46.8, 46.1, 42.7, 42.6, 25.49, 25.46, 24.5 (two carbons missing due to overlap).
HRMS (ESI) calcd for $C_{30}H_{34}O_2N$ $[M+H]^+$: 440.2584, found: 440.2578.

Synthesis of 2,4,5-triphenyl-4*H*-pyran (6)

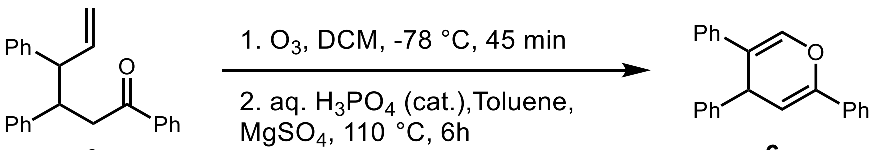

3aa → **6**

Ketone **3aa** (32.6 mg, 0.1 mmol, 1.0 equiv) was dissolved in CH_2Cl_2 (10 mL) in a round bottom flask and cooled to -78 °C. A stream of O_3 is bubbled through the solution until a blue/gray color persists (ca. 45 min). The reaction is quenched by adding dimethyl sulfide (1 mL) to the flask and allowing the reaction mixture to slowly warm to room temperature. Volatiles were evaporated under reduced pressure, and the resulting residue was used directly in the next step.

The following reaction was based on a literature procedure.[20] The crude residue was transferred to a reaction tube equipped with a magnetic stir bar and dissolved in 1.6 mL of anhydrous toluene. $MgSO_4$ (ca. 100 mg) and catalytic phosphoric acid (85% aq. solution, ca. 10 drops) were added under air. The reaction tube was capped with a Teflon/silicone septum screw cap and was stirred at 110 °C for 6 h. The reaction mixture was cooled to room temperature and extracted with ethyl acetate. The combined organic layers were washed with water and brine, dried over $MgSO_4$, and evaporated under reduced pressure. The crude product was purified by flash chromatography (hexanes/ethyl acetate = 100:1) to give the pure product **6** as a white solid (13.9 mg, 45% yield).

^{1}H NMR (500 MHz, $CDCl_3$) δ 7.61 (d, J = 7.9 Hz, 2H), 7.39 – 7.13 (m, 15H), 5.59 (d, J = 4.6 Hz, 1H), 4.65 (d, J = 4.5 Hz, 1H).

^{13}C NMR (126 MHz, $CDCl_3$) δ 146.9, 145.4, 139.2, 137.4, 134.0, 128.8, 128.5, 128.5, 128.4, 128.2, 126.9, 126.7, 126.0, 124.7, 116.2, 102.0, 40.7.

HRMS (ESI) calcd for $C_{23}H_{19}O$ $[M+H]^+$: 311.1430, found: 311.1428.

4. X-Ray determination of diastereomer assignments

Sample 3aa was diastereomerically enriched through repeated recrystallization from diethyl ether and hexanes.

NMR of the material from the diffractable crystal showing >20:1 diastereomer enrichment

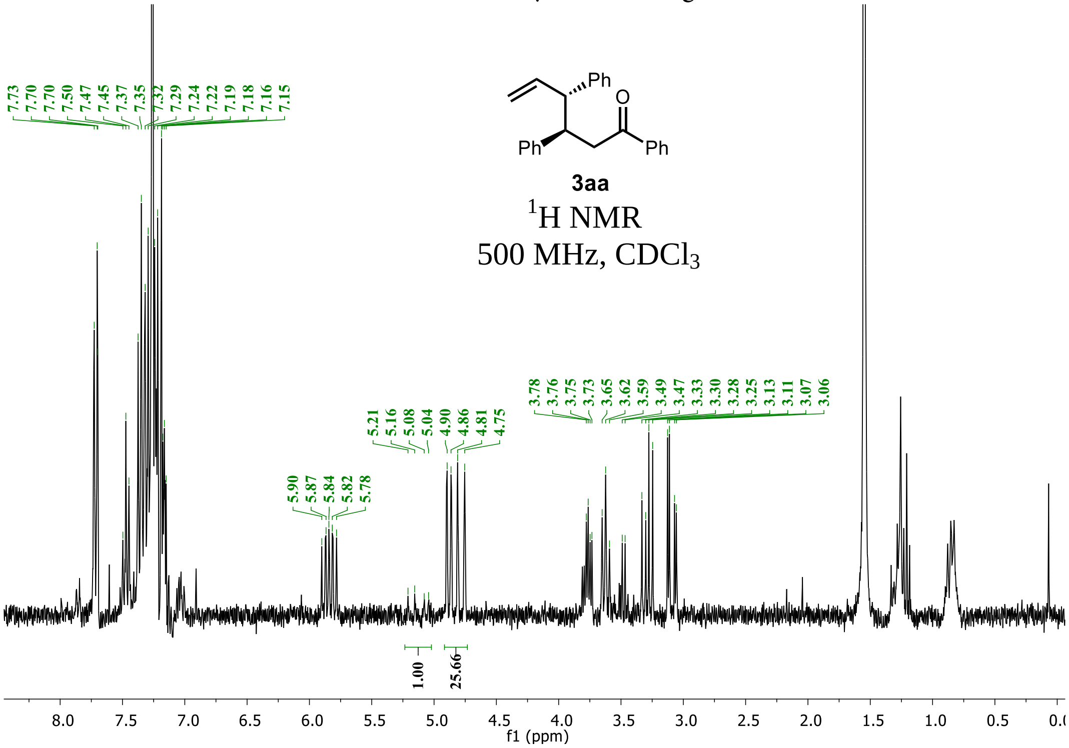

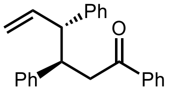

3aa
CCDC 2226347

≡

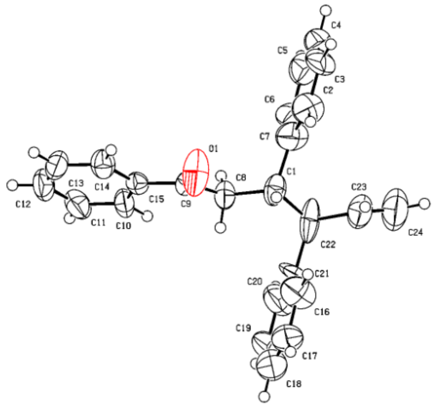

Structure of diastereomerically enriched **3aa**. Ellipsoid contour probability is set at 50%.

Datablock: Scrivener9622_0m_a

Bond precision:	C-C = 0.0135 A		Wavelength=1.54178
Cell:	a=37.373(2)	b=5.7738(4)	c=17.9306(10)
	alpha=90	beta=106.121(4)	gamma=90
Temperature:	296 K		

	Calculated	Reported
Volume	3717.0(4)	3717.0(4)
Space group	C 2/c	C 2/c
Hall group	-C 2yc	-C 2yc
Moiety formula	C24 H22 O	?
Sum formula	C24 H22 O	C24 H22 O
Mr	326.42	326.41
Dx,g cm-3	1.167	1.167
Z	8	8
Mu (mm-1)	0.533	0.533
F000	1392.0	1392.0
F000'	1395.70	
h,k,lmax	32,5,15	32,5,15
Nref	1289	1284
Tmin,Tmax	0.987,0.995	0.600,0.800
Tmin'	0.928	

Correction method= # Reported T Limits: Tmin=0.600 Tmax=0.800
AbsCorr = MULTI-SCAN

Data completeness= 0.996 Theta(max)= 42.374

R(reflections)= 0.0605(702) wR2(reflections)= 0.1588(1284)

S = 0.947 Npar= 226

5. Reaction optimization

Table 1. Solvent, Base, and Temperature Optimization

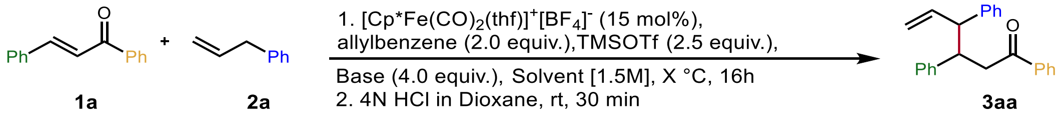

Entry	Solvent	Base	***T*** (°C)	Yield[a] (%)
1	PhCl	*sym*-Col	80	46
2	1,2-Difluorobenzene	*sym*-Col	80	36
3	Chloroform	*sym*-Col	80	35
4	DCE	*sym*-Col	80	35
5	Trifluorotoluene	*sym*-Col	80	37
6	Toluene	*sym*-Col	80	52
7	1,2-Dichlorobenzene	*sym*-Col	80	46
8	Chlorocyclohexene	*sym*-Col	80	42
9	Toluene	Lutidine	80	20
10	Toluene	4-Cl-Lutidine	80	21
11	Toluene	TMPH	80	29
12	Toluene	Tetramethylpiperazine	80	0
13	Toluene	PMP	80	10
14	Toluene	2,6-diethylpyridine	80	16
15	Toluene	Collidine	80	39
16	Toluene	4-OMe-Lutidine	80	38
17	Toluene	*sym*-Col	60	26
18	Toluene	*sym*-Col	80	**46**
19	Toluene	*sym*-Col	100	35

[a]Yields determined by ^{1}H NMR using 2,4-dinitrobenzene as the internal standard. TMPH = 2,2,6,6-tetramethylpiperidine, *sym*-Col = 2,4,6-trimethyl pyridine.

Table 2. Stoichiometry and Silyl Triflate Investigation – Early halt to observe Chalcone Decomposition

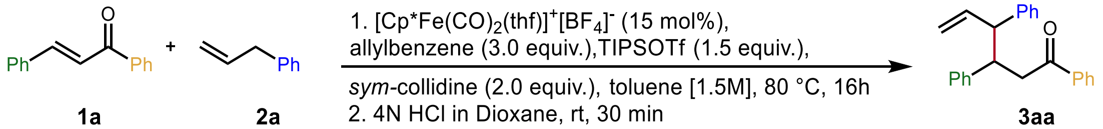

Entry	Ratio (**1a**/**2a**/SiR_3/Base)	Silyl Triflate	Time	NMR Chalcone (%)	NMR Yield (%)[a]

1	1/3/2.5/4	TMSOTf	3.5	-	44
2	1/3/2.5/3	TMSOTf	3.5	<5	36
3	1/3/1.5/3	TMSOTf	3.5	<5	40
4	1/3/1.5/2	TMSOTf	3.5	12/10 (180-1)	43/39
5	1/3/2.5/3	TESOTf	3.5	28	29
6	1/3/2.5/3	TESOTf	16	0	50
7	1/3/1.5/2	TESOTf	16	0	61
8	1/3/1.5/2	TIPSOTf	16	0	76
9	1/3/1.5/2	TBSOTf	16	4	48
10	1/3/1.5/2	TBDPSOTf	16	4	65

[a]Yields determined by ^{1}H NMR using 2,4-dinitrobenzene as the internal standard.

Table 3. Additional Stoichiometry and Catalyst Optimization

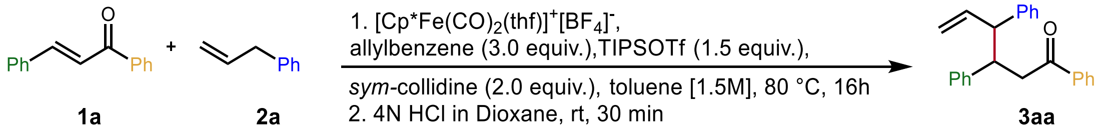

Entry	Ratio (**1a**/**2a**/SiR$_3$/Base)	Catalyst Loading	Catalyst Ligand	NMR Chalcone (%)	NMR Yield (%)[a]
1	1/3/1.5/2	20	Cp*	0	76
2	1/3.5/1.5/2	20	Cp*	0	69
3	1/3/1.1/3	20	Cp*	13	48
4	1/3/1.1/1.5	20	Cp*	16	52
5	1/3/1.5/2	15	Cp*	0	69
6	1/3/1.5/2	10	Cp*	12	31
7	1/3/1.5/2	20	PentapropylCp	51	25
8	1/3/1.5/2	20	TetramethylCp	11	34

[a]Yields determined by ^{1}H NMR using 2,4-dinitrobenzene as the internal standard.

Table 4. Additive Optimization: LiNTf$_2$

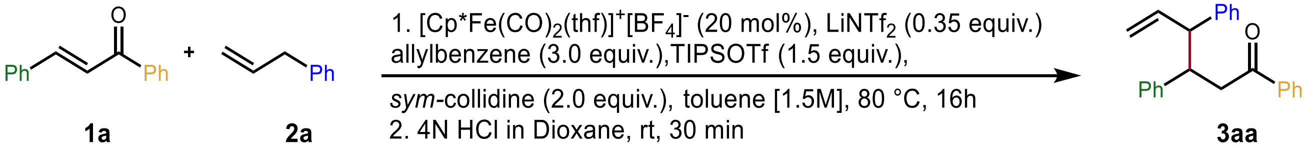

Entry	Additive (equiv.)	NMR Yield (%)[a]
1	0.3	79
2	0.2	75

3	0.4	81
4	0.35	82

[a]Yields determined by ^{1}H NMR using 2,4-dinitrobenzene as the internal standard.

6. Unsuccessful substrates

Pronucleophile substrates:

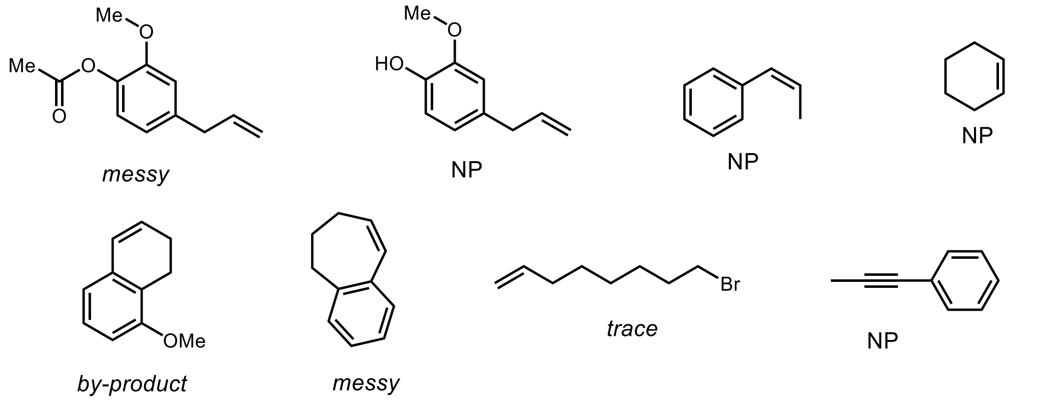

Unsucessful Michael acceptors:

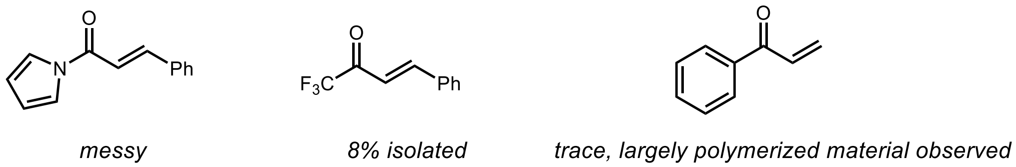

7. Copies of NMR spectra of products and substrates

Copies of NMR spectra of products: Michael acceptor scope

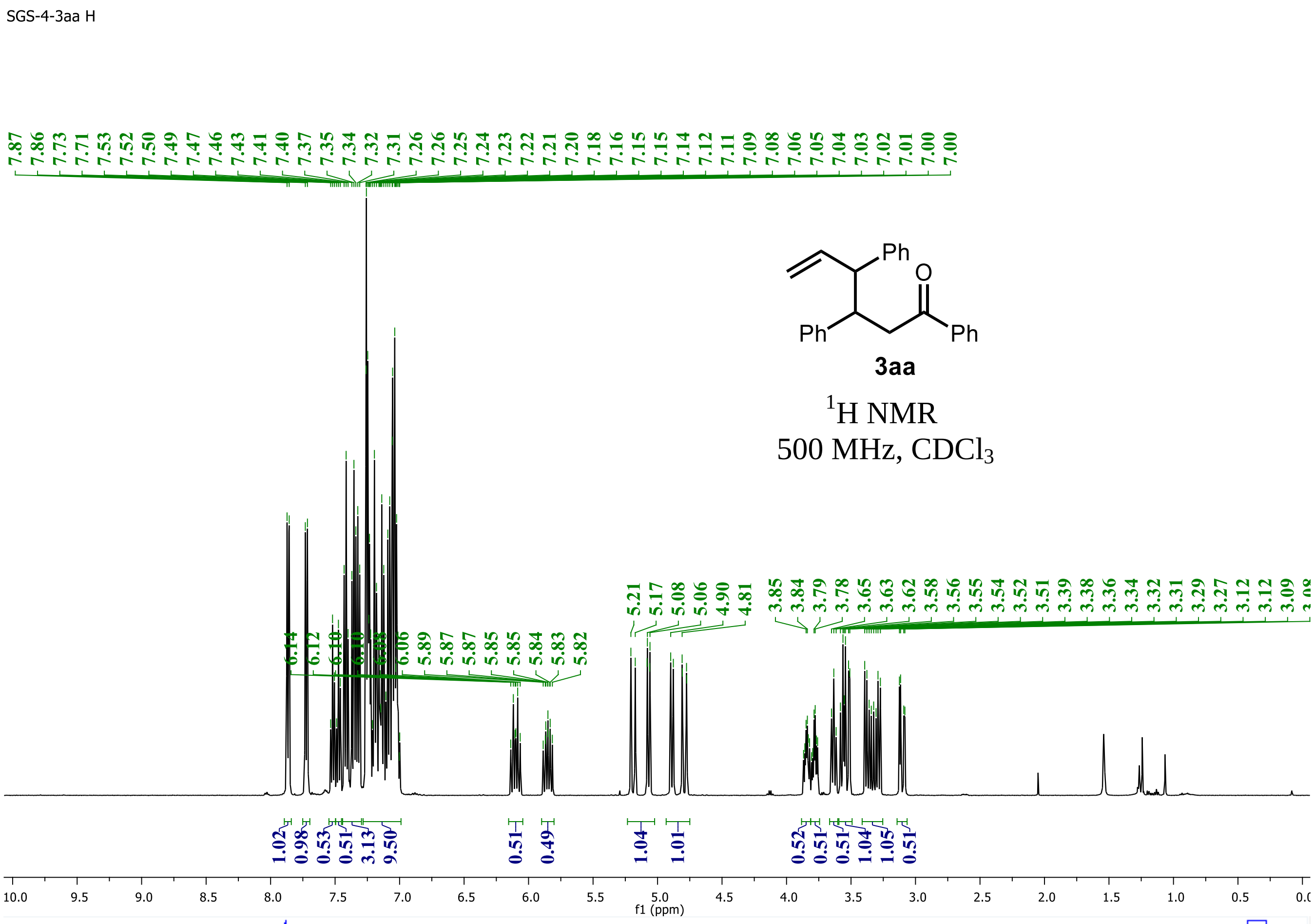

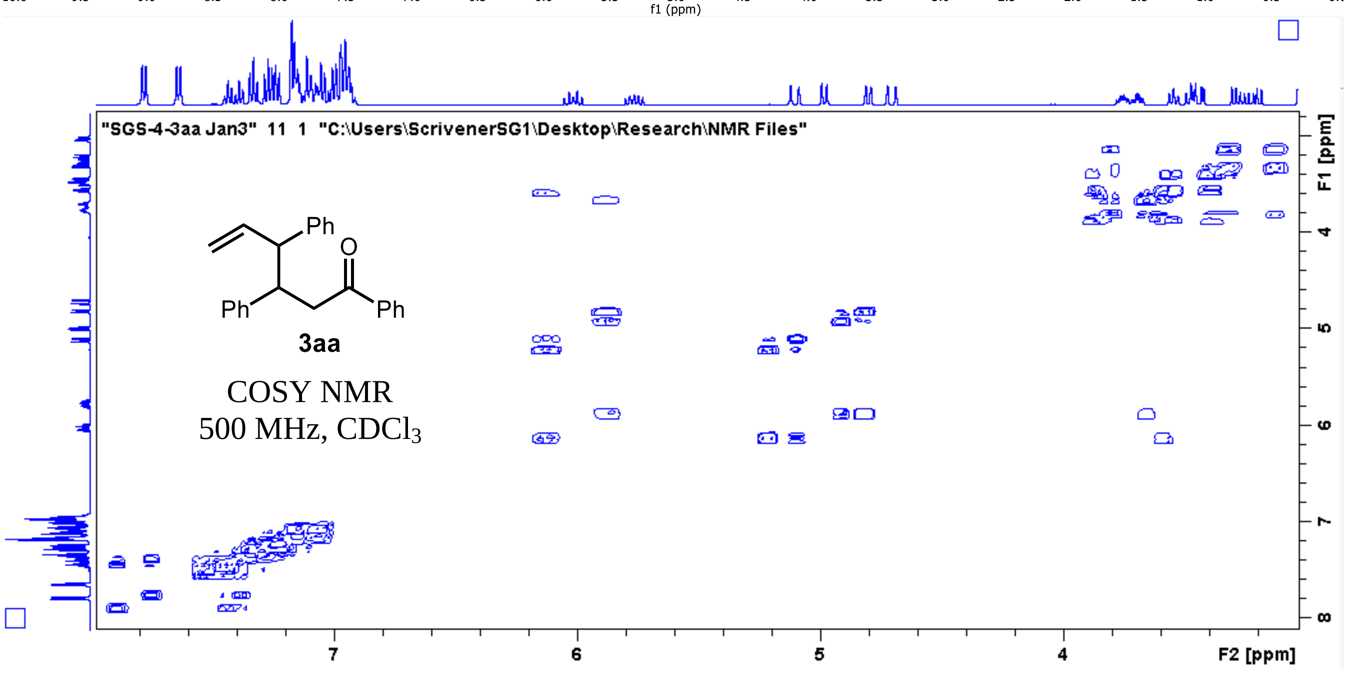

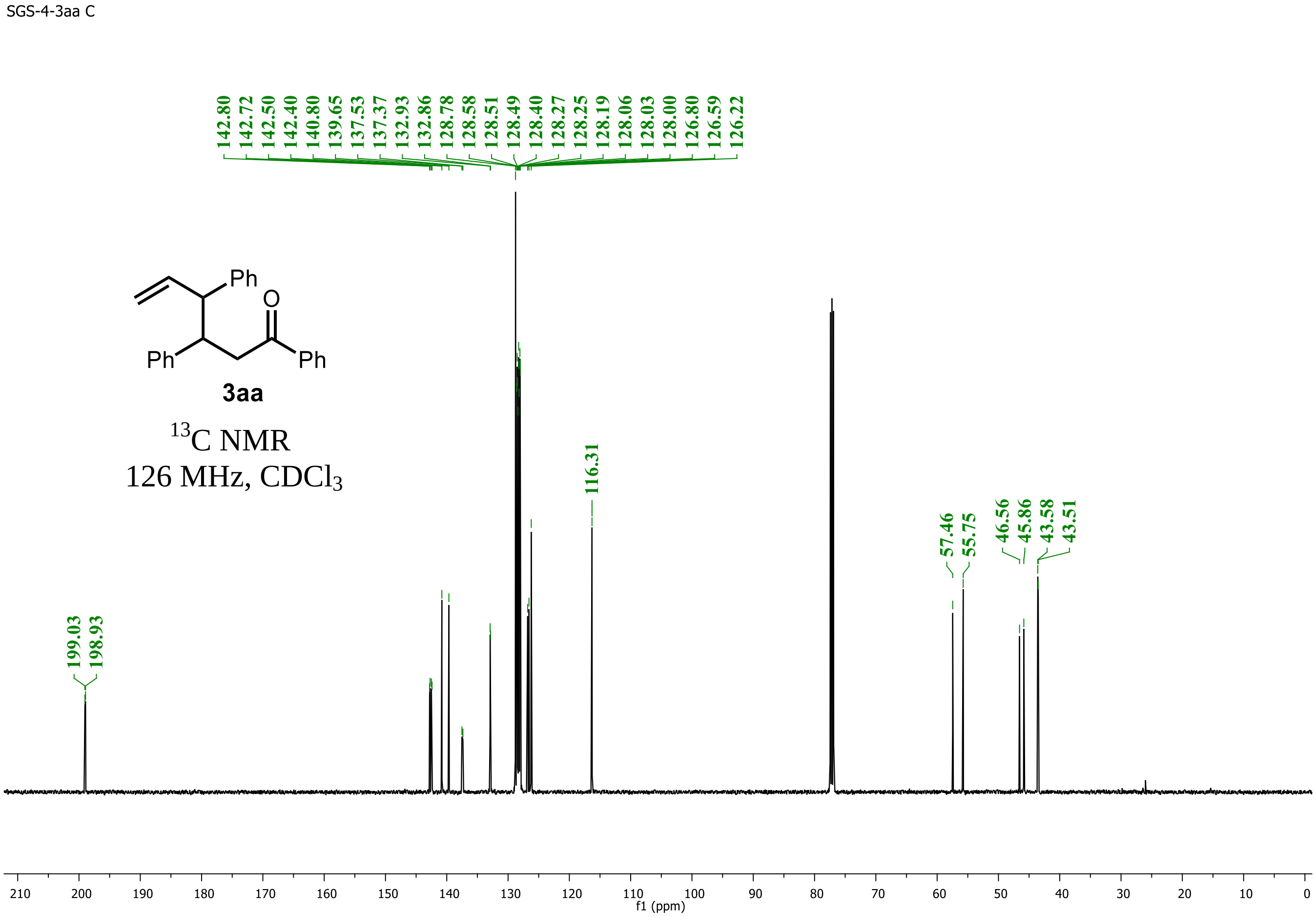
SGS-4-3aa C
142.80 142.72 142.50 142.40 140.80 139.65 137.53 137.37 132.93 132.86 128.78 128.58 128.51 128.49 128.40 128.27 128.25 128.19 128.06 128.03 128.00 126.80 126.59 126.22
116.31
199.03 198.93
57.46 55.75
46.56 45.86 43.58 43.51
Ph
O
Ph
Ph
3aa
^{13}C NMR
126 MHz, $CDCl_3$
210 200 190 180 170 160 150 140 130 120 110 100 90 80 70 60 50 40 30 20 10 0
f1 (ppm)

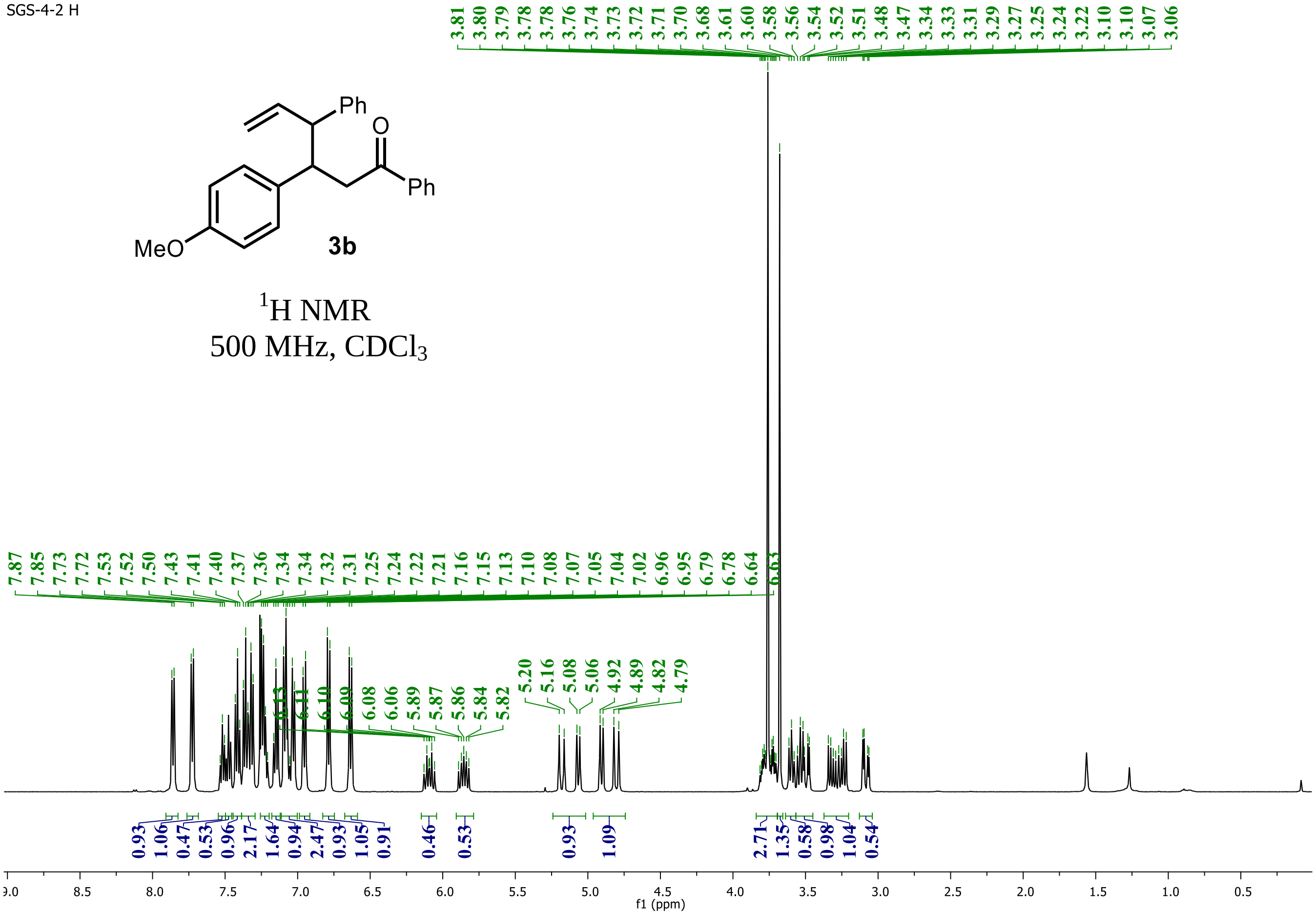
SGS-4-2 H
3.81 3.80 3.79 3.78 3.78 3.76 3.74 3.73 3.72 3.71 3.70 3.68 3.61 3.60 3.58 3.56 3.54 3.52 3.51 3.48 3.47 3.34 3.33 3.31 3.29 3.27 3.25 3.24 3.22 3.10 3.10 3.07 3.06
7.87 7.85 7.73 7.72 7.53 7.52 7.50 7.43 7.41 7.40 7.37 7.36 7.34 7.34 7.32 7.31 7.25 7.24 7.22 7.21 7.16 7.15 7.13 7.10 7.08 7.07 7.05 7.04 7.02 6.96 6.95 6.79 6.78 6.64 6.63
6.13 6.11 6.10 6.09 6.08 6.06 5.89 5.87 5.86 5.84 5.82
5.20 5.16 5.08 5.06 4.92 4.89 4.82 4.79
Ph
O
Ph
MeO
3b
^{1}H NMR
500 MHz, $CDCl_3$
0.93 1.06 0.47 0.53 0.96 2.17 1.64 0.94 2.47 0.93 1.05 0.91 0.46 0.53 0.93 1.09 2.71 1.35 0.58 0.98 1.04 0.54
9.0 8.5 8.0 7.5 7.0 6.5 6.0 5.5 5.0 4.5 4.0 3.5 3.0 2.5 2.0 1.5 1.0 0.5
f1 (ppm)

SGS-4-2 C

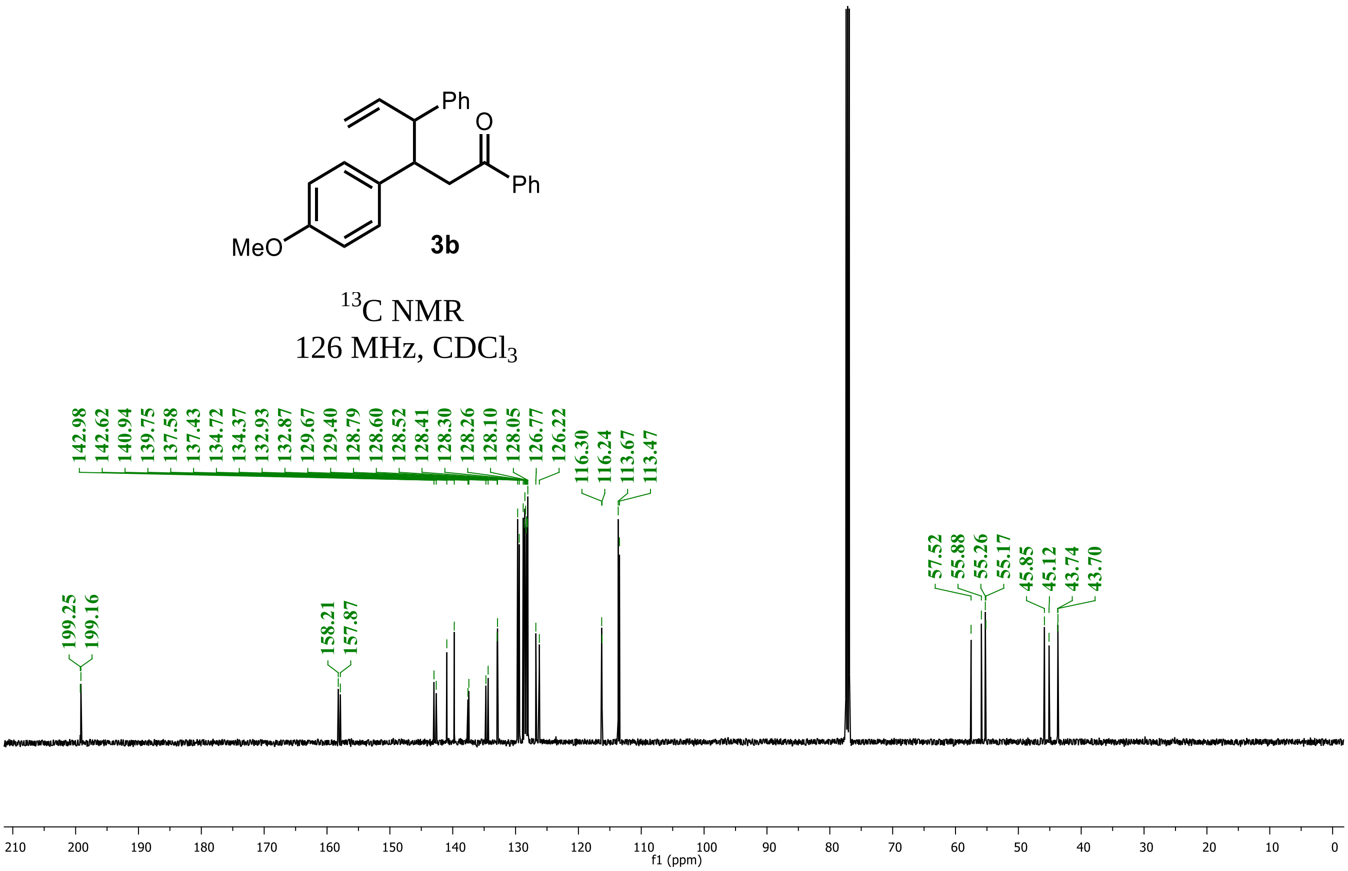

SGS-4-32 H

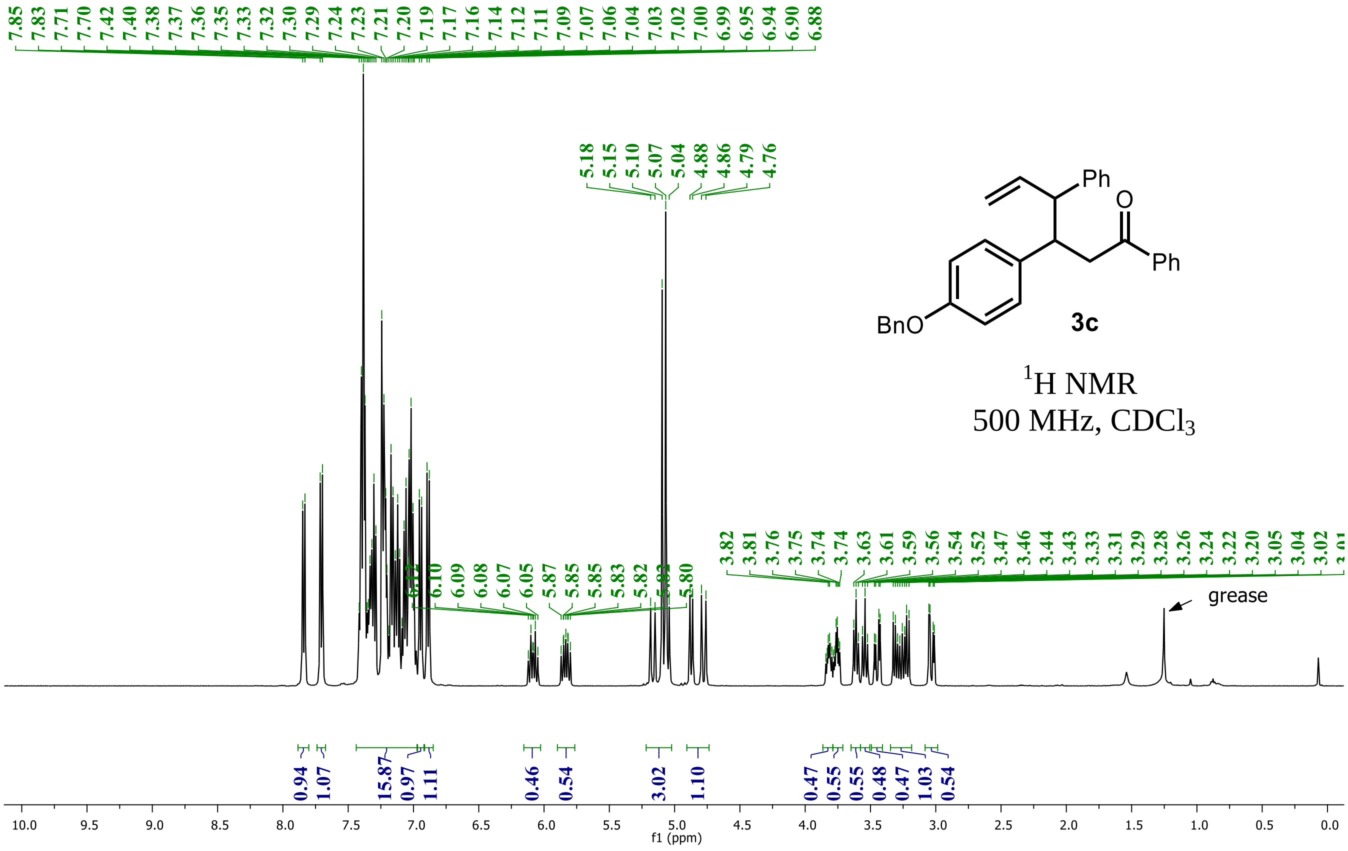

SGS-4-32 C

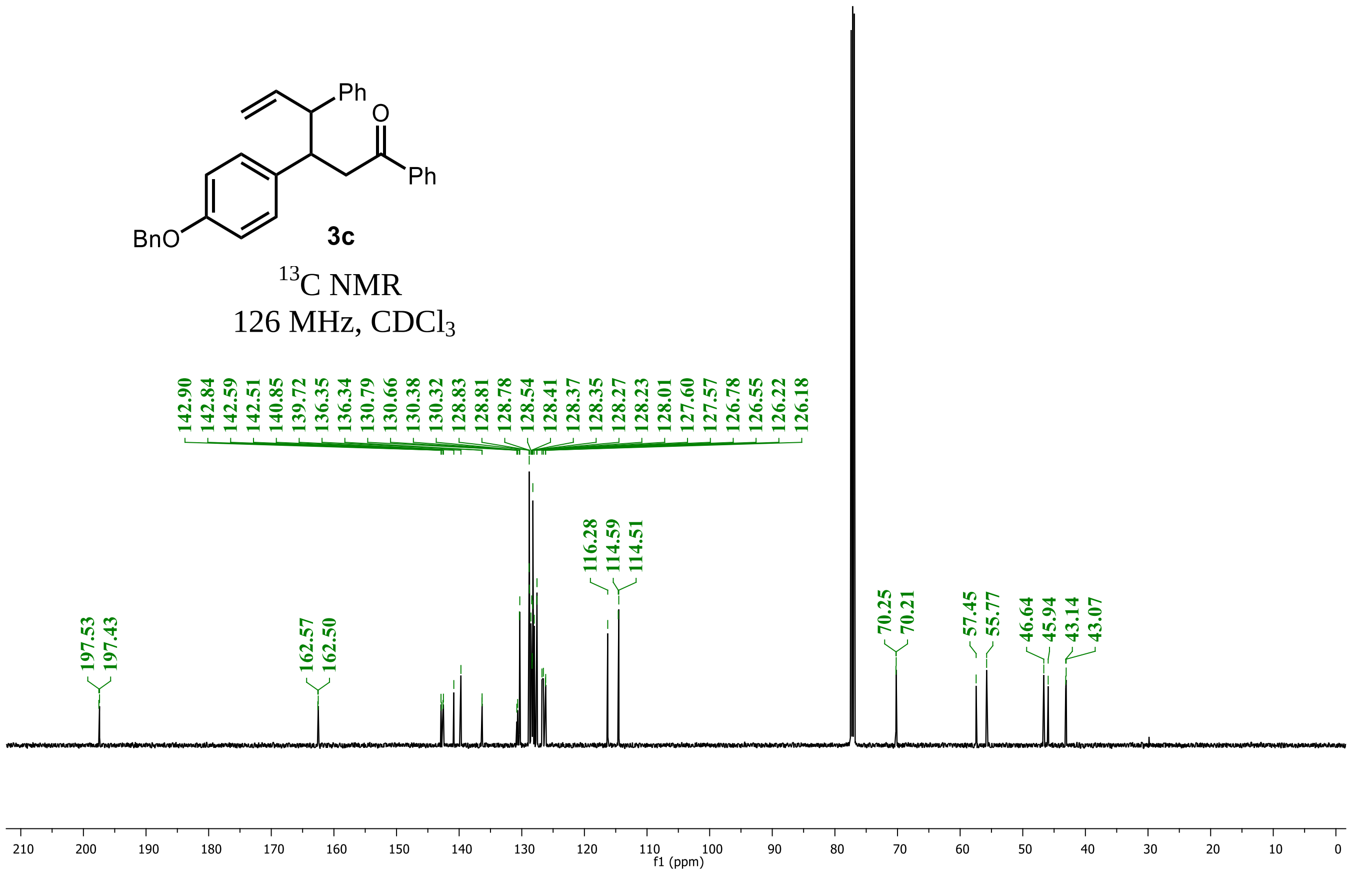

SGS-4-26 1H

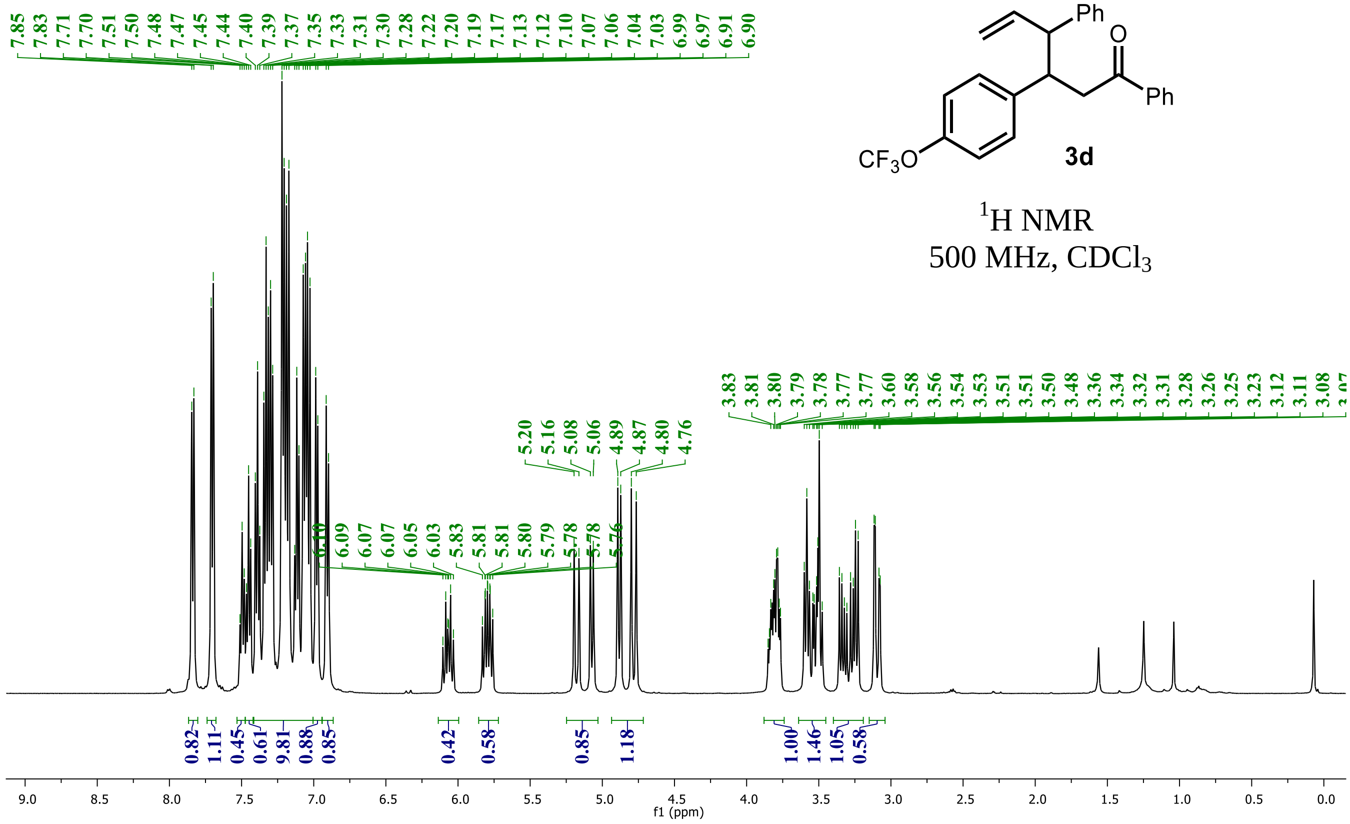

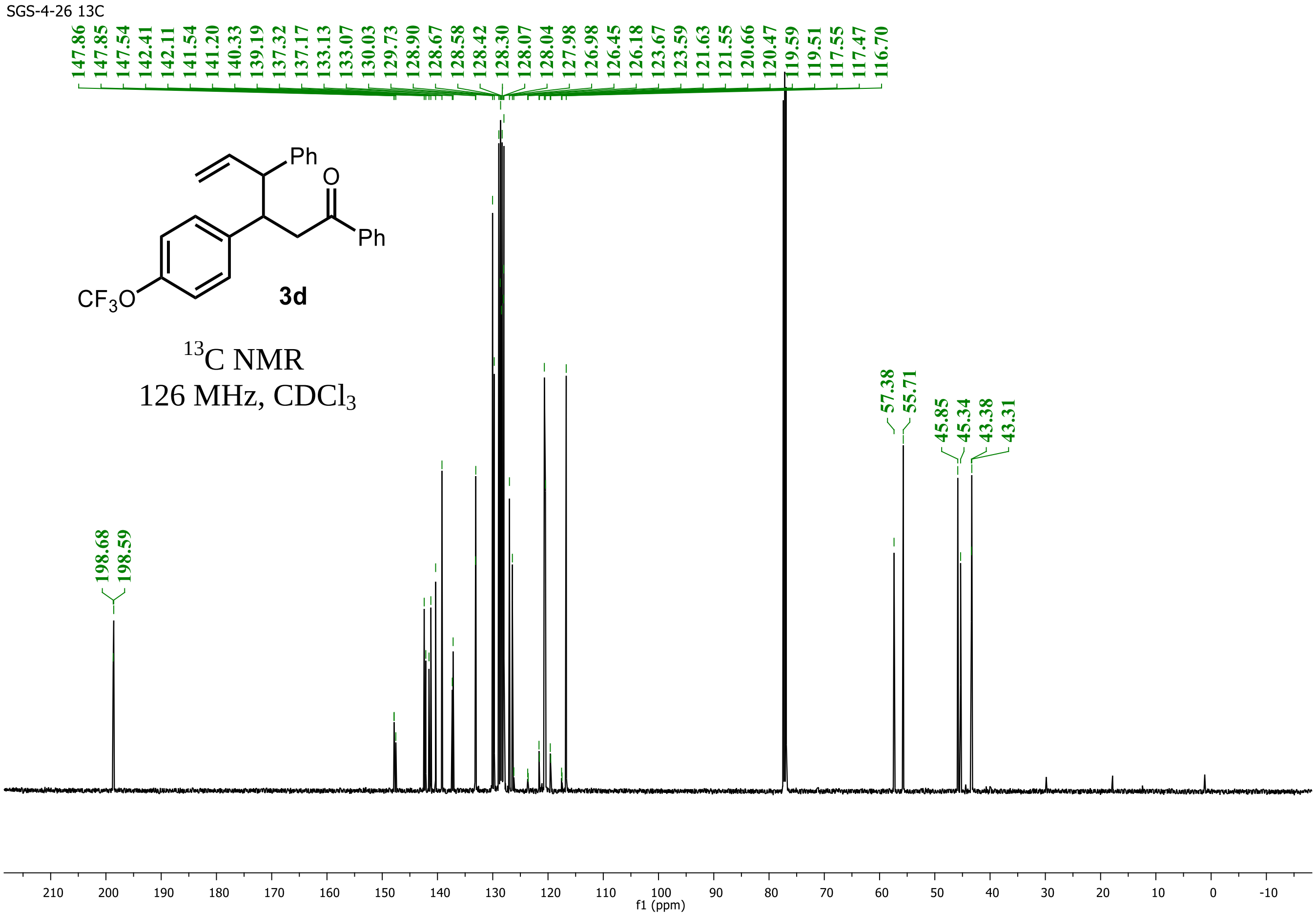
SGS-4-26 13C
3d
^{13}C NMR
126 MHz, CDCl$_3$
f1 (ppm)

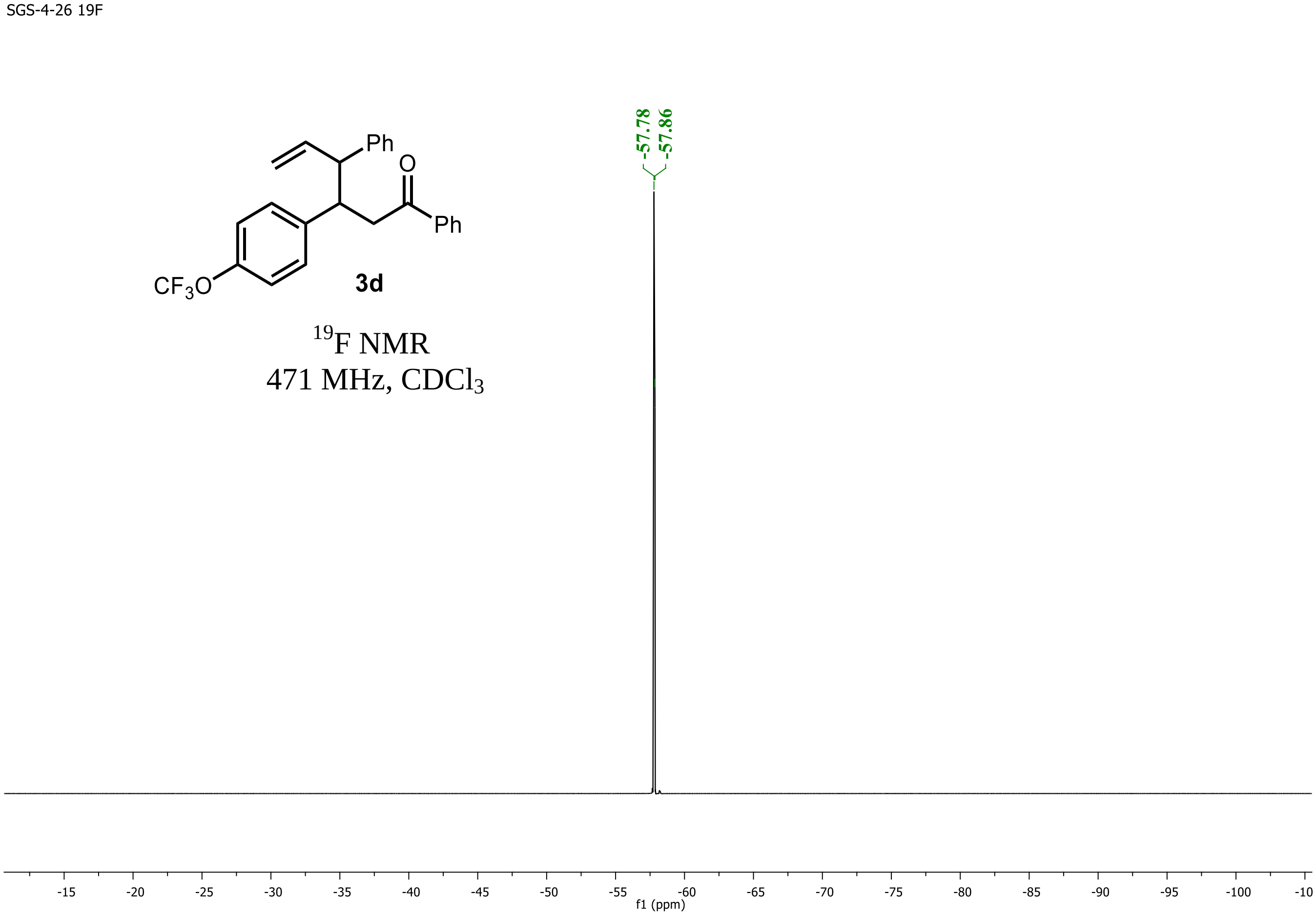
SGS-4-26 19F
3d
^{19}F NMR
471 MHz, CDCl$_3$
-57.78
-57.86
f1 (ppm)

SGS-4-1 Proton

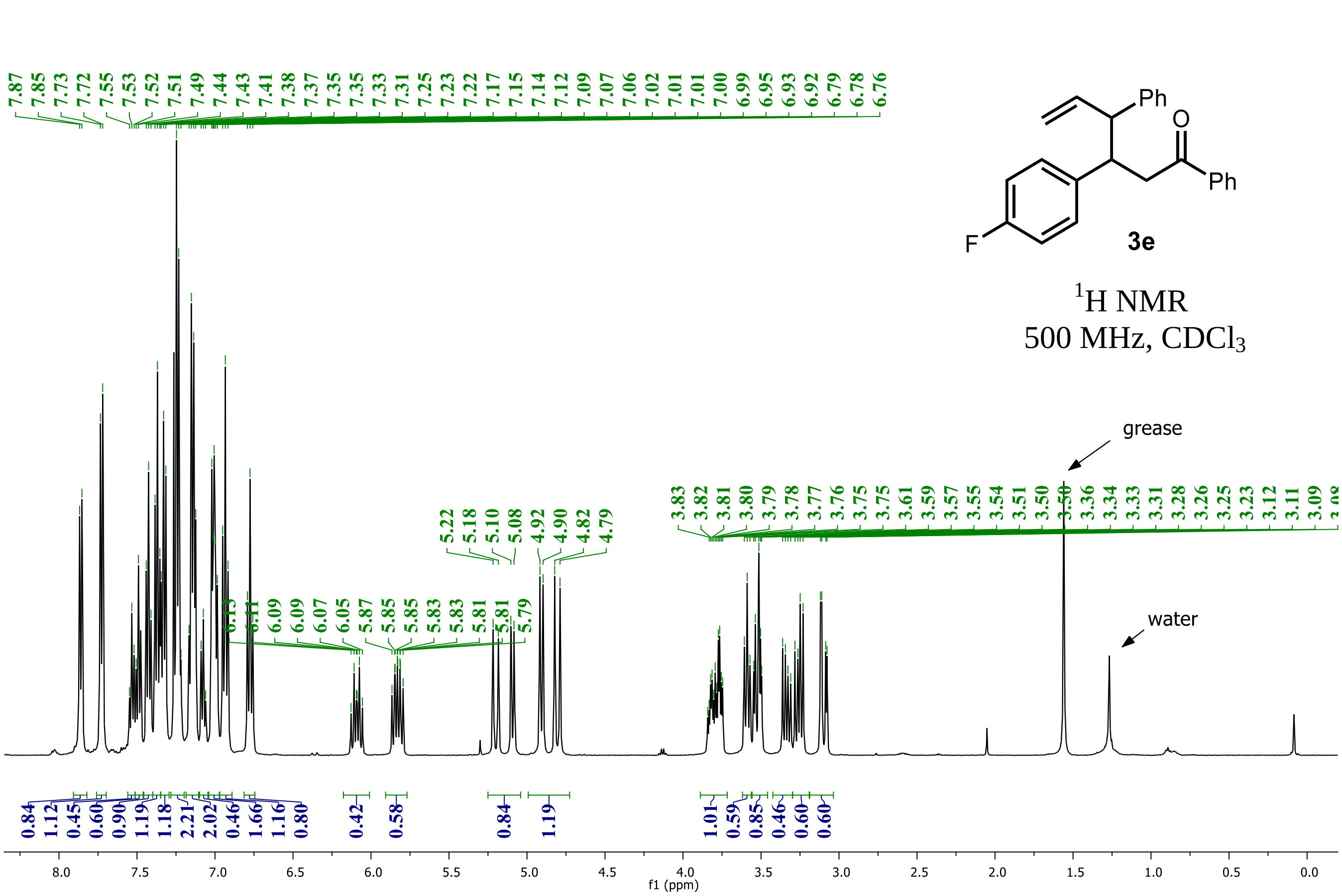

SGS-4-1 C

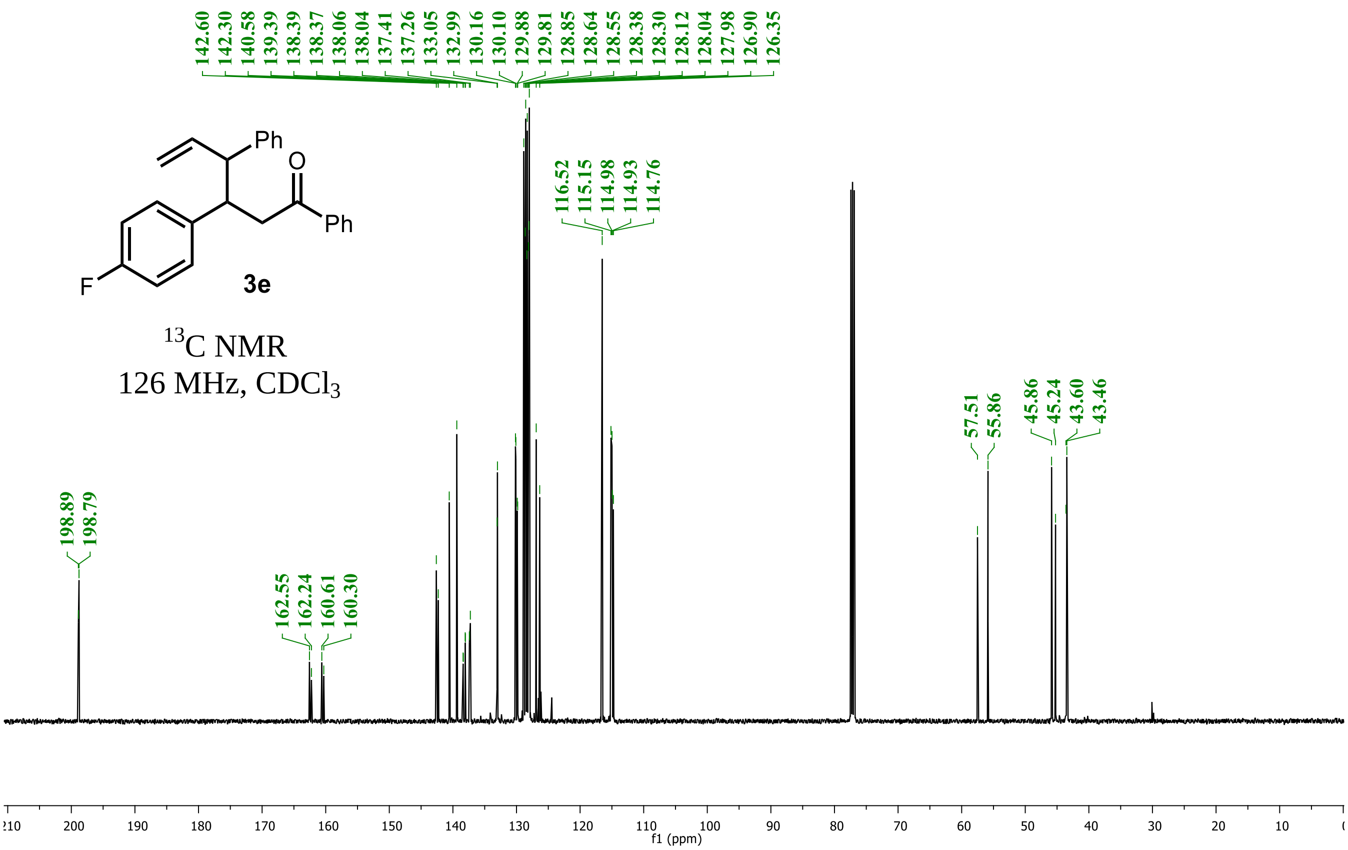

SGS-4-1 F

3e

^{19}F NMR
471 MHz, $CDCl_3$

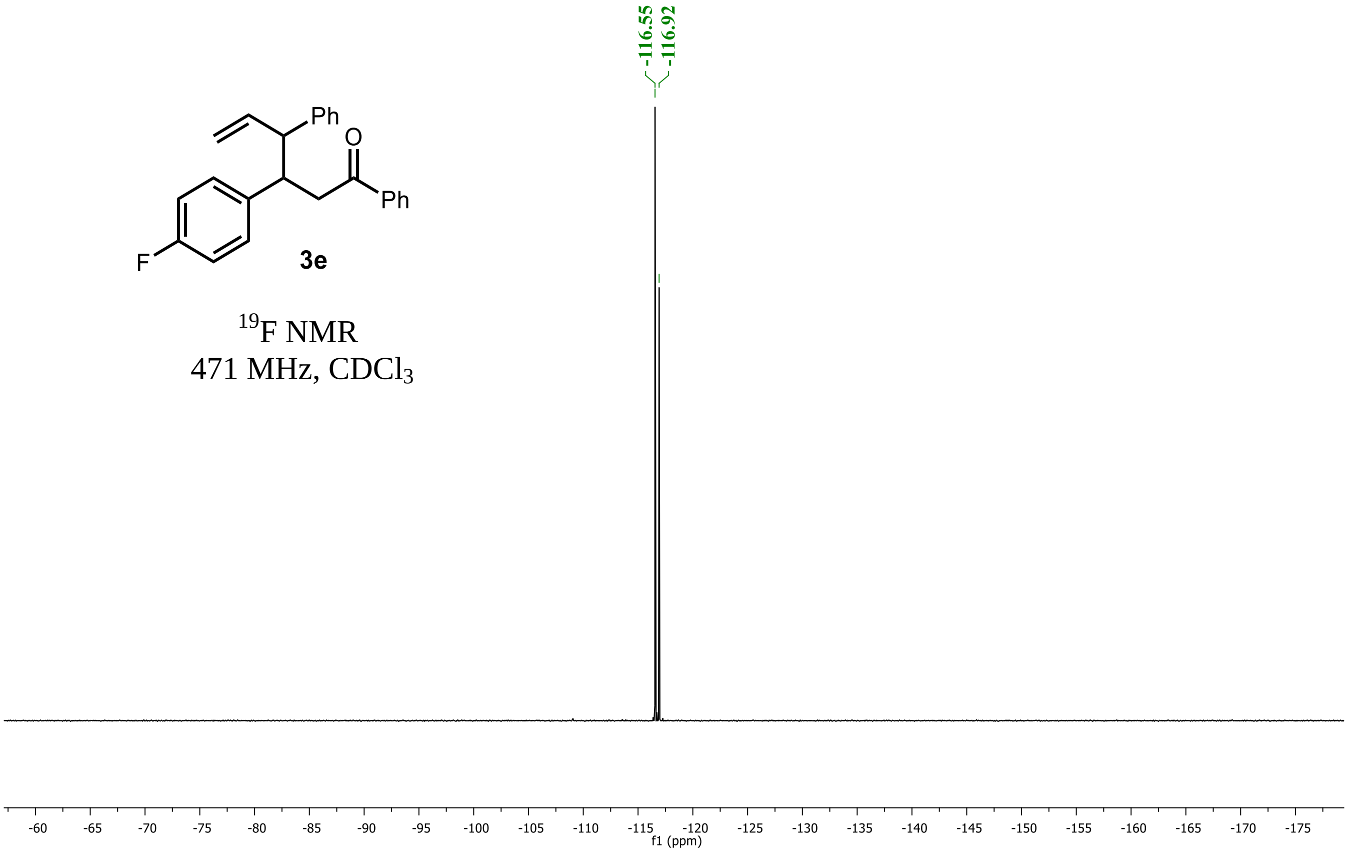

SGS-4-3 H

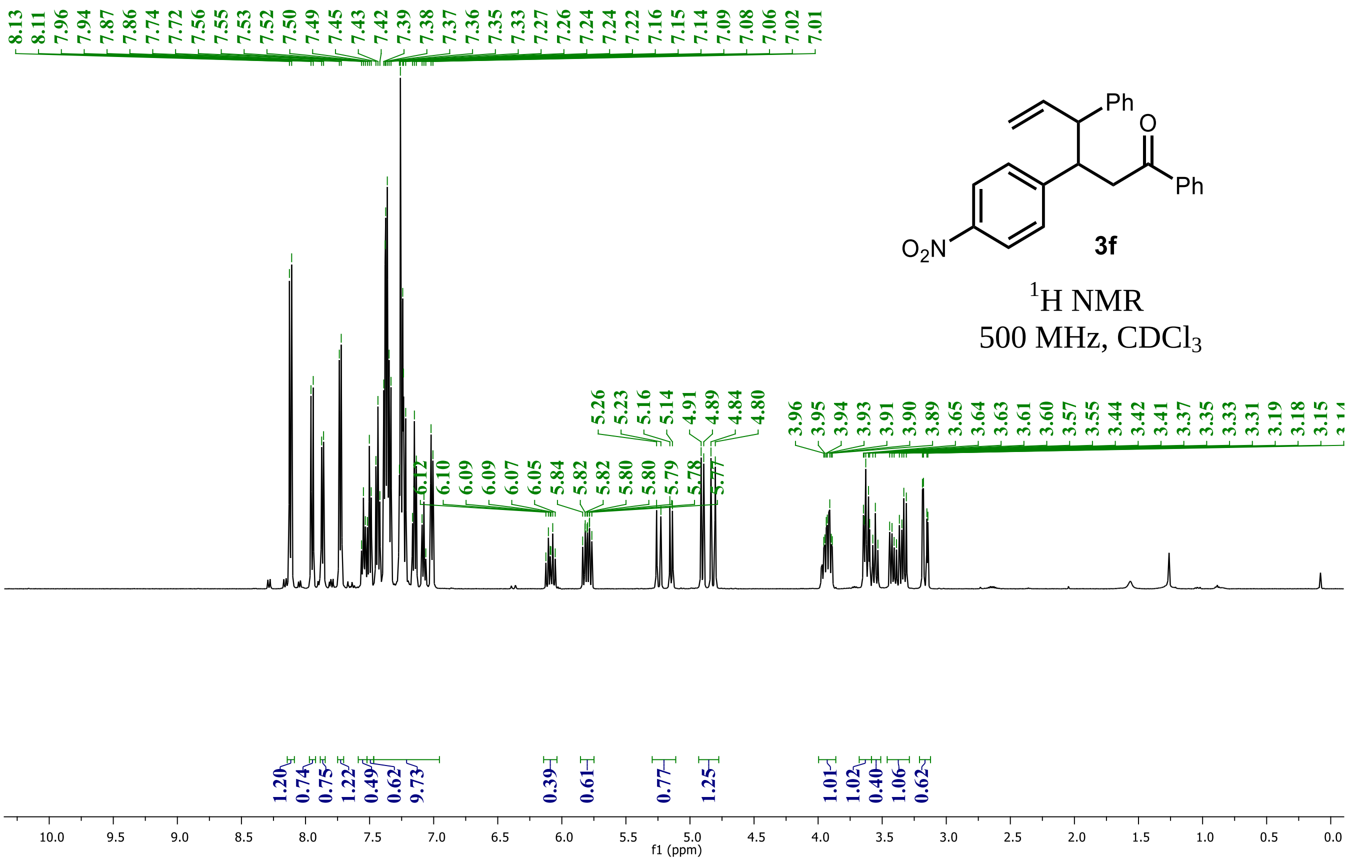

SGS-4-3 C

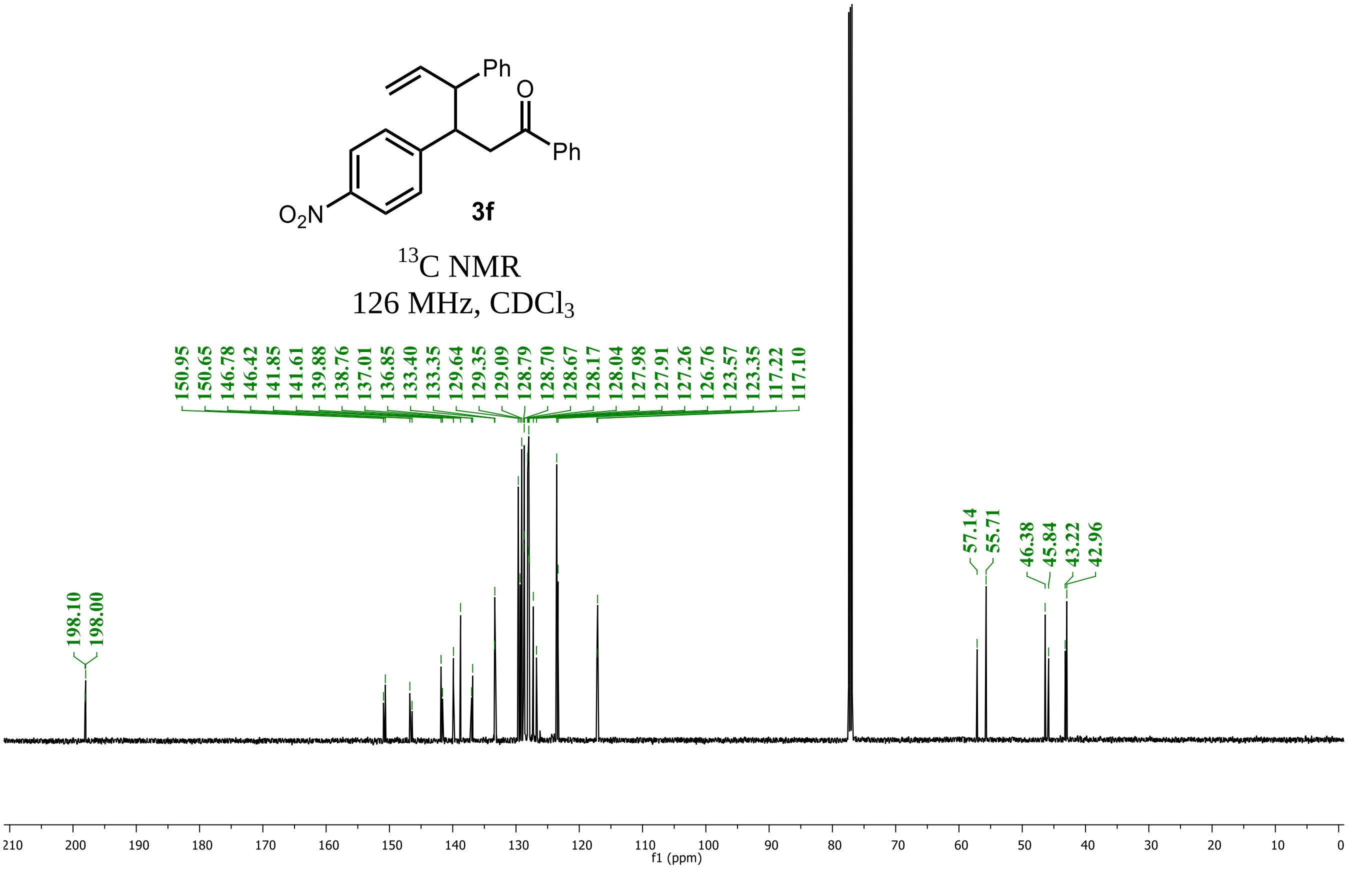

SGS-4-20 H

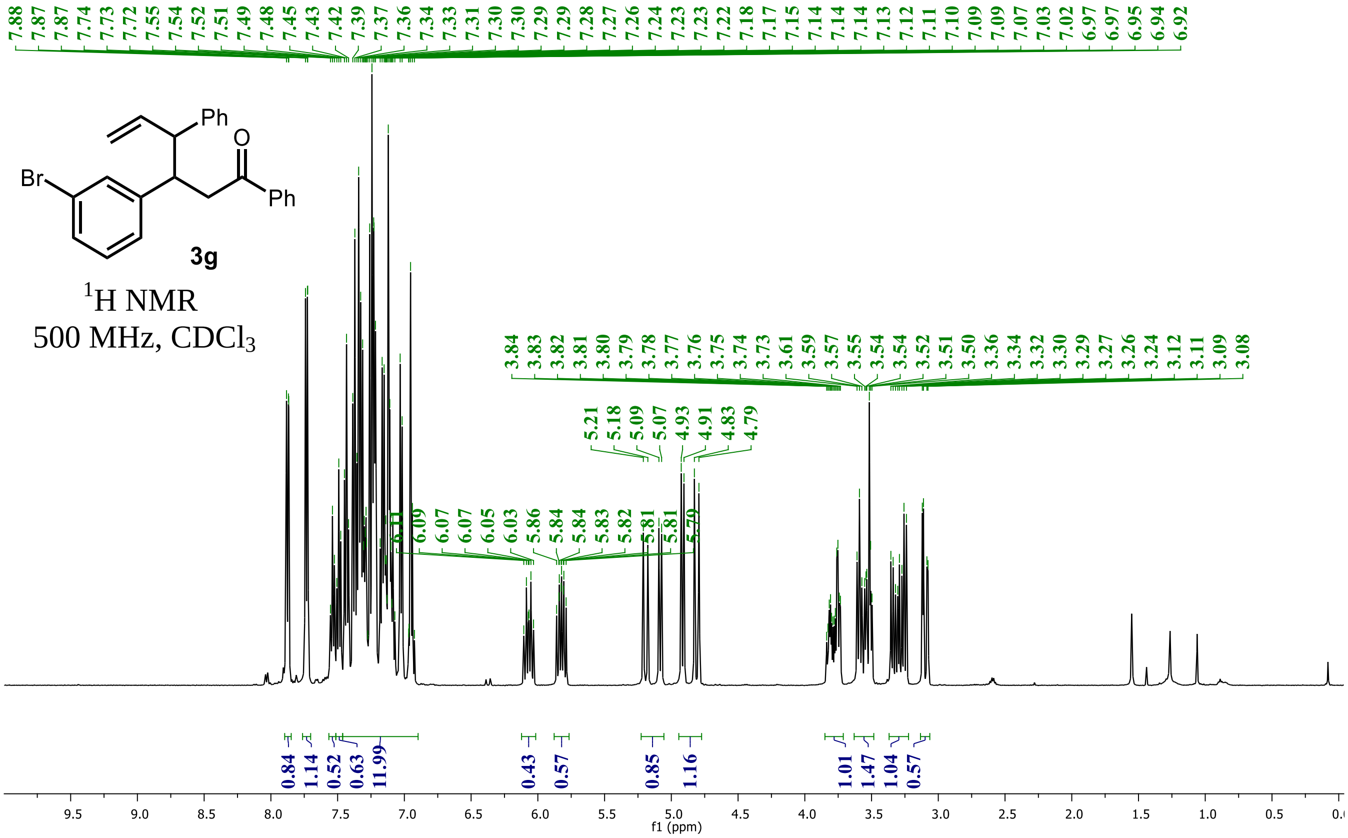

SGS-4-20 C

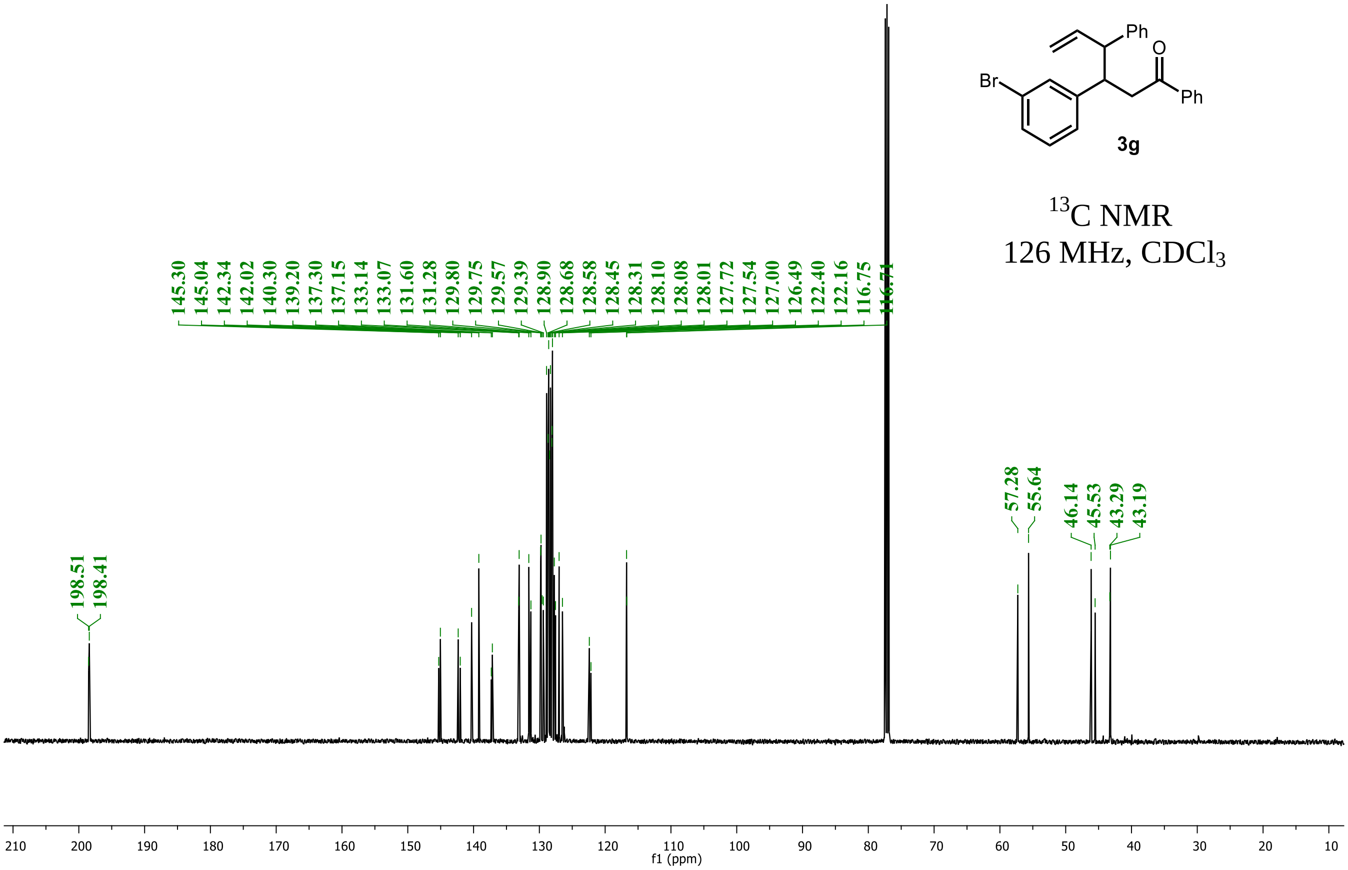

SGS-4-28 1H

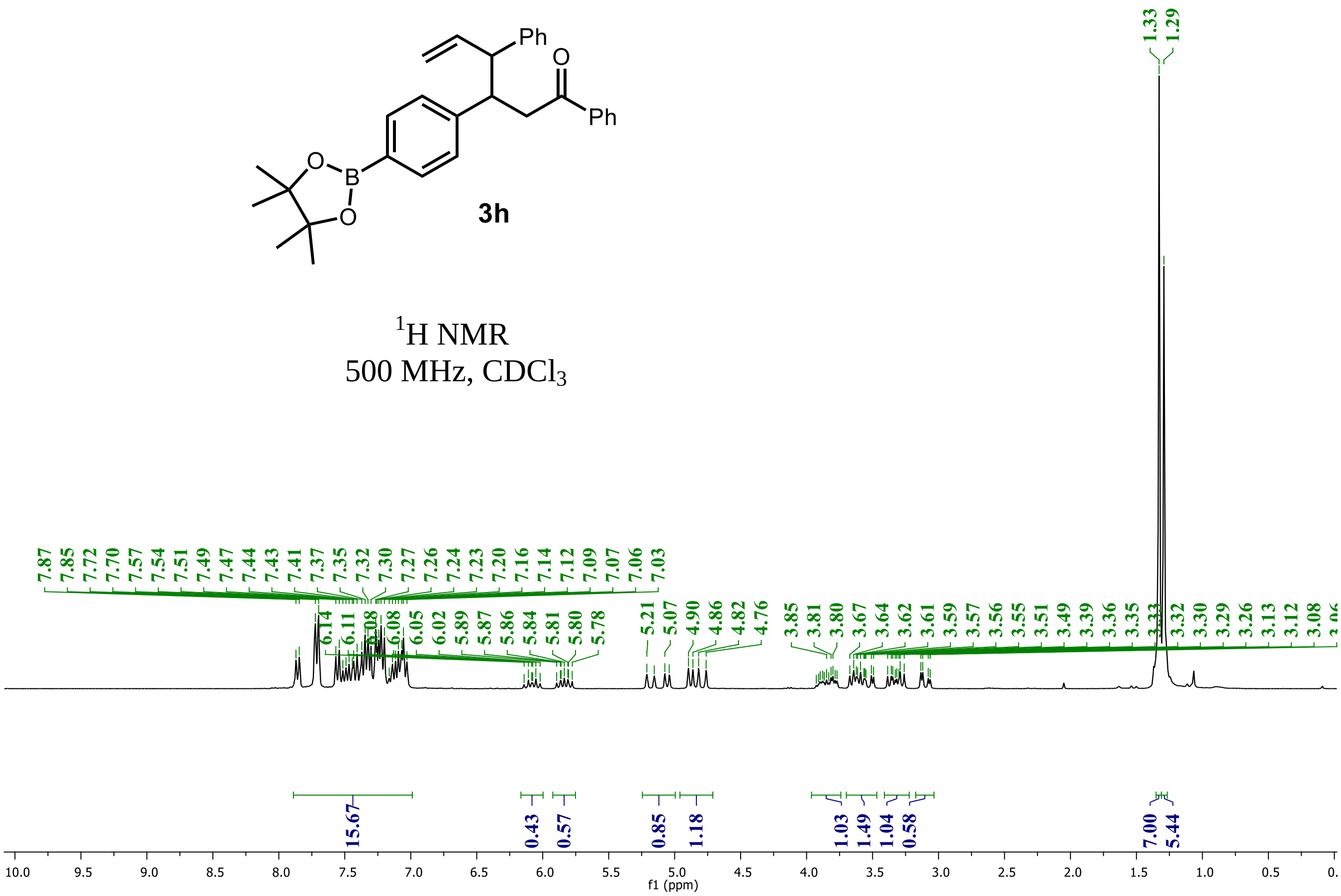

SGS-4-28 (p-1)_11

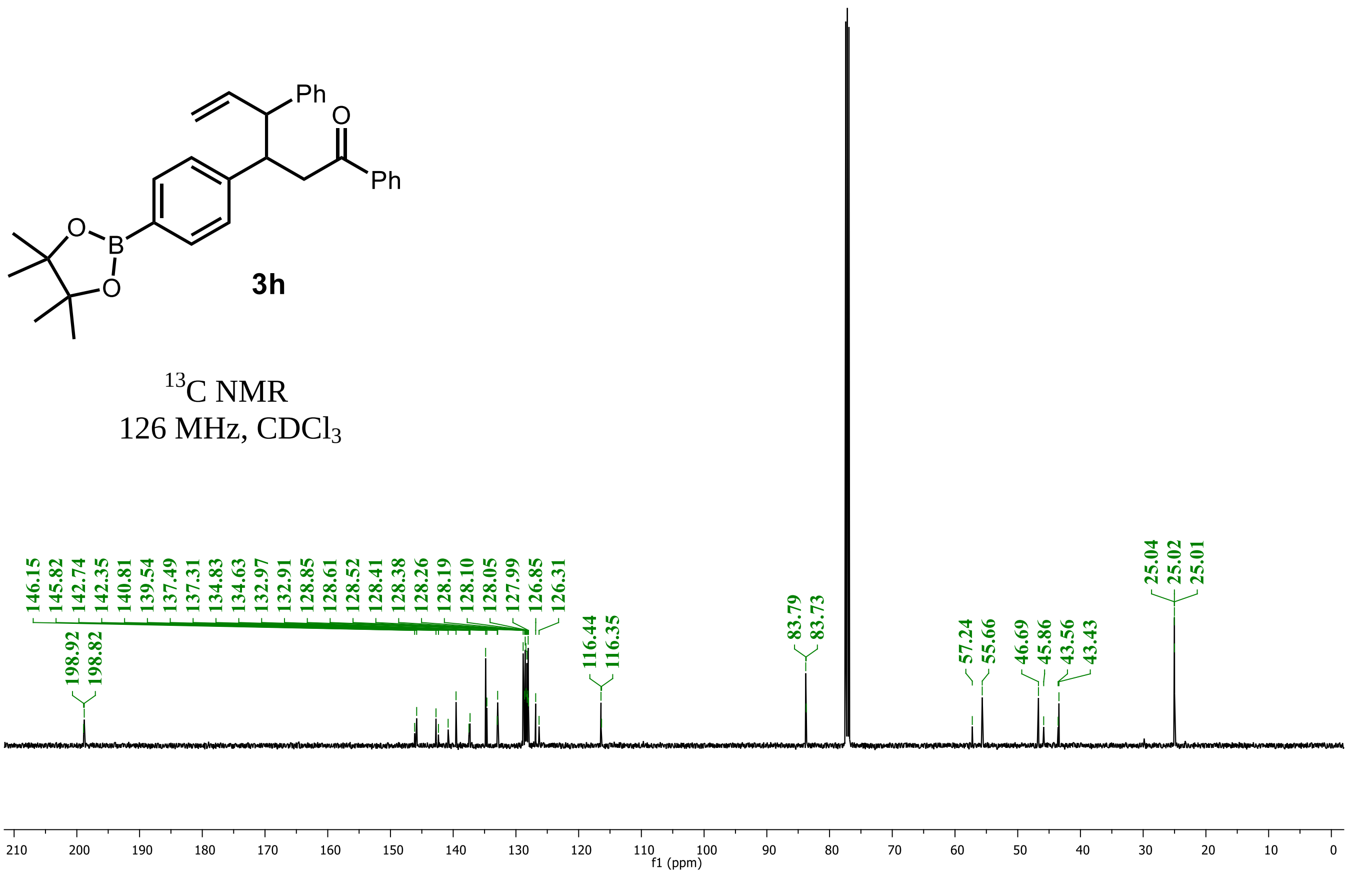

SGS-4-6 H

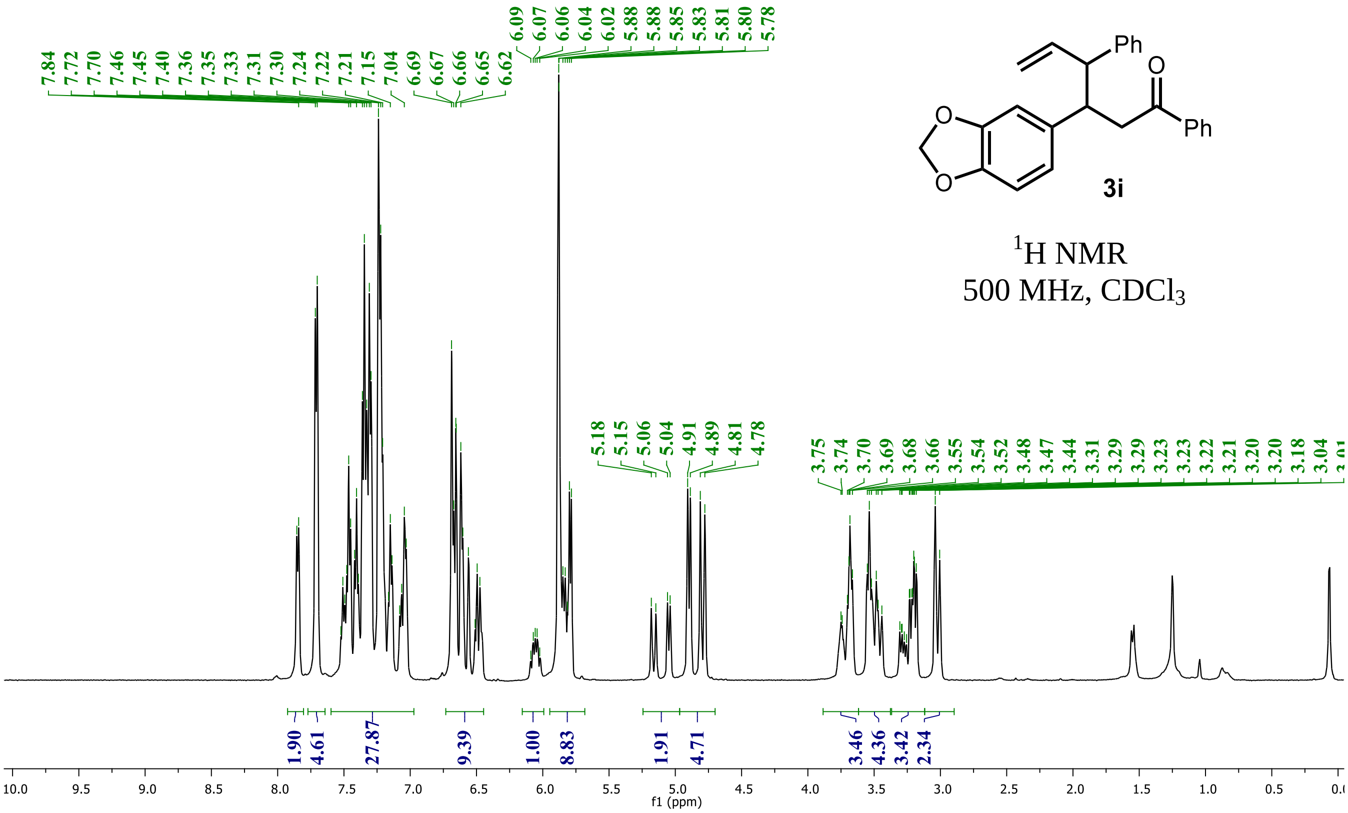

SGS-4-6 C

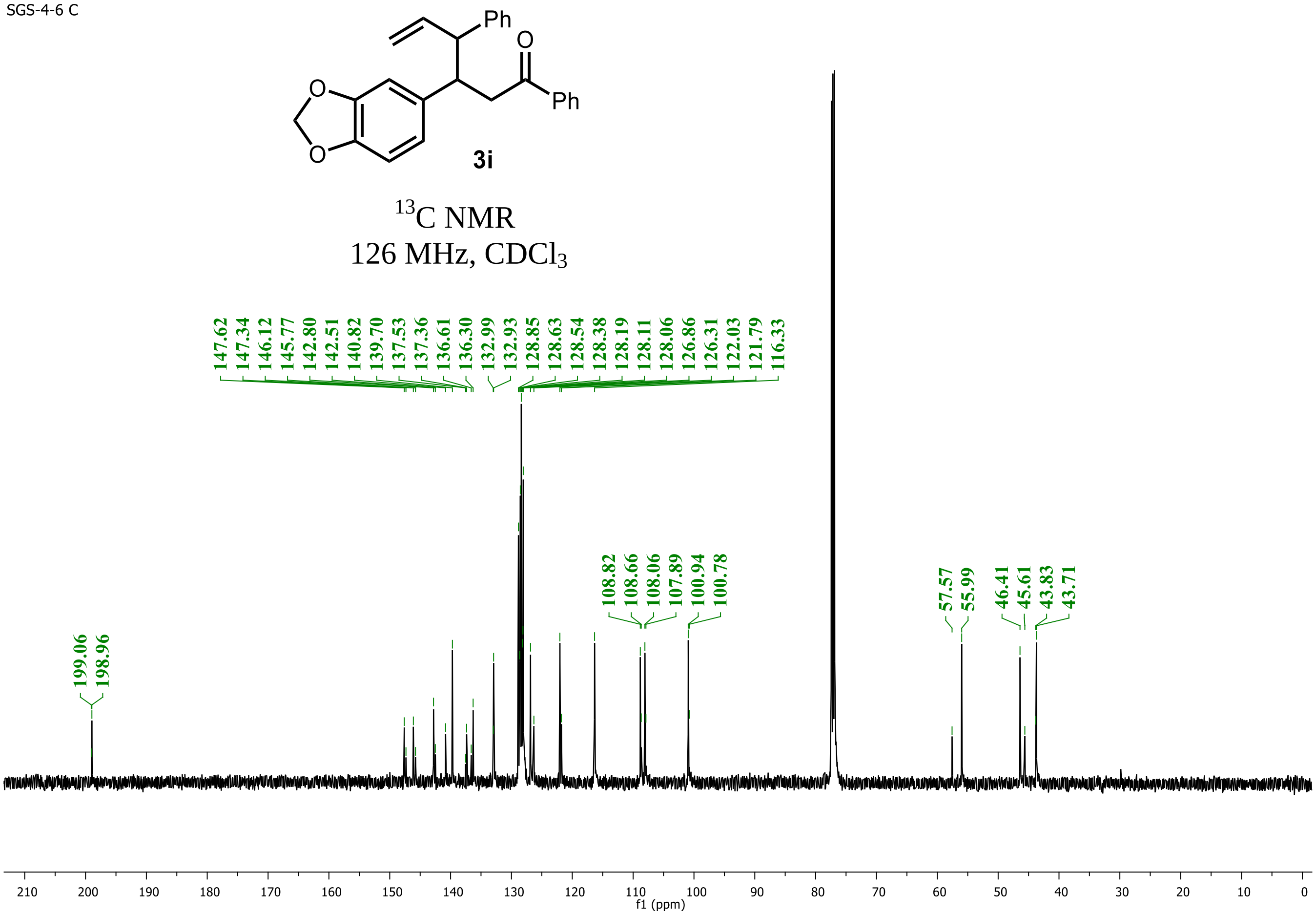

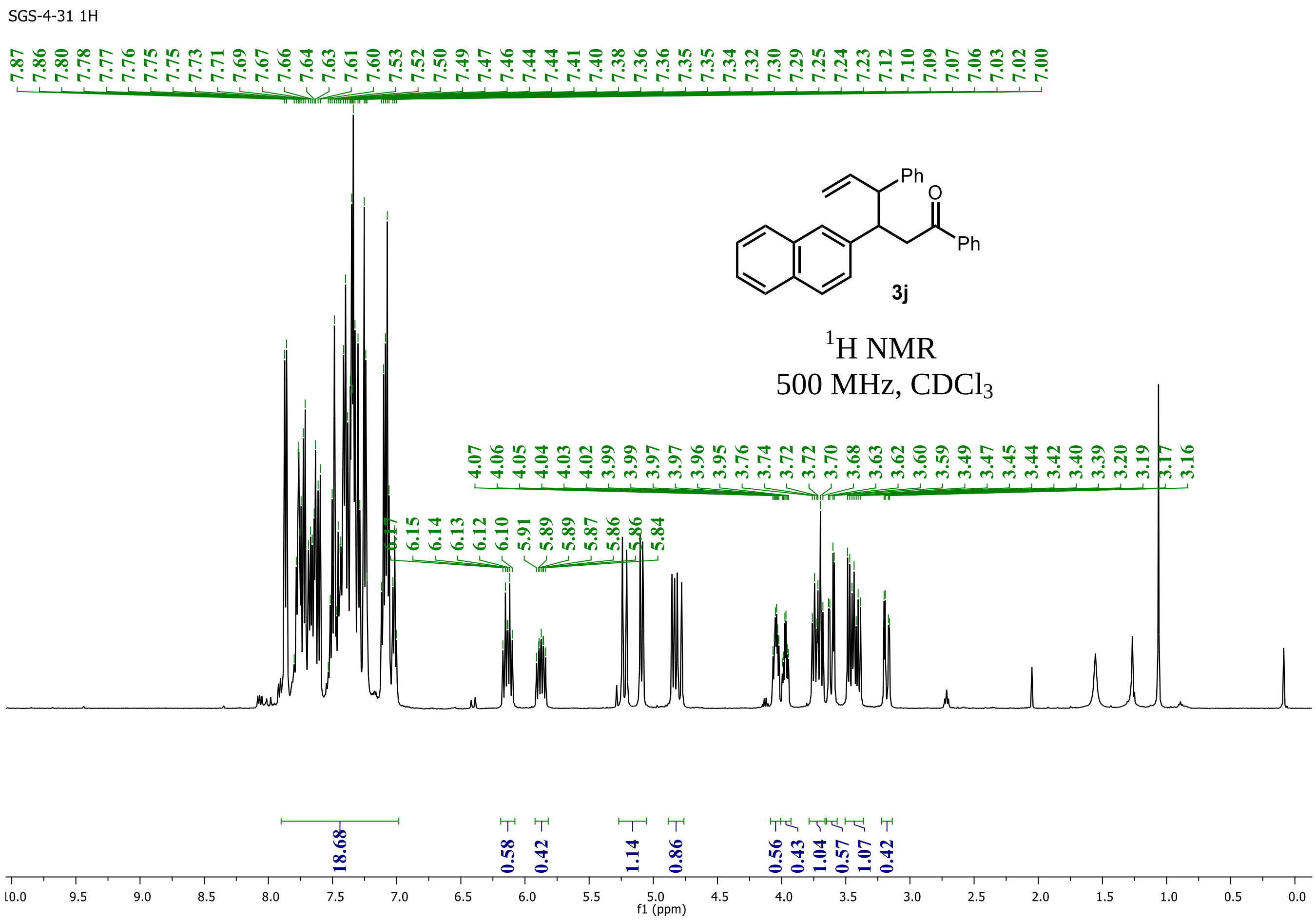
SGS-4-31 1H
3j
1H NMR
500 MHz, CDCl3
f1 (ppm)

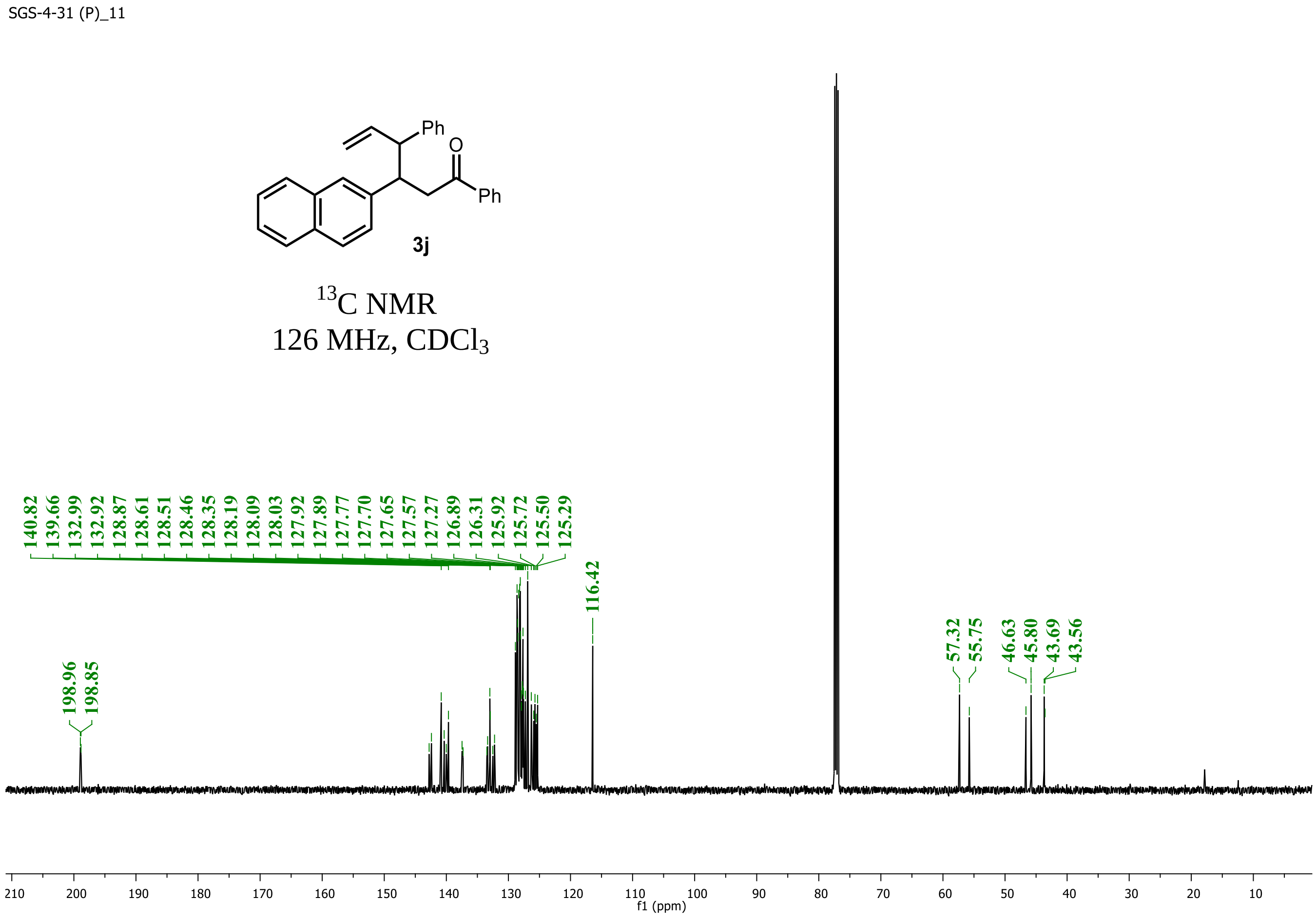
SGS-4-31 (P)_11
3j
13C NMR
126 MHz, CDCl3
f1 (ppm)

SGS-4-21 1H

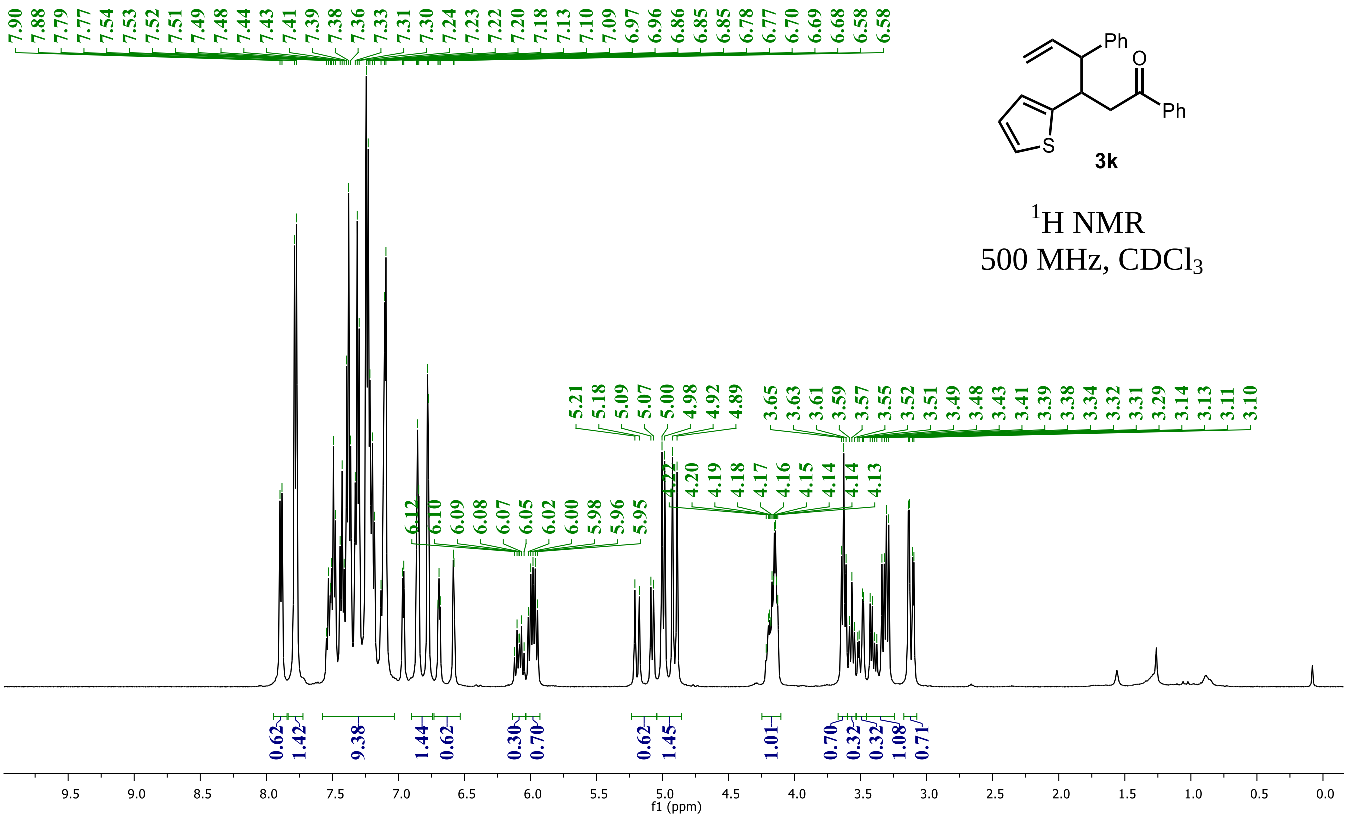

SGS-4-21 C

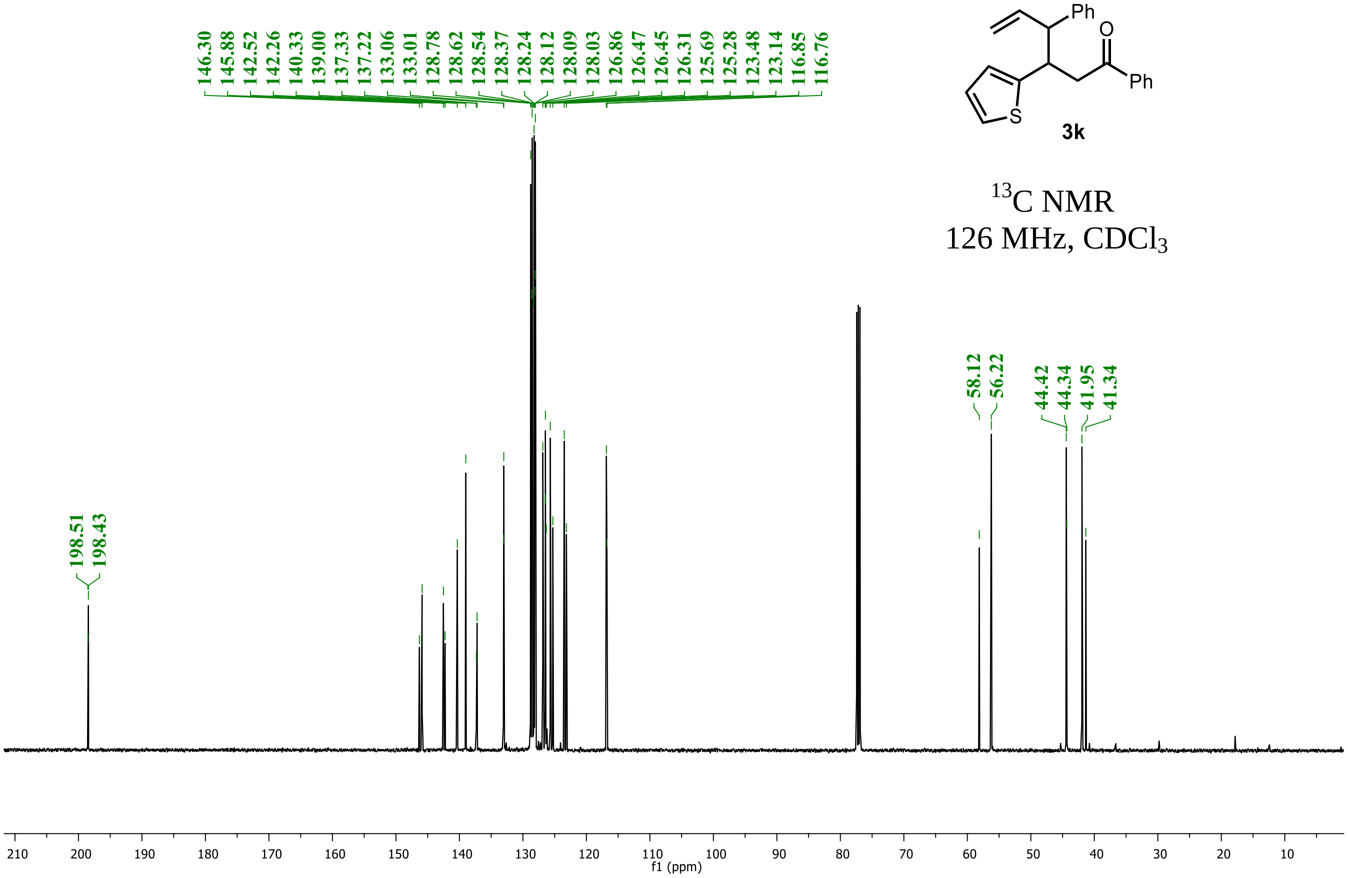

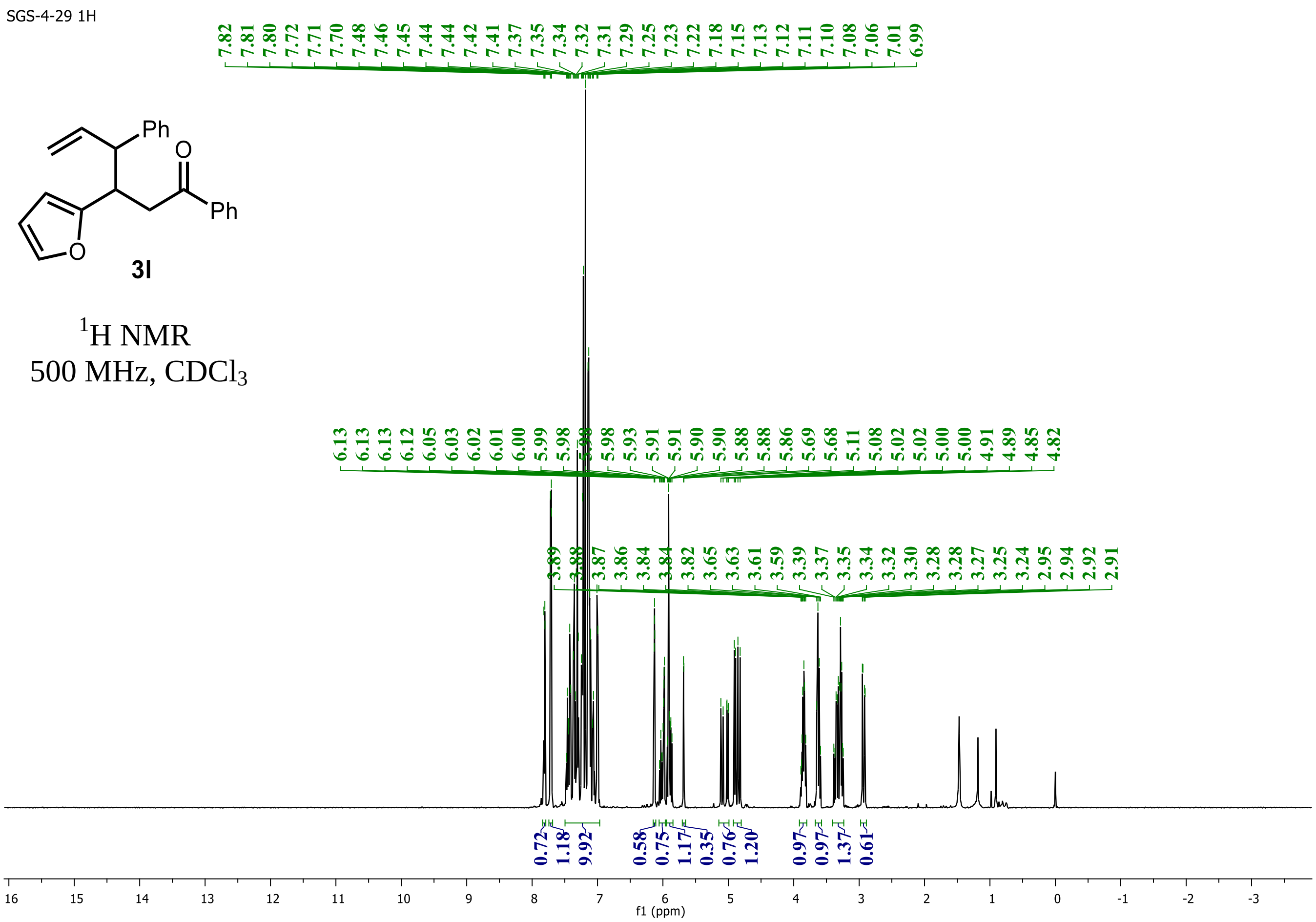
SGS-4-29 1H
Ph
O
Ph
O
3l
^{1}H NMR
500 MHz, CDCl$_3$
7.82 7.81 7.80 7.72 7.71 7.70 7.48 7.46 7.45 7.44 7.44 7.42 7.41 7.37 7.35 7.34 7.32 7.31 7.29 7.25 7.23 7.22 7.18 7.15 7.13 7.12 7.11 7.10 7.08 7.06 7.01 6.99
6.13 6.13 6.13 6.12 6.05 6.03 6.02 6.01 6.00 5.99 5.98 5.98 5.98 5.93 5.91 5.91 5.90 5.90 5.88 5.88 5.86 5.69 5.68 5.11 5.08 5.02 5.02 5.00 5.00 4.91 4.89 4.85 4.82
3.89 3.88 3.87 3.86 3.84 3.84 3.82 3.65 3.63 3.61 3.59 3.39 3.37 3.35 3.34 3.32 3.30 3.28 3.28 3.27 3.25 3.24 2.95 2.94 2.92 2.91
0.72 1.18 9.92 0.58 0.75 1.17 0.35 0.76 1.20 0.97 0.97 1.37 0.61
16 15 14 13 12 11 10 9 8 7 6 5 4 3 2 1 0 -1 -2 -3
f1 (ppm)

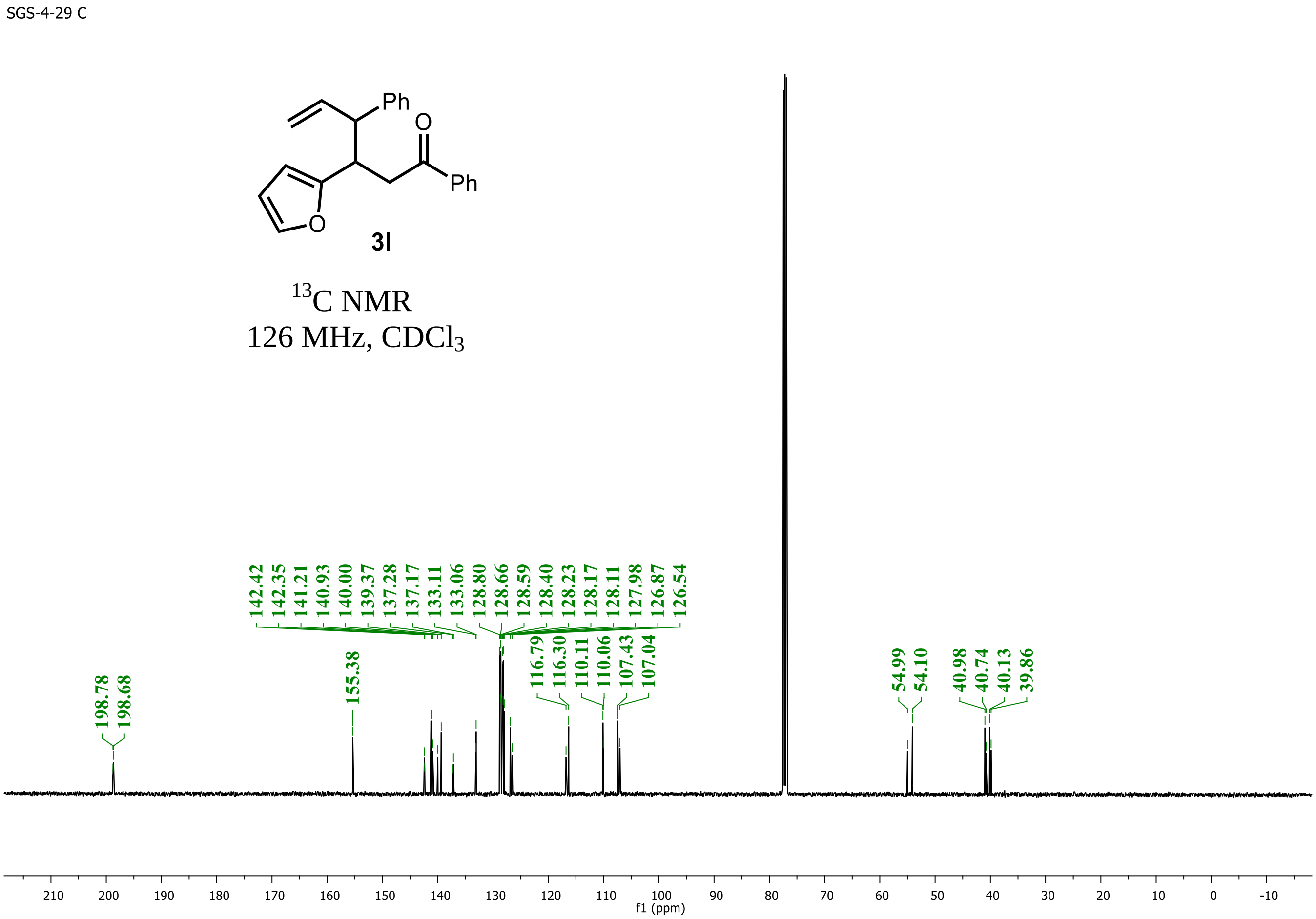
SGS-4-29 C
Ph
O
Ph
O
3l
^{13}C NMR
126 MHz, CDCl$_3$
198.78 198.68
155.38
142.42 142.35 141.21 140.93 140.00 139.37 137.28 137.17 133.11 133.06 128.80 128.66 128.59 128.40 128.23 128.17 128.11 127.98 126.87 126.54
116.79 116.30 110.11 110.06 107.43 107.04
54.99 54.10
40.98 40.74 40.13 39.86
210 200 190 180 170 160 150 140 130 120 110 100 90 80 70 60 50 40 30 20 10 0 -10
f1 (ppm)

SGS-4-51 H

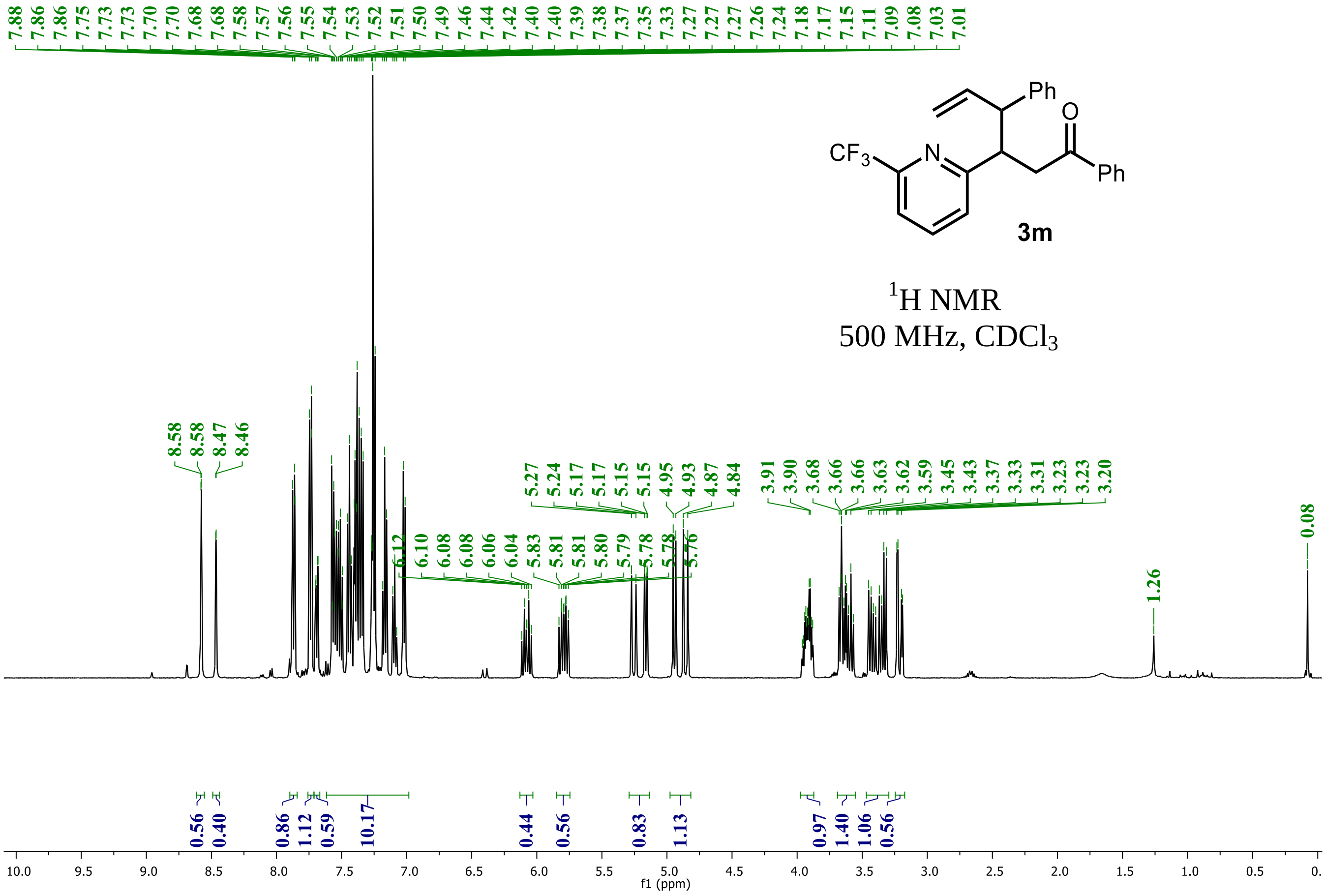

SGS-4-51 C

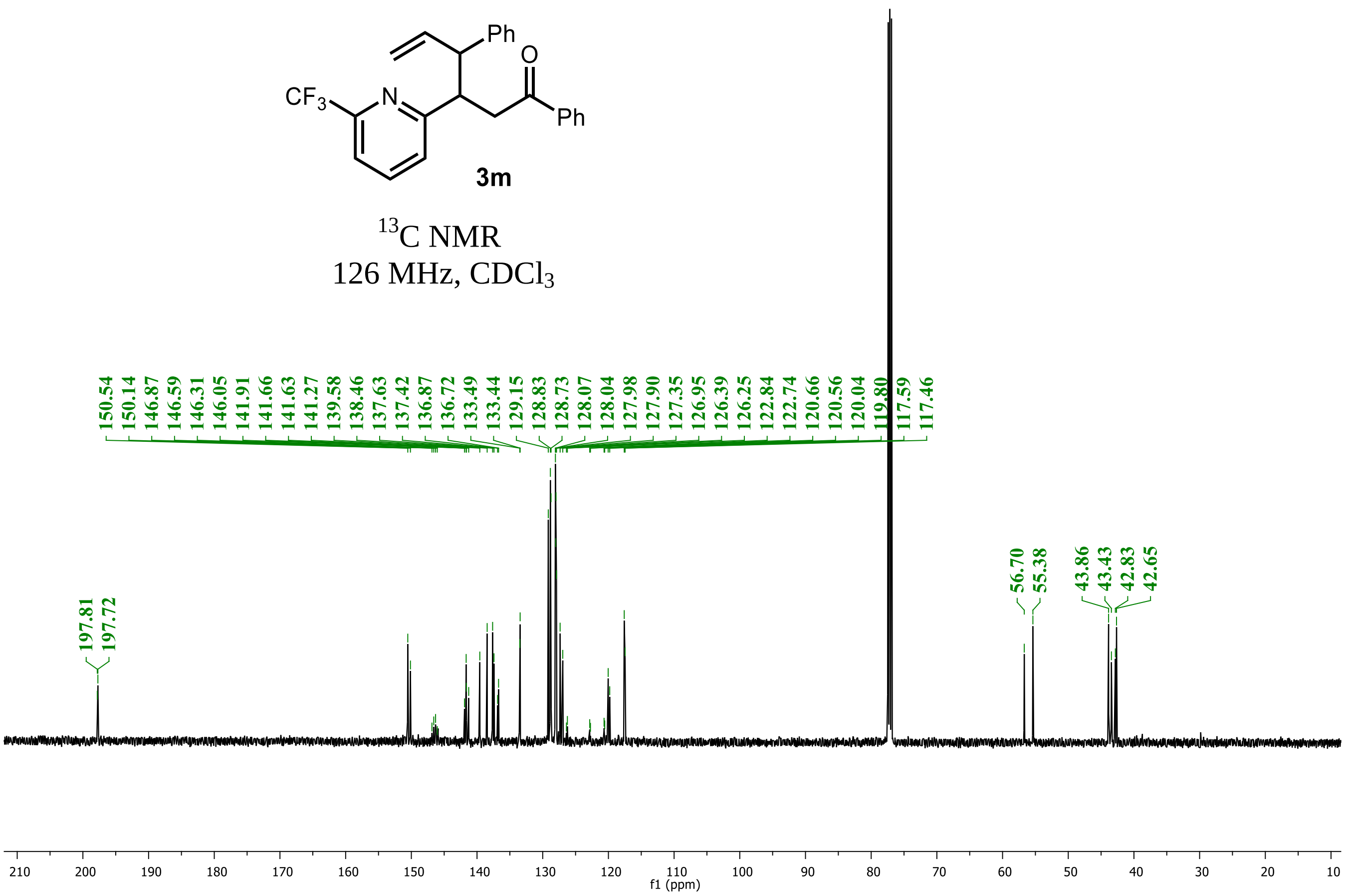

SGS-4-51 F

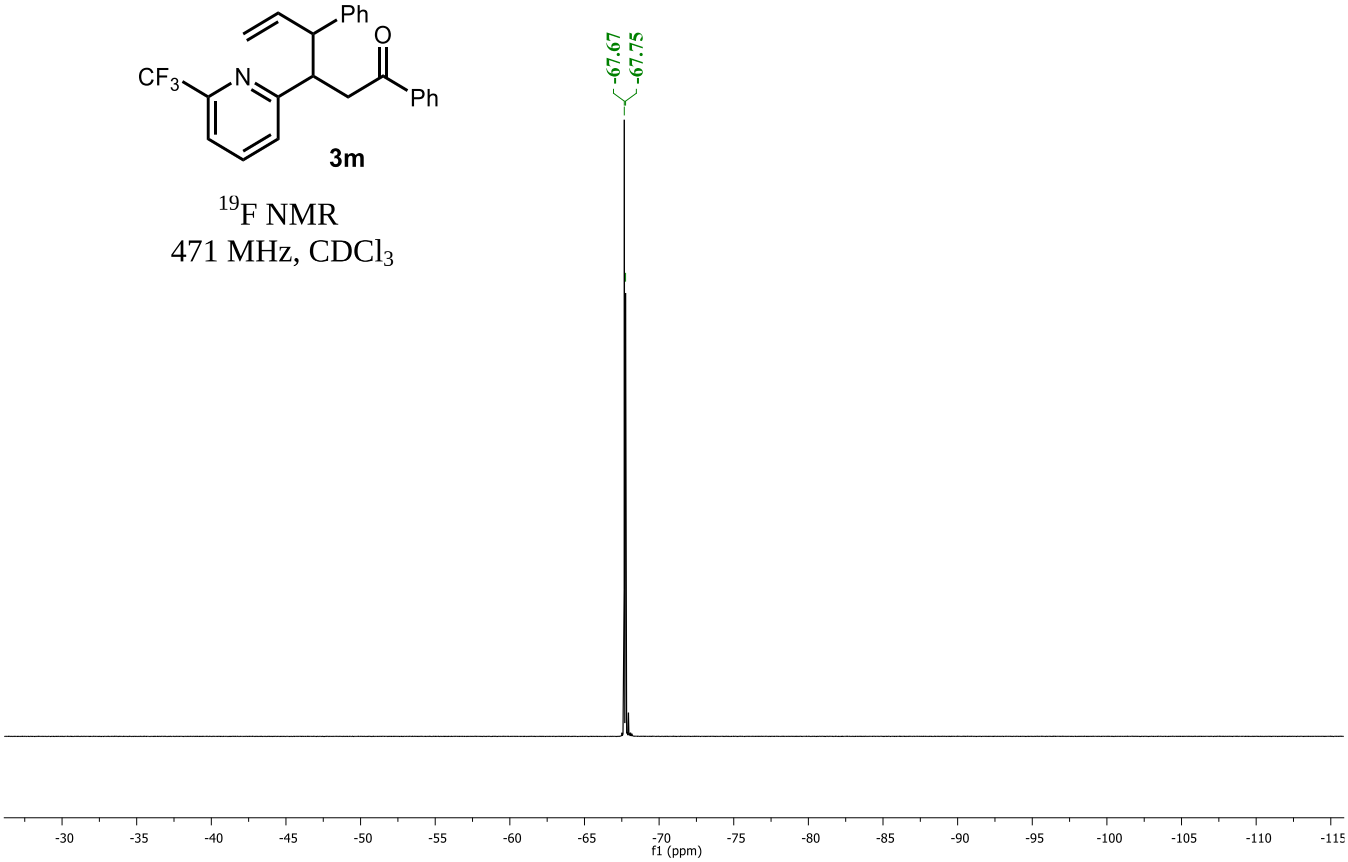

SGS-4-23 H

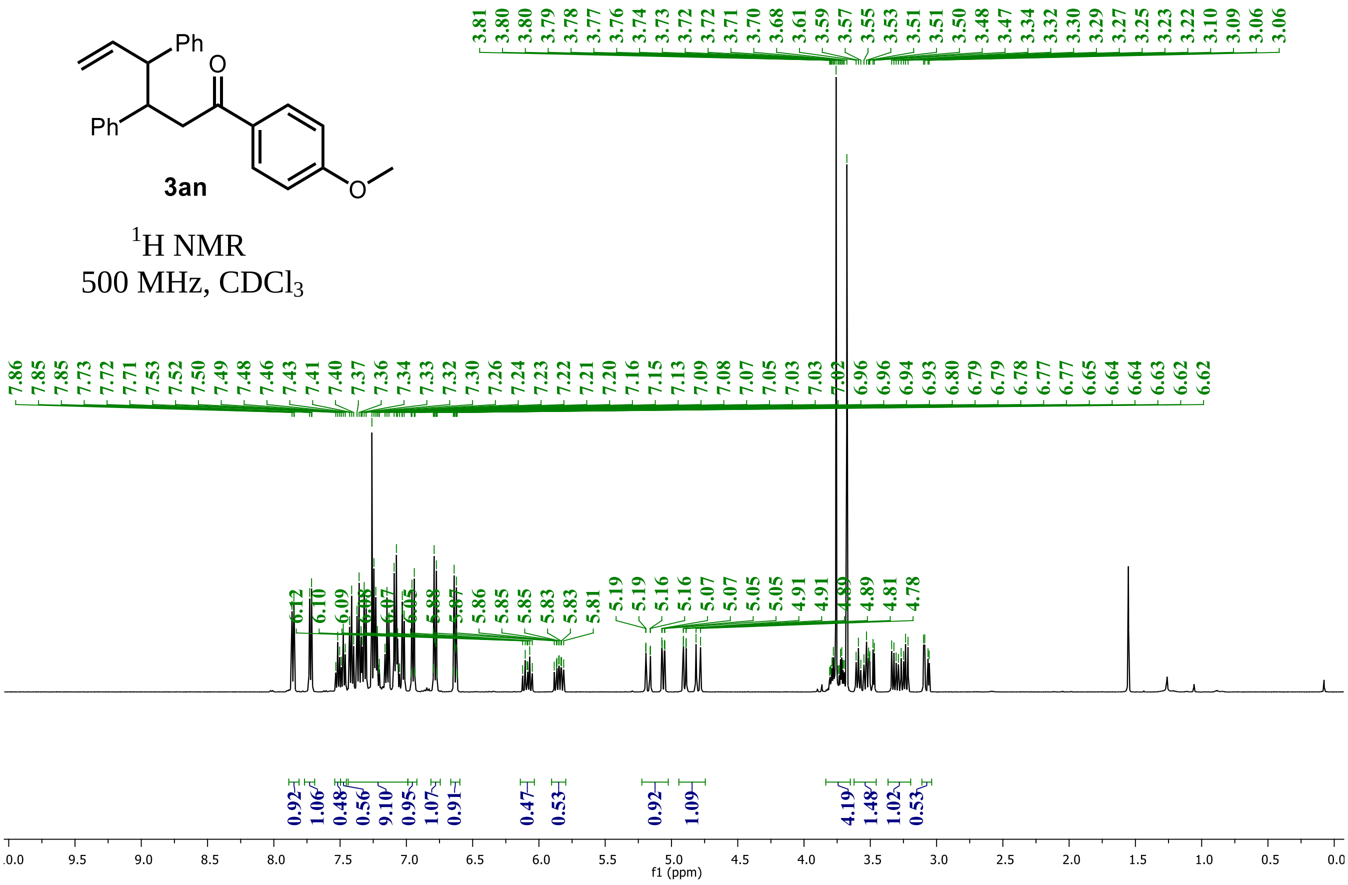

SGS-4-23-2 C

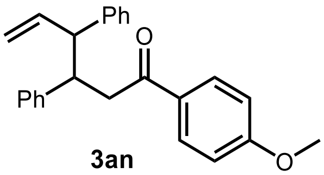

3an

^{13}C NMR
126 MHz, $CDCl_3$

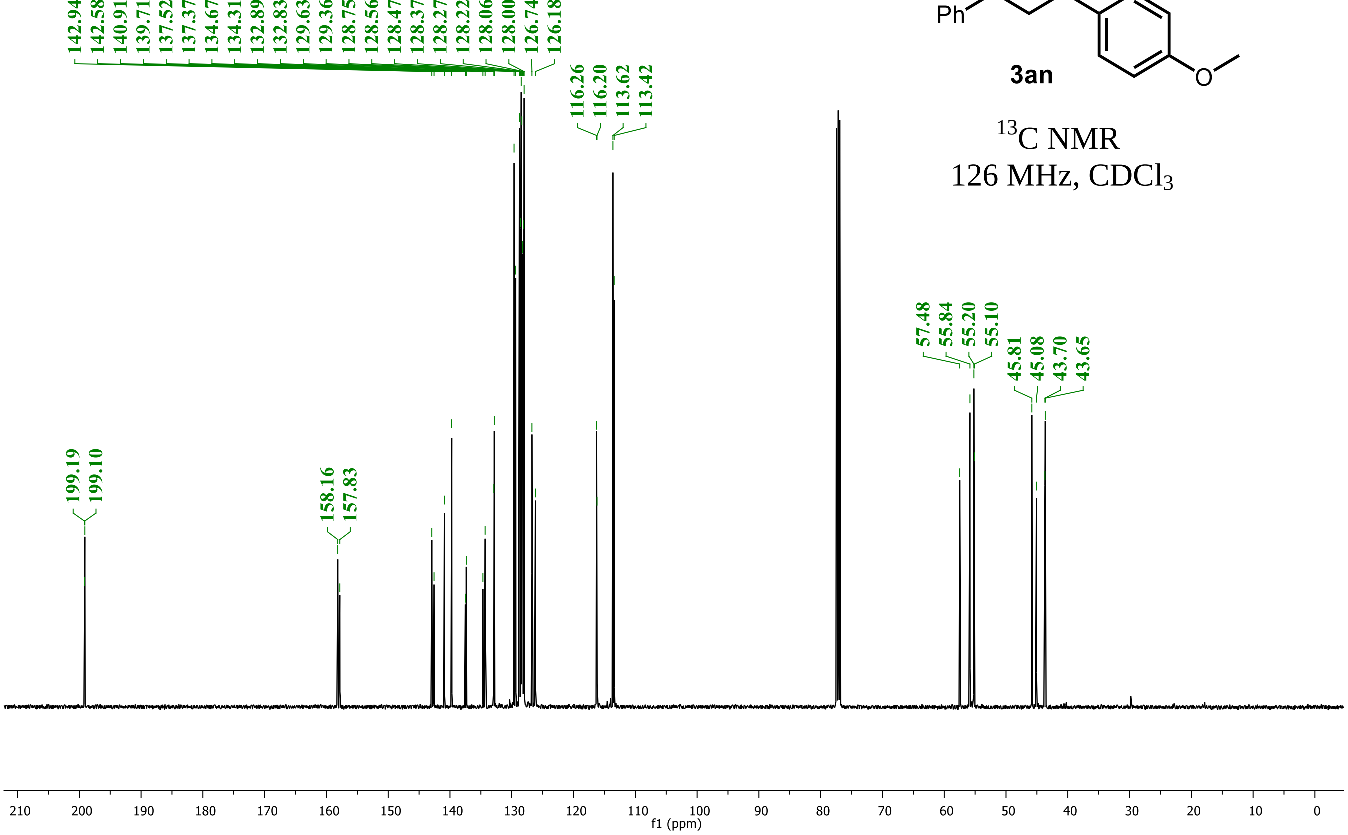

SGS-4-4 H

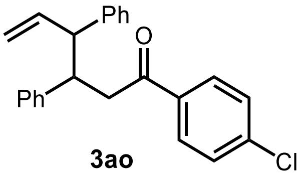

3ao

^{1}H NMR
500 MHz, $CDCl_3$

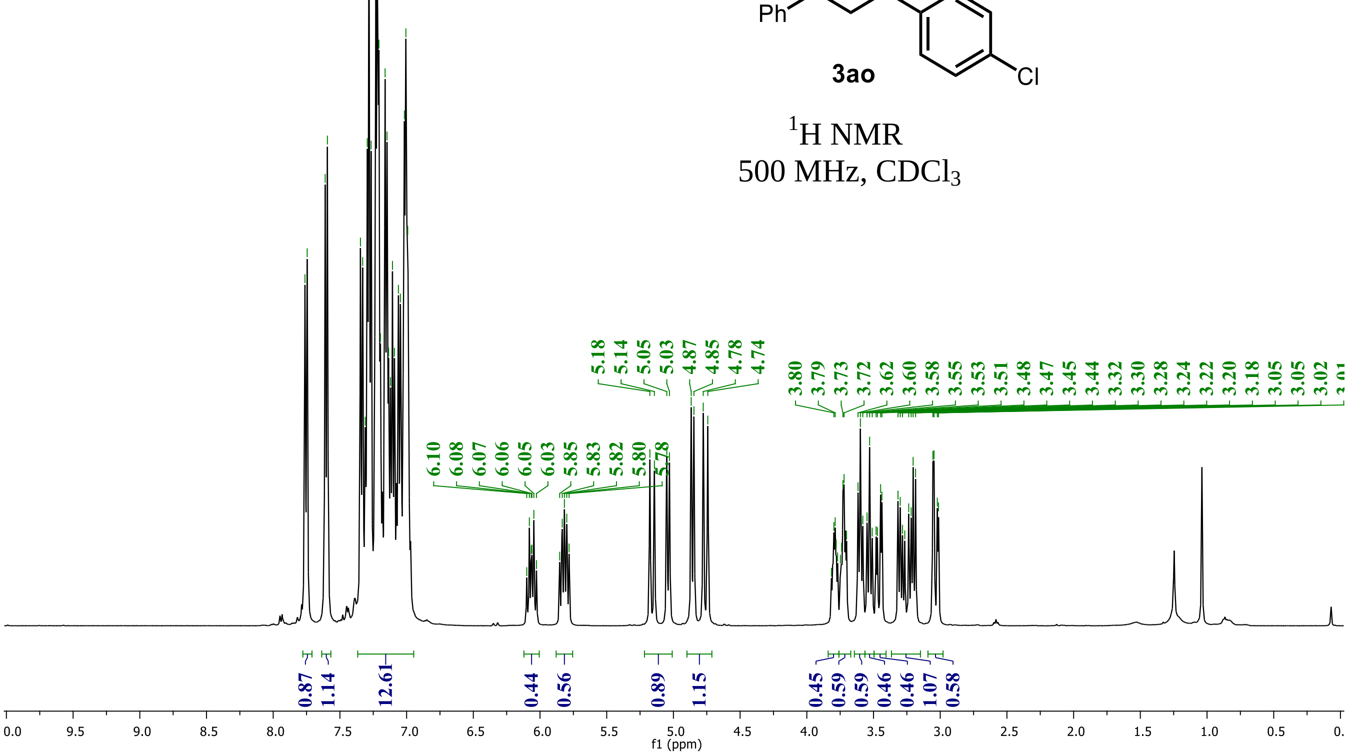

SGS-4-4 C

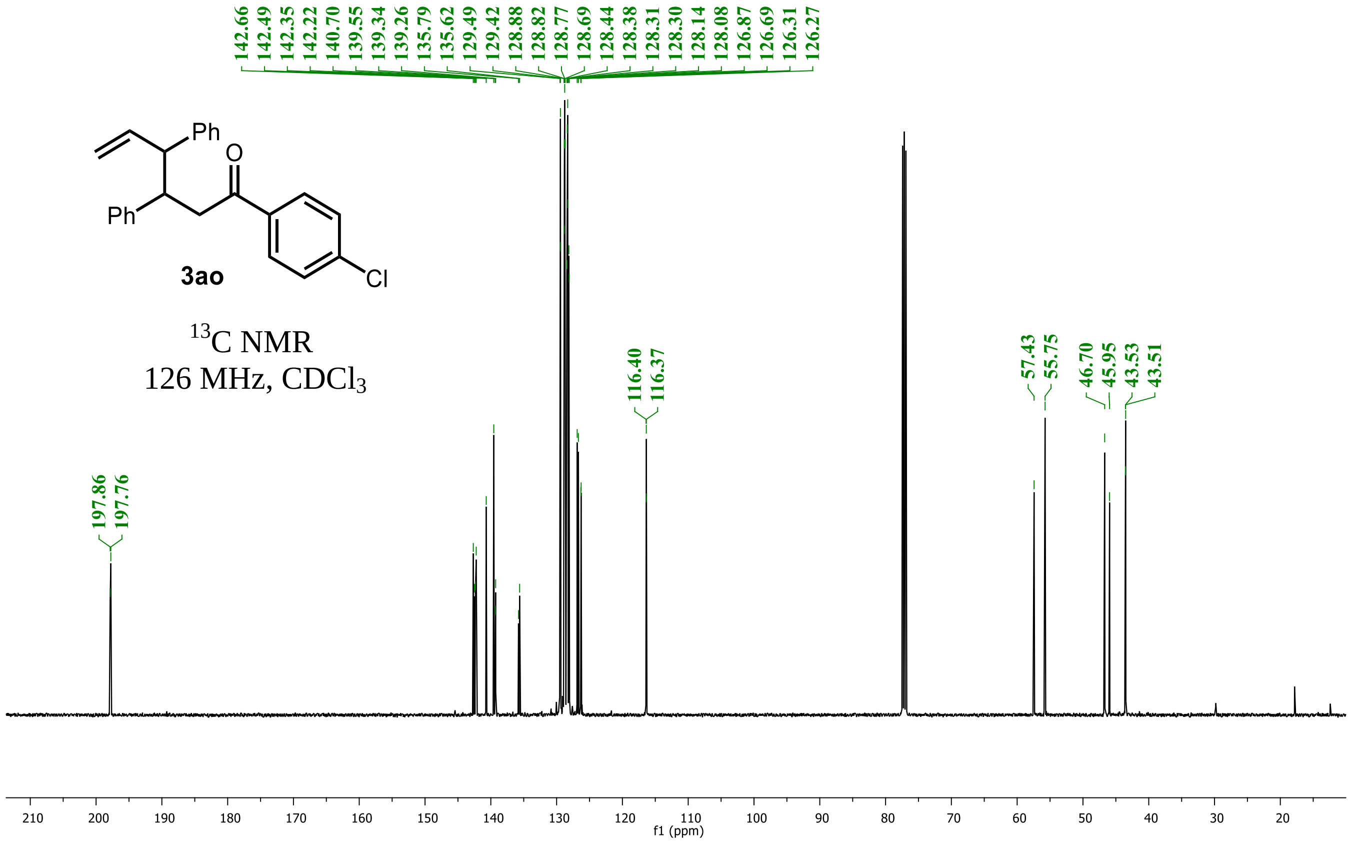

SGS-4-5 H

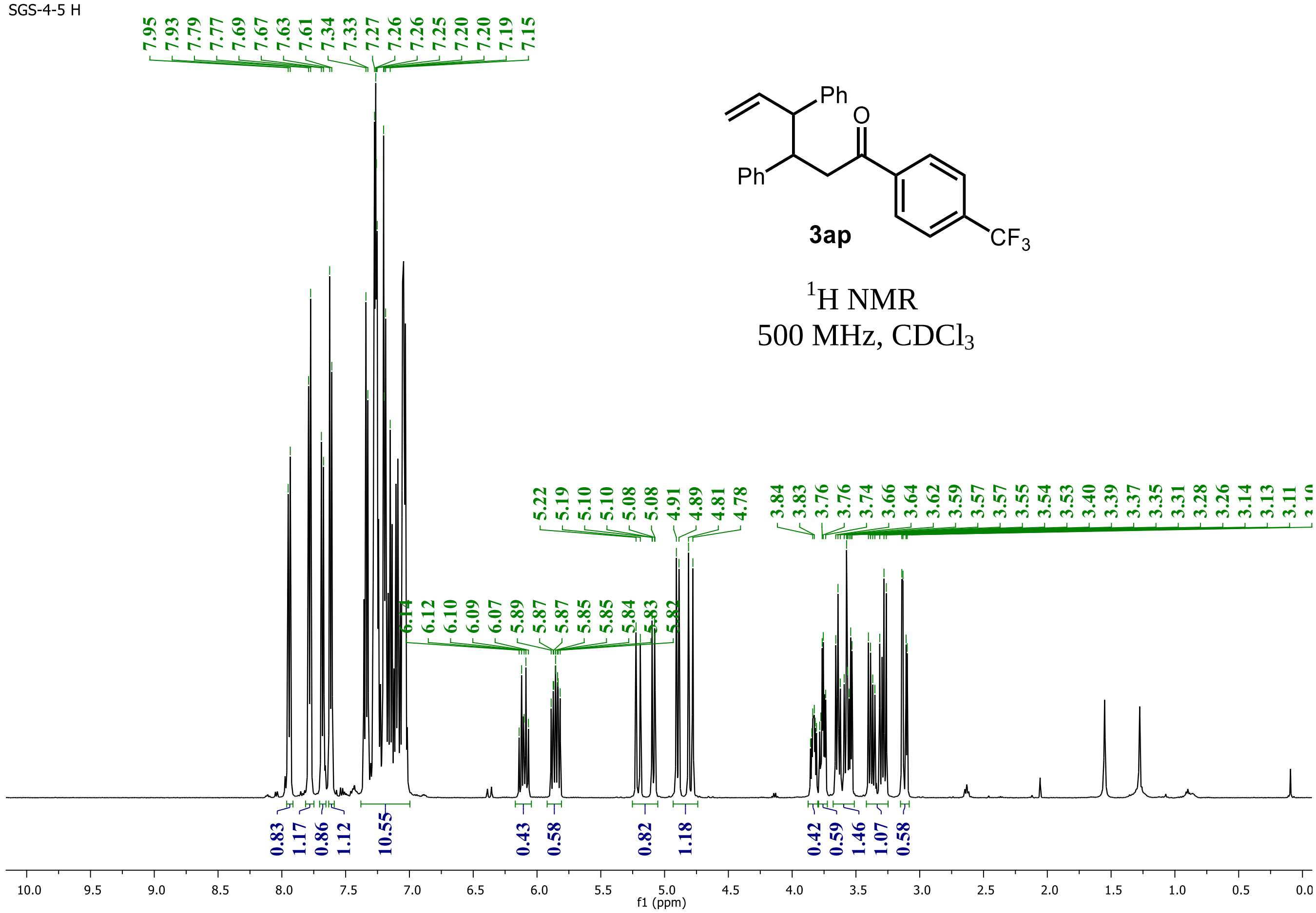

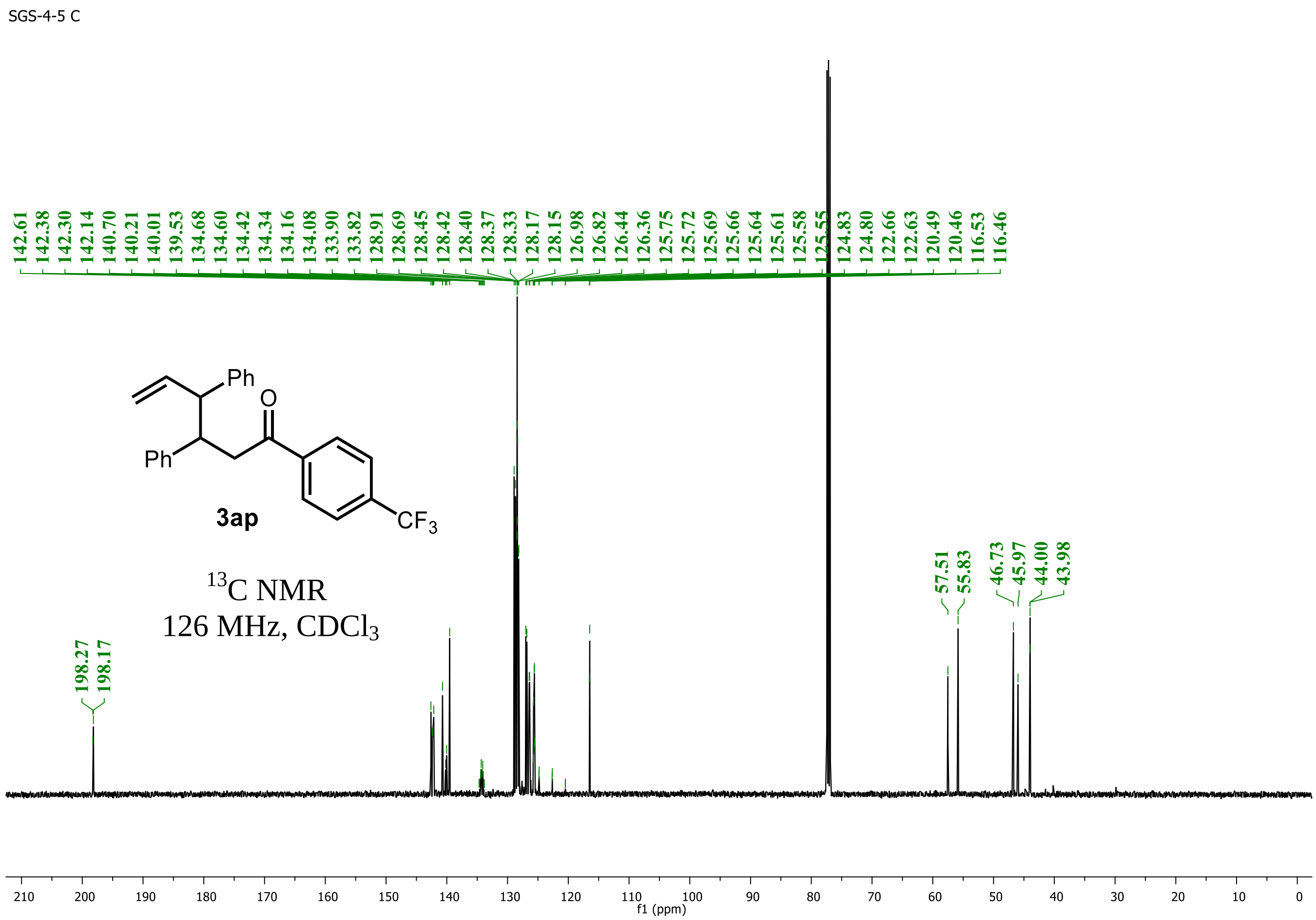
SGS-4-5 C
142.61
142.38
142.30
142.14
140.70
140.21
140.01
139.53
134.68
134.60
134.42
134.34
134.16
134.08
133.90
133.82
128.91
128.69
128.45
128.42
128.40
128.37
128.33
128.17
128.15
126.98
126.82
126.44
126.36
125.75
125.72
125.69
125.66
125.64
125.61
125.58
125.55
124.83
124.80
122.66
122.63
120.49
120.46
116.53
116.46
Ph
O
Ph
CF3
3ap
13C NMR
126 MHz, CDCl3
198.27
198.17
57.51
55.83
46.73
45.97
44.00
43.98
f1 (ppm)

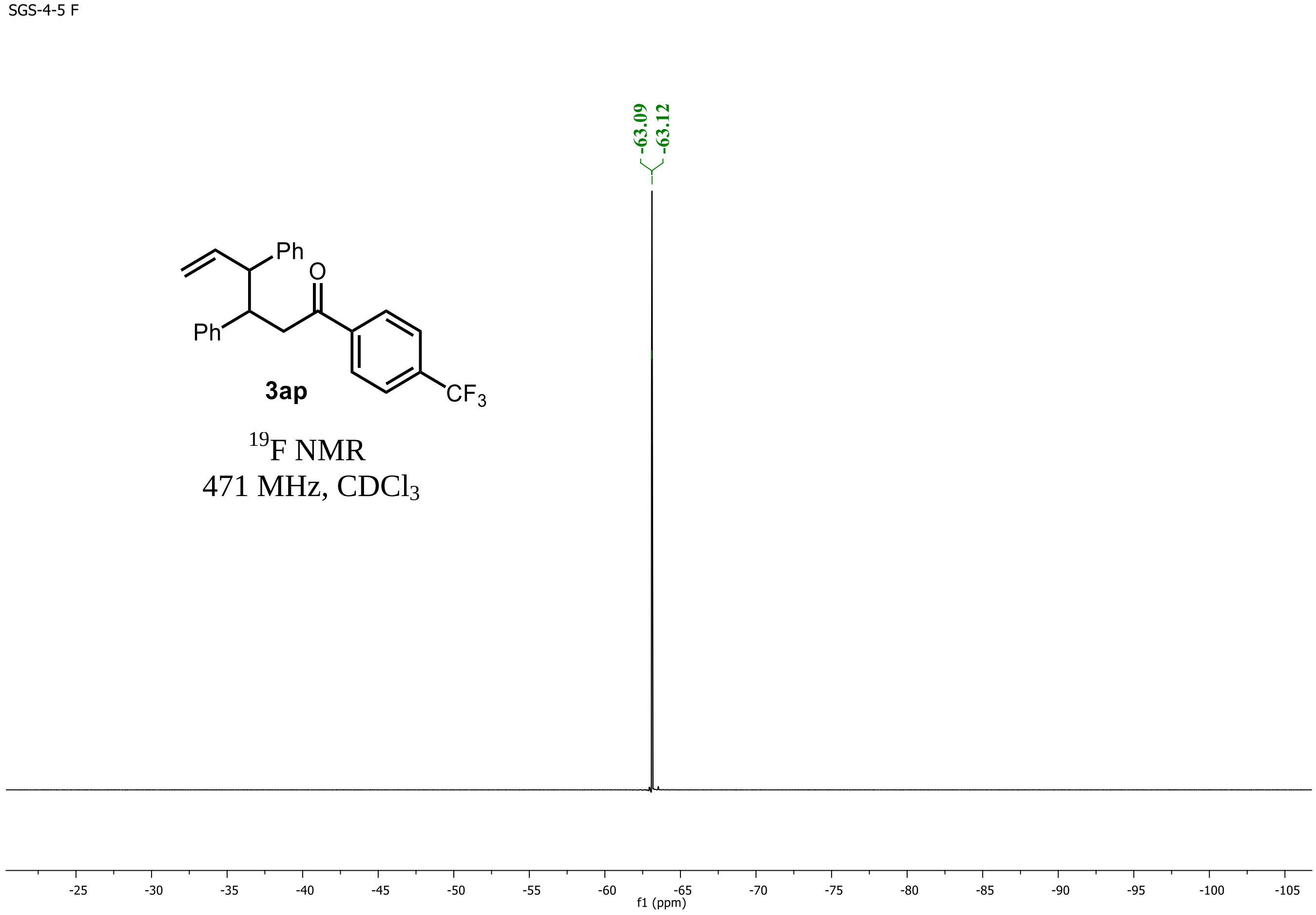
SGS-4-5 F
-63.09
-63.12
Ph
O
Ph
CF3
3ap
19F NMR
471 MHz, CDCl3
f1 (ppm)

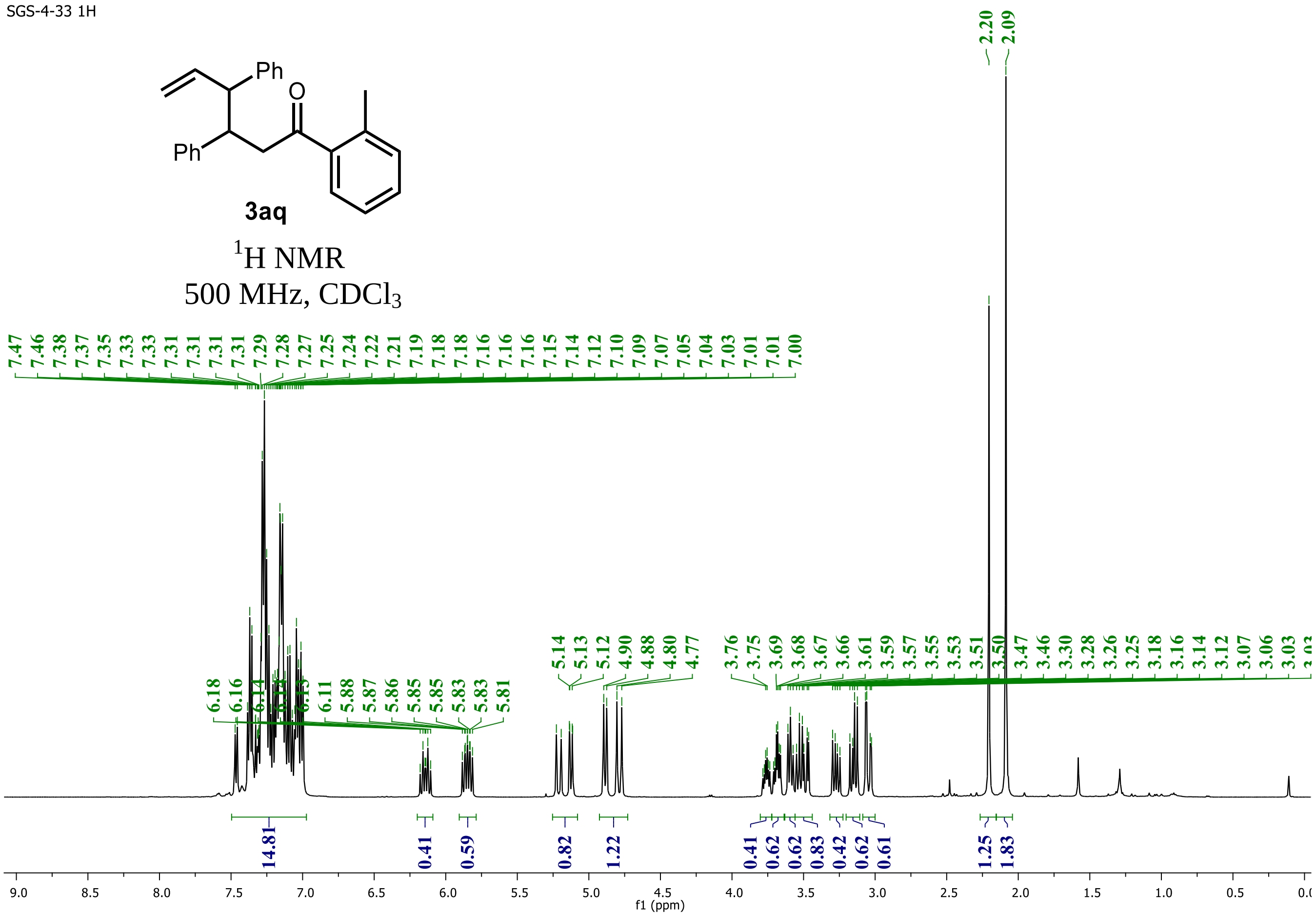
SGS-4-33 1H
Ph
O
Ph
3aq
1H NMR
500 MHz, CDCl3
7.47 7.46 7.38 7.37 7.35 7.33 7.33 7.31 7.31 7.31 7.31 7.29 7.28 7.27 7.25 7.24 7.22 7.21 7.19 7.18 7.18 7.16 7.16 7.16 7.15 7.14 7.12 7.10 7.09 7.07 7.05 7.04 7.03 7.01 7.01 7.00
6.18 6.16 6.14 6.11 5.88 5.87 5.86 5.85 5.85 5.83 5.83 5.81
5.14 5.13 5.12 4.90 4.88 4.80 4.77
3.76 3.75 3.69 3.68 3.67 3.66 3.61 3.59 3.57 3.55 3.53 3.51 3.50 3.47 3.46 3.30 3.28 3.26 3.25 3.18 3.16 3.14 3.12 3.07 3.06 3.03
2.20 2.09
14.81 0.41 0.59 0.82 1.22 0.41 0.62 0.62 0.83 0.42 0.62 0.61 1.25 1.83
9.0 8.5 8.0 7.5 7.0 6.5 6.0 5.5 5.0 4.5 4.0 3.5 3.0 2.5 2.0 1.5 1.0 0.5
f1 (ppm)

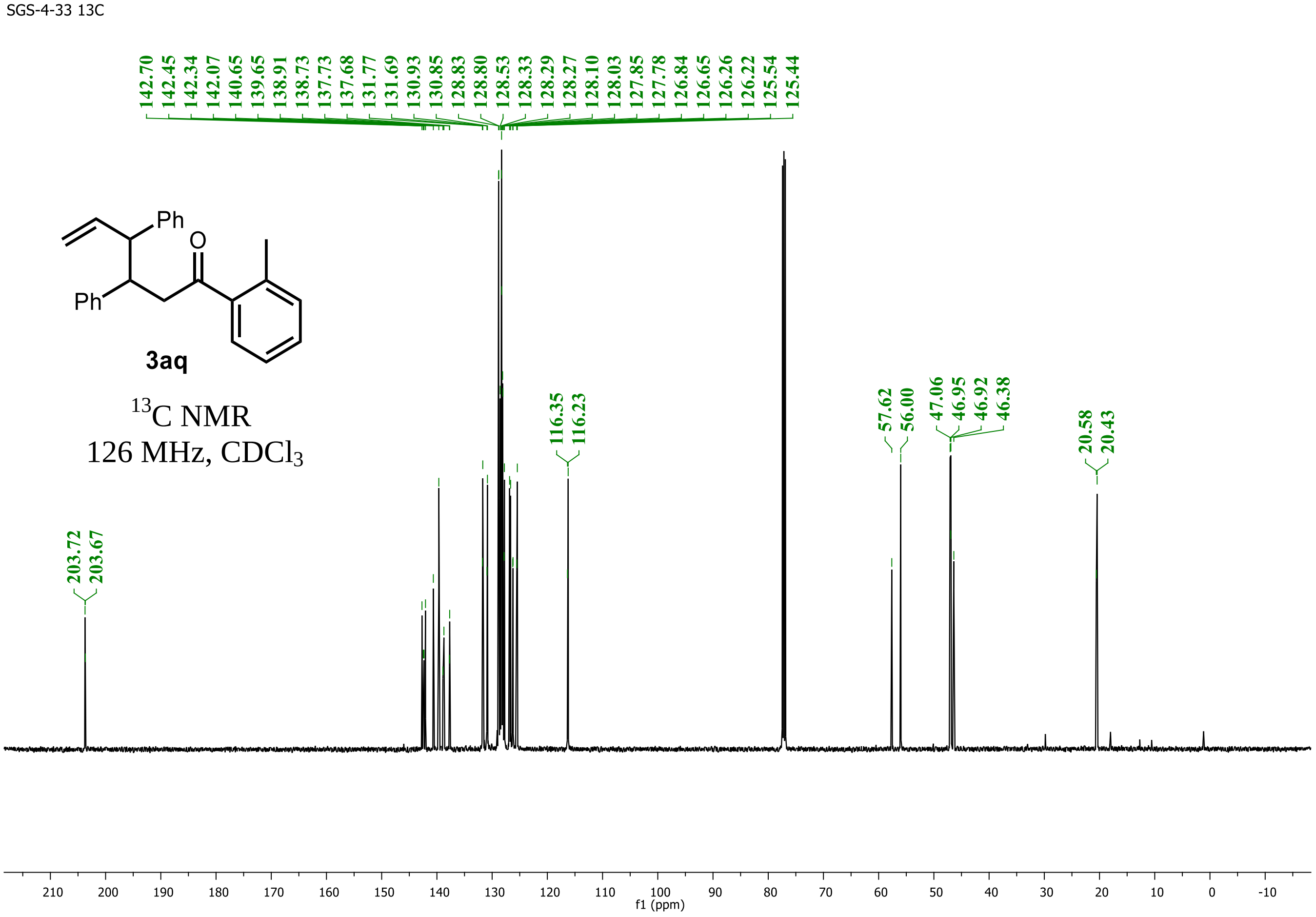
SGS-4-33 13C
142.70 142.45 142.34 142.07 140.65 139.65 138.91 138.73 137.73 137.68 131.77 131.69 130.93 130.85 128.83 128.80 128.53 128.33 128.29 128.27 128.10 128.03 127.85 127.78 126.84 126.65 126.26 126.22 125.54 125.44
Ph
O
Ph
3aq
13C NMR
126 MHz, CDCl3
203.72 203.67
116.35 116.23
57.62 56.00
47.06 46.95 46.92 46.38
20.58 20.43
210 200 190 180 170 160 150 140 130 120 110 100 90 80 70 60 50 40 30 20 10 0 -10
f1 (ppm)

SGS-4-34 1H

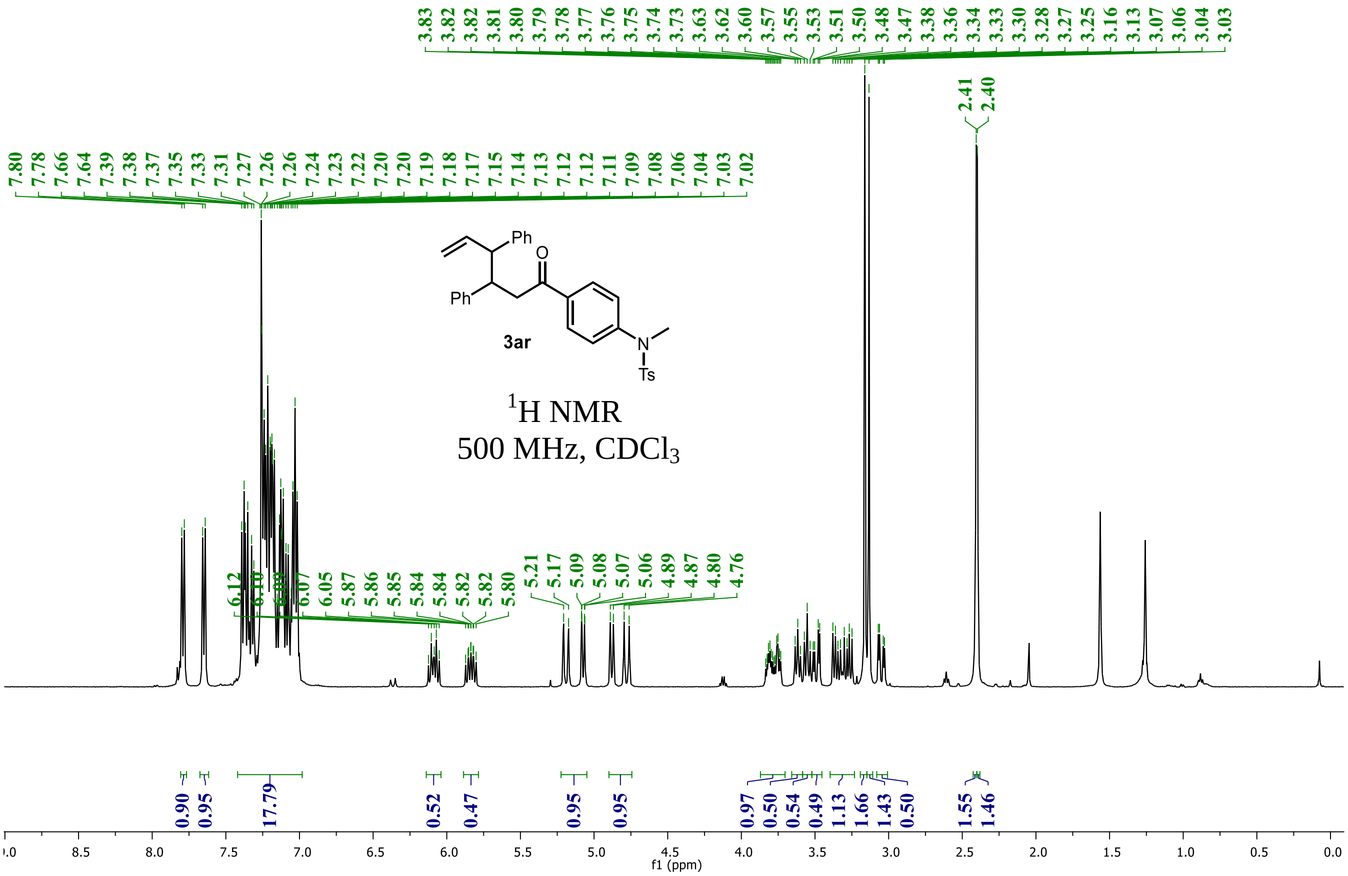

SGS-4-34 13C

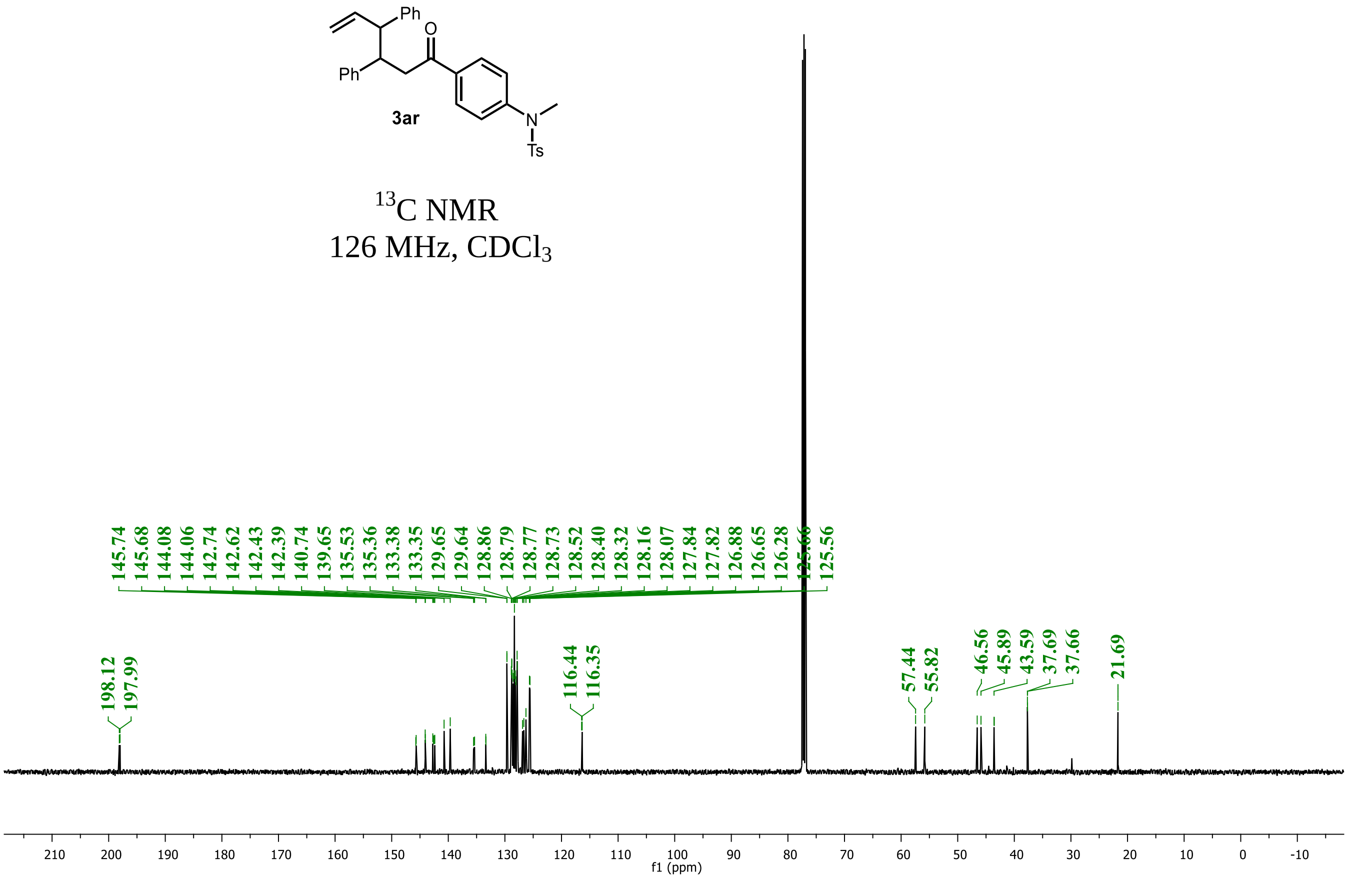

SGS-4-52 H

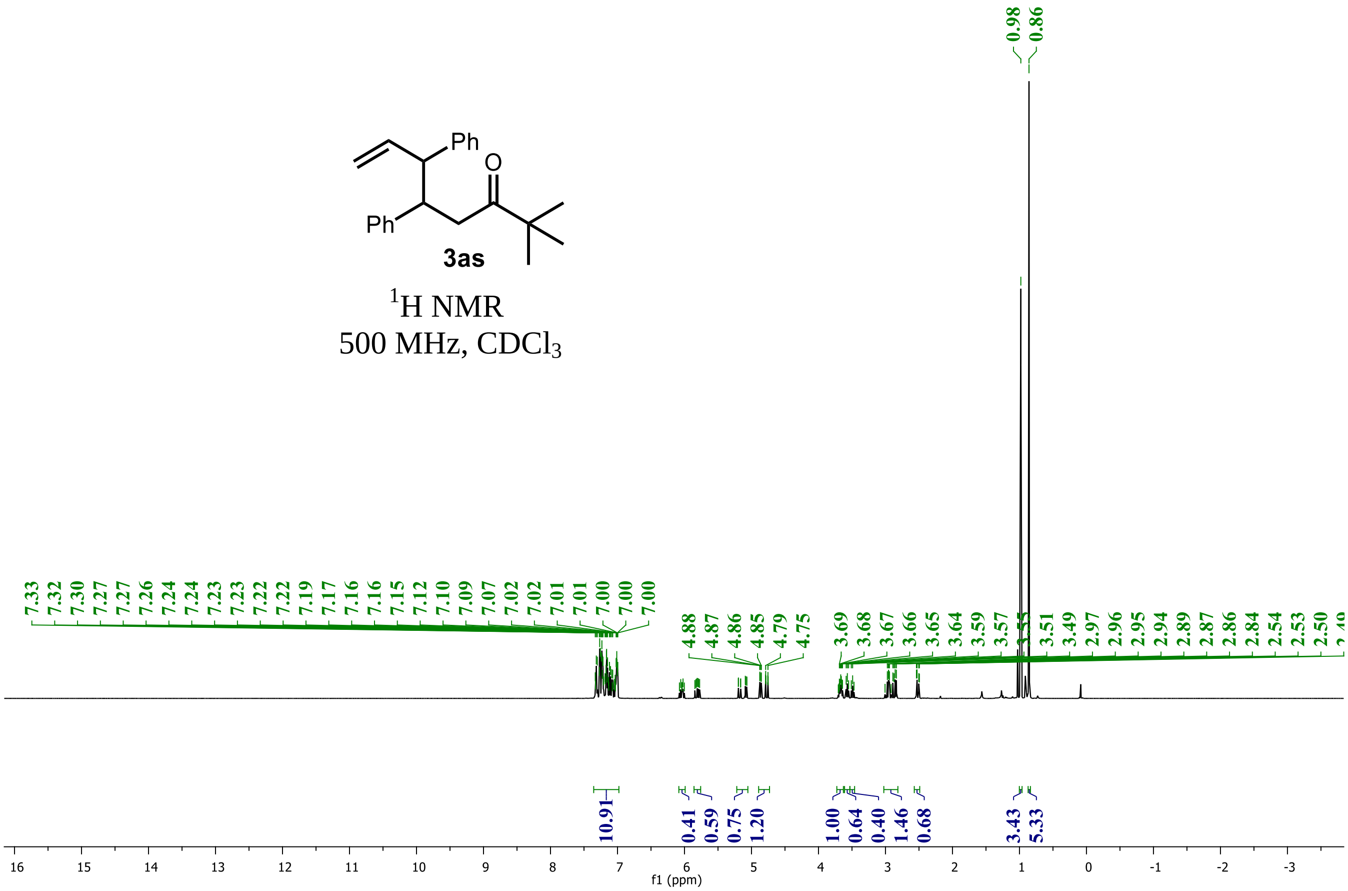

SGS-4-52 C

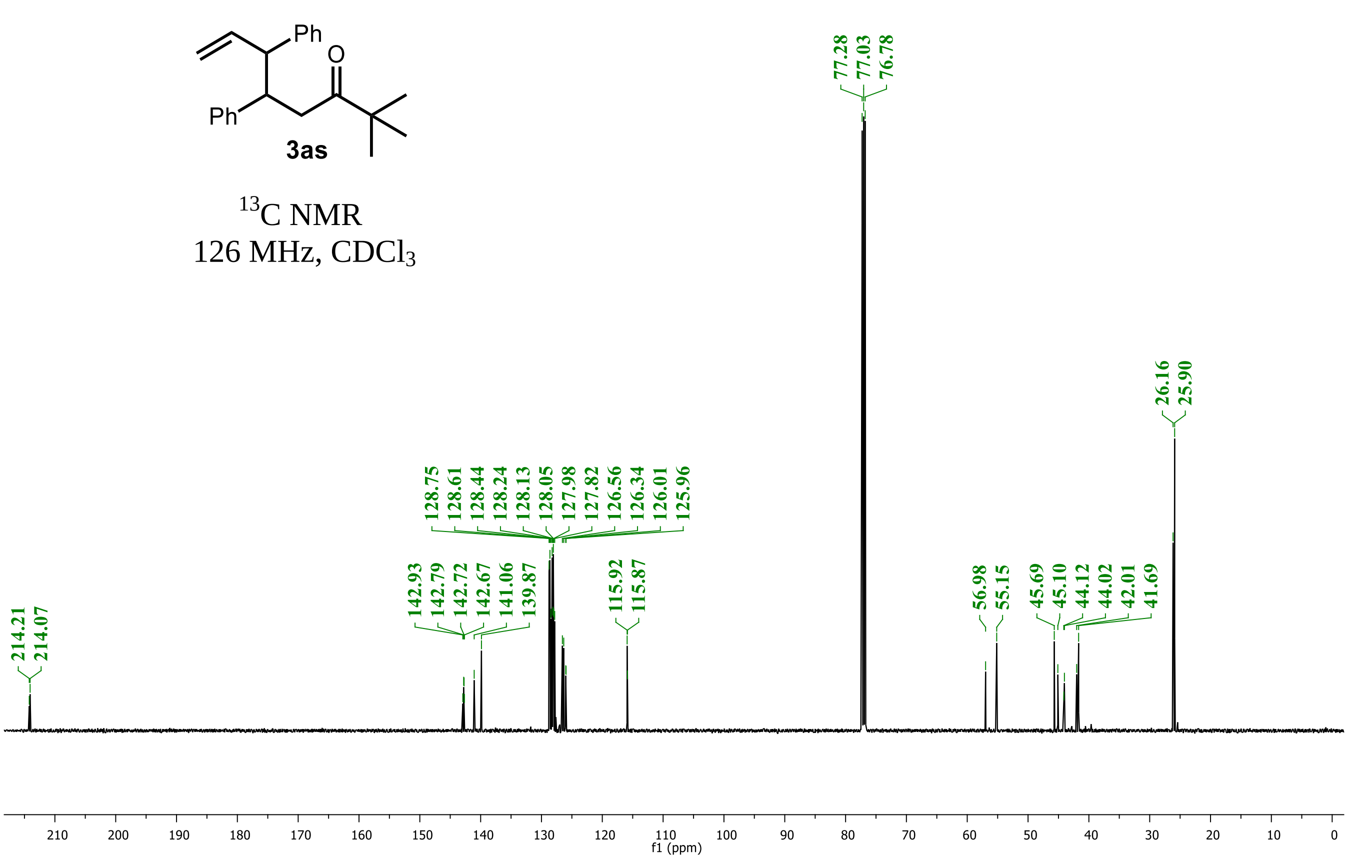

SGS-4-55 H

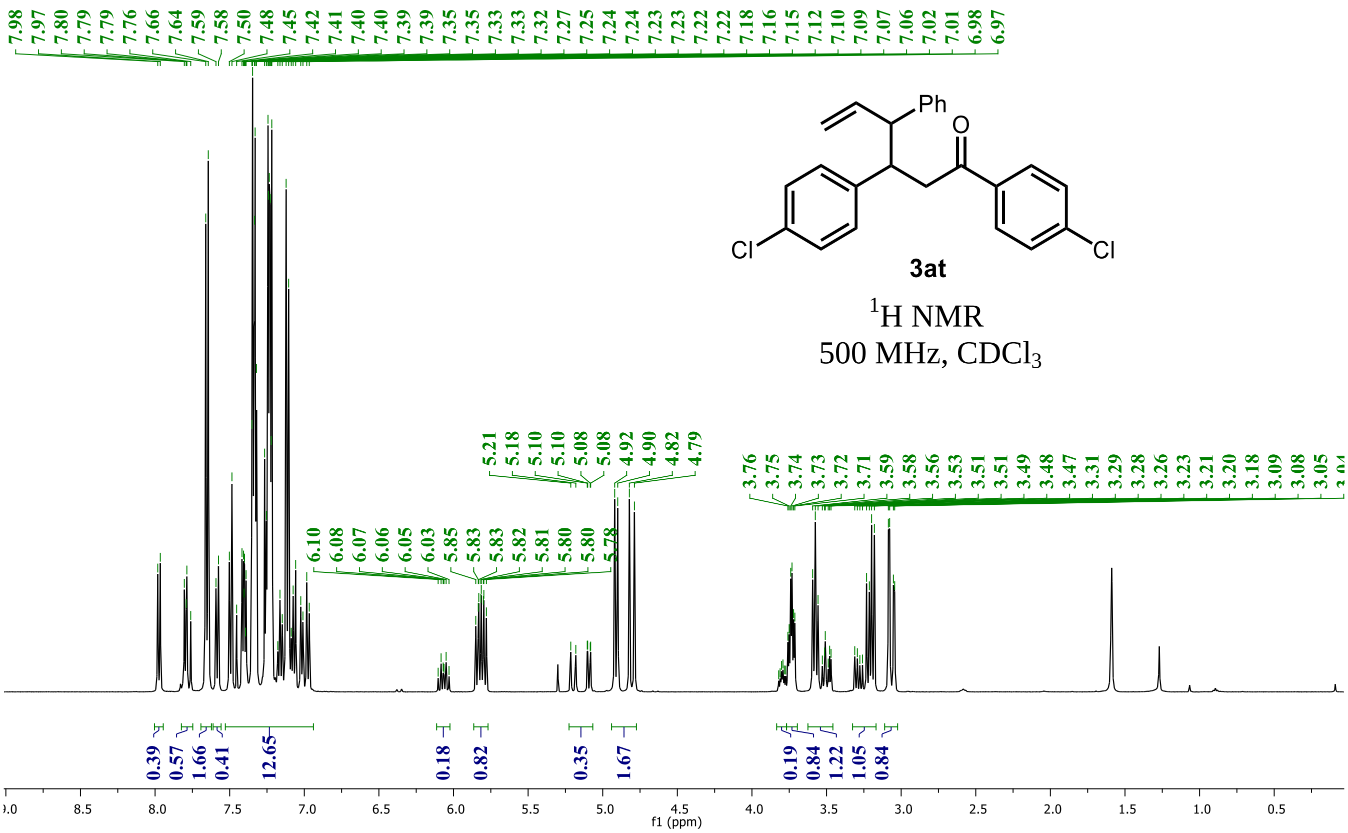

SGS-4-55 C

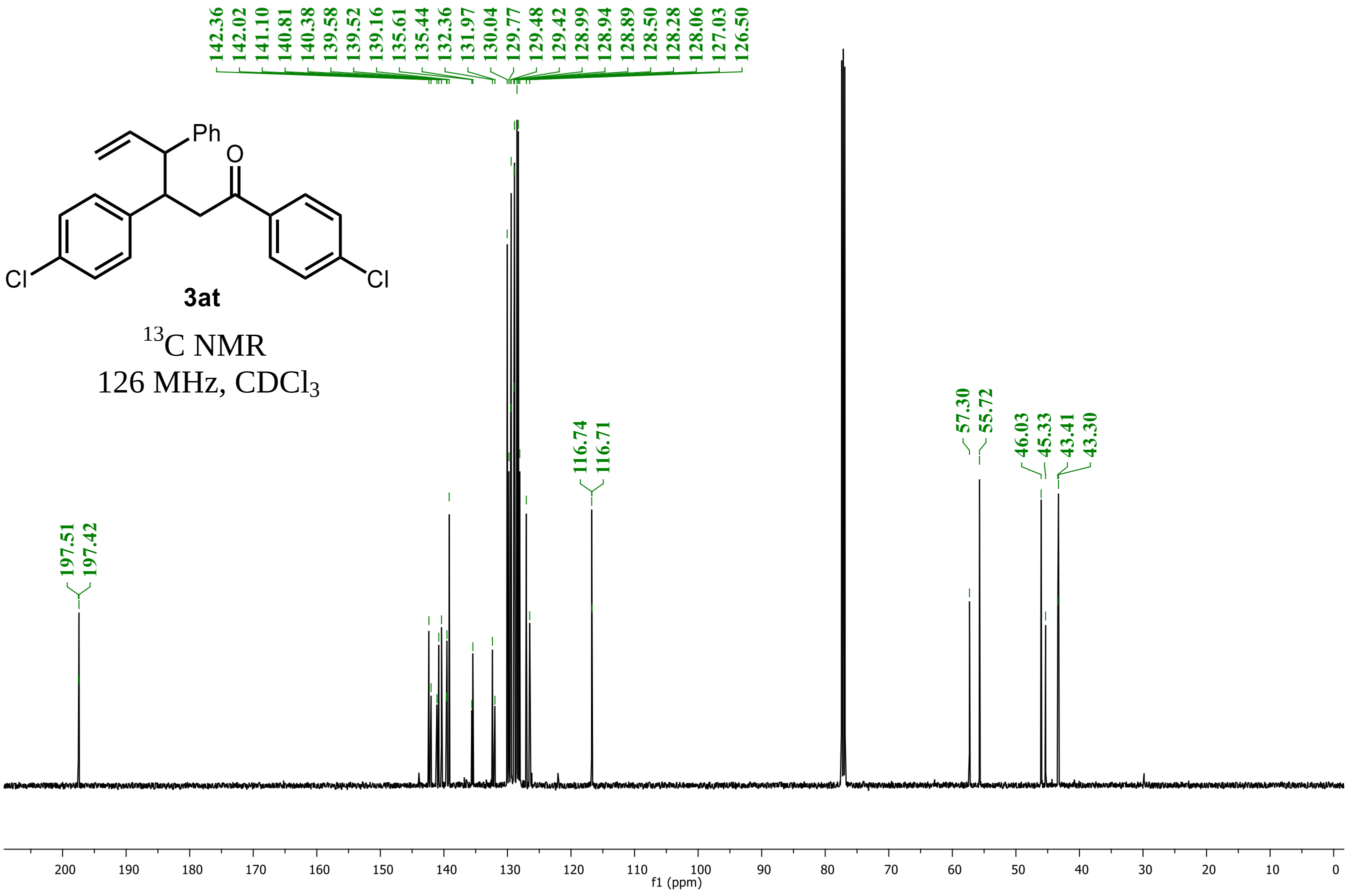

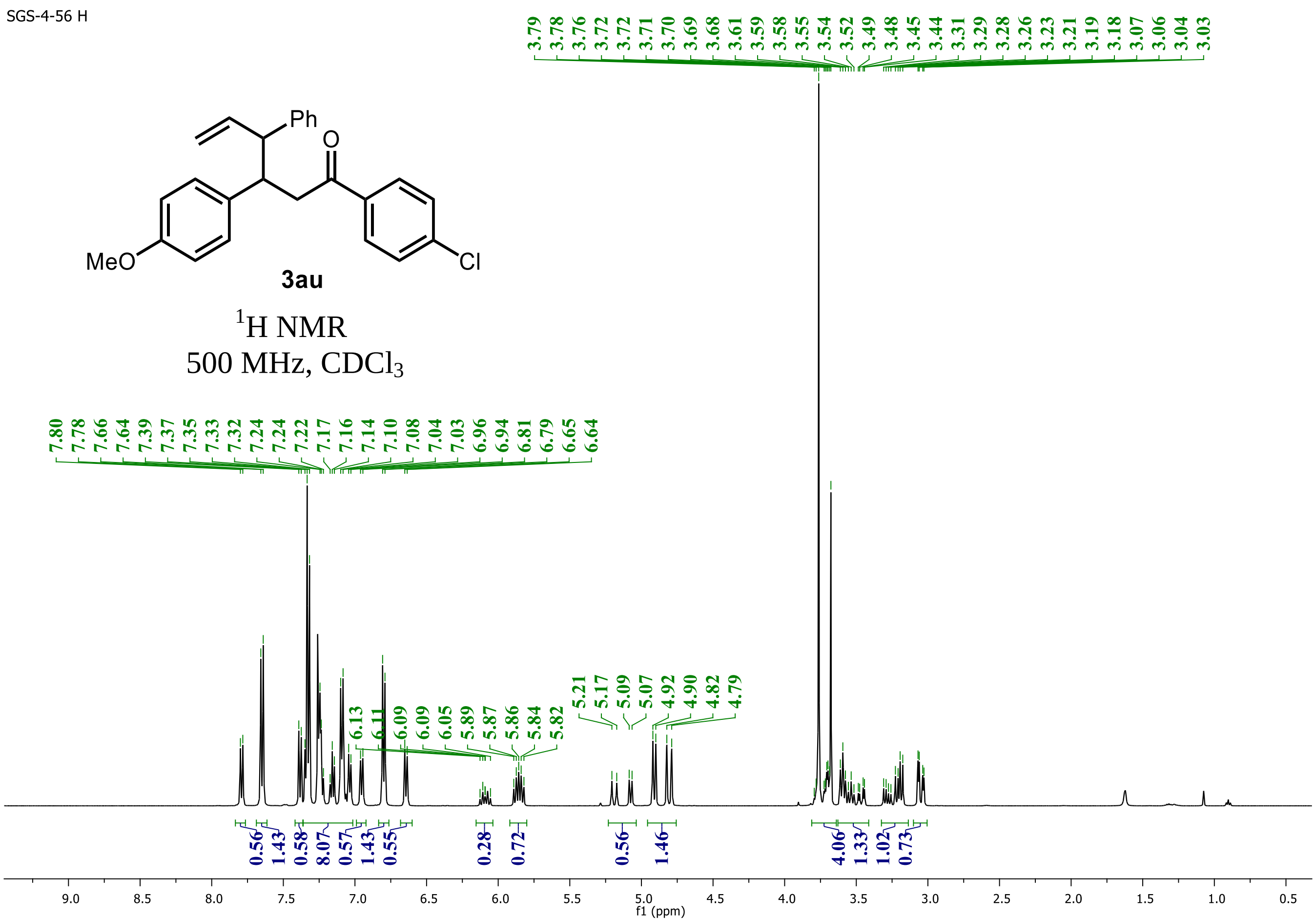
SGS-4-56 H
Ph
O
MeO
Cl
3au
1H NMR
500 MHz, CDCl3
3.79 3.78 3.76 3.72 3.72 3.71 3.70 3.69 3.68 3.61 3.59 3.58 3.55 3.54 3.52 3.49 3.48 3.45 3.44 3.31 3.29 3.28 3.26 3.23 3.21 3.19 3.18 3.07 3.06 3.04 3.03
7.80 7.78 7.66 7.64 7.39 7.37 7.35 7.33 7.32 7.24 7.24 7.22 7.17 7.16 7.14 7.10 7.08 7.04 7.03 6.96 6.94 6.81 6.79 6.65 6.64
6.13 6.11 6.09 6.09 6.05 5.89 5.87 5.86 5.84 5.82
5.21 5.17 5.09 5.07 4.92 4.90 4.82 4.79
0.56 1.43 0.58 8.07 0.57 1.43 0.55 0.28 0.72 0.56 1.46 4.06 1.33 1.02 0.73
9.0 8.5 8.0 7.5 7.0 6.5 6.0 5.5 5.0 4.5 4.0 3.5 3.0 2.5 2.0 1.5 1.0 0.5
f1 (ppm)

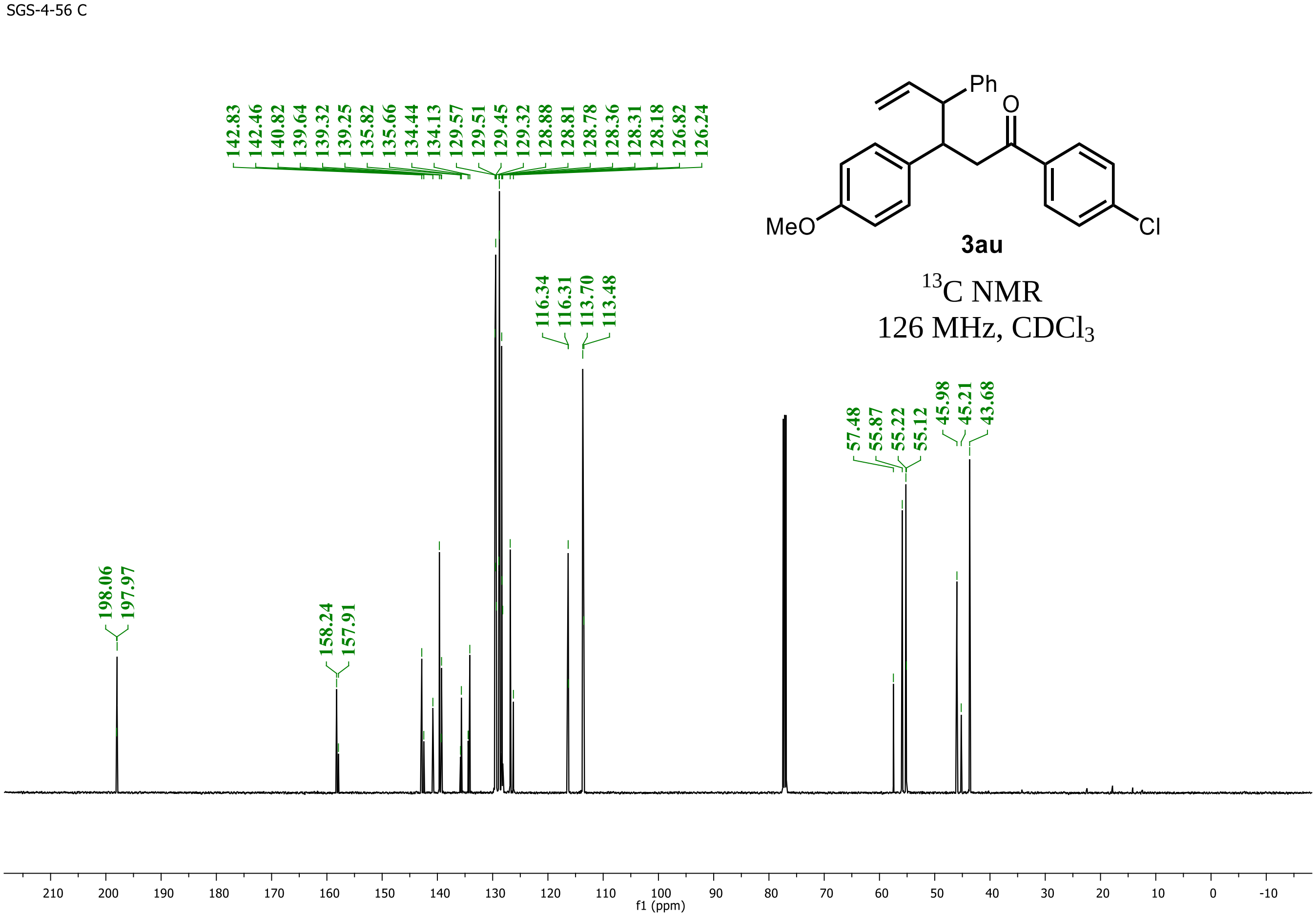
SGS-4-56 C
Ph
O
MeO
Cl
3au
13C NMR
126 MHz, CDCl3
142.83 142.46 140.82 139.64 139.32 139.25 135.82 135.66 134.44 134.13 129.57 129.51 129.45 129.32 128.88 128.81 128.78 128.36 128.31 128.18 126.82 126.24
116.34 116.31 113.70 113.48
57.48 55.87 55.22 55.12 45.98 45.21 43.68
198.06 197.97
158.24 157.91
210 200 190 180 170 160 150 140 130 120 110 100 90 80 70 60 50 40 30 20 10 0 -10
f1 (ppm)

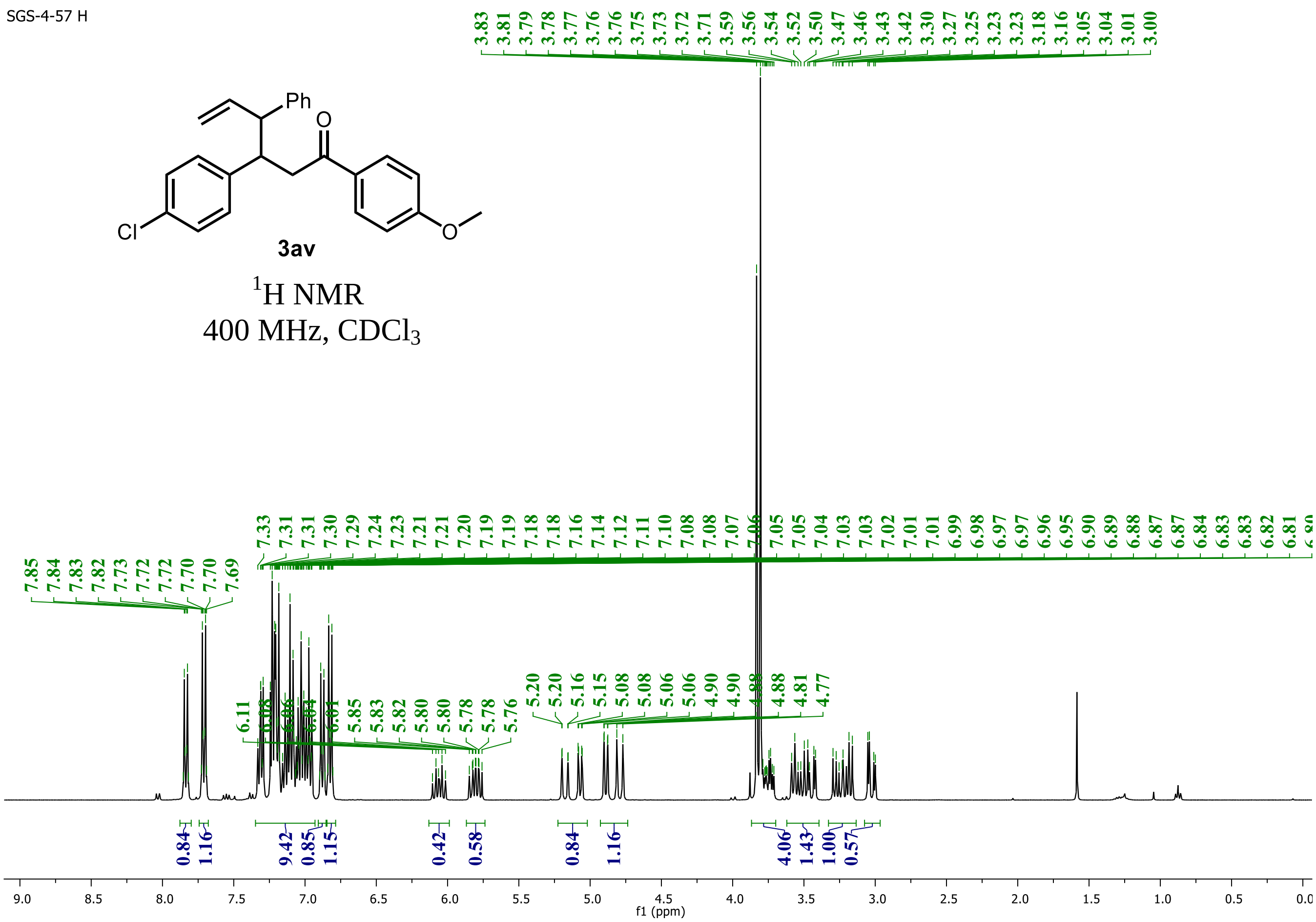
SGS-4-57 H
Ph
O
Cl
O
3av
^{1}H NMR
400 MHz, CDCl$_3$
3.83 3.81 3.79 3.78 3.77 3.76 3.76 3.75 3.73 3.72 3.71 3.59 3.56 3.54 3.52 3.50 3.47 3.46 3.43 3.42 3.30 3.27 3.25 3.23 3.23 3.18 3.16 3.05 3.04 3.01 3.00
7.85 7.84 7.83 7.82 7.73 7.72 7.72 7.70 7.70 7.69
7.33 7.31 7.31 7.30 7.29 7.24 7.23 7.21 7.21 7.20 7.19 7.19 7.18 7.18 7.16 7.14 7.12 7.11 7.10 7.08 7.08 7.07 7.05 7.05 7.04 7.03 7.03 7.02 7.01 7.01 6.99 6.98 6.97 6.97 6.96 6.95 6.90 6.89 6.88 6.87 6.87 6.84 6.83 6.83 6.82 6.81
6.11 5.85 5.83 5.82 5.80 5.80 5.78 5.78 5.76
5.20 5.20 5.16 5.15 5.08 5.08 5.06 5.06 4.90 4.90 4.88 4.81 4.77
0.84 1.16 9.42 0.85 1.15 0.42 0.58 0.84 1.16 4.06 1.43 1.00 0.57
9.0 8.5 8.0 7.5 7.0 6.5 6.0 5.5 5.0 4.5 4.0 3.5 3.0 2.5 2.0 1.5 1.0 0.5 0.0
f1 (ppm)

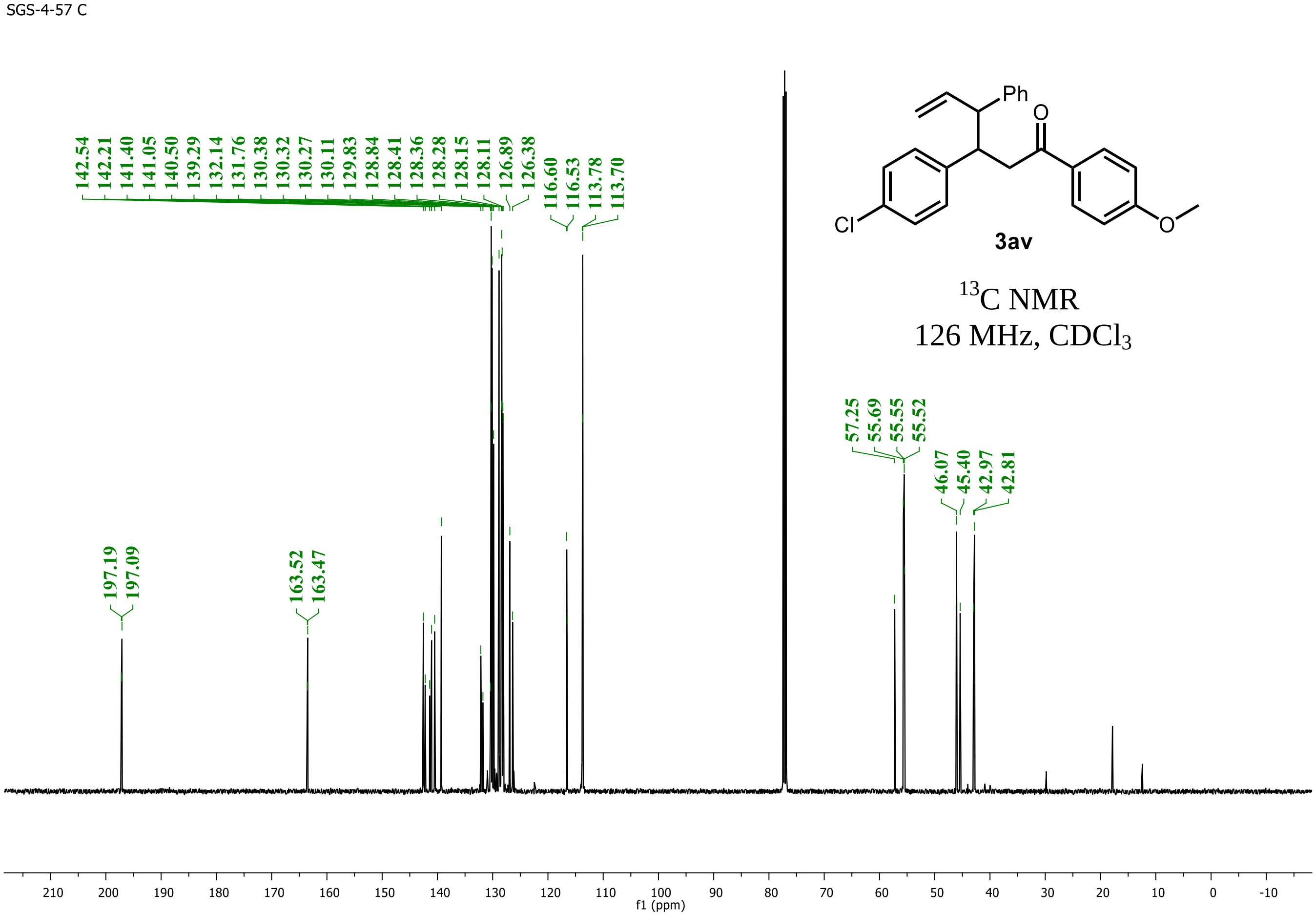
SGS-4-57 C
Ph
O
Cl
O
3av
^{13}C NMR
126 MHz, CDCl$_3$
142.54 142.21 141.40 141.05 140.50 139.29 132.14 131.76 130.38 130.32 130.27 130.11 129.83 128.84 128.41 128.36 128.28 128.15 128.11 126.89 126.38
116.60 116.53 113.78 113.70
57.25 55.69 55.55 55.52
46.07 45.40 42.97 42.81
197.19 197.09
163.52 163.47
210 200 190 180 170 160 150 140 130 120 110 100 90 80 70 60 50 40 30 20 10 0 -10
f1 (ppm)

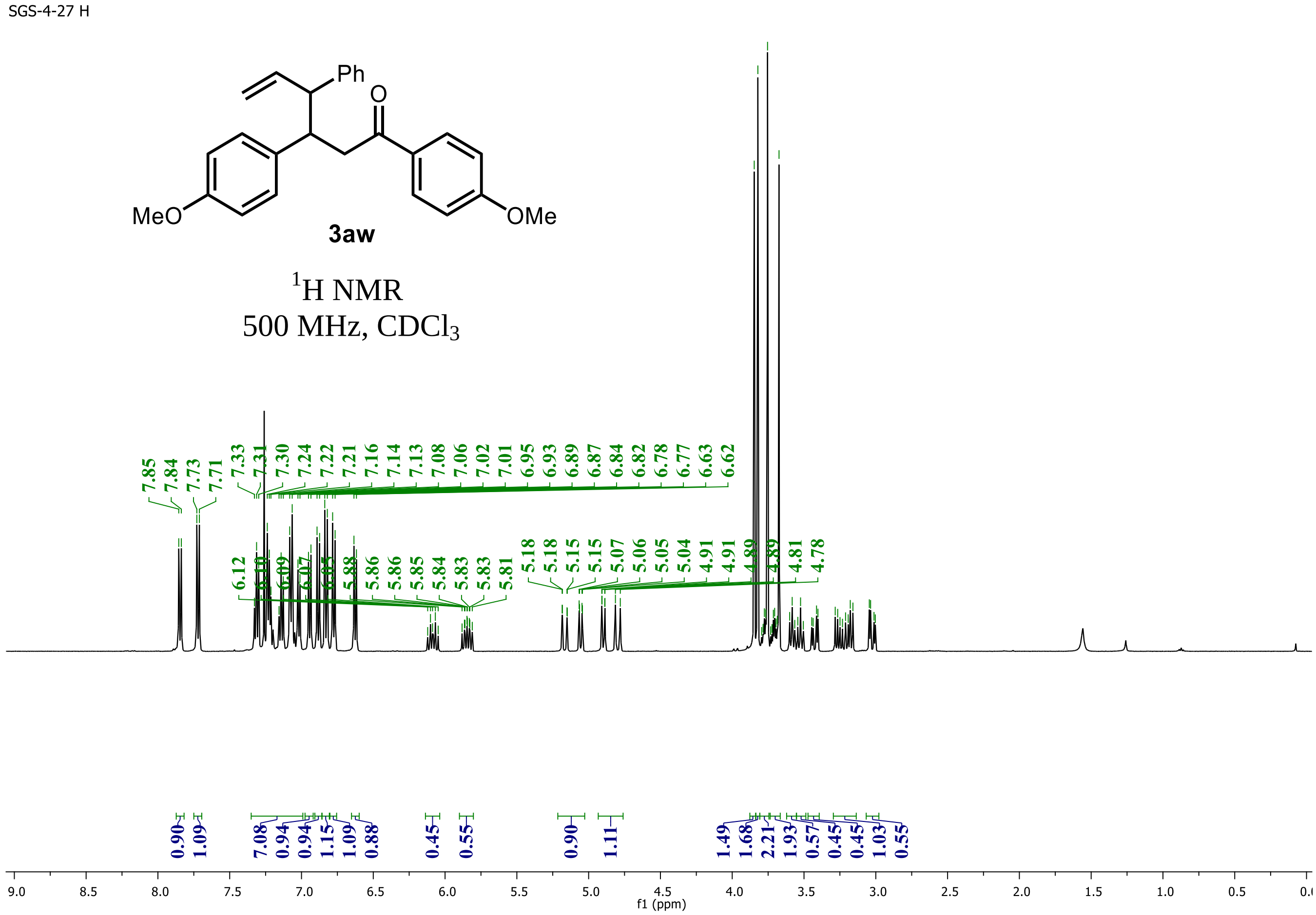
SGS-4-27 H
Ph
O
MeO
OMe
3aw
1H NMR
500 MHz, CDCl3
7.85 7.84 7.73 7.71 7.33 7.31 7.30 7.24 7.22 7.21 7.16 7.14 7.13 7.08 7.06 7.02 7.01 6.95 6.93 6.89 6.87 6.84 6.82 6.78 6.77 6.63 6.62
6.12 6.10 6.09 6.07 6.04 5.88 5.86 5.86 5.85 5.84 5.83 5.83 5.81
5.18 5.18 5.15 5.15 5.07 5.06 5.05 5.04 4.91 4.89 4.89 4.81 4.78
0.90 1.09 7.08 0.94 0.94 1.15 1.09 0.88 0.45 0.55 0.90 1.11 1.49 1.68 2.21 1.93 0.57 0.45 0.45 1.03 0.55
f1 (ppm)

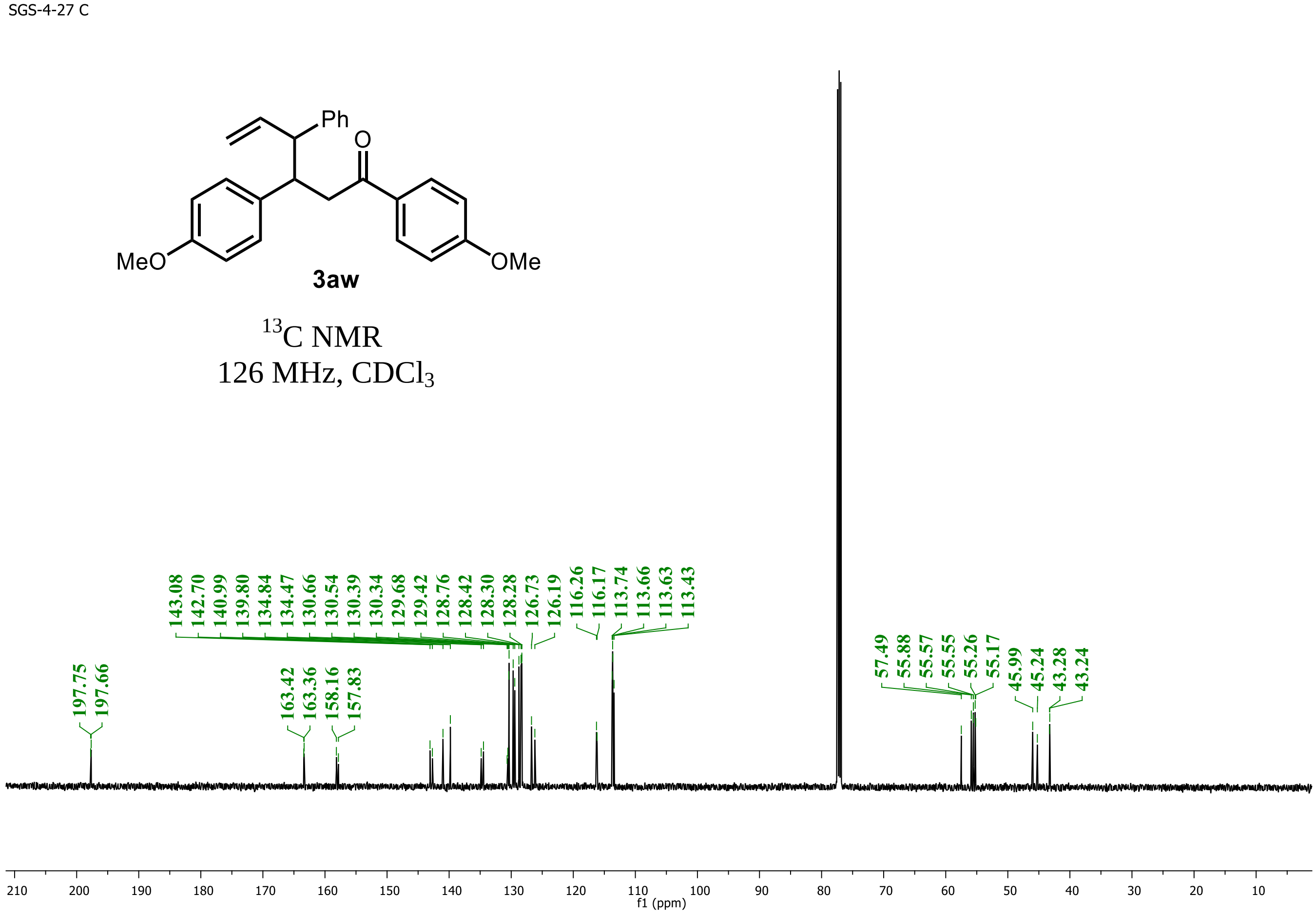
SGS-4-27 C
Ph
O
MeO
OMe
3aw
13C NMR
126 MHz, CDCl3
197.75 197.66
163.42 163.36 158.16 157.83
143.08 142.70 140.99 139.80 134.84 134.47 130.66 130.54 130.39 130.34 129.68 129.42 128.76 128.42 128.30 128.28 126.73 126.19
116.26 116.17 113.74 113.66 113.63 113.43
57.49 55.88 55.57 55.55 55.26 55.17 45.99 45.24 43.28 43.24
f1 (ppm)

Copies of NMR spectra of products: Nucleophile scope

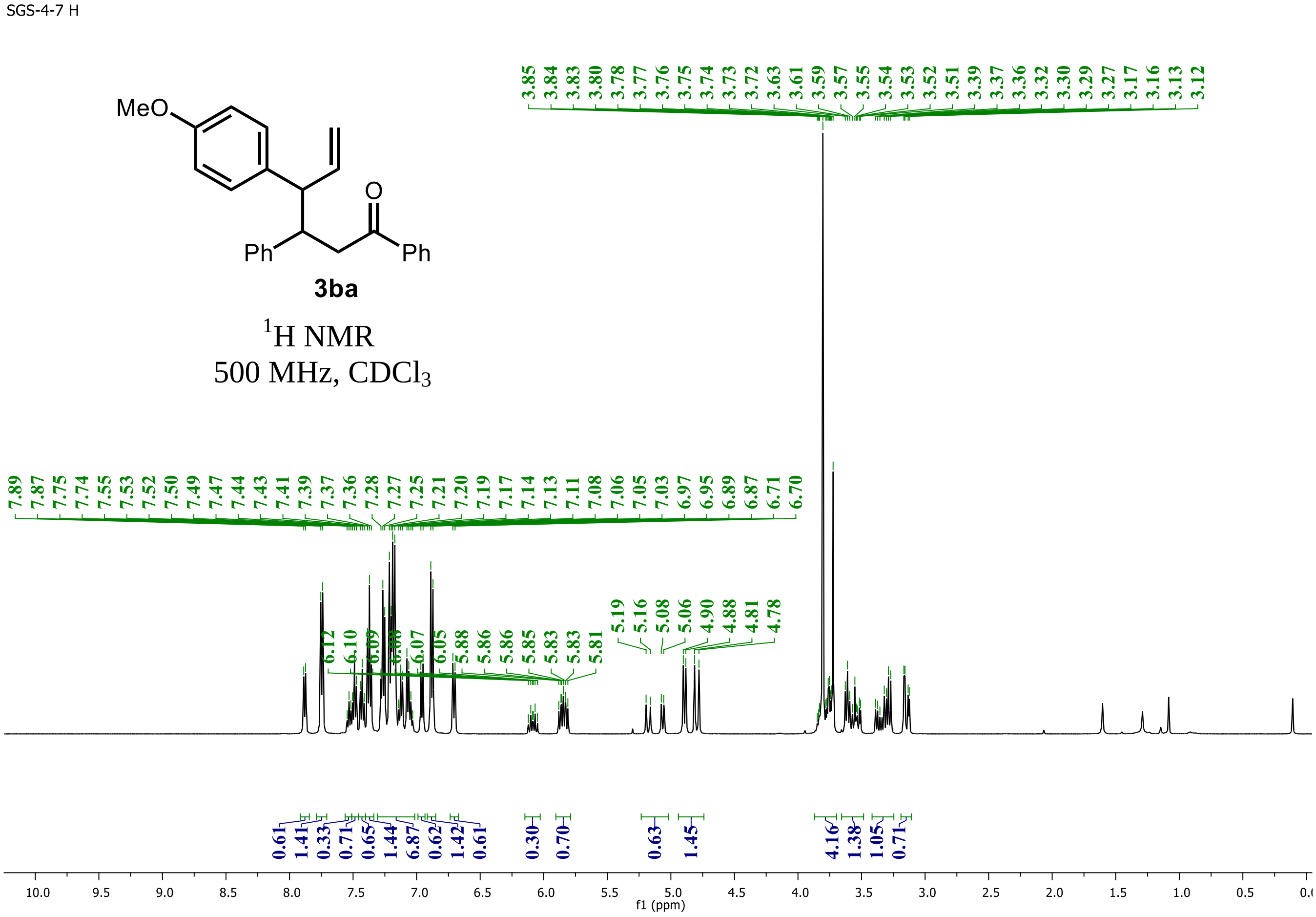

SGS-4-7 C

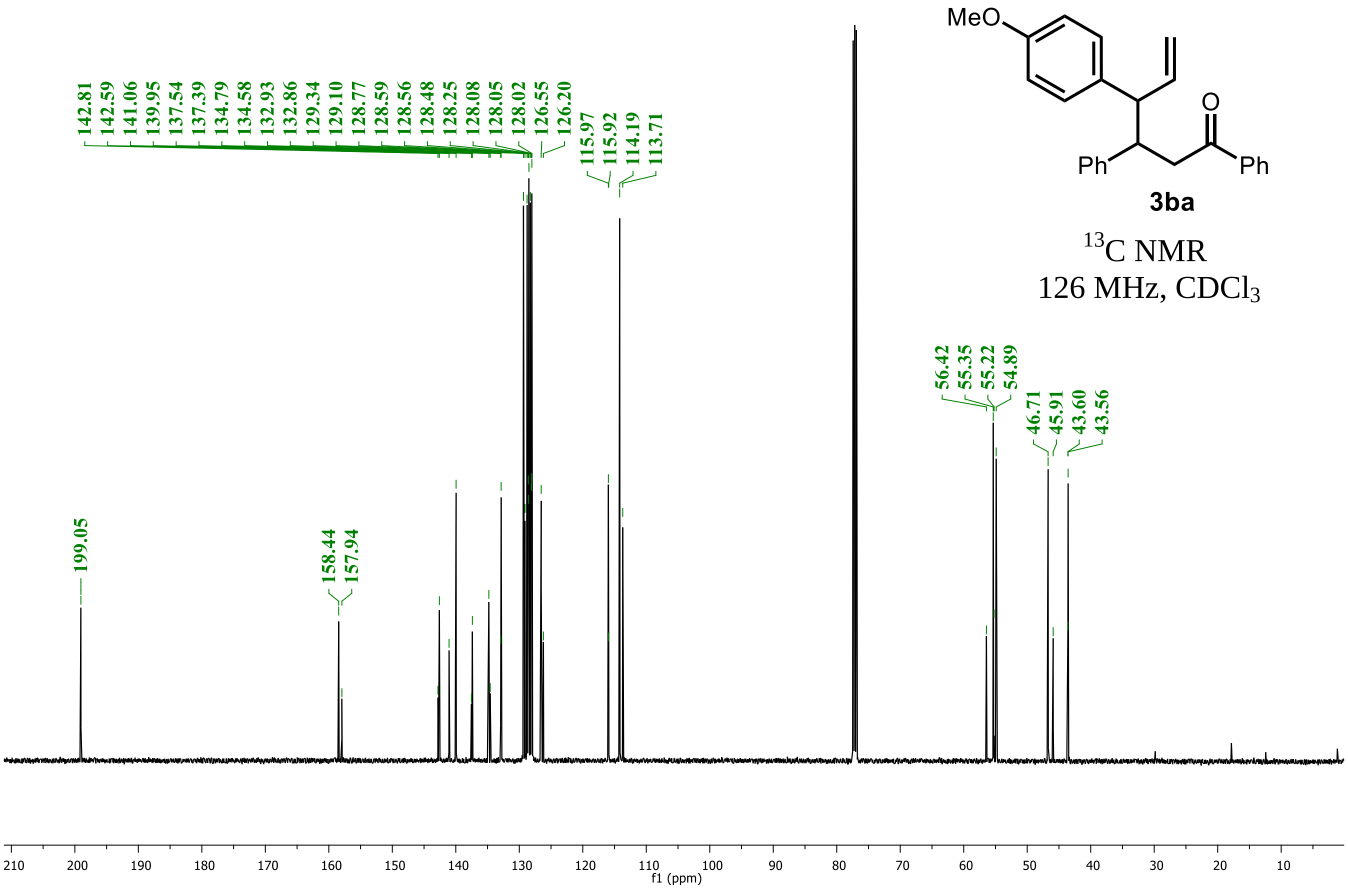

3ba

^{13}C NMR
126 MHz, $CDCl_3$

SGS-4-13 H

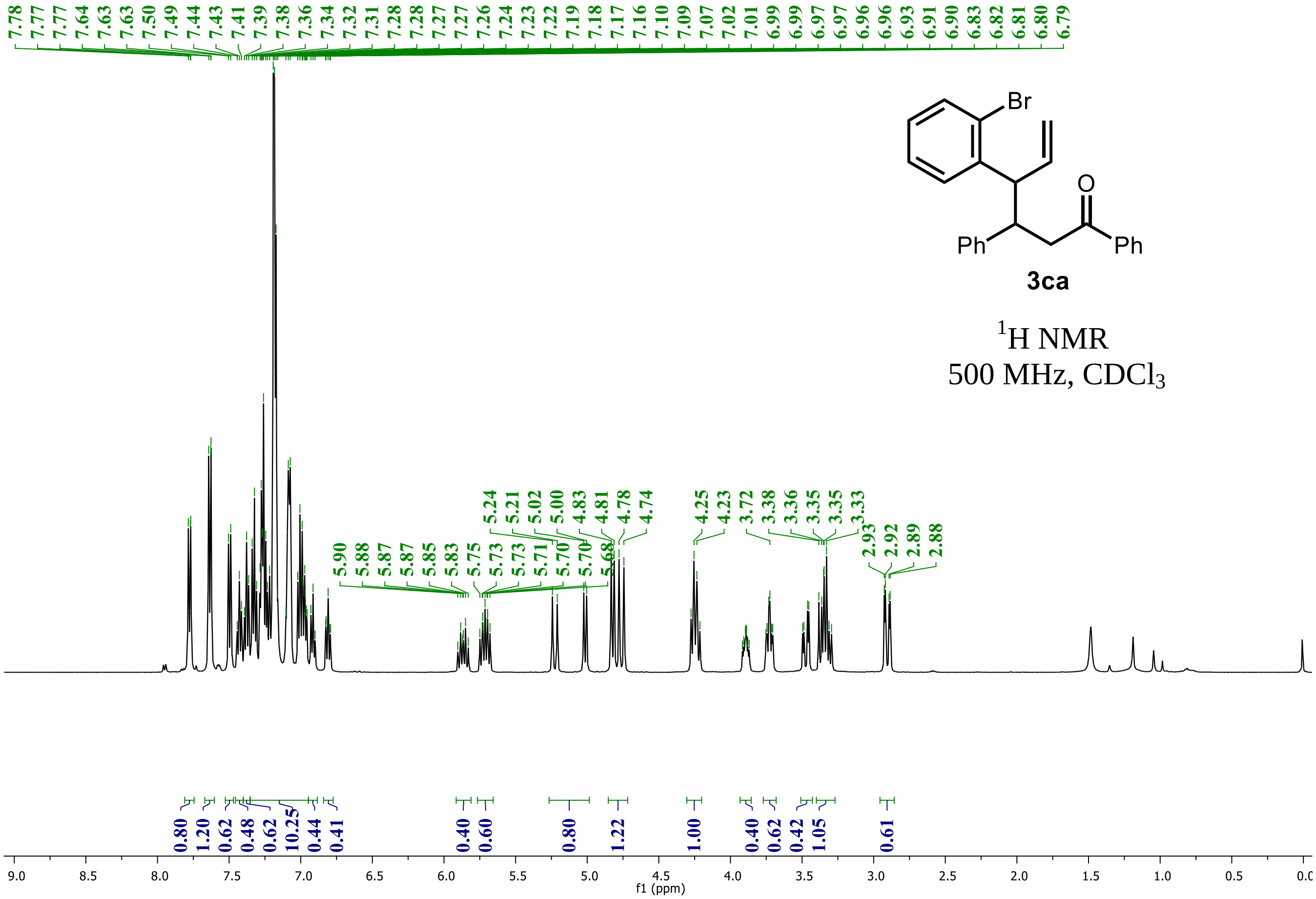

3ca

^{1}H NMR
500 MHz, $CDCl_3$

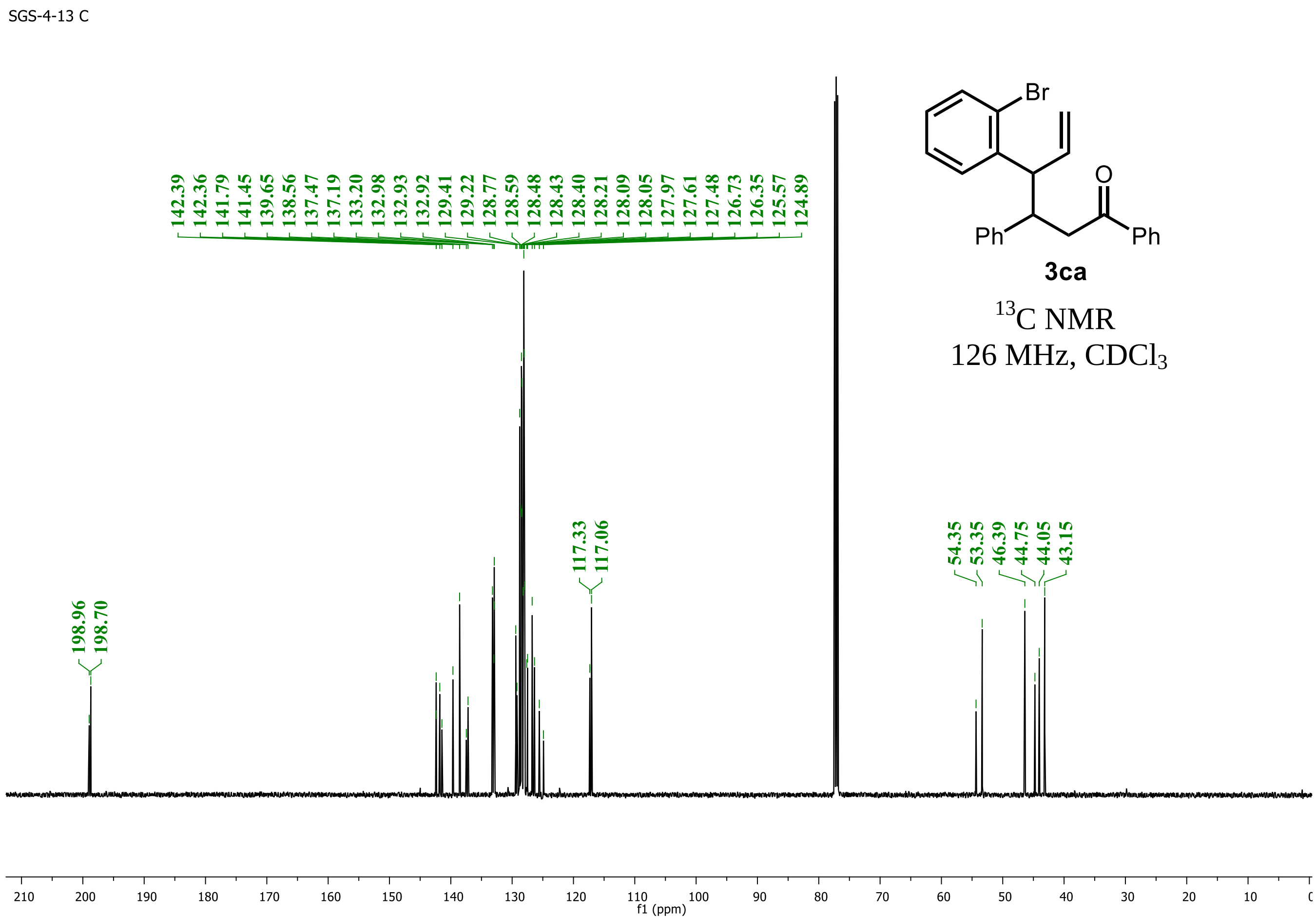
SGS-4-13 C
142.39 142.36 141.79 141.45 139.65 138.56 137.47 137.19 133.20 132.98 132.93 132.92 129.41 129.22 128.77 128.59 128.48 128.43 128.40 128.21 128.09 128.05 127.97 127.61 127.48 126.73 126.35 125.57 124.89
198.96 198.70
117.33 117.06
54.35 53.35 46.39 44.75 44.05 43.15
Br
O
Ph
Ph
3ca
13C NMR
126 MHz, CDCl3
f1 (ppm)

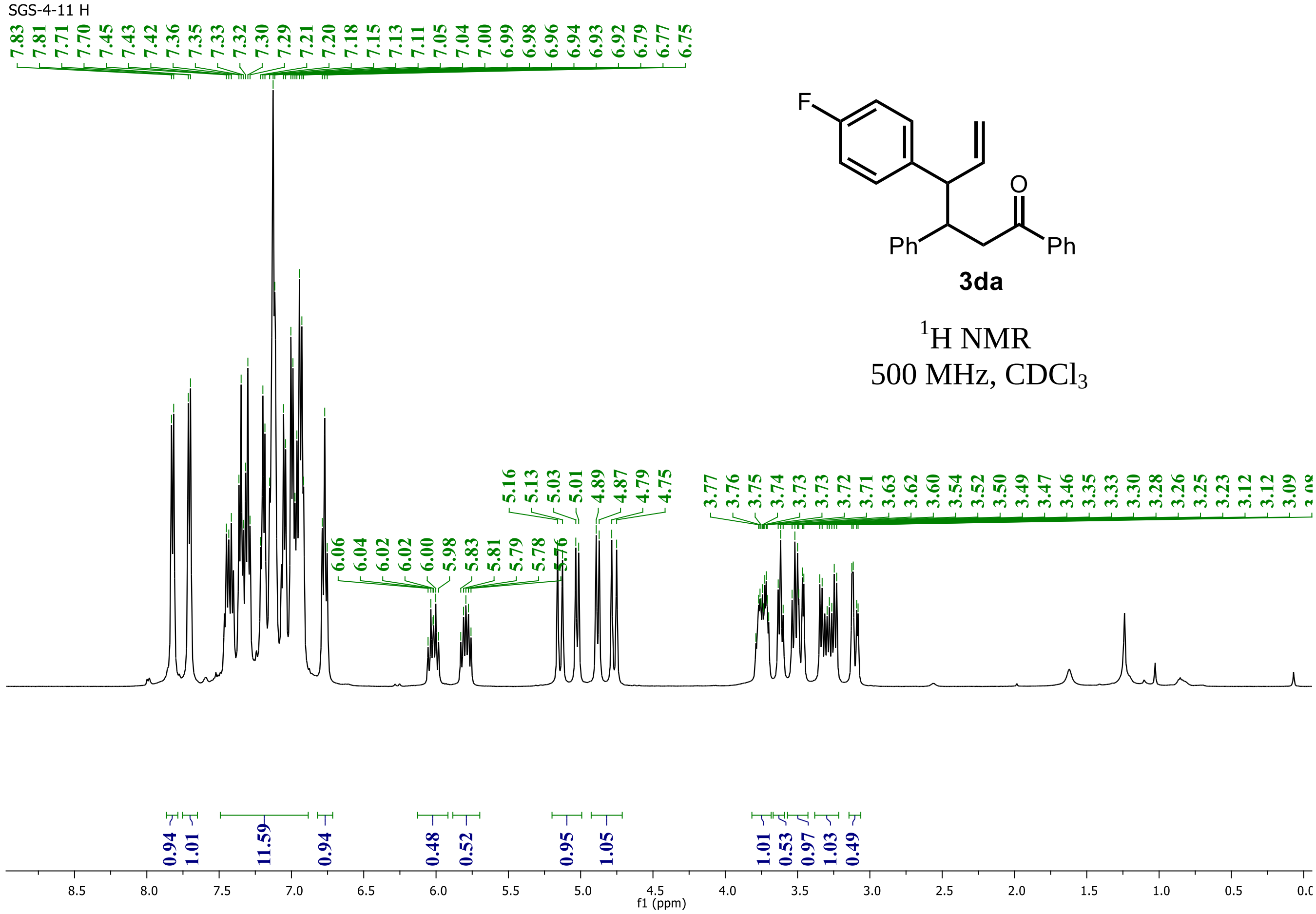
SGS-4-11 H
7.83 7.81 7.71 7.70 7.45 7.43 7.42 7.36 7.35 7.33 7.32 7.30 7.29 7.21 7.20 7.18 7.15 7.13 7.11 7.05 7.04 7.00 6.99 6.98 6.96 6.94 6.93 6.92 6.79 6.77 6.75
6.06 6.04 6.02 6.02 6.00 5.98 5.83 5.81 5.79 5.78 5.76
5.16 5.13 5.03 5.01 4.89 4.87 4.79 4.75
3.77 3.76 3.75 3.74 3.73 3.73 3.72 3.71 3.63 3.62 3.60 3.54 3.52 3.50 3.49 3.47 3.46 3.35 3.33 3.30 3.28 3.26 3.25 3.23 3.12 3.12 3.09
F
O
Ph
Ph
3da
1H NMR
500 MHz, CDCl3
0.94 1.01 11.59 0.94 0.48 0.52 0.95 1.05 1.01 0.53 0.97 1.03 0.49
f1 (ppm)

SGS-4-11 C

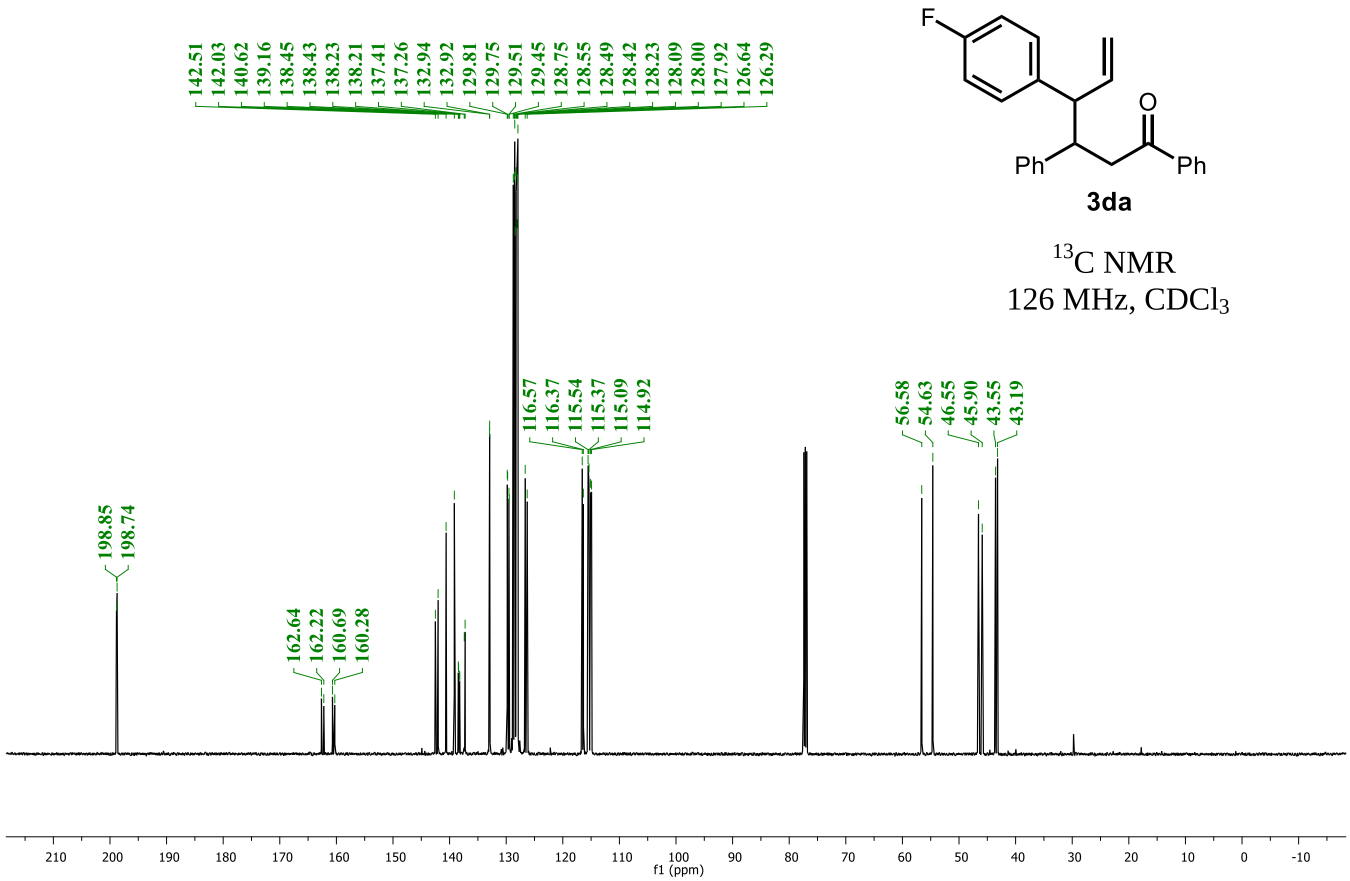

3da

^{13}C NMR
126 MHz, $CDCl_3$

SGS-4-11 F

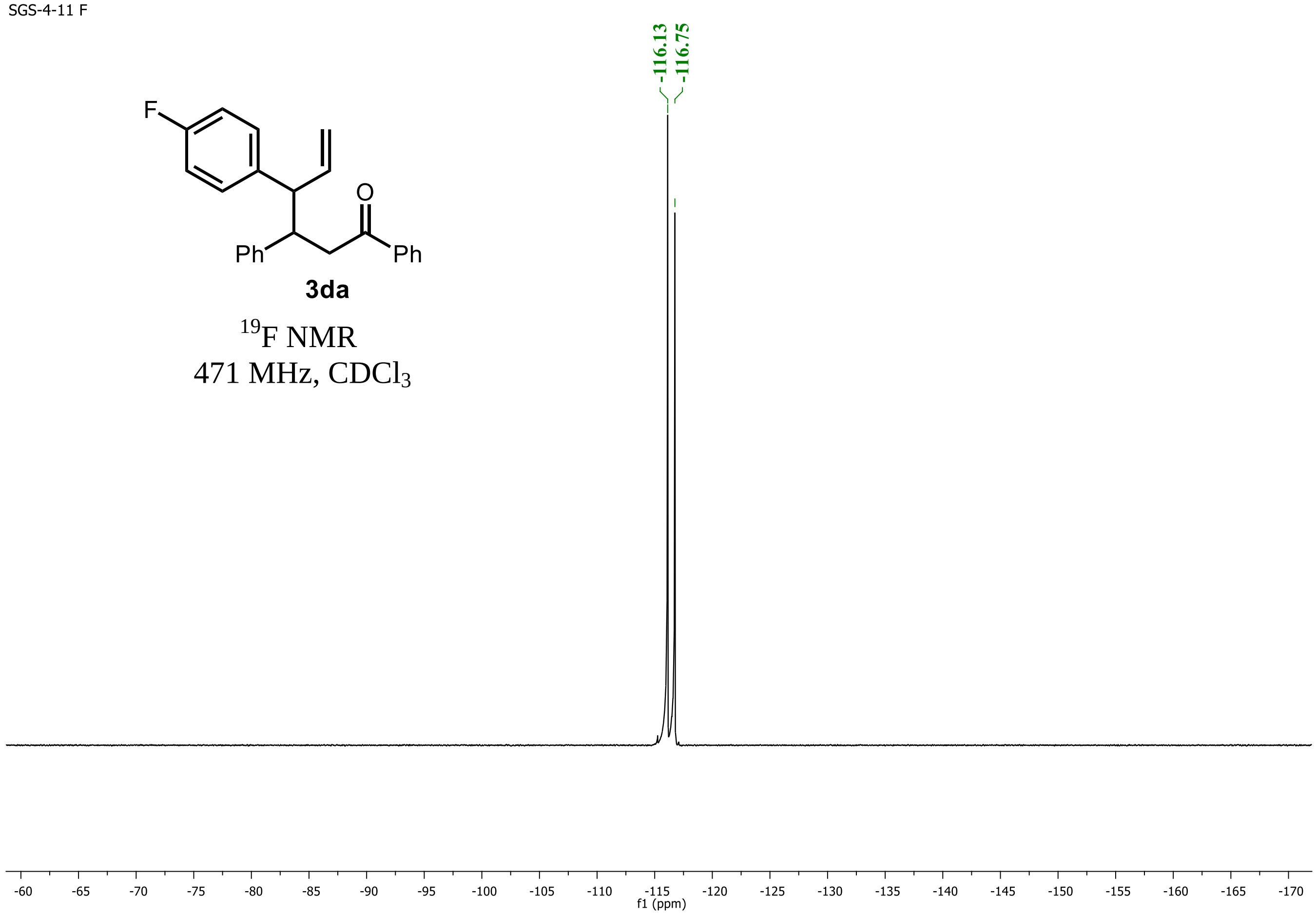

3da

^{19}F NMR
471 MHz, $CDCl_3$

SGS-4-15 H

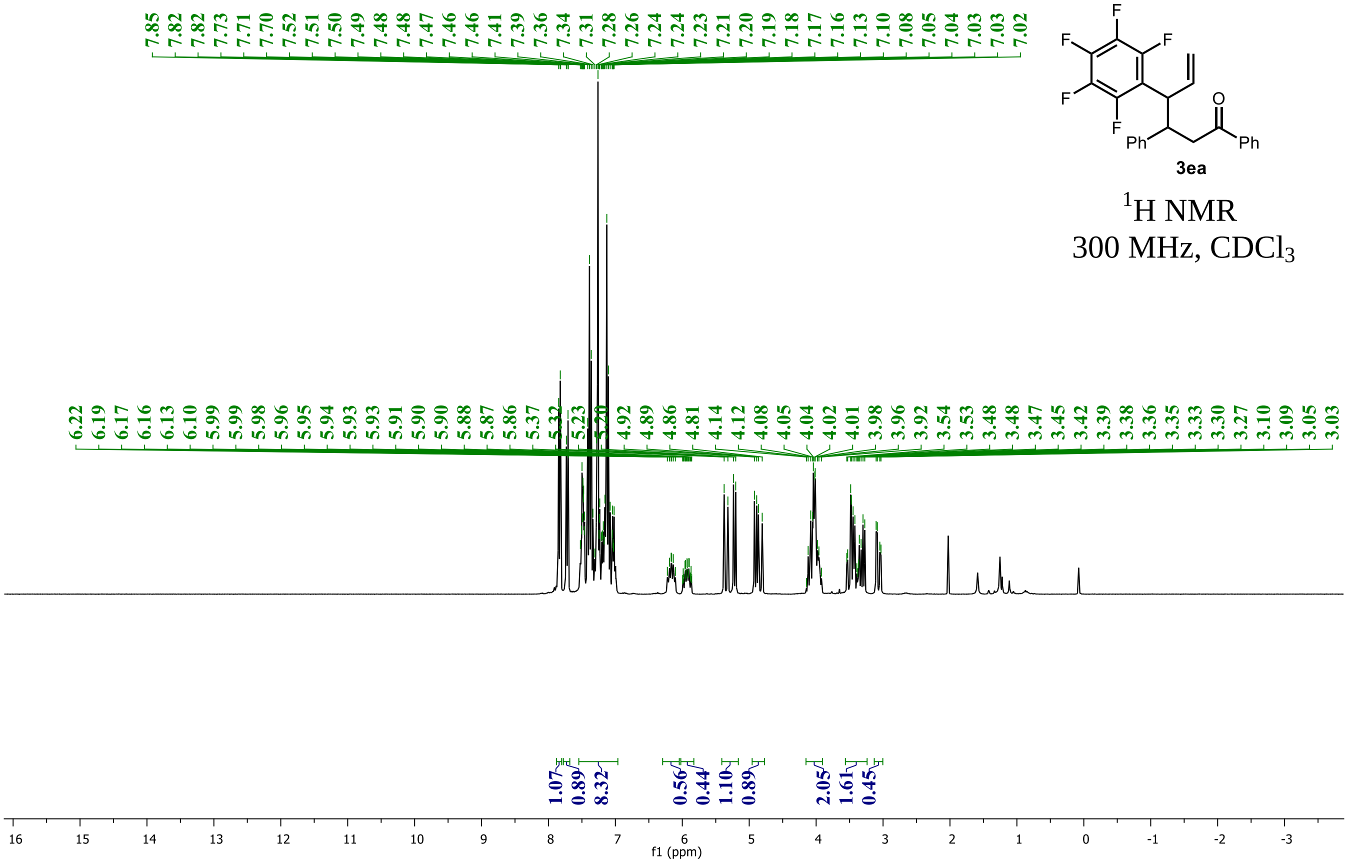

SGS-4-15 C

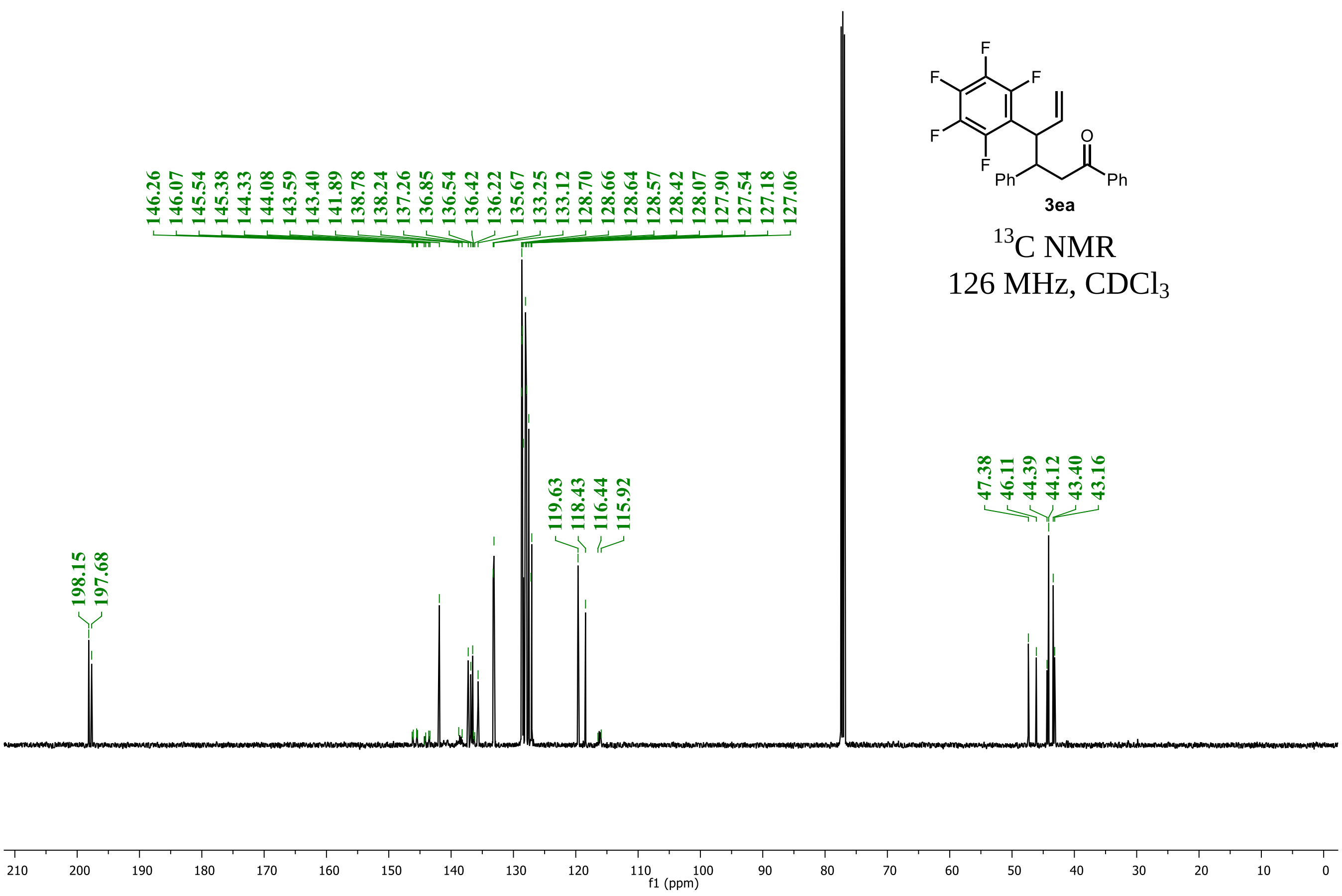

SGS-4-15 F

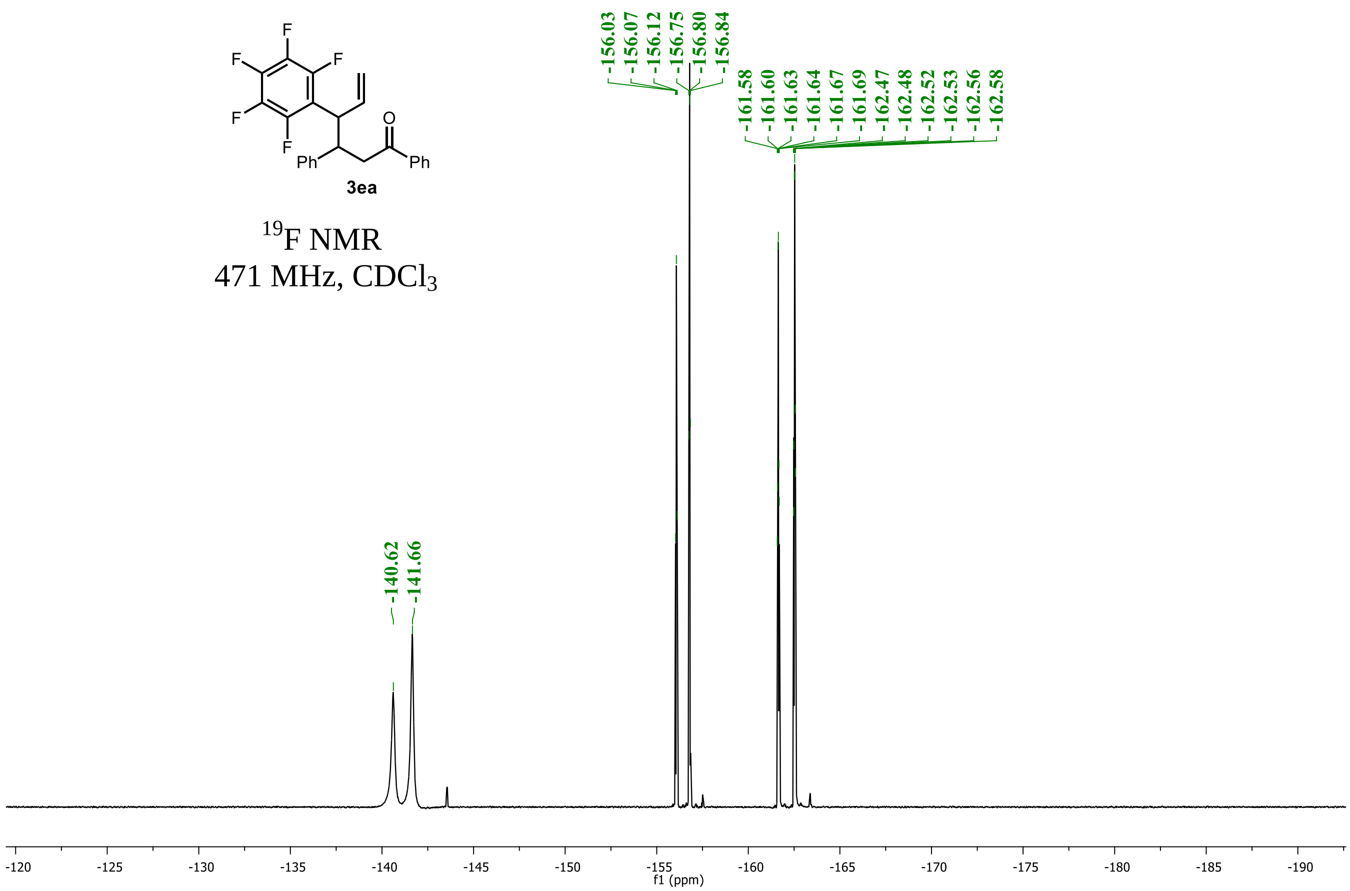

SGS-4-8 H

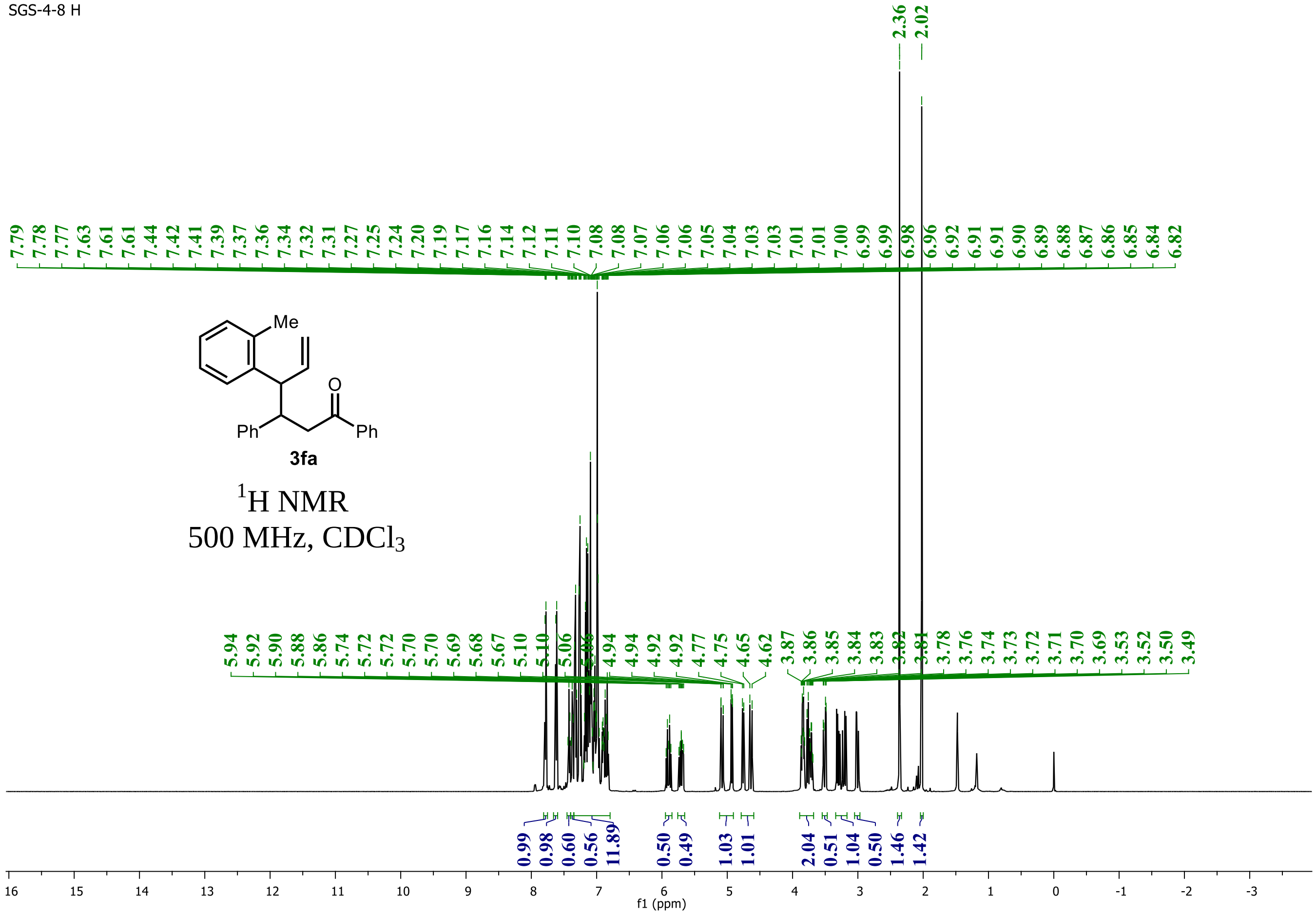

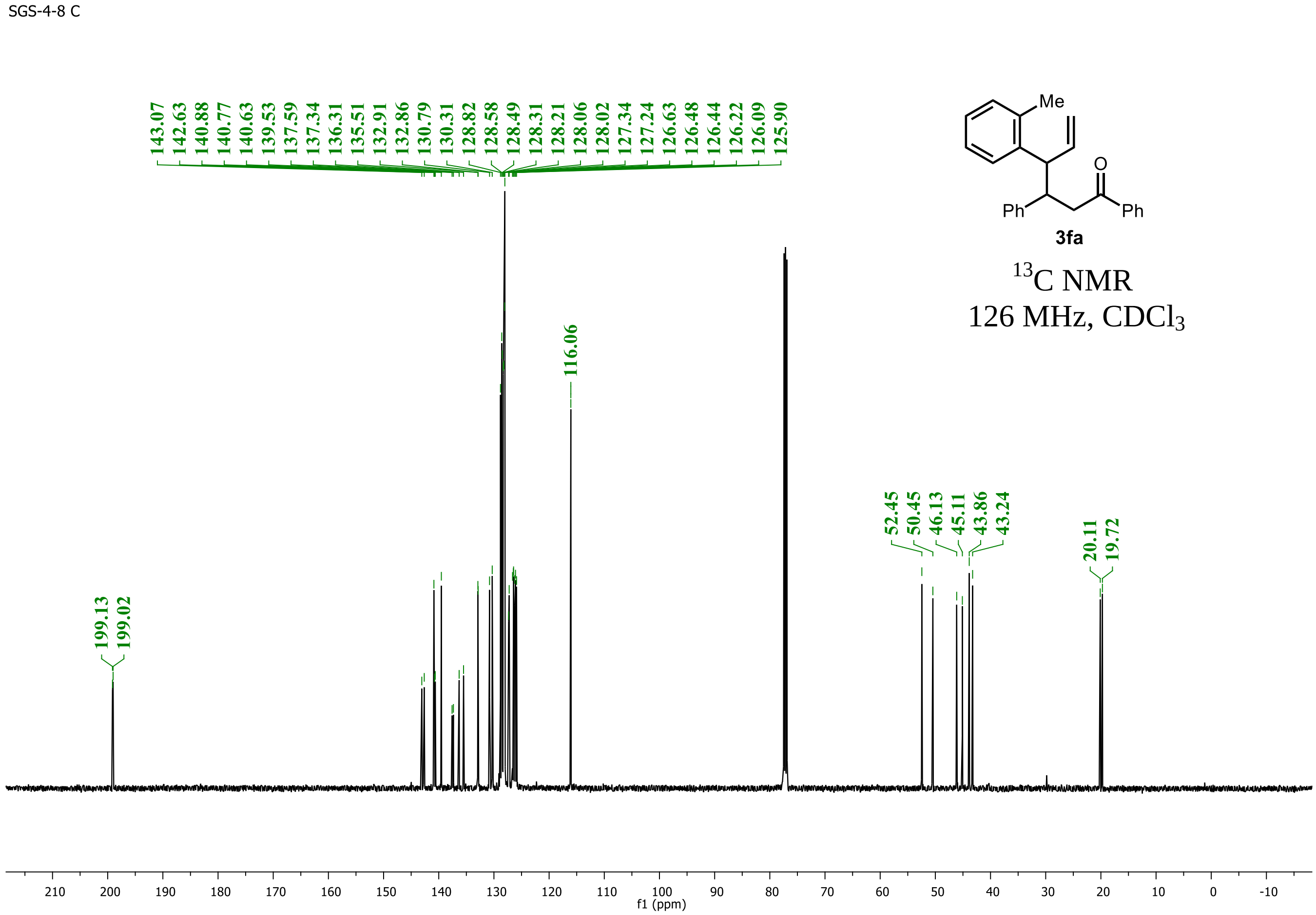
SGS-4-8 C
143.07
142.63
140.88
140.77
140.63
139.53
137.59
137.34
136.31
135.51
132.91
132.86
130.79
130.31
128.82
128.58
128.49
128.31
128.21
128.06
128.02
127.34
127.24
126.63
126.48
126.44
126.22
126.09
125.90
116.06
199.13
199.02
52.45
50.45
46.13
45.11
43.86
43.24
20.11
19.72
Me
O
Ph
Ph
3fa
^{13}C NMR
126 MHz, $CDCl_3$
f1 (ppm)

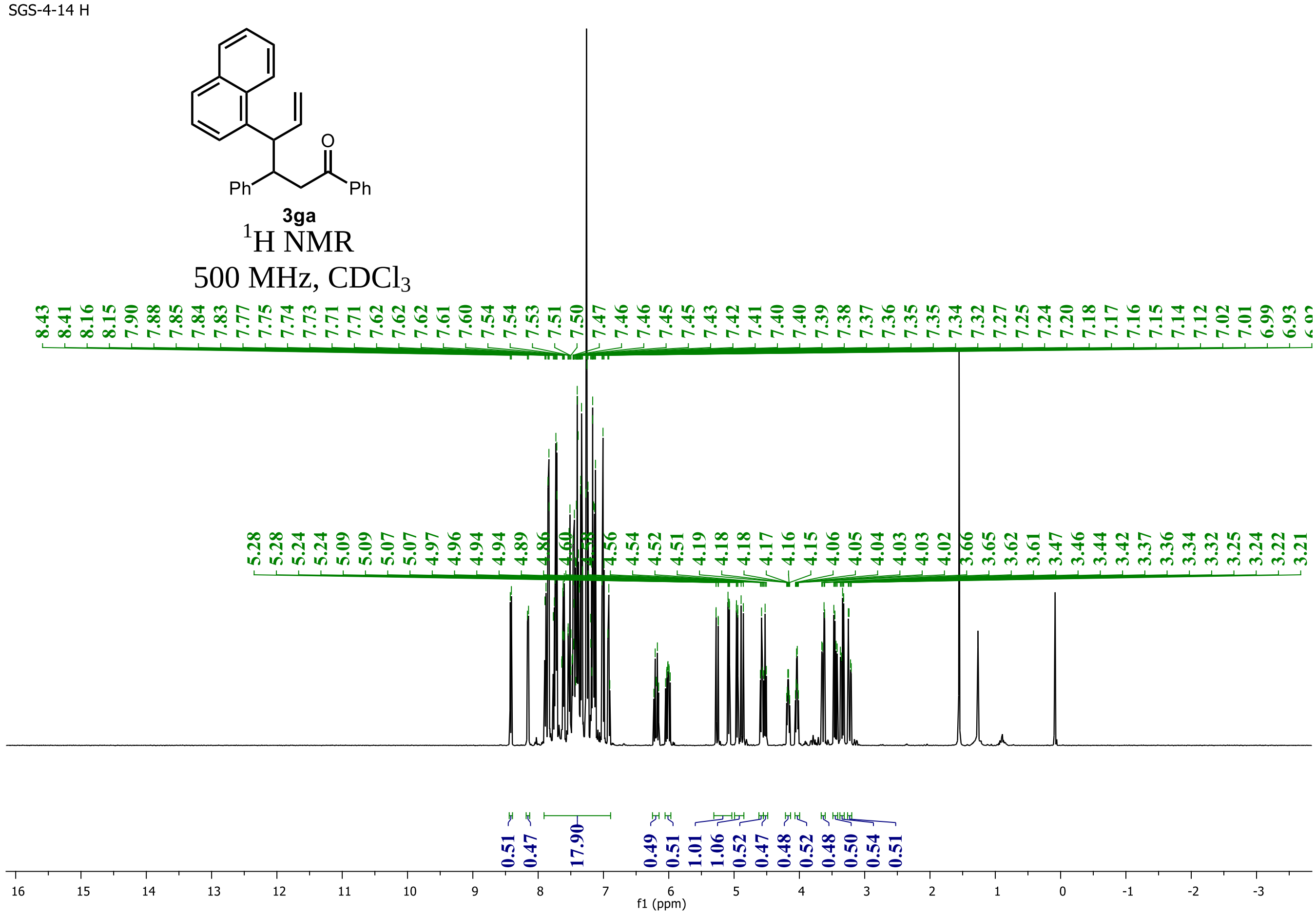
SGS-4-14 H
O
Ph
Ph
3ga
^{1}H NMR
500 MHz, $CDCl_3$
8.43 8.41 8.16 8.15 7.90 7.88 7.85 7.84 7.83 7.77 7.75 7.74 7.73 7.71 7.71 7.62 7.62 7.62 7.61 7.60 7.54 7.54 7.53 7.51 7.50 7.47 7.46 7.46 7.45 7.45 7.43 7.42 7.41 7.40 7.40 7.39 7.38 7.37 7.36 7.35 7.35 7.34 7.32 7.27 7.25 7.24 7.20 7.18 7.17 7.16 7.15 7.14 7.12 7.02 7.01 6.99 6.93
5.28 5.28 5.24 5.24 5.09 5.09 5.07 4.97 4.96 4.94 4.94 4.89 4.86 4.60 4.56 4.54 4.52 4.51 4.19 4.18 4.18 4.17 4.16 4.15 4.06 4.05 4.04 4.03 4.03 4.02 3.66 3.65 3.62 3.61 3.47 3.46 3.44 3.42 3.37 3.36 3.34 3.32 3.25 3.24 3.22 3.21
0.51 0.47 17.90 0.49 0.51 1.01 1.06 0.52 0.47 0.48 0.52 0.48 0.50 0.54 0.51
f1 (ppm)

SGS-4-14 C

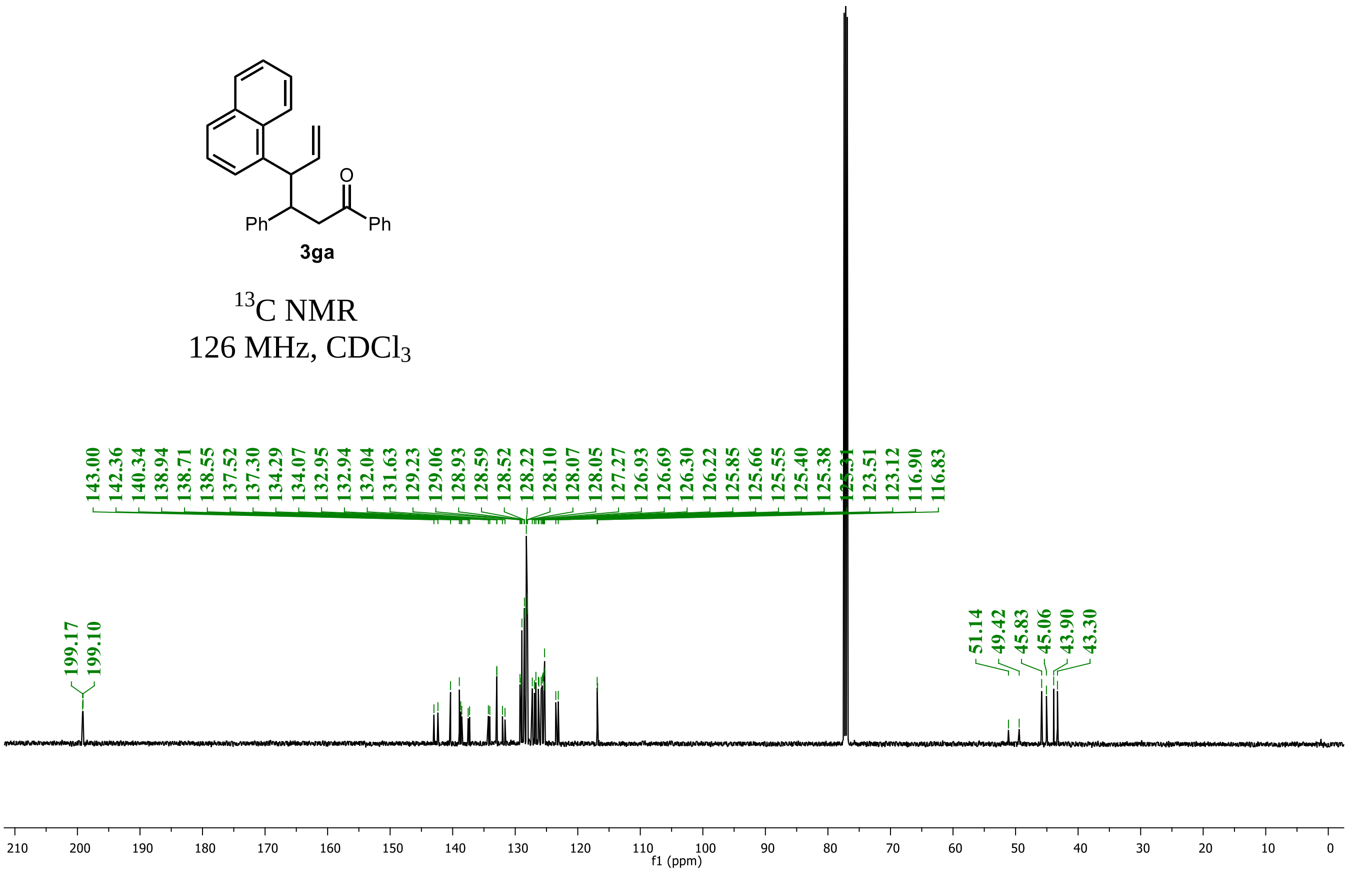

SGS-4-30 1H

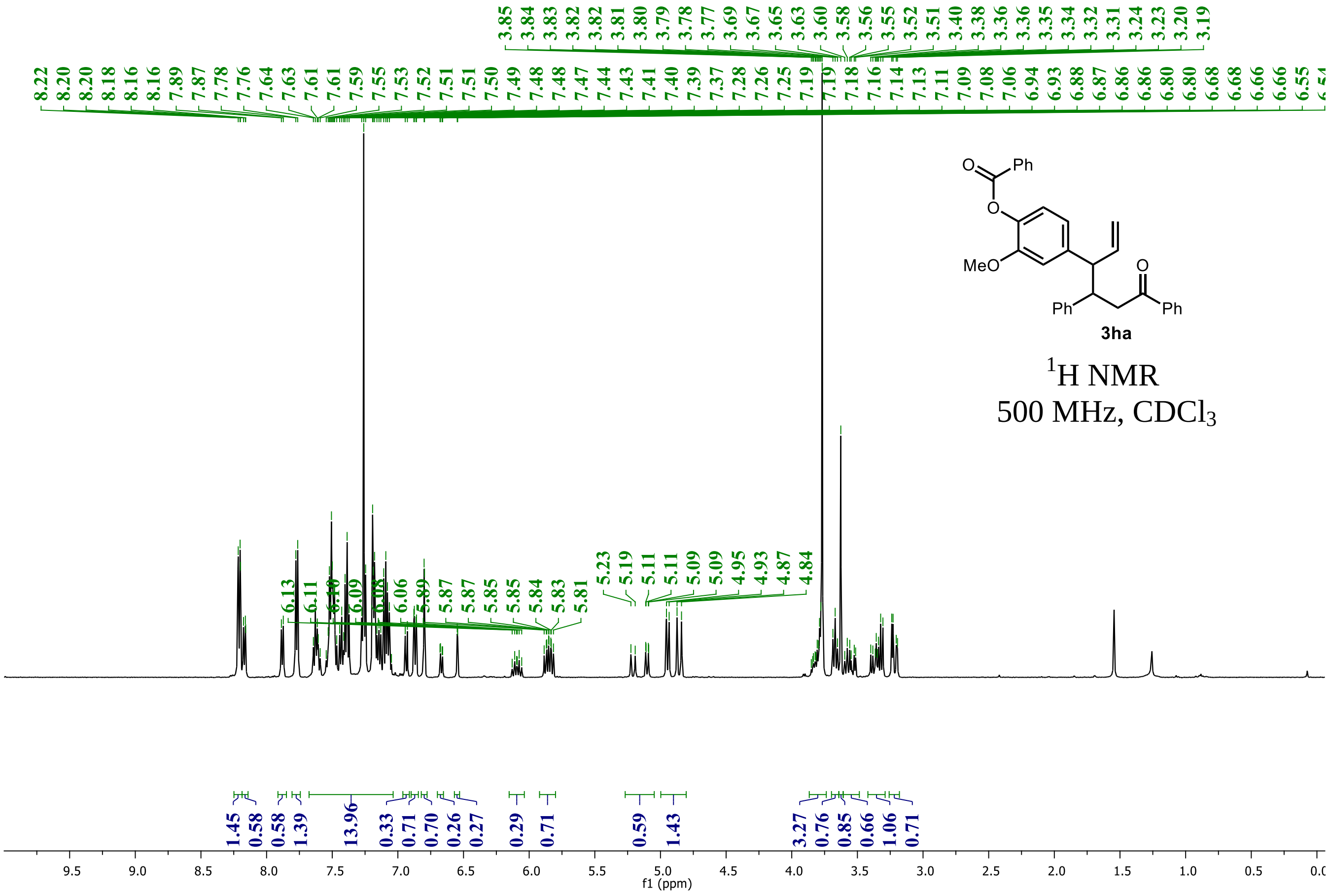

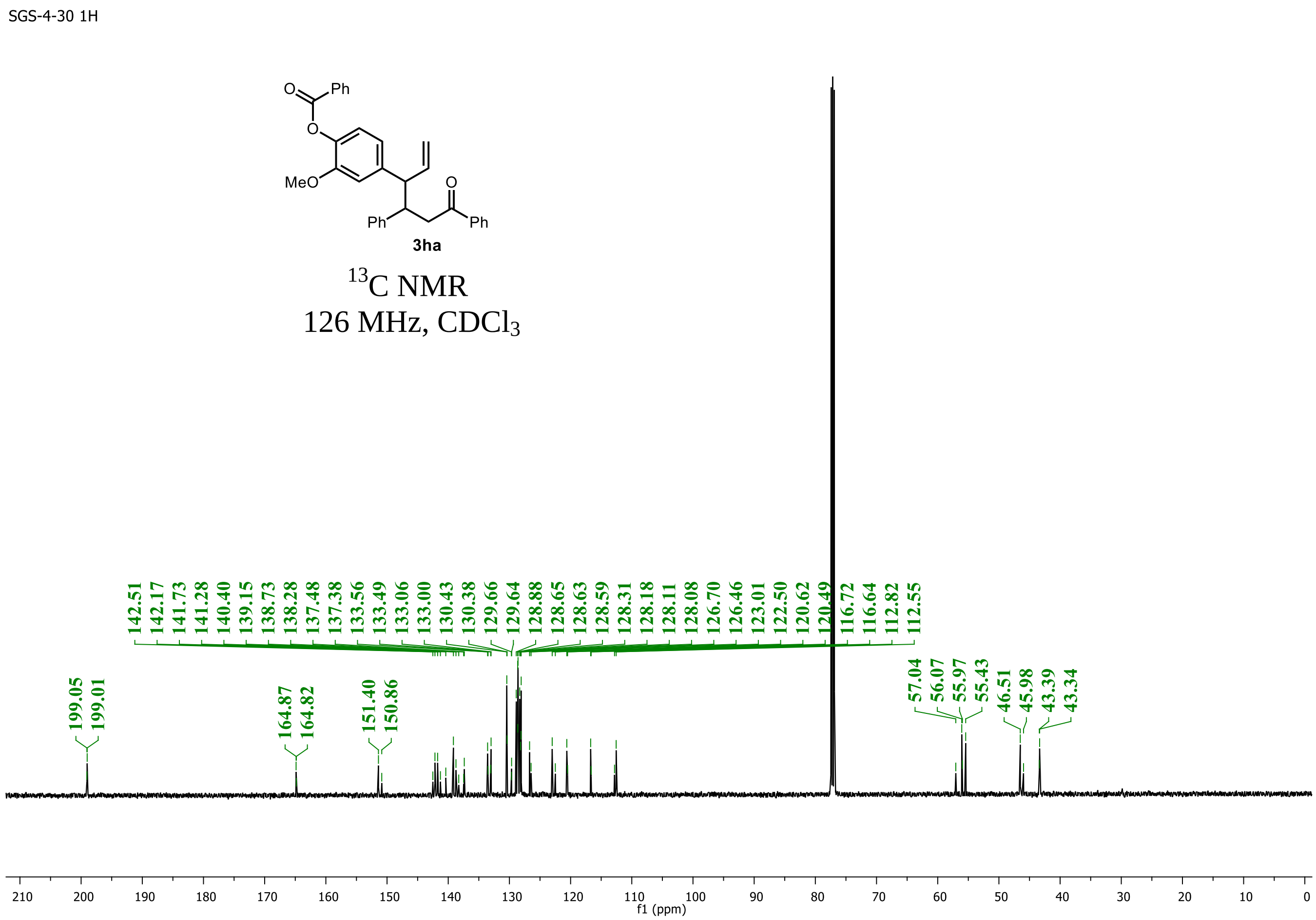
SGS-4-30 1H
Ph
O
O
MeO
O
Ph
Ph
3ha
13C NMR
126 MHz, CDCl3
199.05
199.01
164.87
164.82
151.40
150.86
142.51 142.17 141.73 141.28 140.40 139.15 138.73 138.28 137.48 137.38 133.56 133.49 133.06 133.00 130.43 130.38 129.66 129.64 128.88 128.65 128.63 128.59 128.31 128.18 128.11 128.08 126.70 126.46 123.01 122.50 120.62 120.49 116.72 116.64 112.82 112.55
57.04 56.07 55.97 55.43 46.51 45.98 43.39 43.34
210 200 190 180 170 160 150 140 130 120 110 100 90 80 70 60 50 40 30 20 10 0
f1 (ppm)

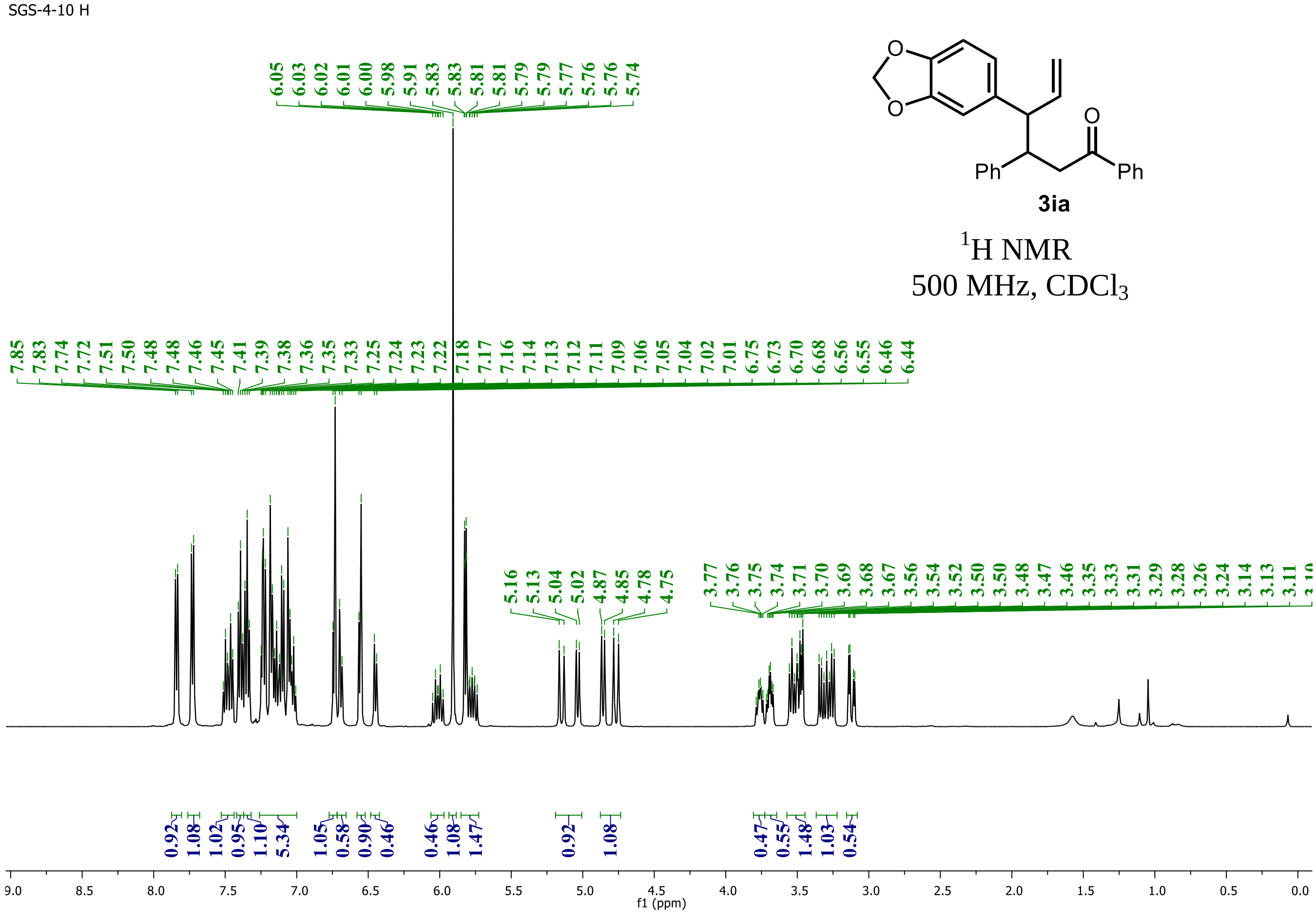
SGS-4-10 H
O
O
O
Ph
Ph
3ia
1H NMR
500 MHz, CDCl3
6.05 6.03 6.02 6.01 6.00 5.98 5.91 5.83 5.83 5.81 5.81 5.79 5.79 5.77 5.76 5.76 5.74
7.85 7.83 7.74 7.72 7.51 7.50 7.48 7.48 7.46 7.45 7.41 7.39 7.38 7.36 7.35 7.33 7.25 7.24 7.23 7.22 7.18 7.17 7.16 7.14 7.13 7.12 7.11 7.09 7.06 7.05 7.04 7.02 7.01 6.75 6.73 6.70 6.68 6.56 6.55 6.46 6.44
5.16 5.13 5.04 5.02 4.87 4.85 4.78 4.75
3.77 3.76 3.75 3.74 3.71 3.70 3.69 3.68 3.67 3.56 3.54 3.52 3.50 3.50 3.48 3.47 3.46 3.35 3.33 3.31 3.29 3.28 3.26 3.24 3.14 3.13 3.11
0.92 1.08 1.02 0.95 1.10 5.34 1.05 0.58 0.90 0.46 0.46 1.08 1.47 0.92 1.08 0.47 0.55 1.48 1.03 0.54
9.0 8.5 8.0 7.5 7.0 6.5 6.0 5.5 5.0 4.5 4.0 3.5 3.0 2.5 2.0 1.5 1.0 0.5 0.0
f1 (ppm)

SGS-4-10 C

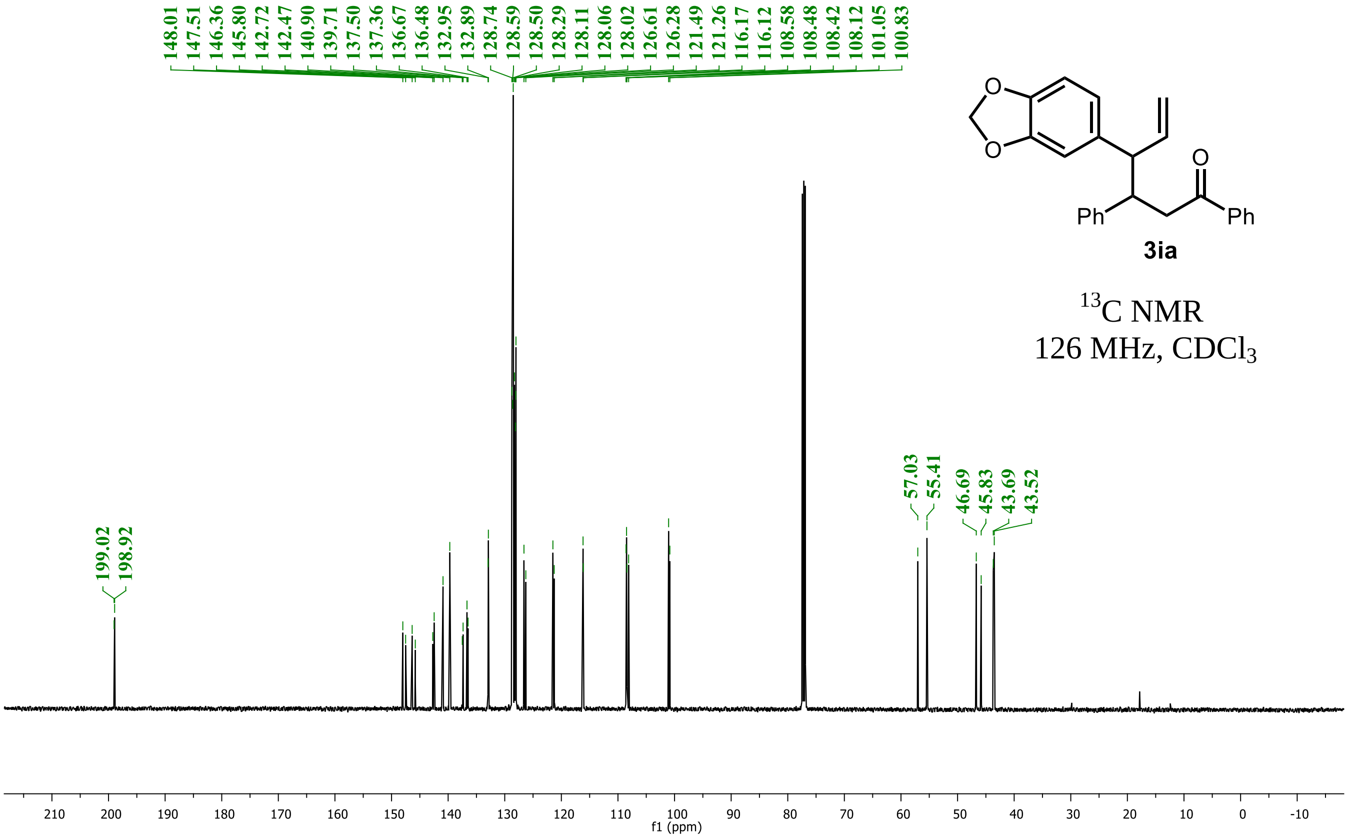

SGS-3-184-4 1H

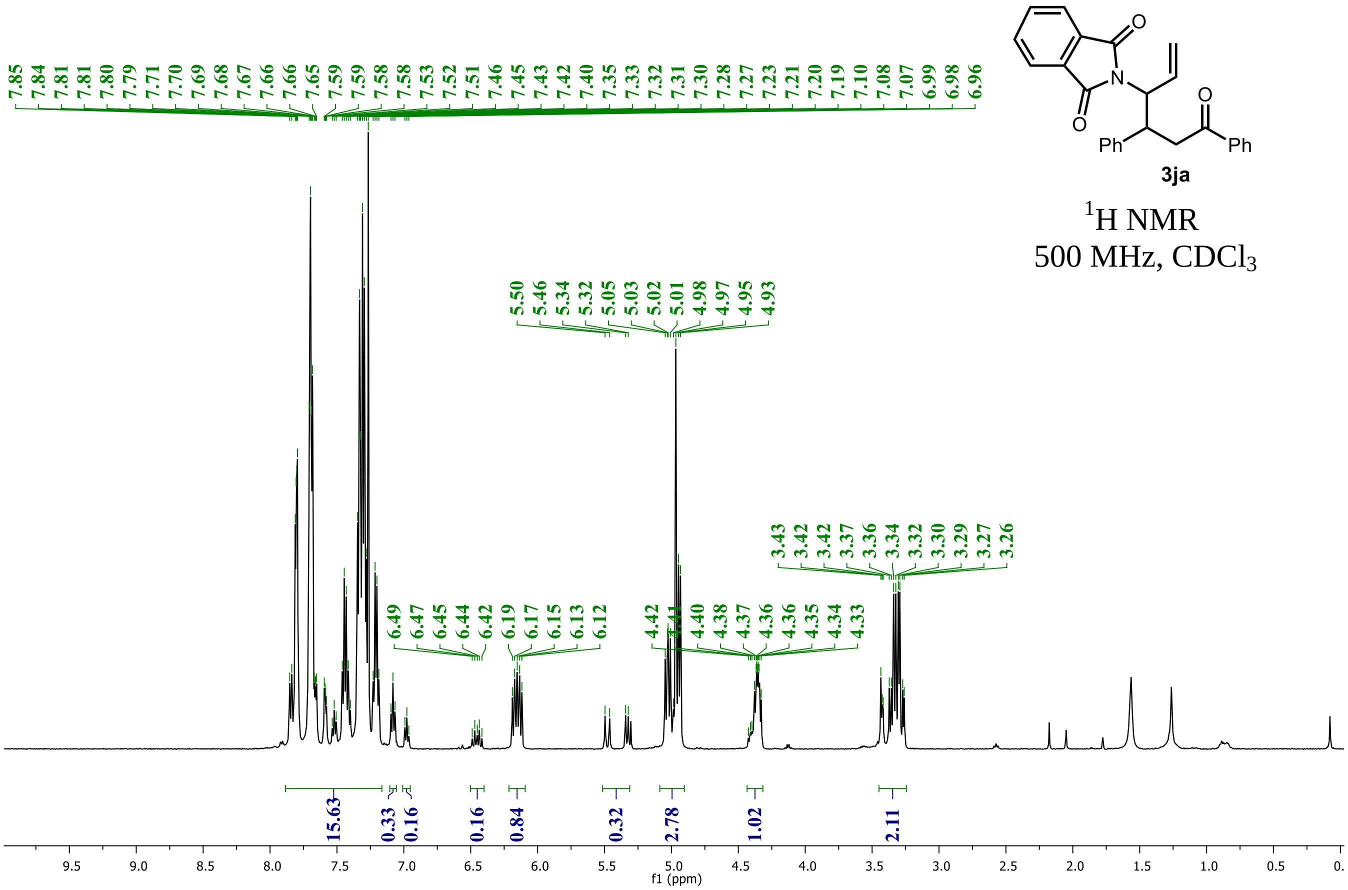

SGS-3-184-4 C

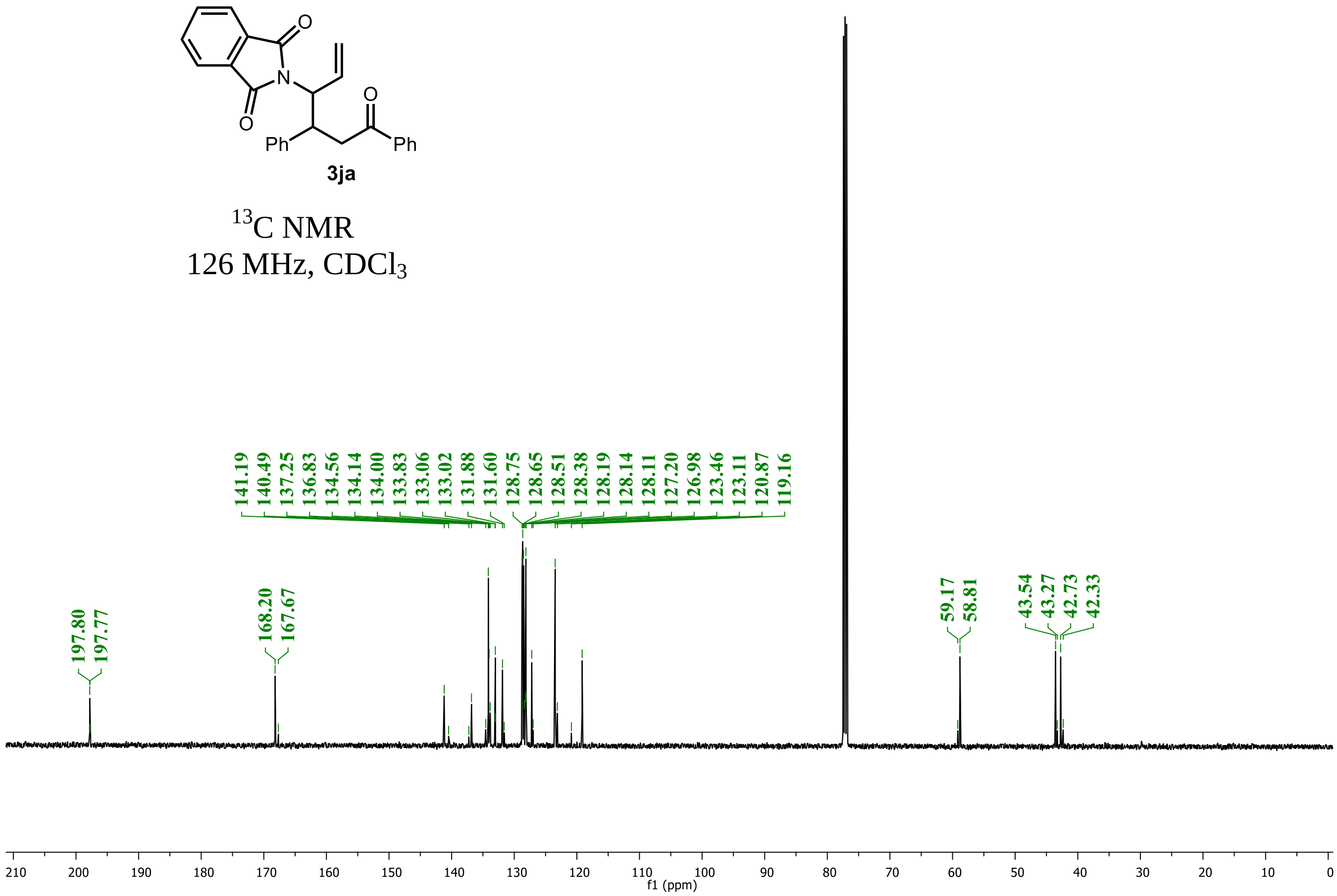

SGS-4-24 H

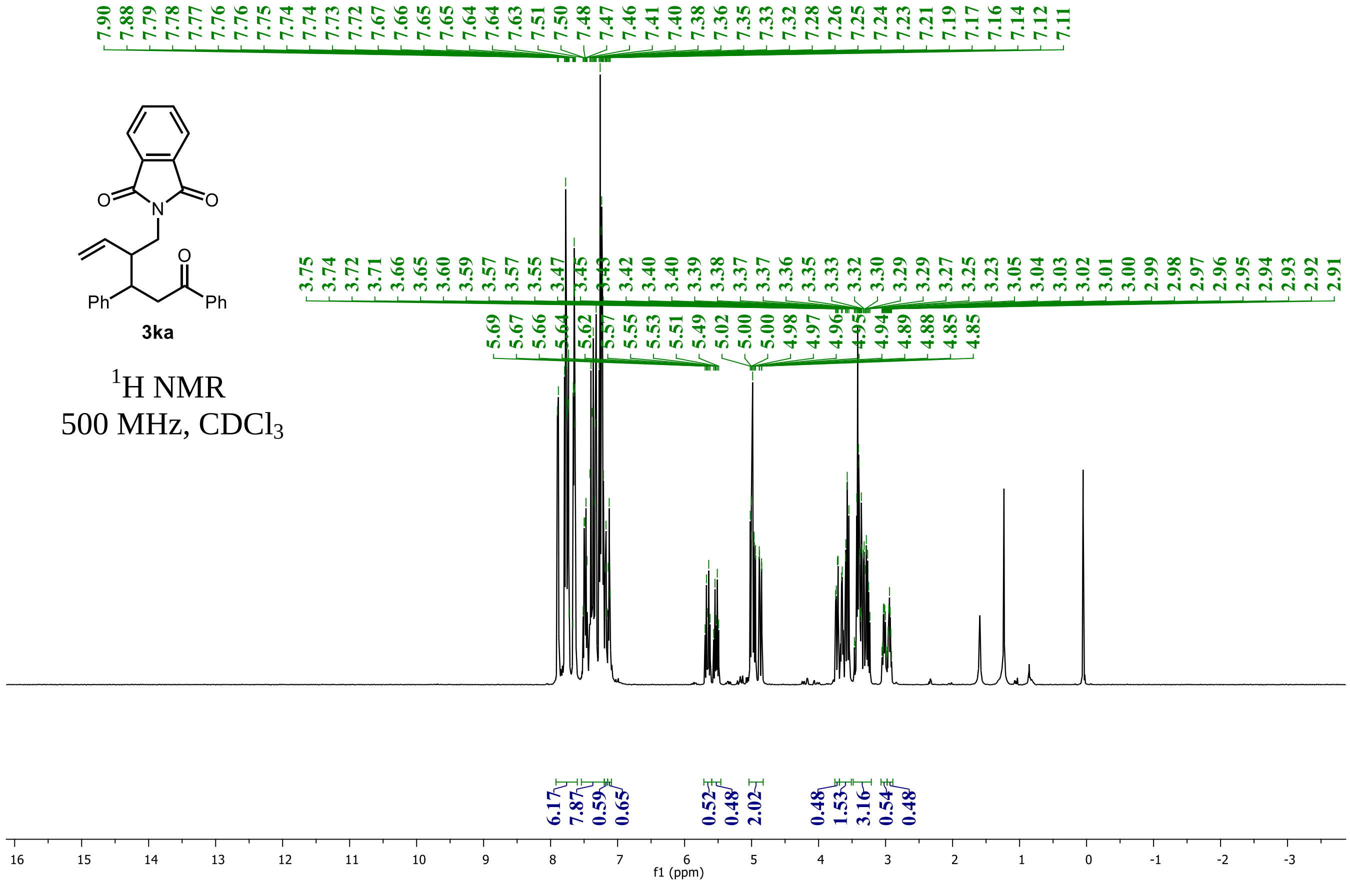

SGS-4-24 C

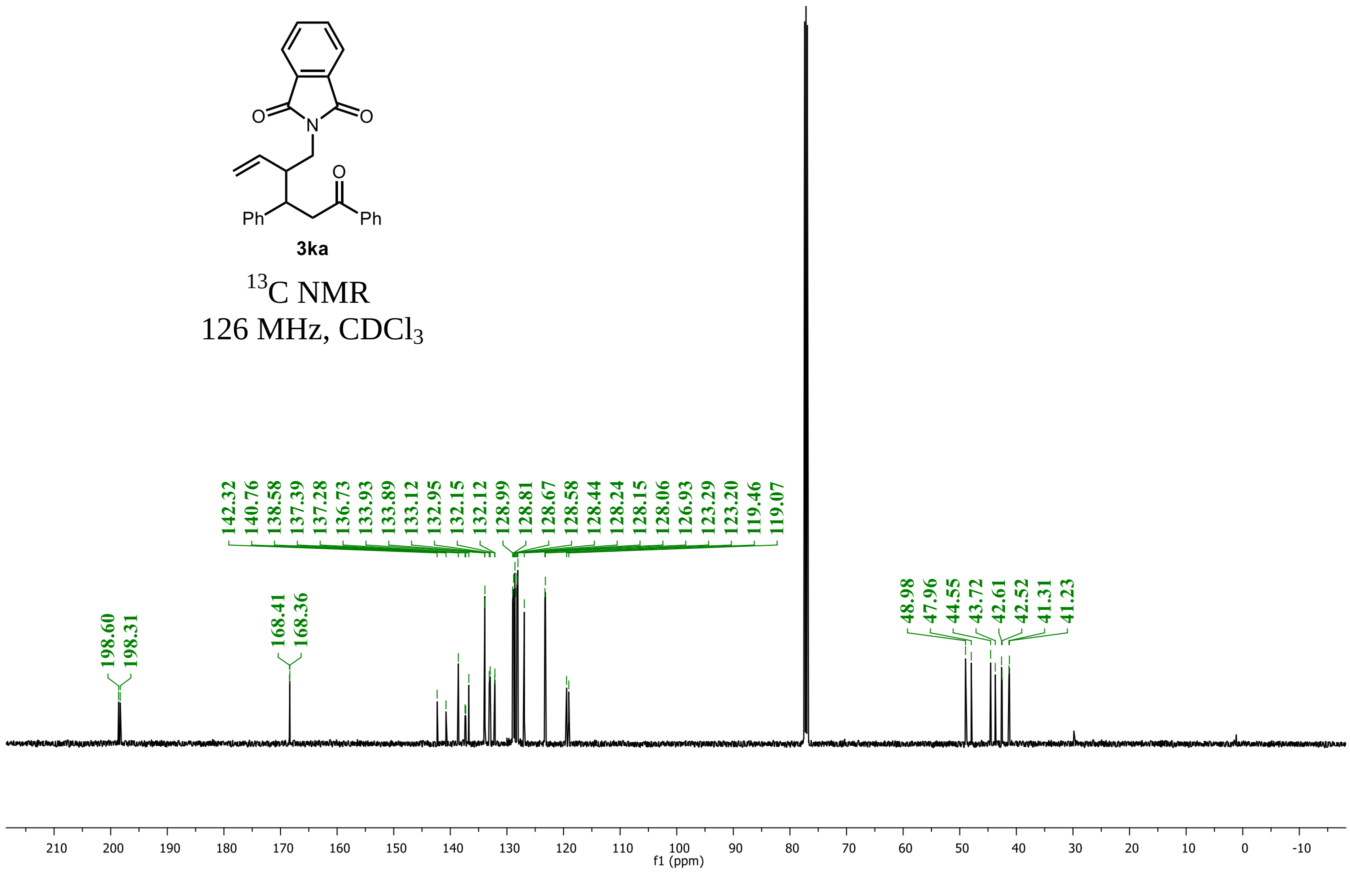

SGS-4-18 H

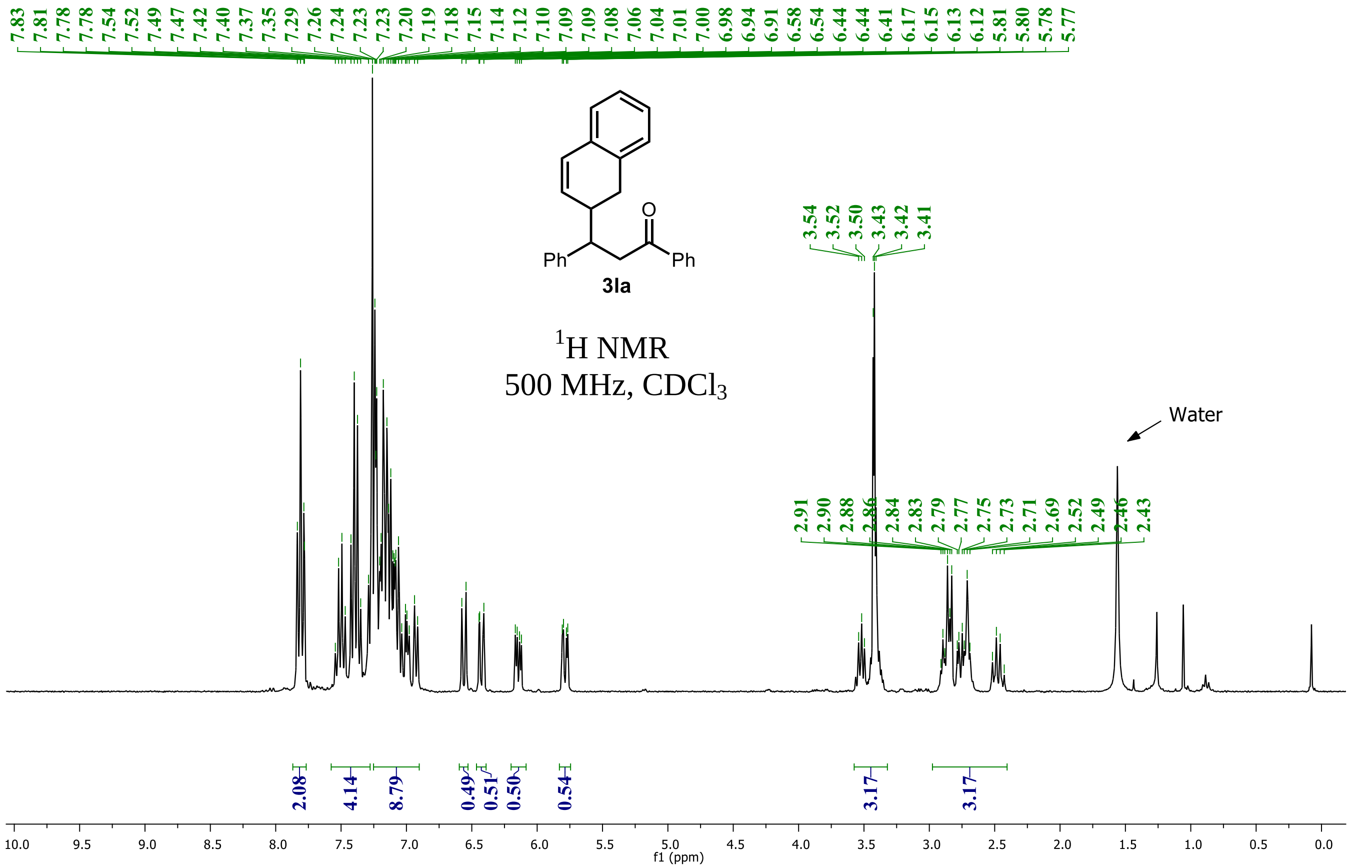

SGS-4-18 C

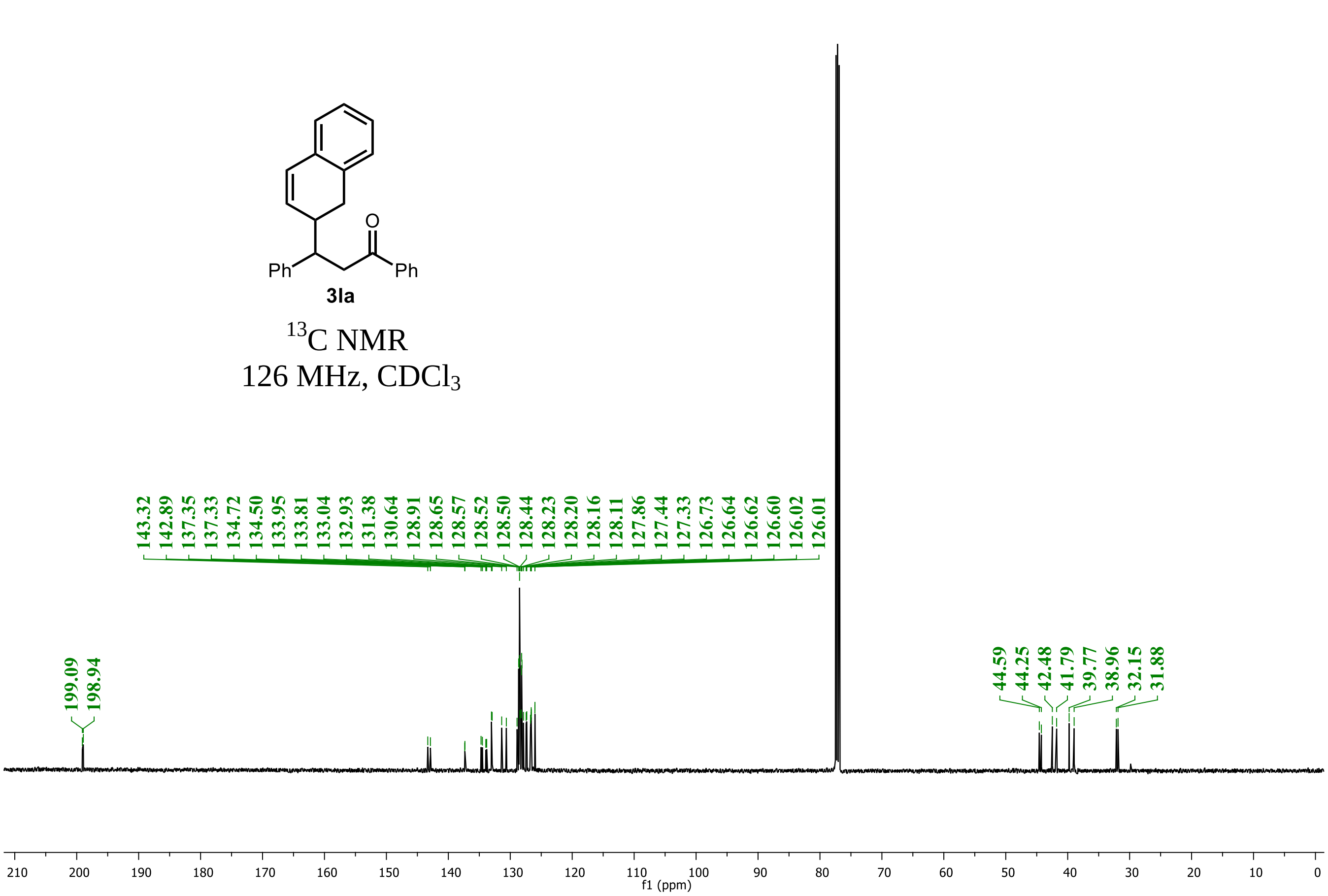

SGS-4-16 H

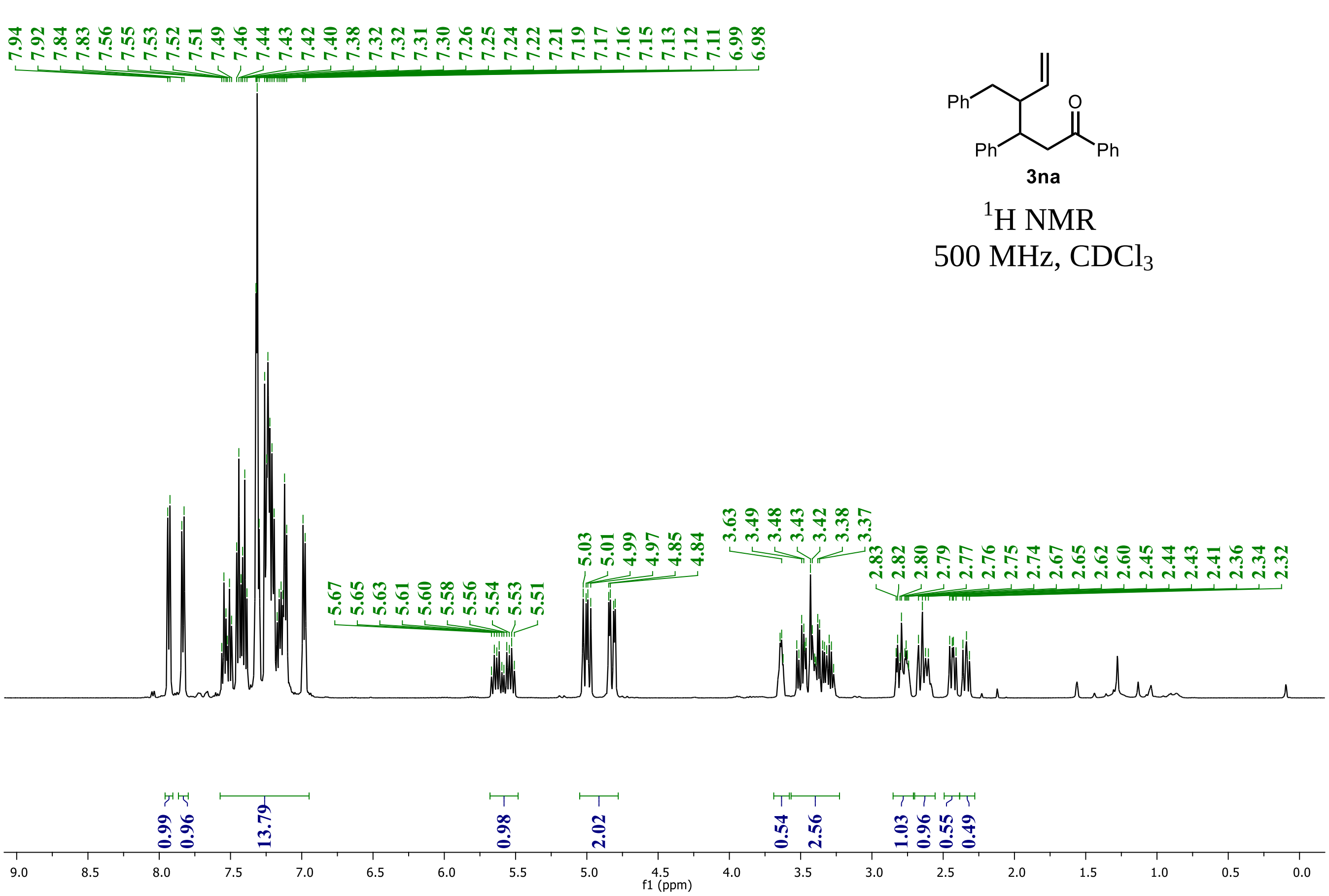

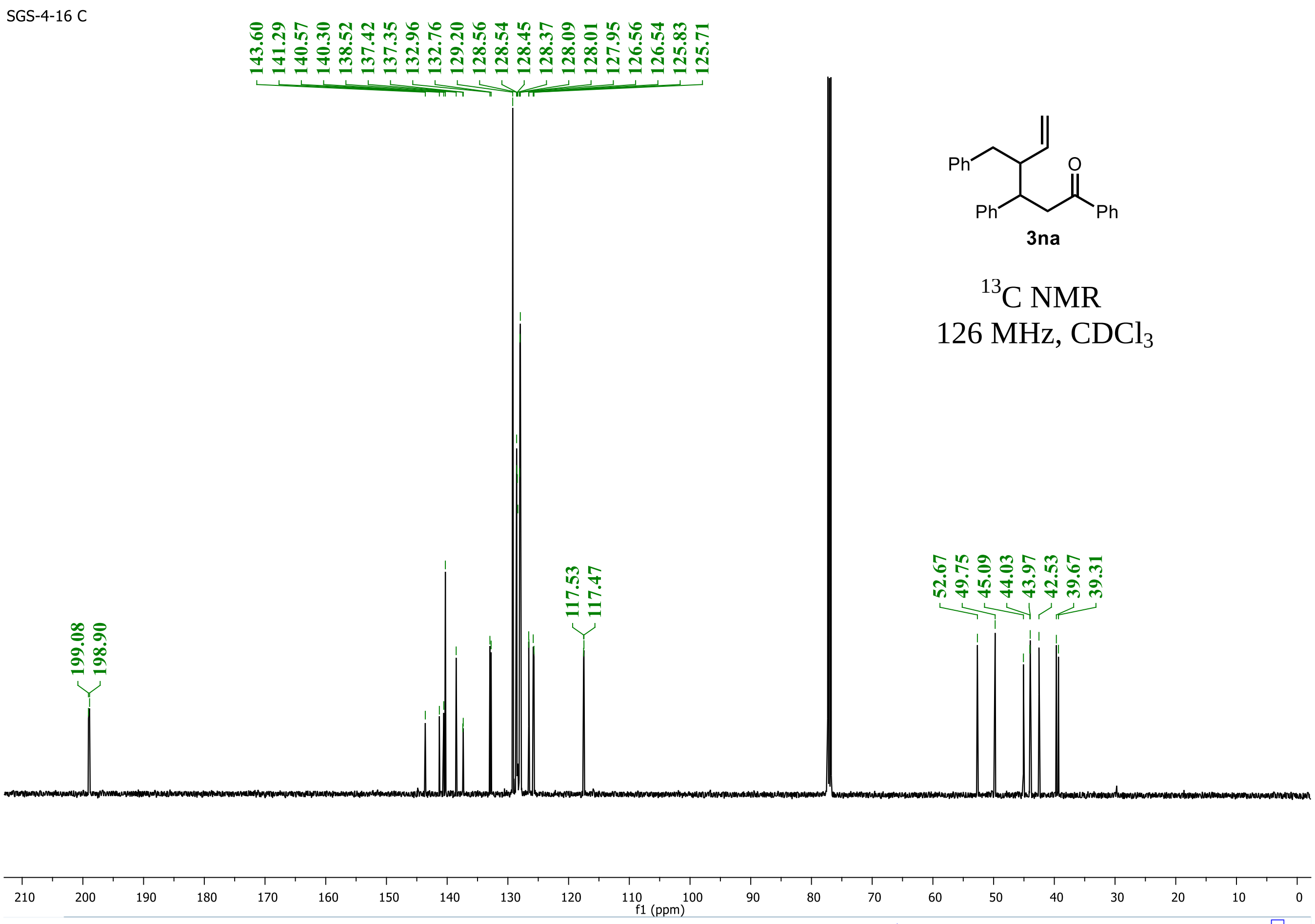
SGS-4-16 C
143.60
141.29
140.57
140.30
138.52
137.42
137.35
132.96
132.76
129.20
128.56
128.54
128.45
128.37
128.09
128.01
127.95
126.56
126.54
125.83
125.71
117.53
117.47
199.08
198.90
52.67
49.75
45.09
44.03
43.97
42.53
39.67
39.31
Ph
O
Ph
Ph
3na
^{13}C NMR
126 MHz, $CDCl_3$
f1 (ppm)

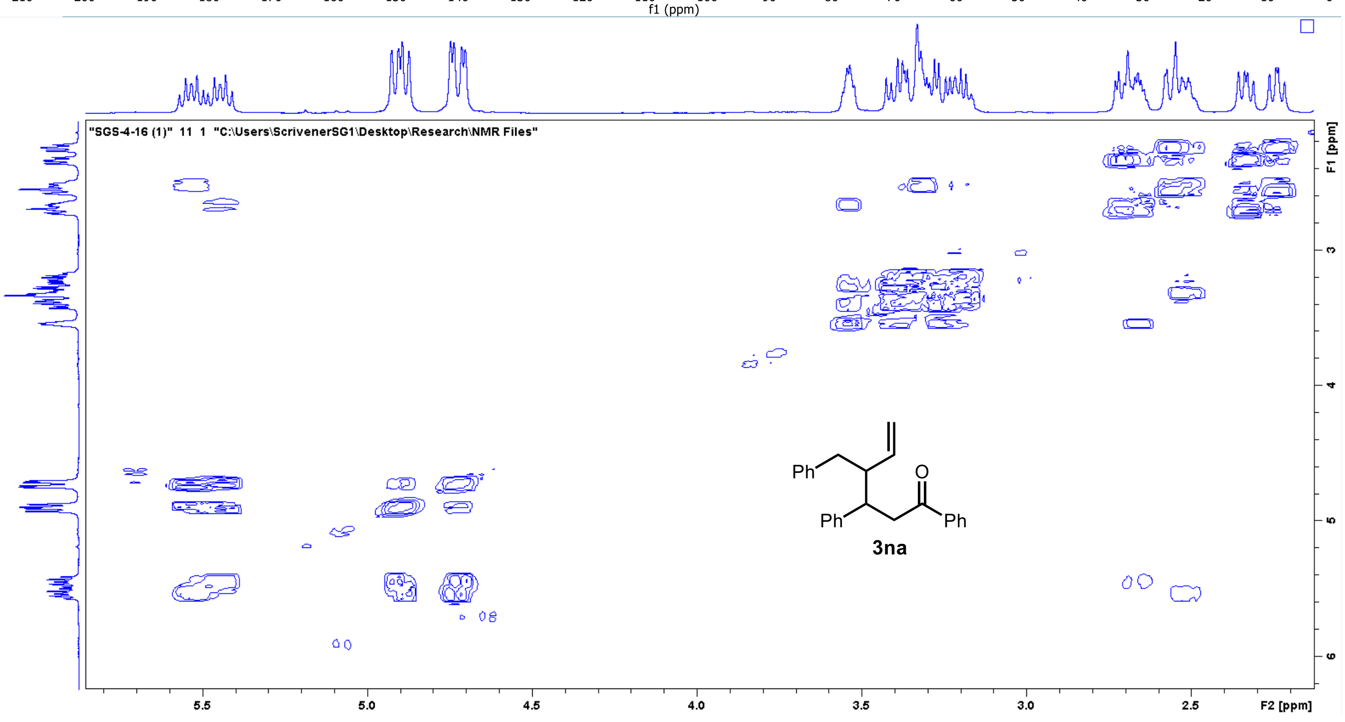
"SGS-4-16 (1)" 11 1 "C:\Users\ScrivenerSG1\Desktop\Research\NMR Files"
F1 [ppm]
F2 [ppm]
Ph
O
Ph
Ph
3na

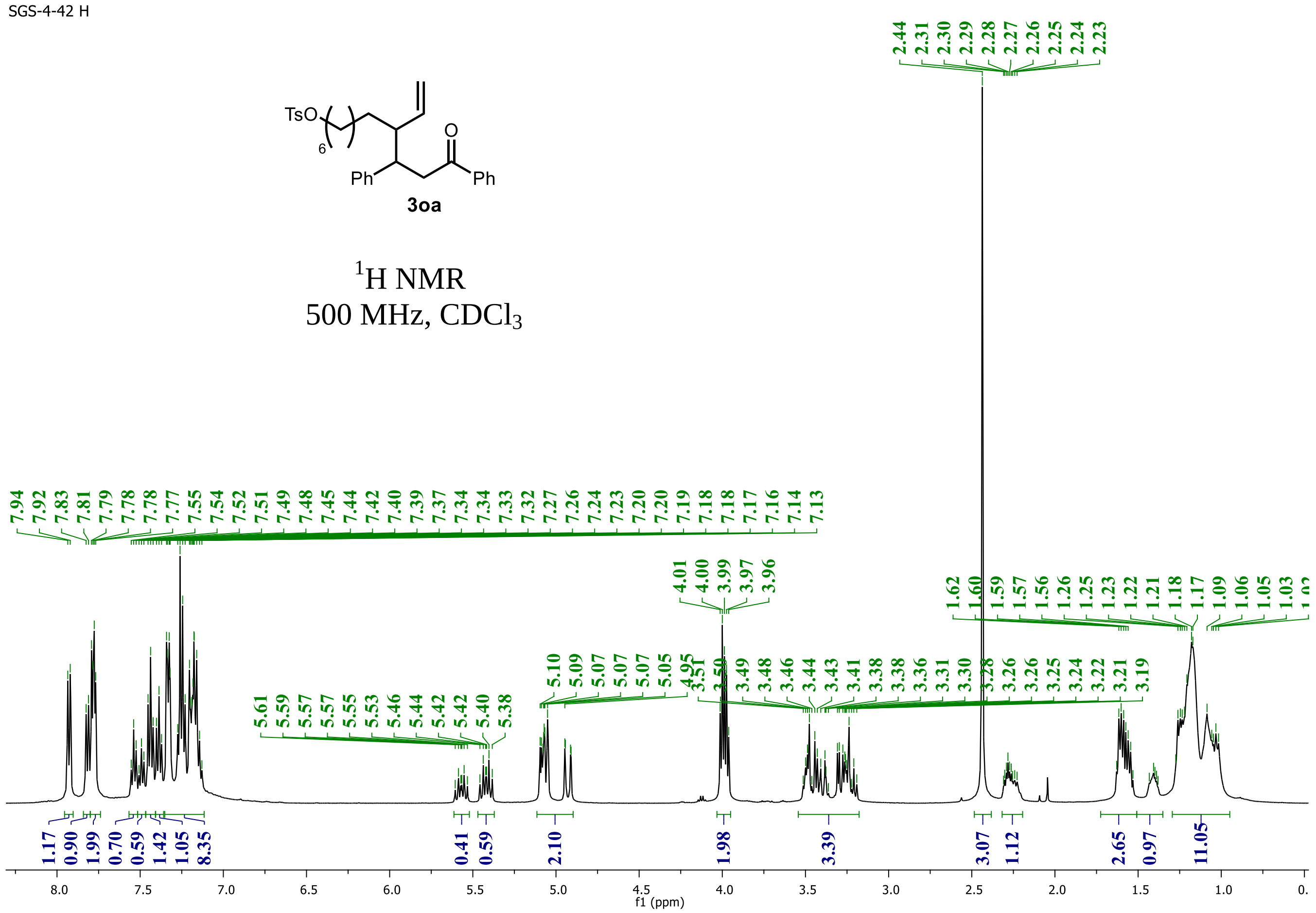
SGS-4-42 H
TsO
6
O
Ph
Ph
3oa
1H NMR
500 MHz, CDCl3
f1 (ppm)

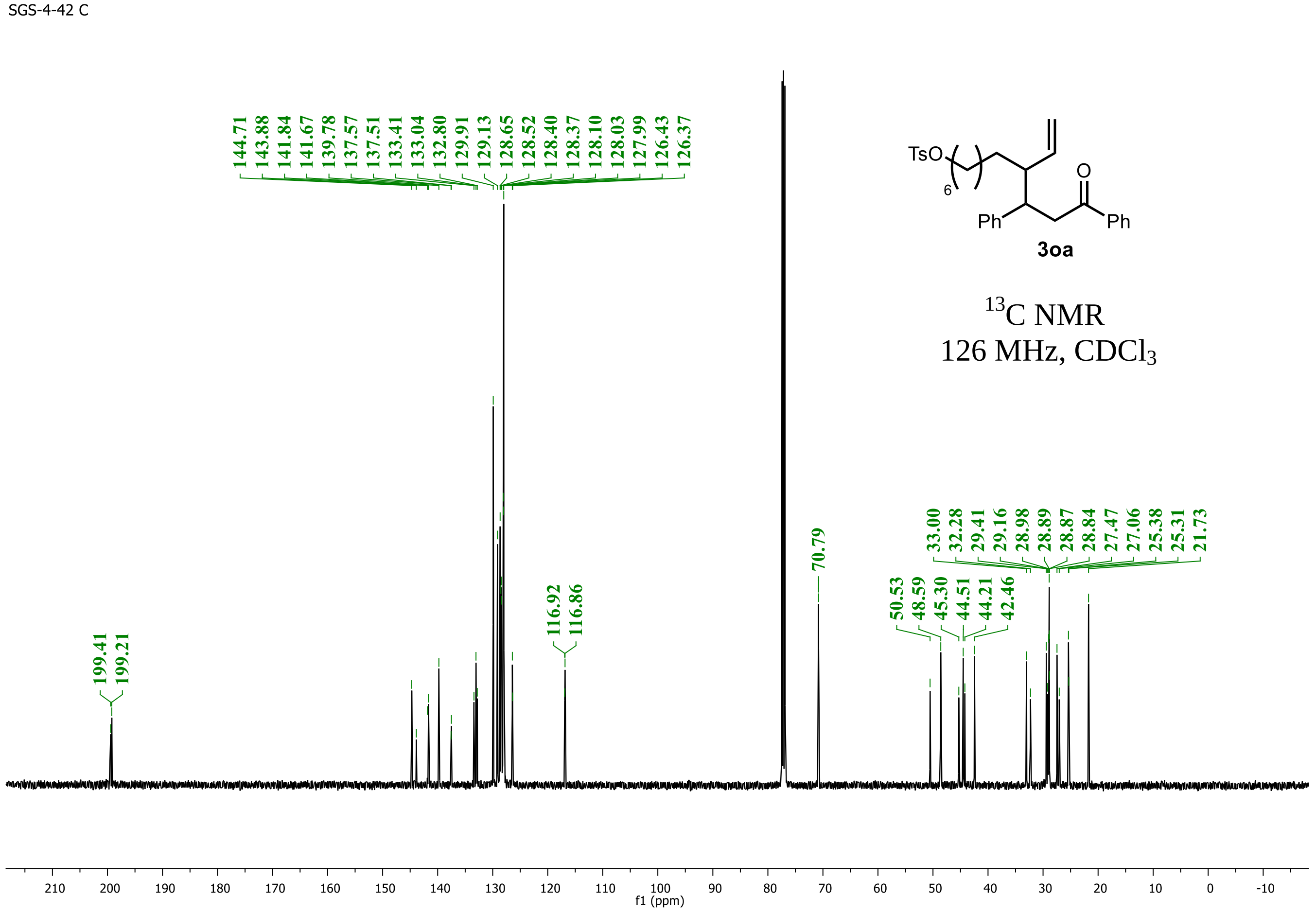
SGS-4-42 C
TsO
6
O
Ph
Ph
3oa
13C NMR
126 MHz, CDCl3
f1 (ppm)

SGS-4-40 H

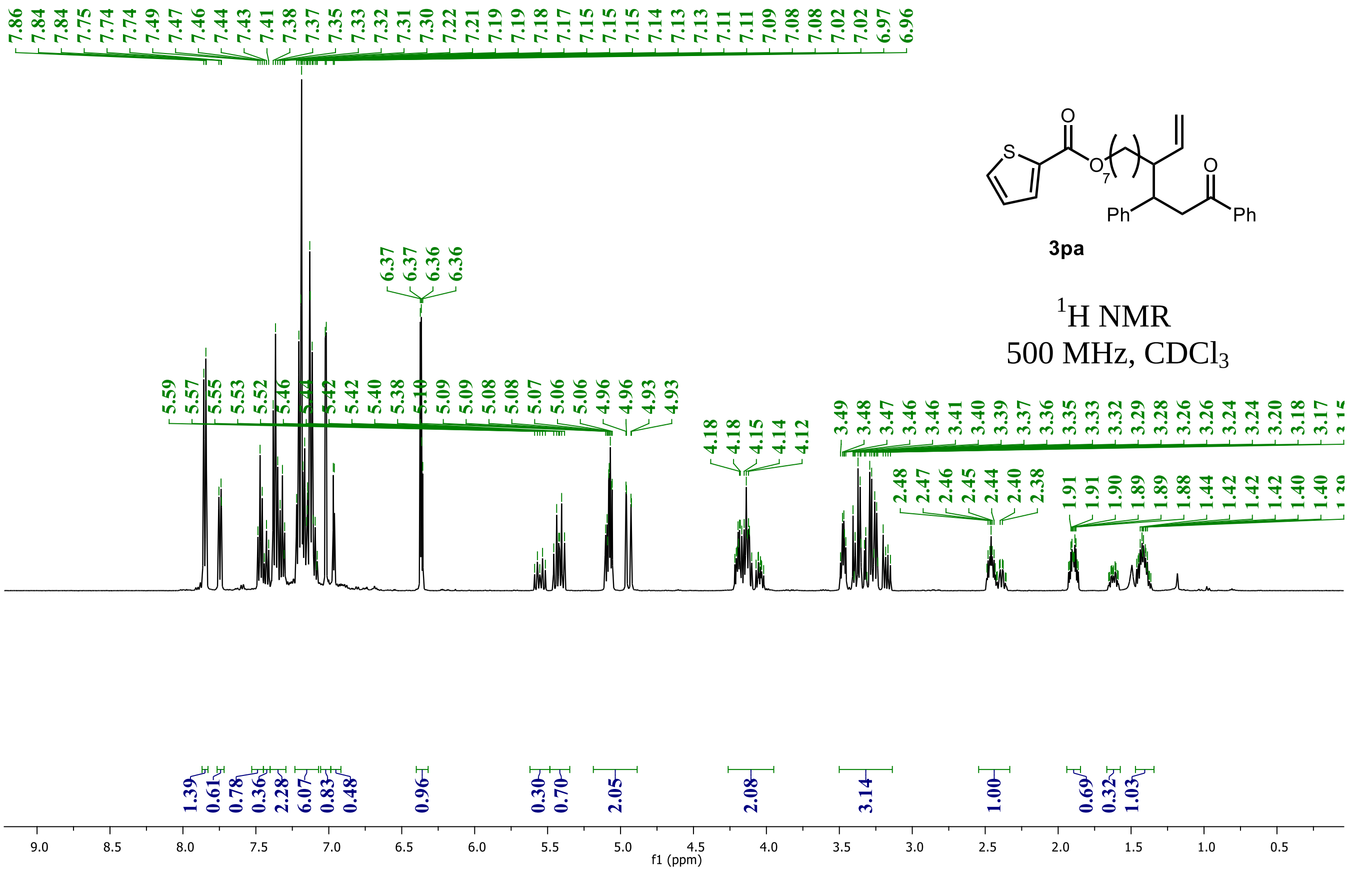

SGS-4-40 C

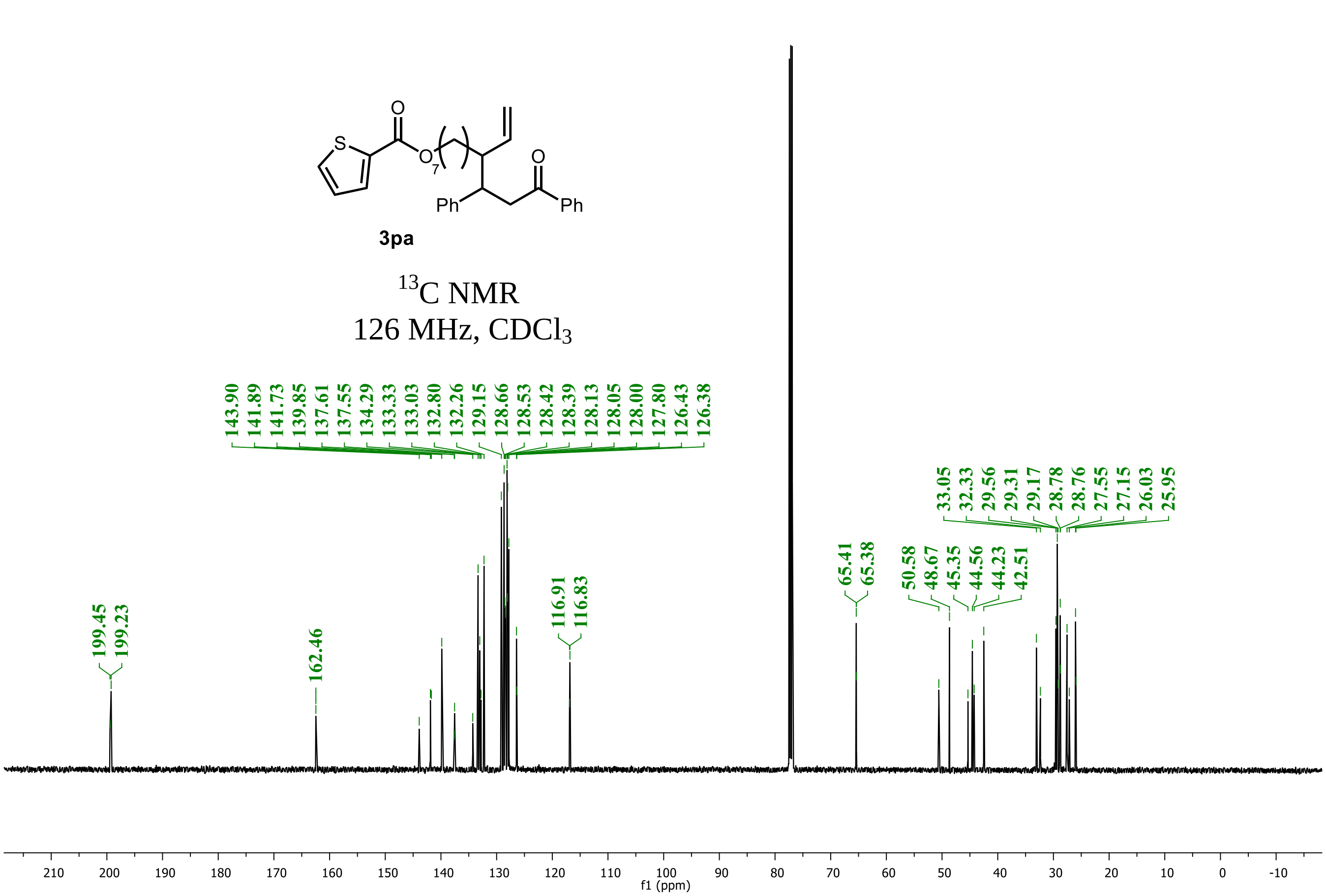

SGS-4-41 H

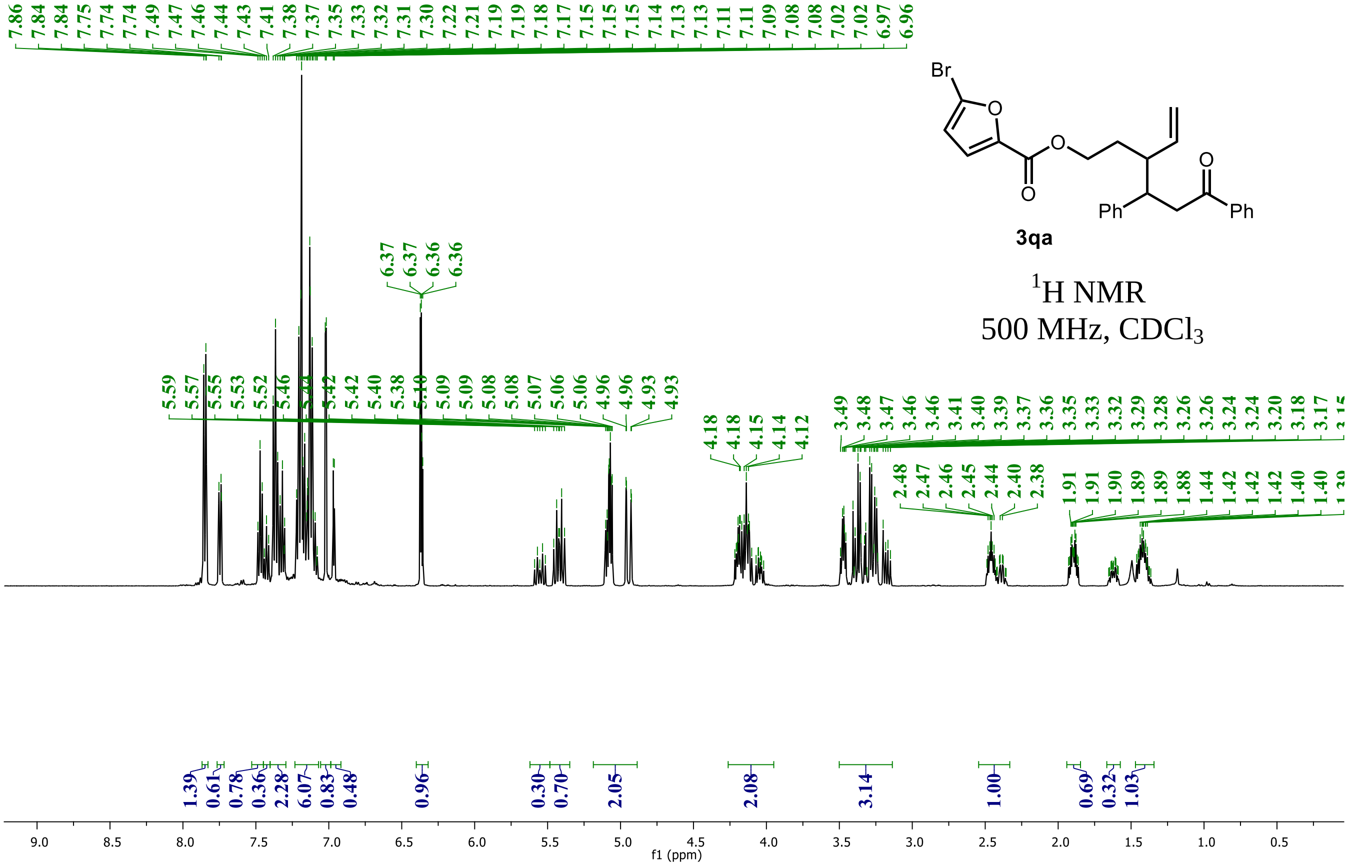

SGS-4-41 C

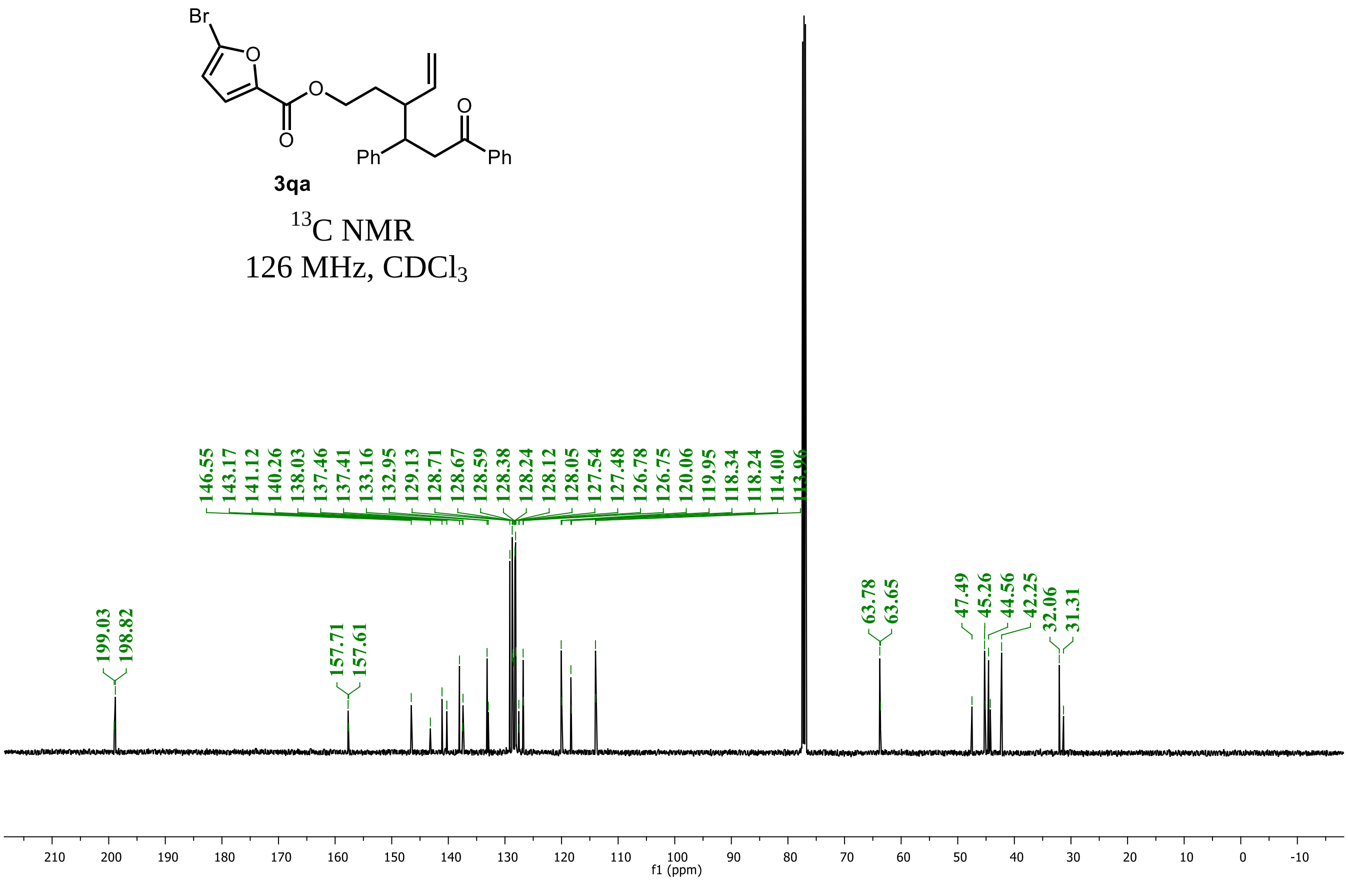

SGS-4-39 H

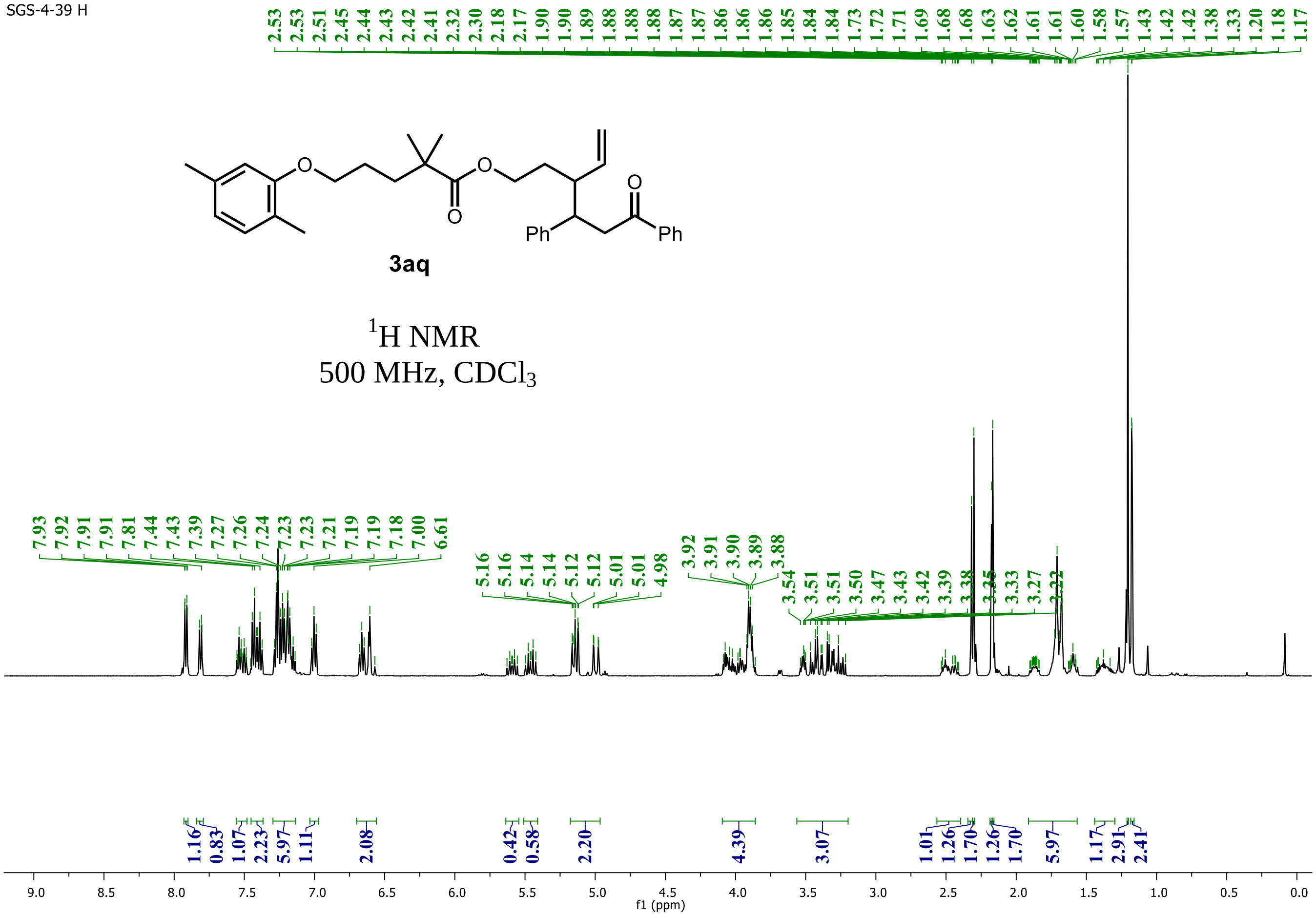

SGS-4-39 C

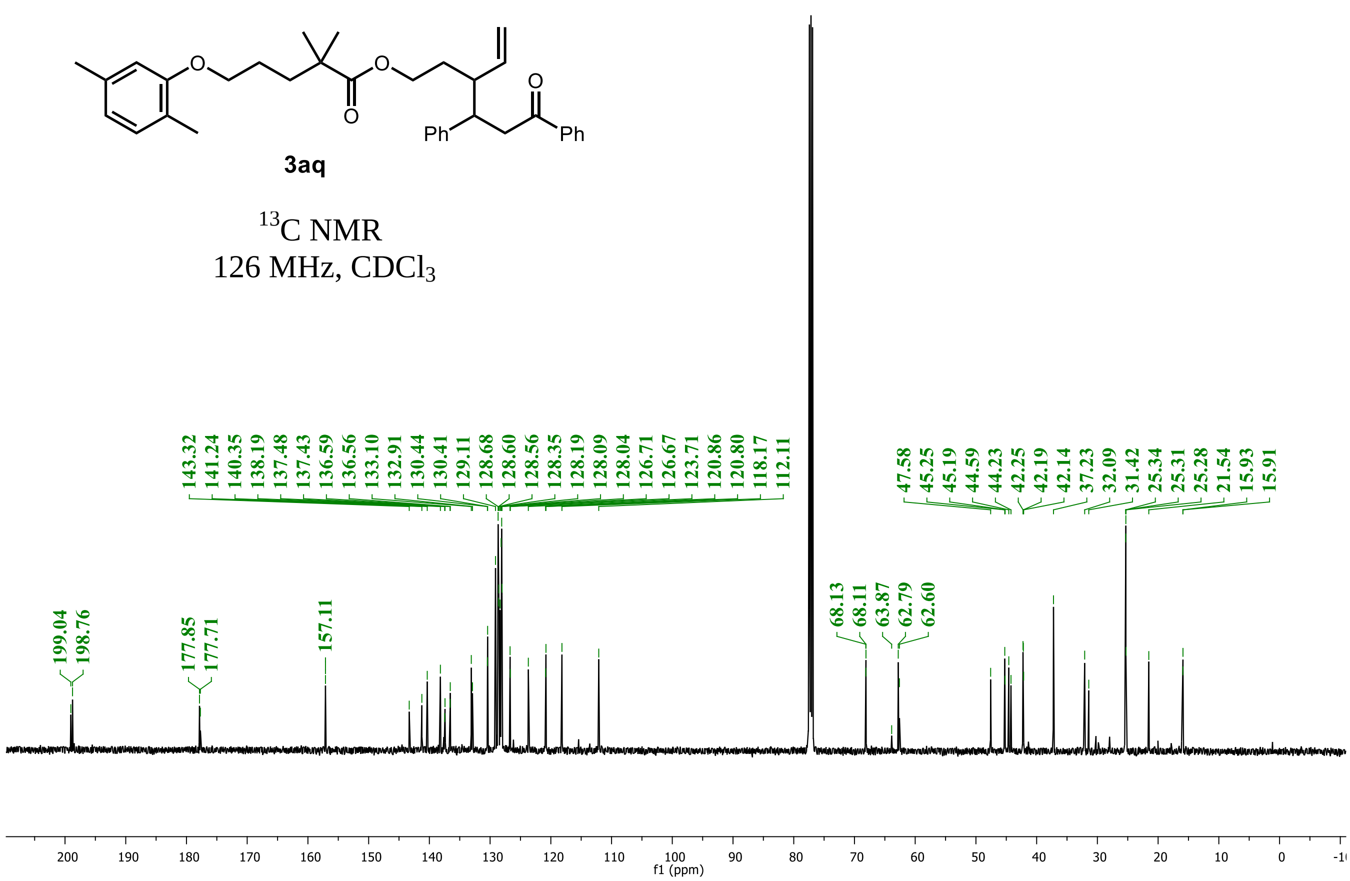

SGS-4-36 H

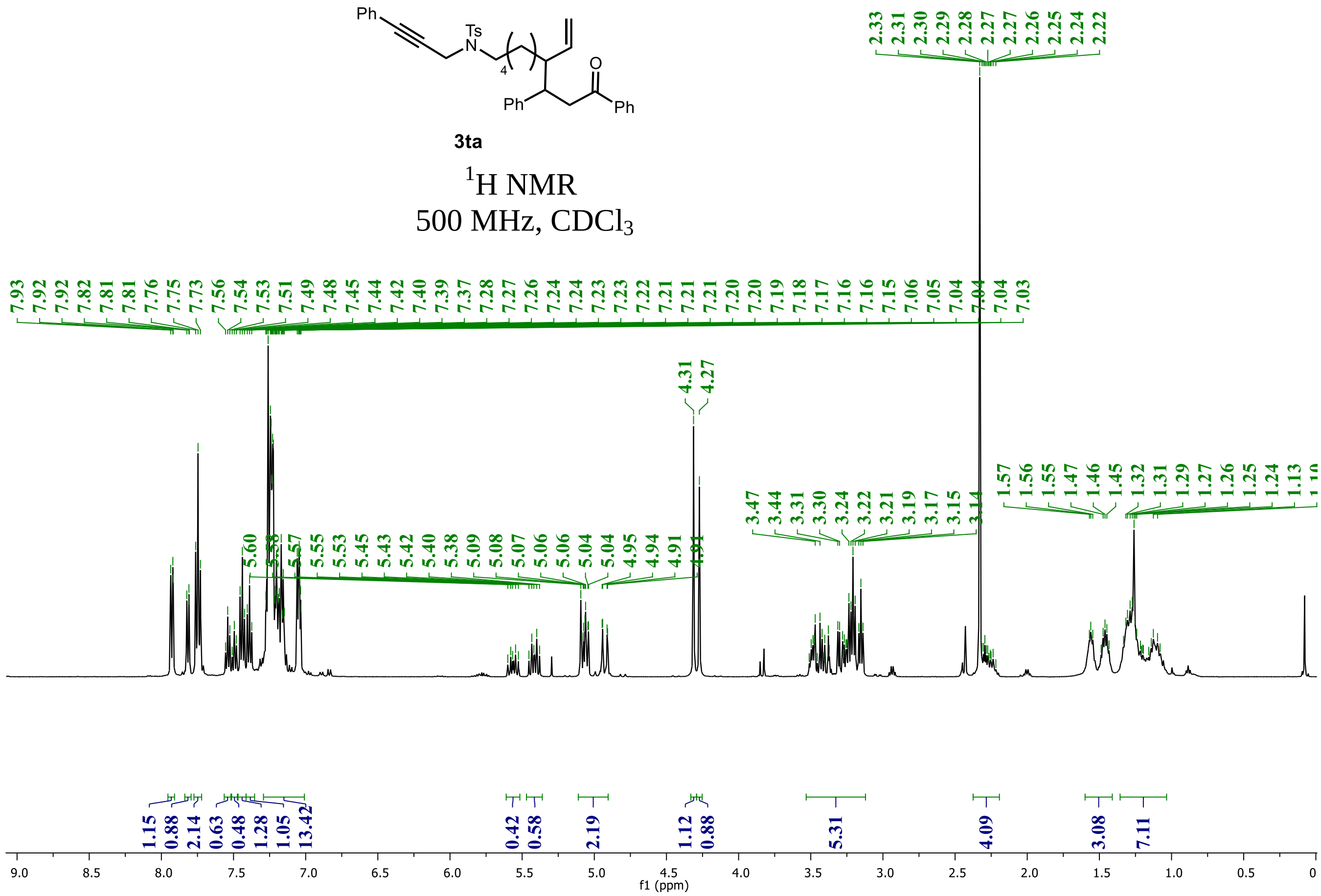

SGS-4-36 C

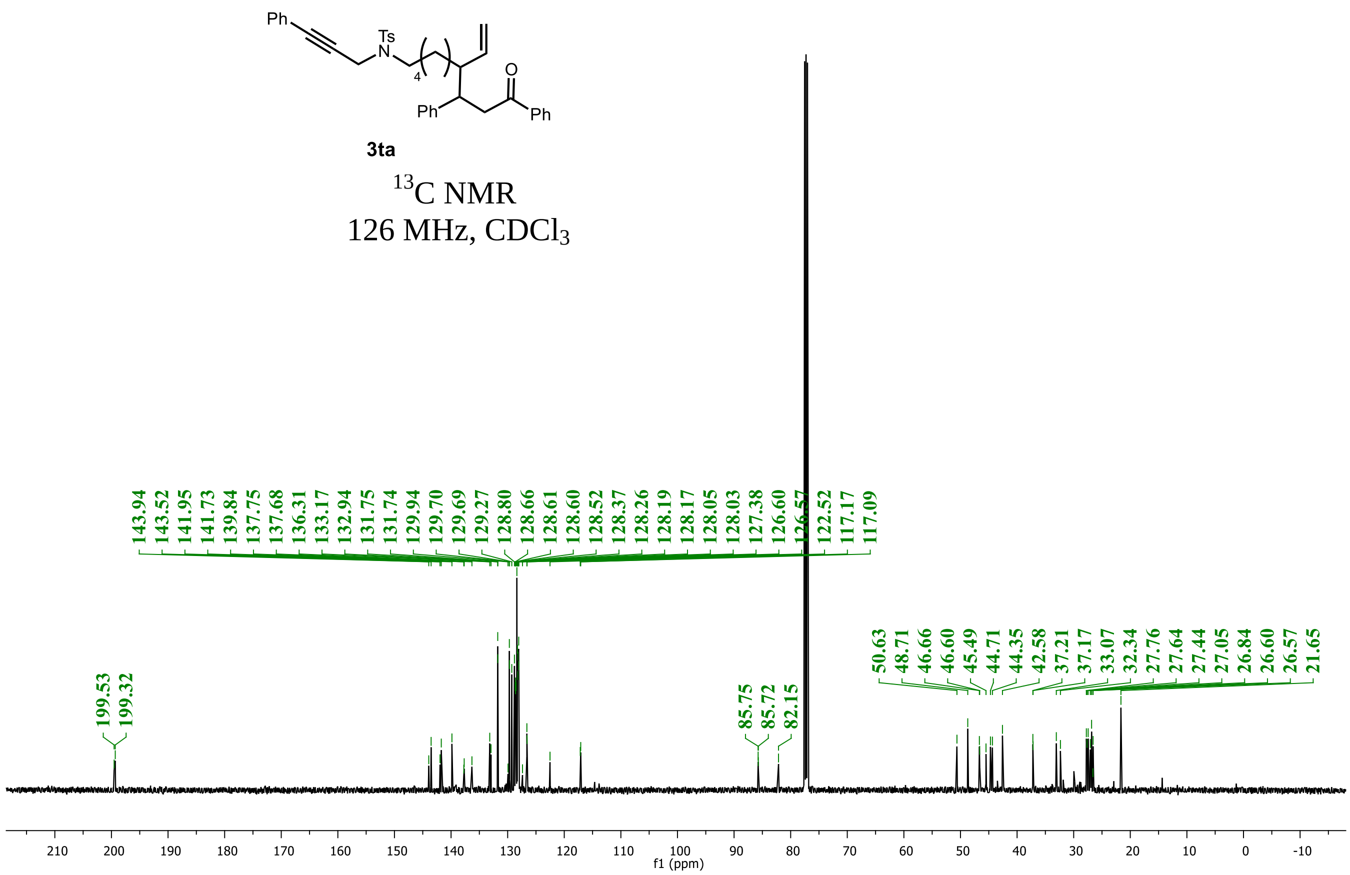

SGS-4-76-1 H

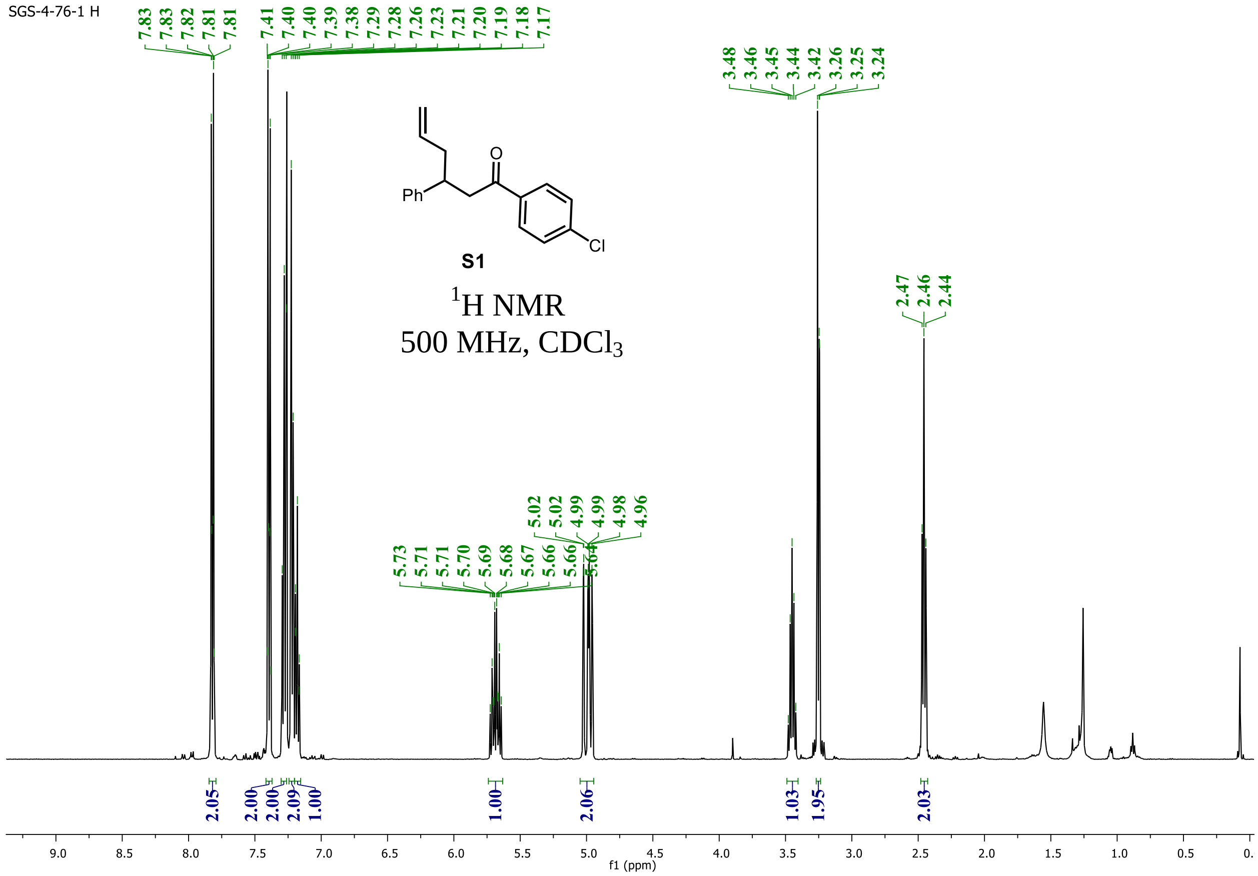

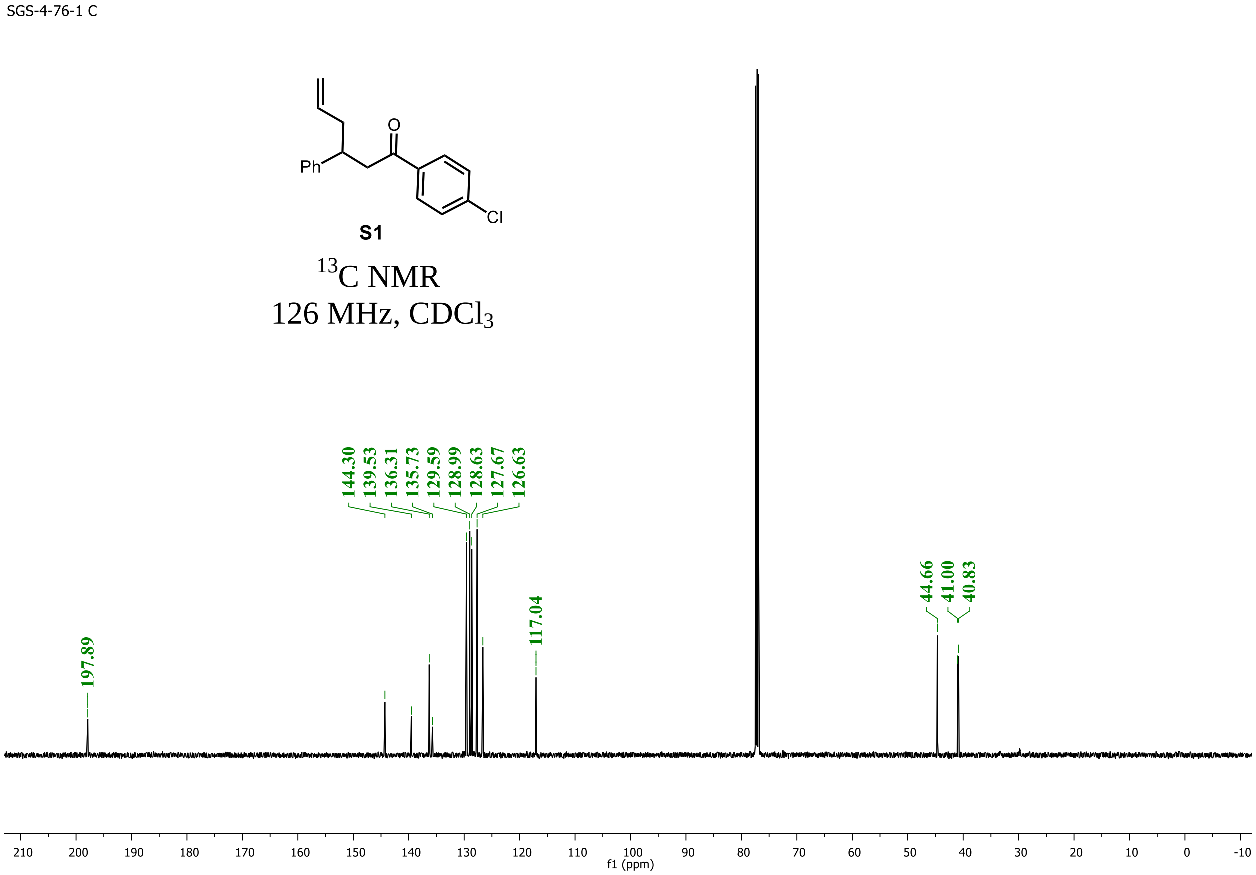
SGS-4-76-1 C
O
Ph
Cl
S1
^{13}C NMR
126 MHz, $CDCl_3$
144.30
139.53
136.31
135.73
129.59
128.99
128.63
127.67
126.63
117.04
197.89
44.66
41.00
40.83
f1 (ppm)

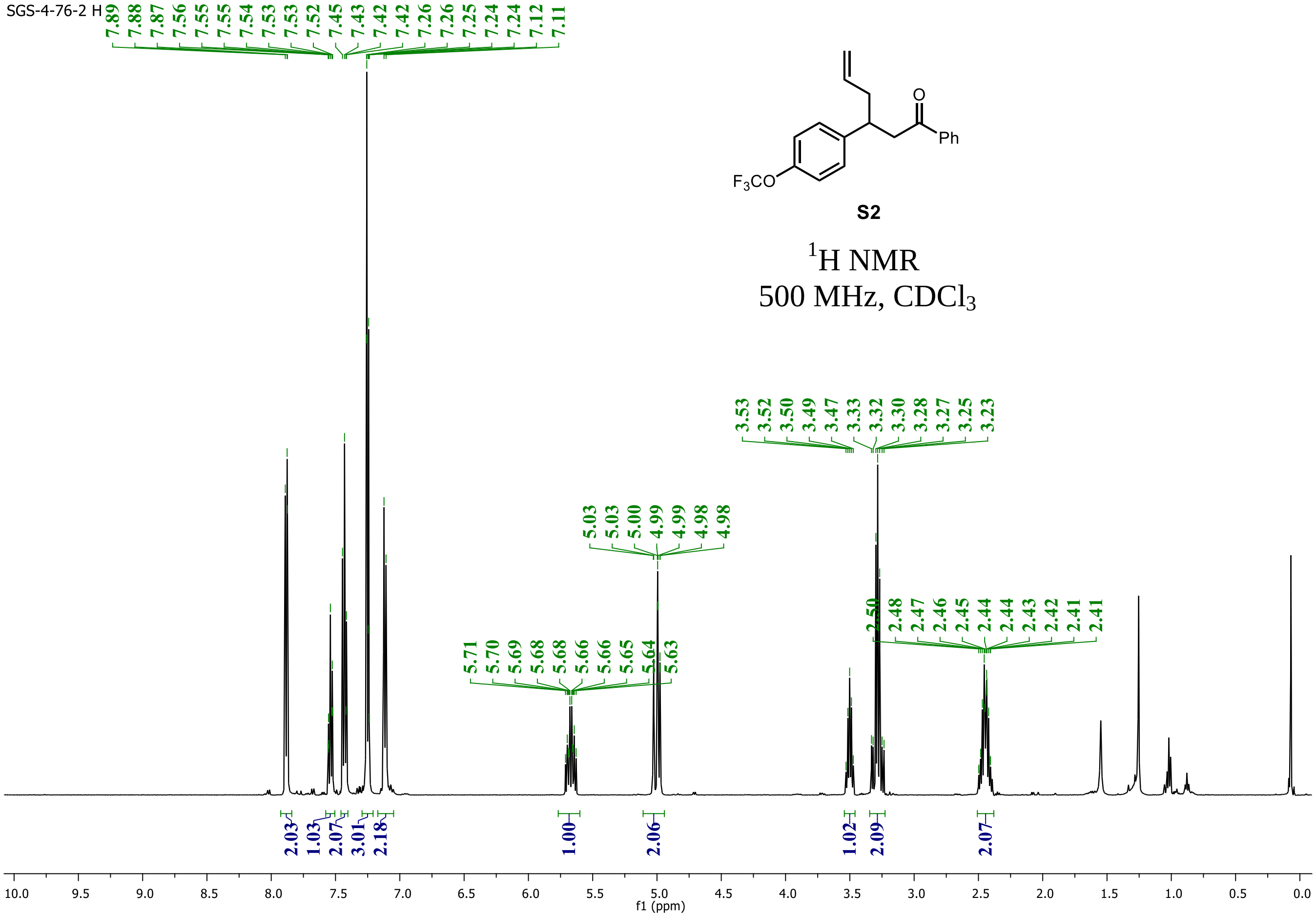
SGS-4-76-2 H
7.89 7.88 7.87 7.56 7.55 7.55 7.54 7.53 7.53 7.52 7.45 7.43 7.42 7.42 7.26 7.26 7.25 7.24 7.24 7.12 7.11
O
Ph
F_3CO
S2
^{1}H NMR
500 MHz, $CDCl_3$
3.53 3.52 3.50 3.49 3.47 3.33 3.32 3.30 3.28 3.27 3.25 3.23
5.03 5.03 5.00 4.99 4.99 4.98 4.98
2.50 2.48 2.47 2.46 2.45 2.44 2.44 2.43 2.42 2.41 2.41
5.71 5.70 5.69 5.68 5.68 5.66 5.66 5.65 5.64 5.63
2.03 1.03 2.07 3.01 2.18 1.00 2.06 1.02 2.09 2.07
f1 (ppm)

SGS-4-76-2 C

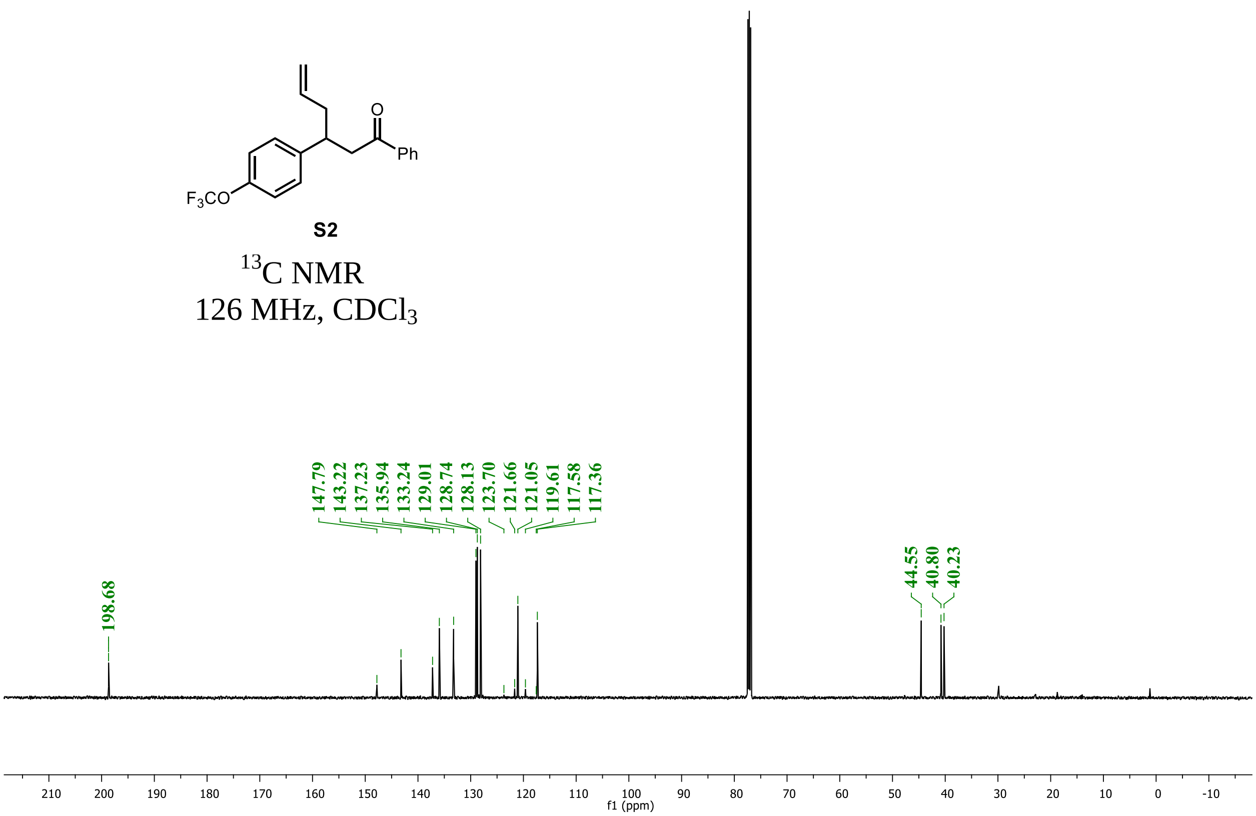

SGS-4-76-2 F

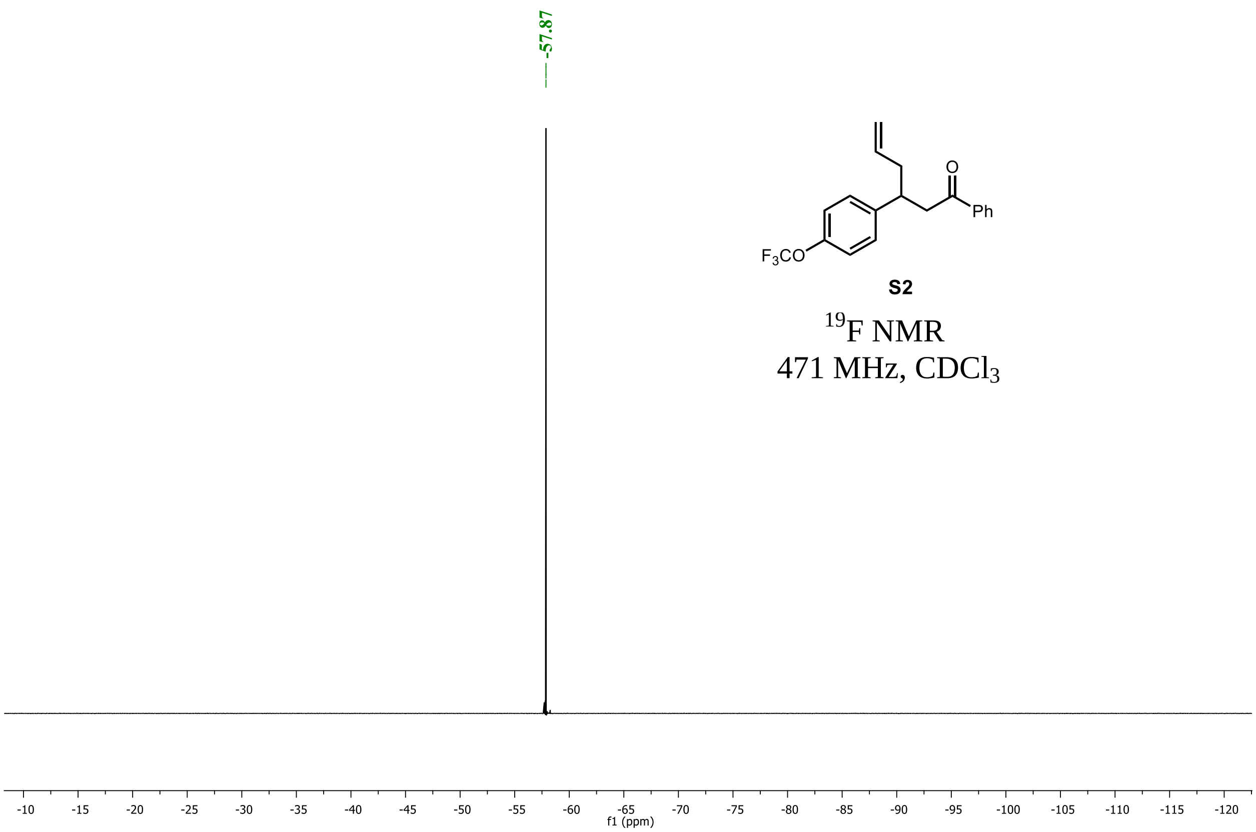

Copies of NMR spectra of products: Large scale reaction and synthetic applications of products

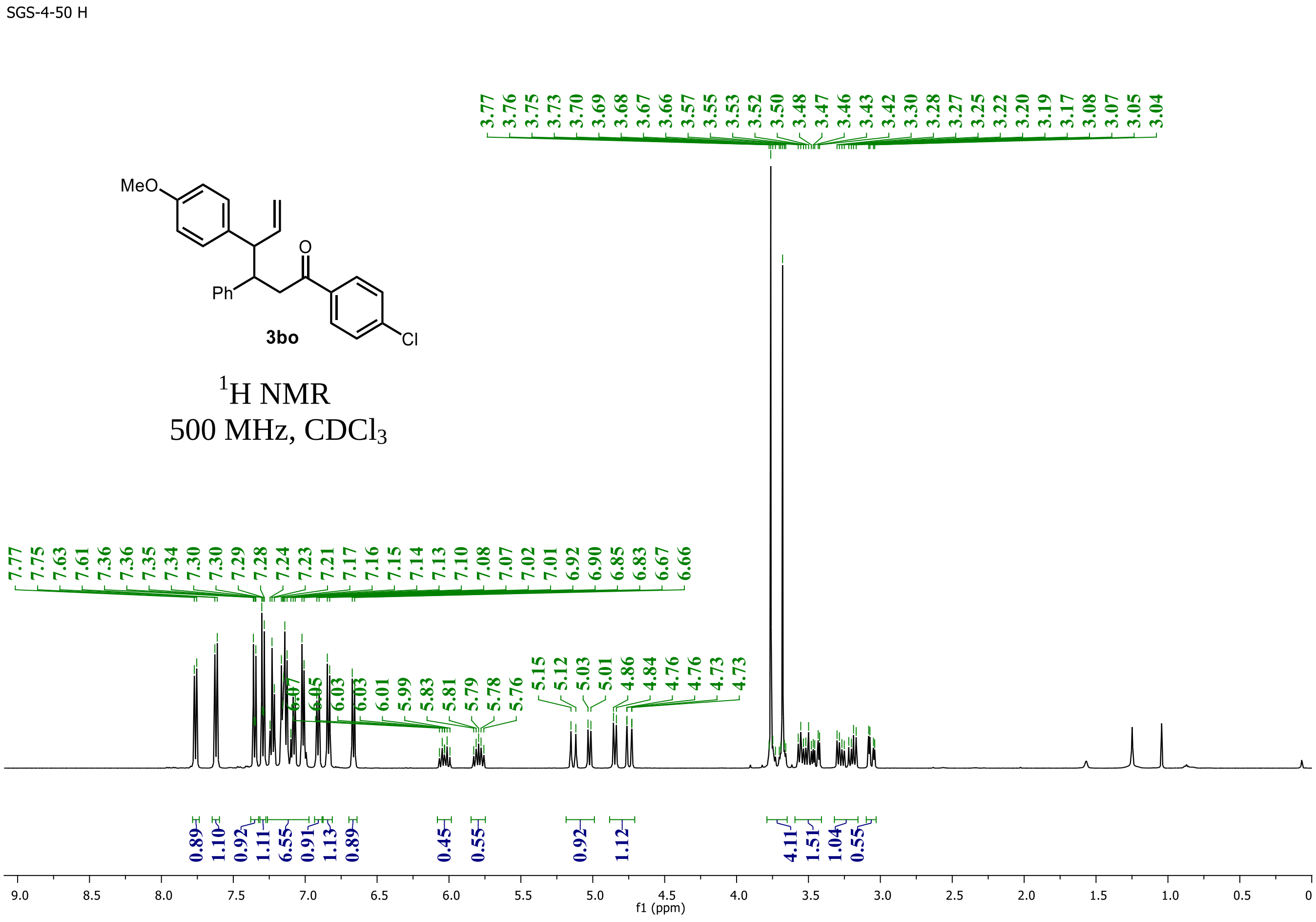

SGS-4-50 C

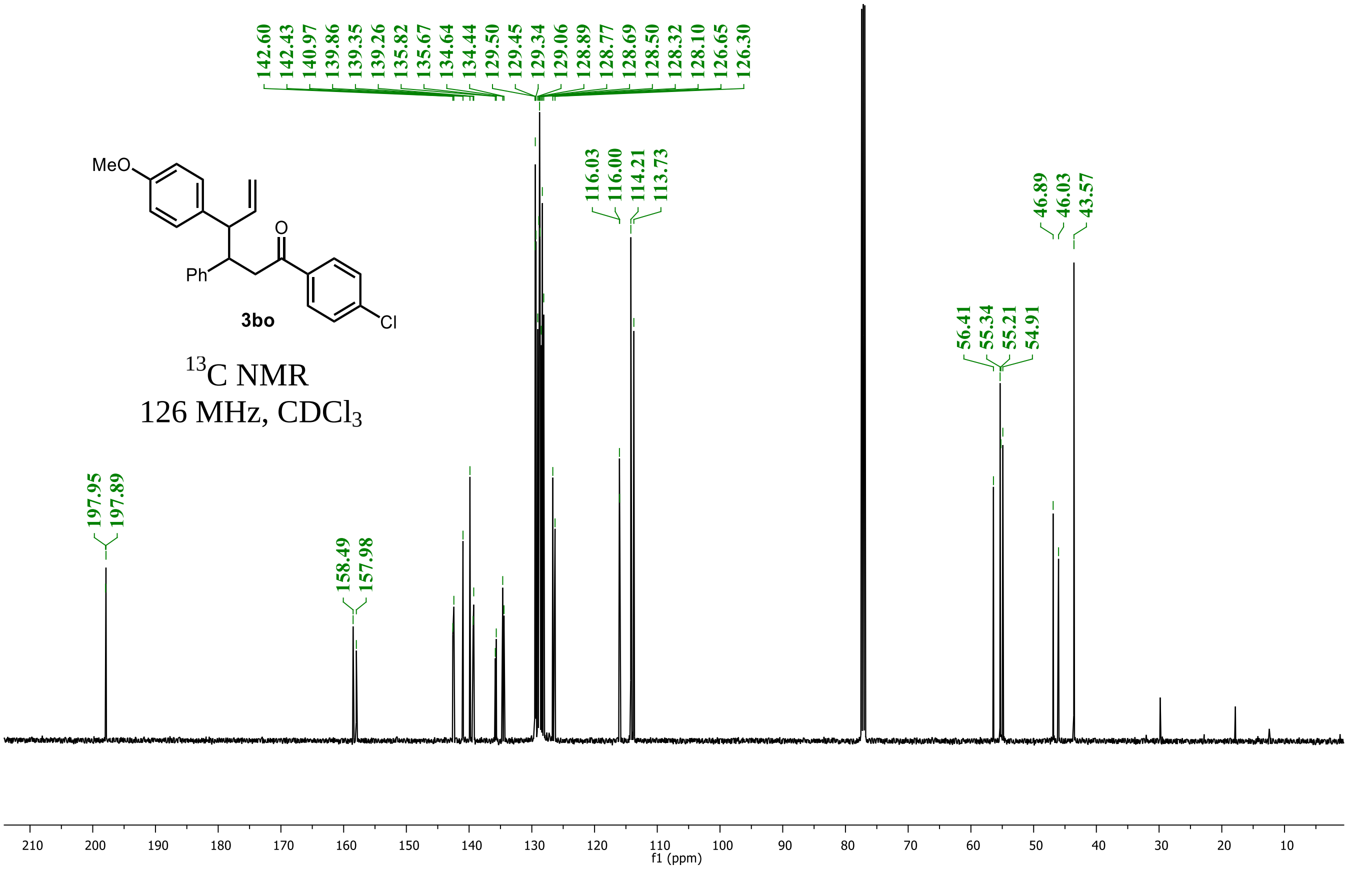

SGS-4-74 H

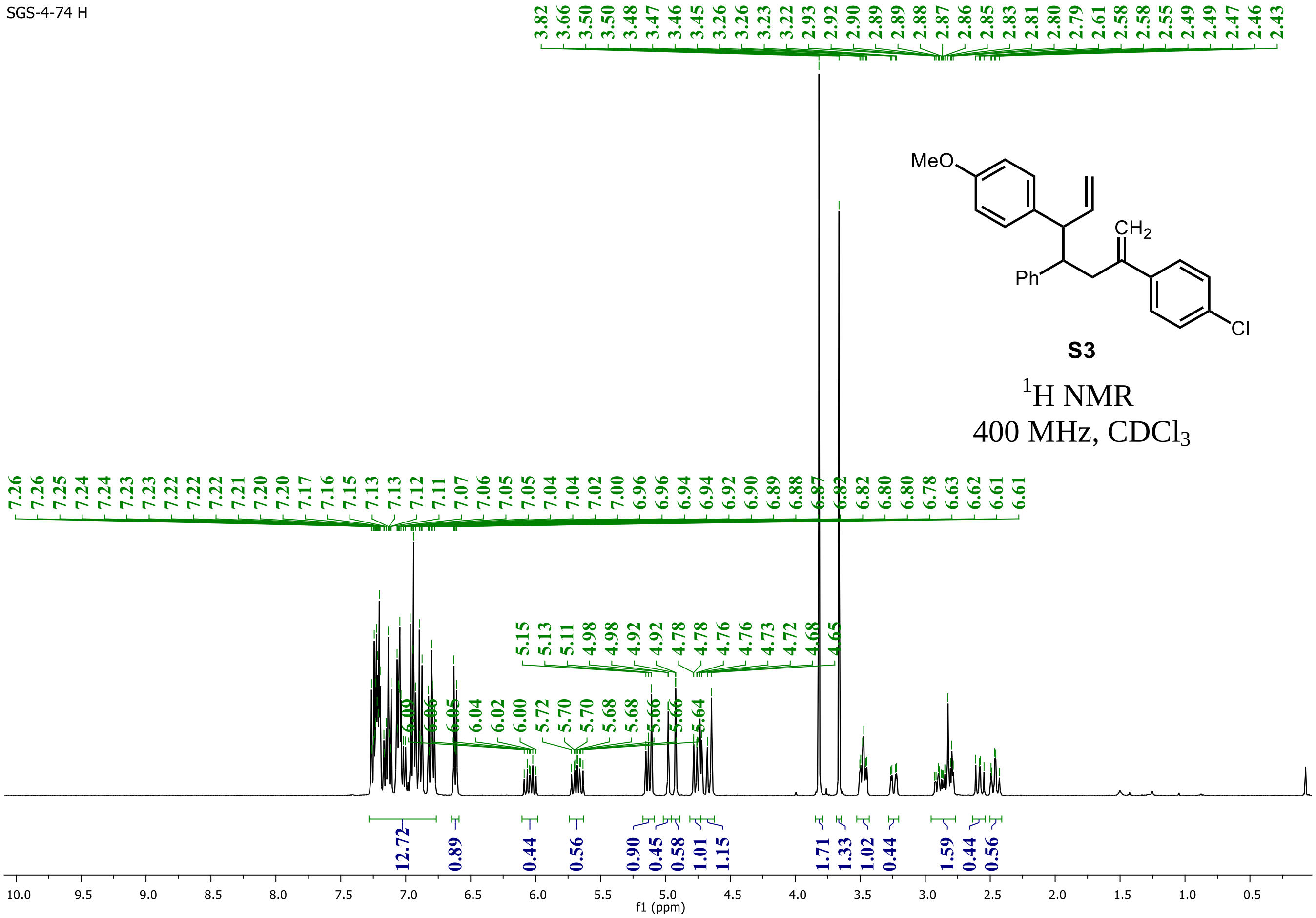

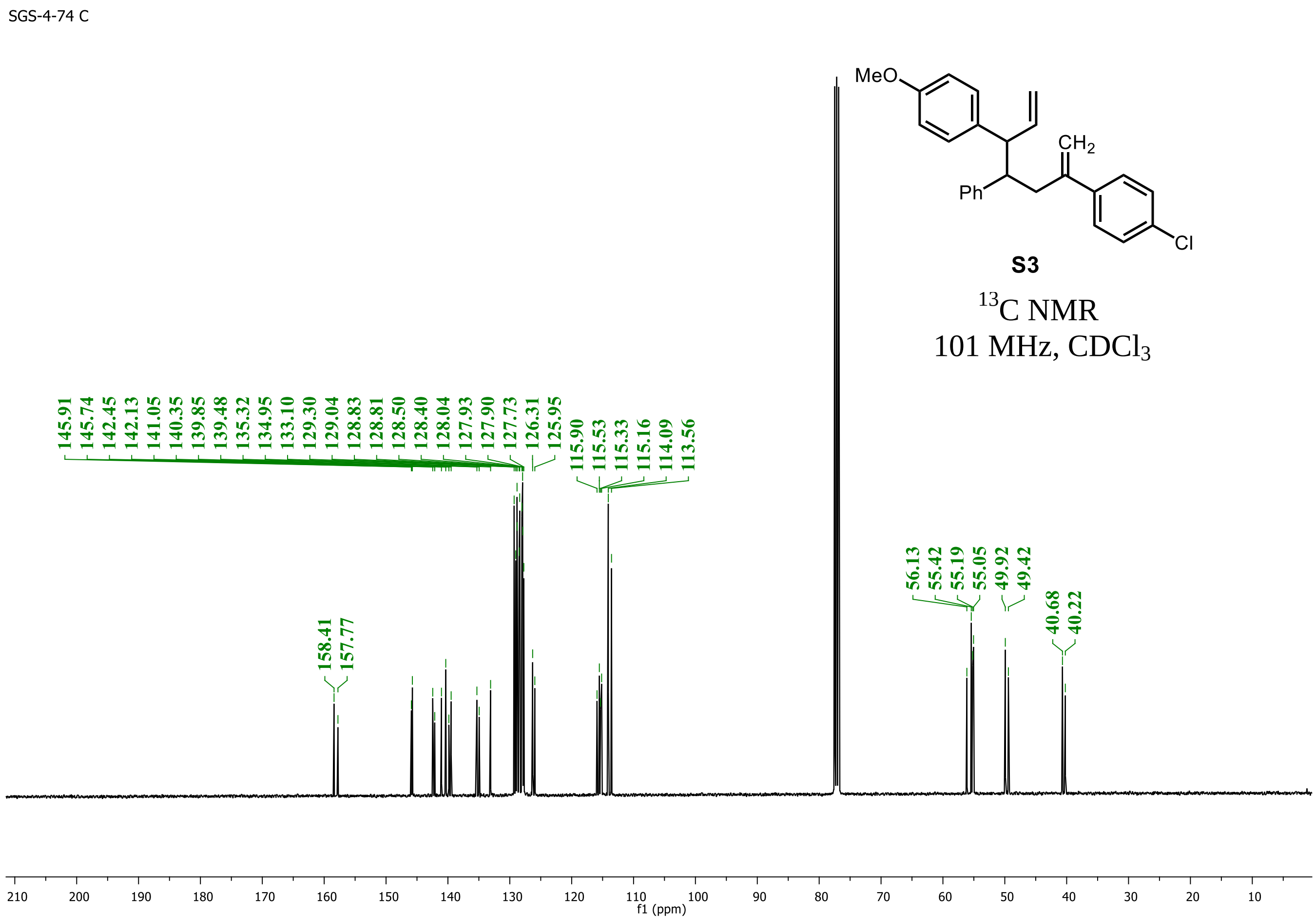
SGS-4-74 C
MeO
CH2
Ph
Cl
S3
13C NMR
101 MHz, CDCl3
145.91
145.74
142.45
142.13
141.05
140.35
139.85
139.48
135.32
134.95
133.10
129.30
129.04
128.83
128.81
128.50
128.40
128.04
127.93
127.90
127.73
126.31
125.95
115.90
115.53
115.33
115.16
114.09
113.56
158.41
157.77
56.13
55.42
55.19
55.05
49.92
49.42
40.68
40.22
f1 (ppm)

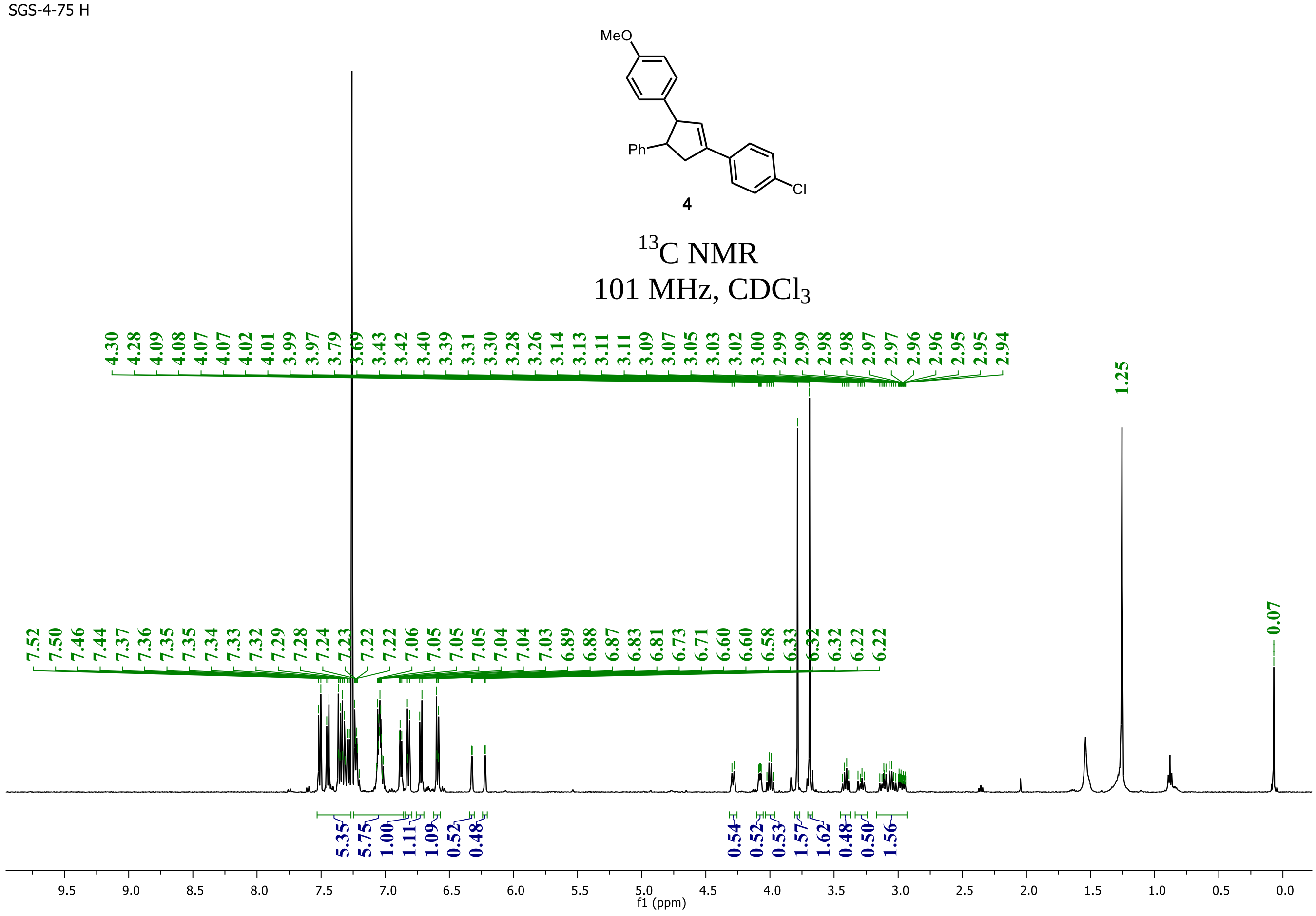
SGS-4-75 H
MeO
Ph
Cl
4
13C NMR
101 MHz, CDCl3
4.30
4.28
4.09
4.08
4.07
4.07
4.02
4.01
3.99
3.97
3.79
3.69
3.43
3.42
3.40
3.39
3.31
3.30
3.28
3.26
3.14
3.13
3.11
3.11
3.09
3.07
3.05
3.03
3.02
3.00
2.99
2.99
2.98
2.98
2.97
2.97
2.96
2.96
2.95
2.95
2.94
1.25
0.07
7.52
7.50
7.46
7.44
7.37
7.36
7.35
7.35
7.34
7.33
7.32
7.29
7.28
7.24
7.23
7.22
7.22
7.06
7.05
7.05
7.05
7.04
7.04
7.03
6.89
6.88
6.87
6.83
6.81
6.73
6.71
6.60
6.60
6.58
6.33
6.32
6.32
6.22
6.22
5.35
5.75
1.00
1.11
1.09
0.52
0.48
0.54
0.52
0.53
1.57
1.62
0.48
0.50
1.56
f1 (ppm)

SGS-4-75-4 C

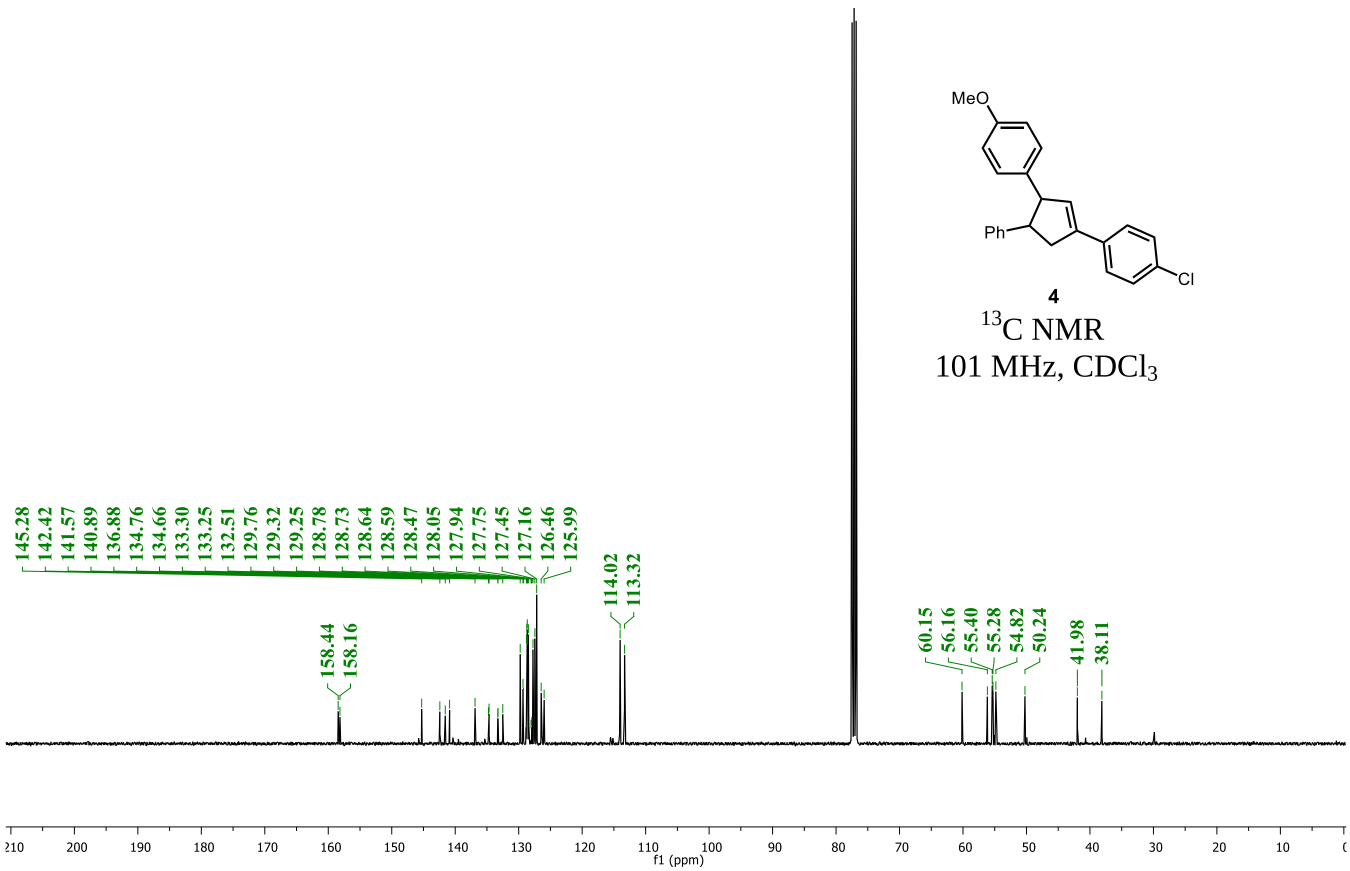

MeO
Ph
Cl
4
^{13}C NMR
101 MHz, CDCl$_3$
145.28
142.42
141.57
140.89
136.88
134.76
134.66
133.30
133.25
132.51
129.76
129.32
129.25
128.78
128.73
128.64
128.59
128.47
128.05
127.94
127.75
127.45
127.16
126.46
125.99
158.44
158.16
114.02
113.32
60.15
56.16
55.40
55.28
54.82
50.24
41.98
38.11
f1 (ppm)

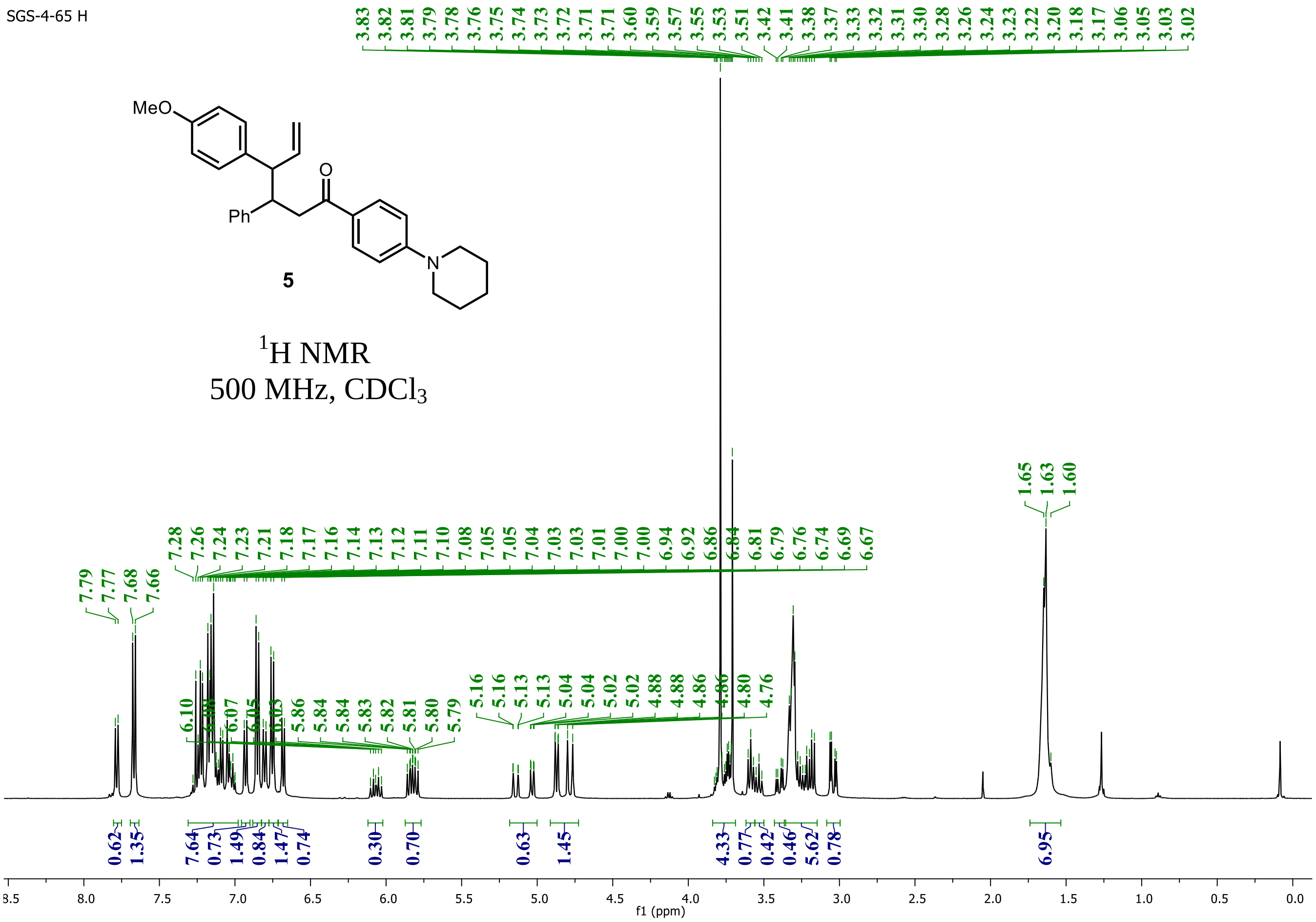
SGS-4-65 H
MeO
O
Ph
N
5
1H NMR
500 MHz, CDCl3
3.83 3.82 3.81 3.79 3.78 3.76 3.75 3.74 3.73 3.72 3.71 3.71 3.60 3.59 3.57 3.55 3.53 3.51 3.42 3.41 3.38 3.37 3.33 3.32 3.31 3.30 3.28 3.26 3.24 3.23 3.22 3.20 3.18 3.17 3.06 3.05 3.03 3.02
1.65 1.63 1.60
7.79 7.77 7.68 7.66
7.28 7.26 7.24 7.23 7.21 7.18 7.17 7.16 7.14 7.13 7.12 7.11 7.10 7.08 7.05 7.05 7.04 7.03 7.03 7.01 7.00 7.00 6.94 6.92 6.86 6.84 6.81 6.79 6.76 6.74 6.69 6.67
6.10 6.07 6.05 5.86 5.84 5.84 5.83 5.82 5.81 5.80 5.79
5.16 5.16 5.13 5.13 5.04 5.04 5.02 5.02 4.88 4.88 4.86 4.80 4.76
0.62 1.35 7.64 0.73 1.49 0.84 1.47 0.74 0.30 0.70 0.63 1.45 4.33 0.77 0.42 0.46 5.62 0.78 6.95
f1 (ppm)

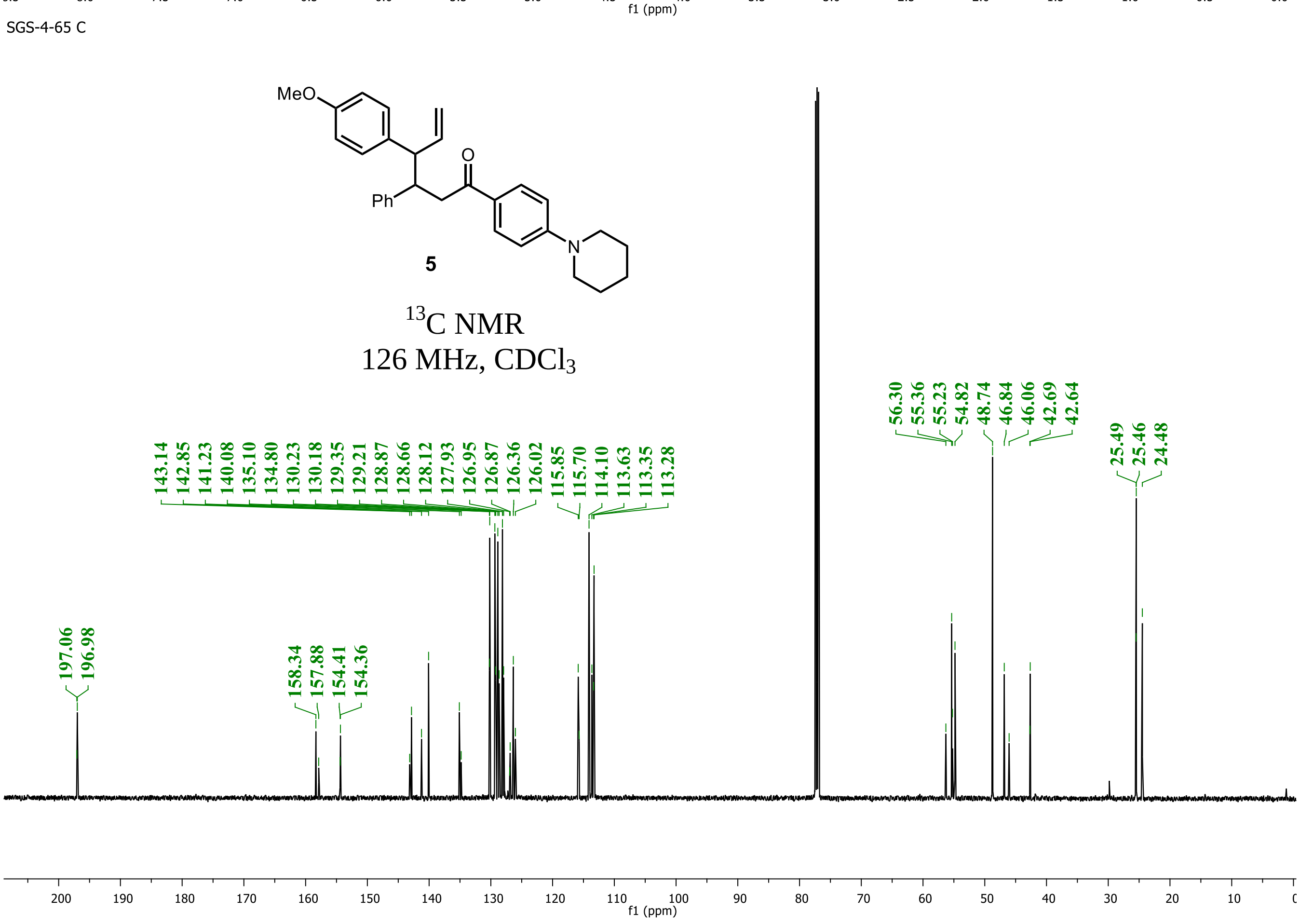
SGS-4-65 C
MeO
O
Ph
N
5
13C NMR
126 MHz, CDCl3
197.06 196.98
158.34 157.88 154.41 154.36
143.14 142.85 141.23 140.08 135.10 134.80 130.23 130.18 129.35 129.21 128.87 128.66 128.12 127.93 126.95 126.87 126.36 126.02
115.85 115.70 114.10 113.63 113.35 113.28
56.30 55.36 55.23 54.82 48.74 46.84 46.06 42.69 42.64
25.49 25.46 24.48
f1 (ppm)

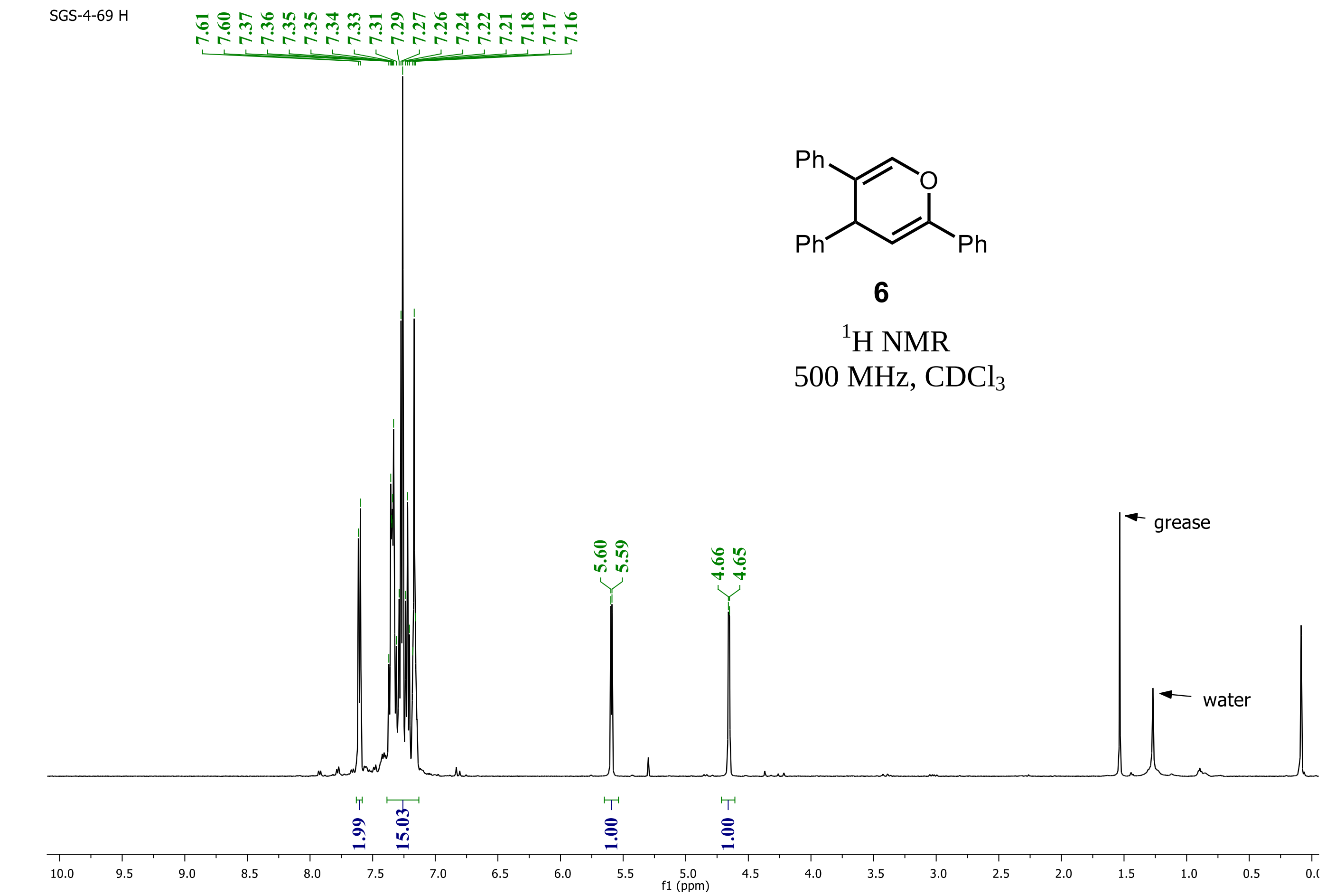
SGS-4-69 H
7.61
7.60
7.37
7.36
7.35
7.35
7.34
7.33
7.31
7.29
7.27
7.26
7.24
7.22
7.21
7.18
7.17
7.16
Ph
O
Ph
Ph
6
1H NMR
500 MHz, CDCl3
grease
water
5.60
5.59
4.66
4.65
1.99
15.03
1.00
1.00
f1 (ppm)

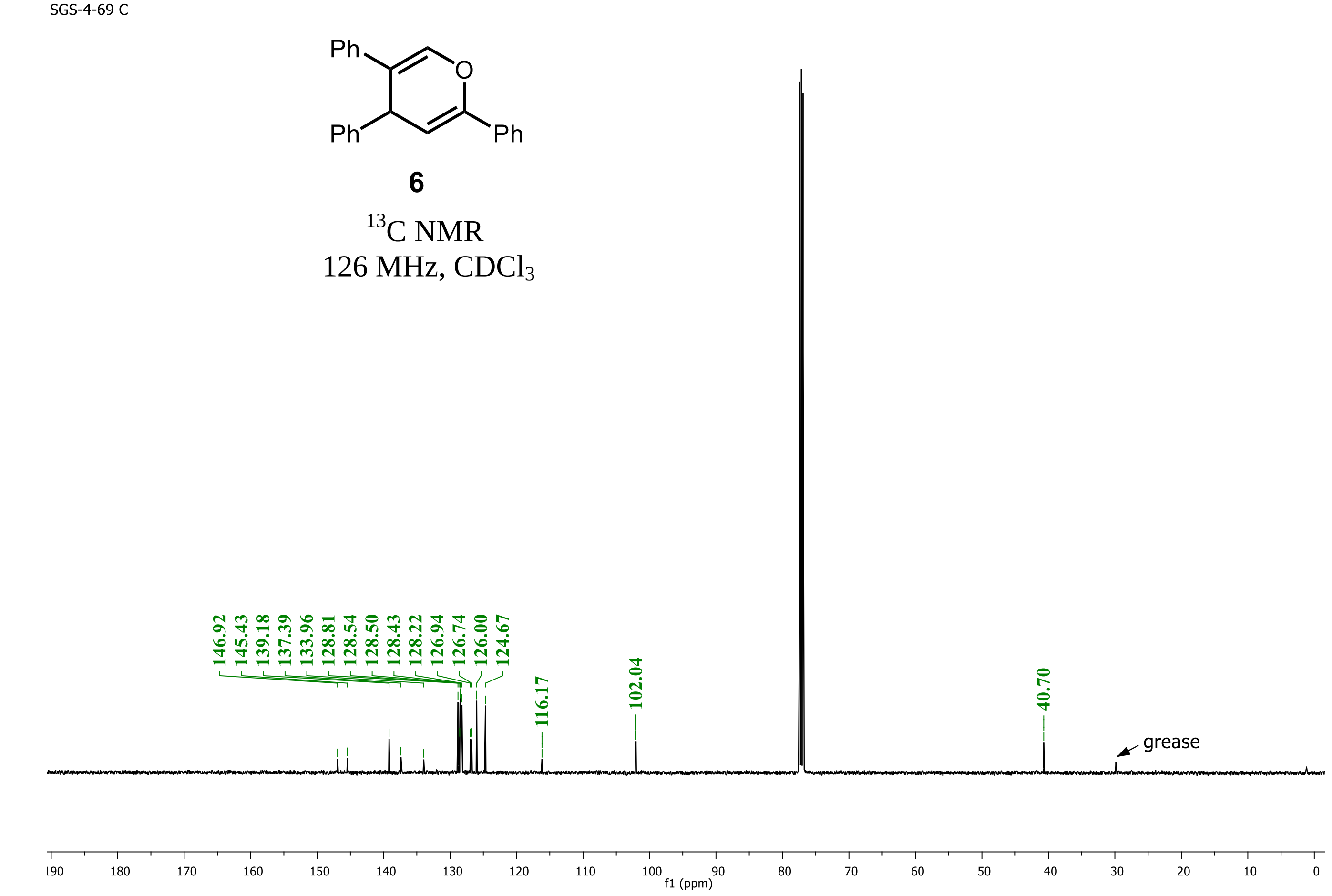
SGS-4-69 C
Ph
O
Ph
Ph
6
13C NMR
126 MHz, CDCl3
146.92
145.43
139.18
137.39
133.96
128.81
128.54
128.50
128.43
128.22
126.94
126.74
126.00
124.67
116.17
102.04
40.70
grease
f1 (ppm)

Copies of NMR spectra of starting materials

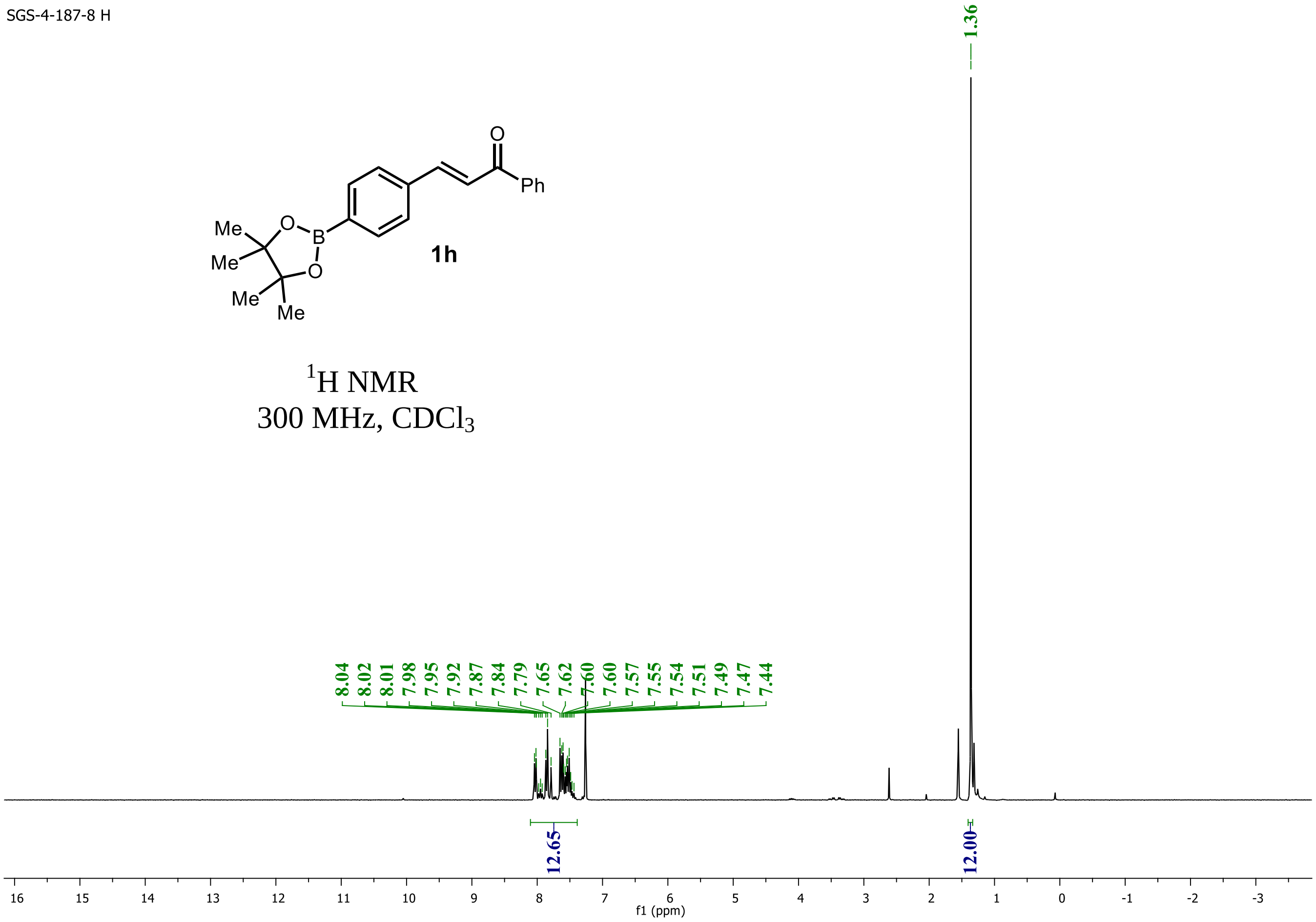

SGS-3-187-8 C

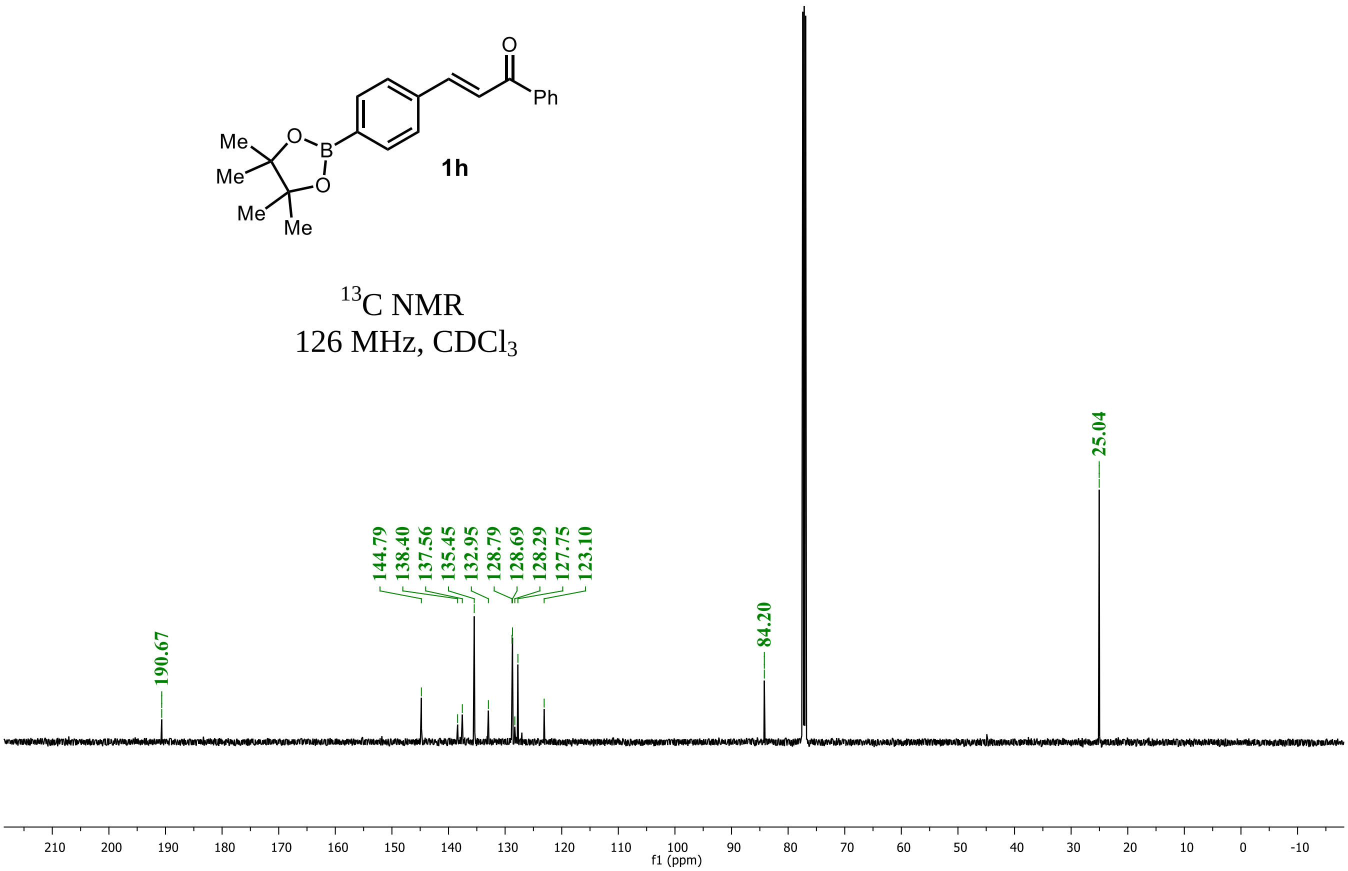

SGS-3-187-12 H

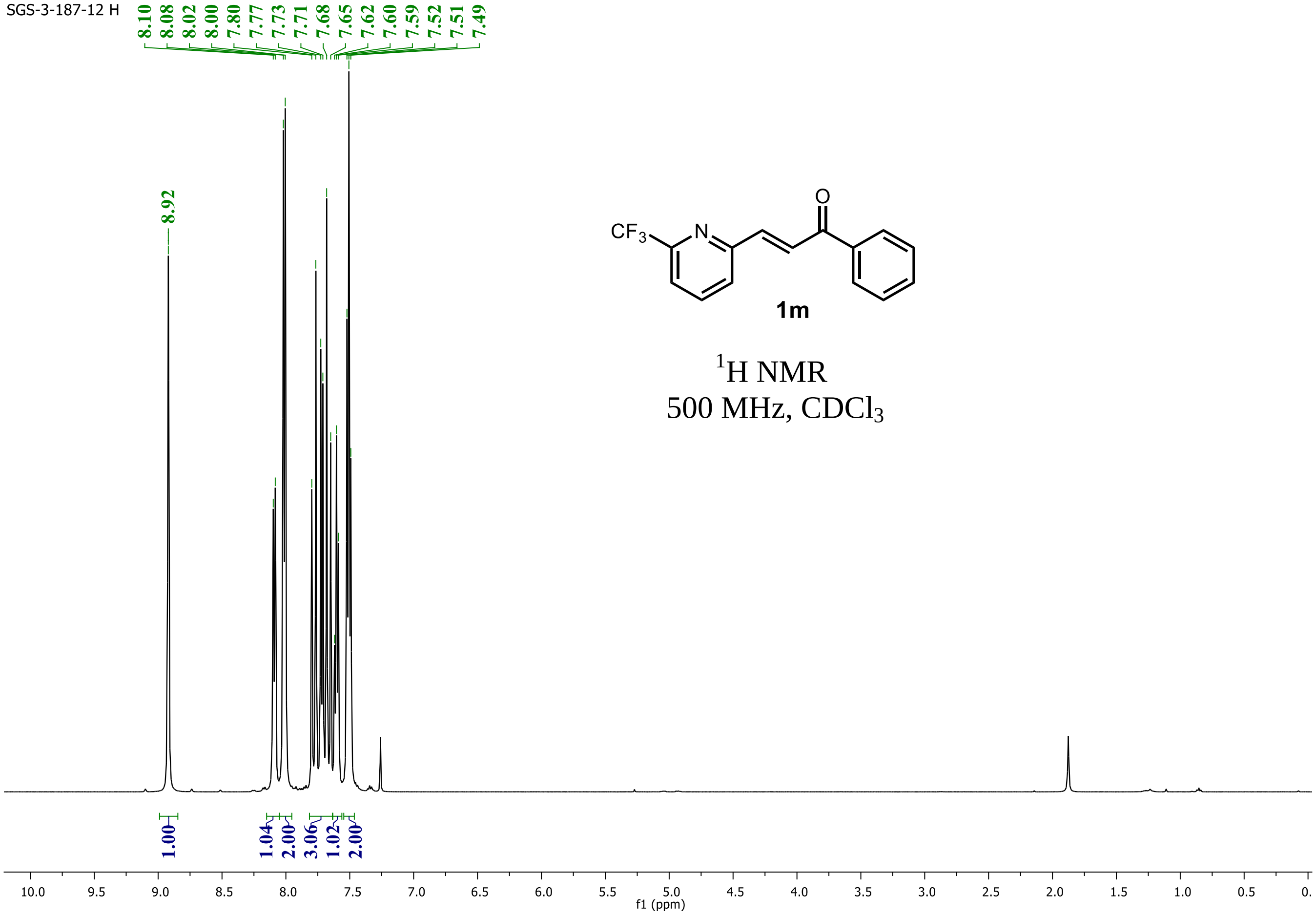

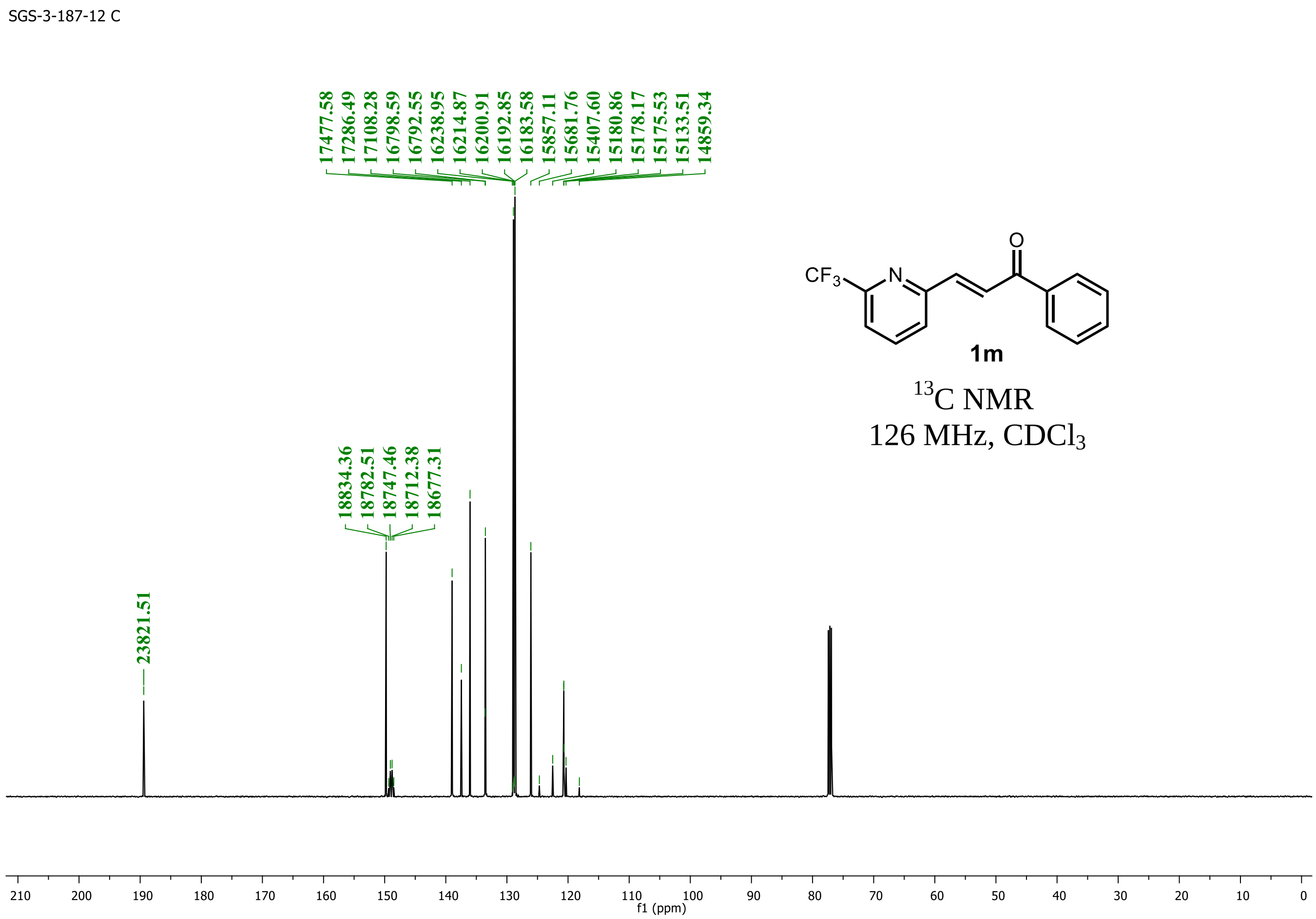
SGS-3-187-12 C
17477.58
17286.49
17108.28
16798.59
16792.55
16238.95
16214.87
16200.91
16192.85
16183.58
15857.11
15681.76
15407.60
15180.86
15178.17
15175.53
15133.51
14859.34
18834.36
18782.51
18747.46
18712.38
18677.31
23821.51
CF3
N
O
1m
13C NMR
126 MHz, CDCl3
210 200 190 180 170 160 150 140 130 120 110 100 90 80 70 60 50 40 30 20 10 0
f1 (ppm)

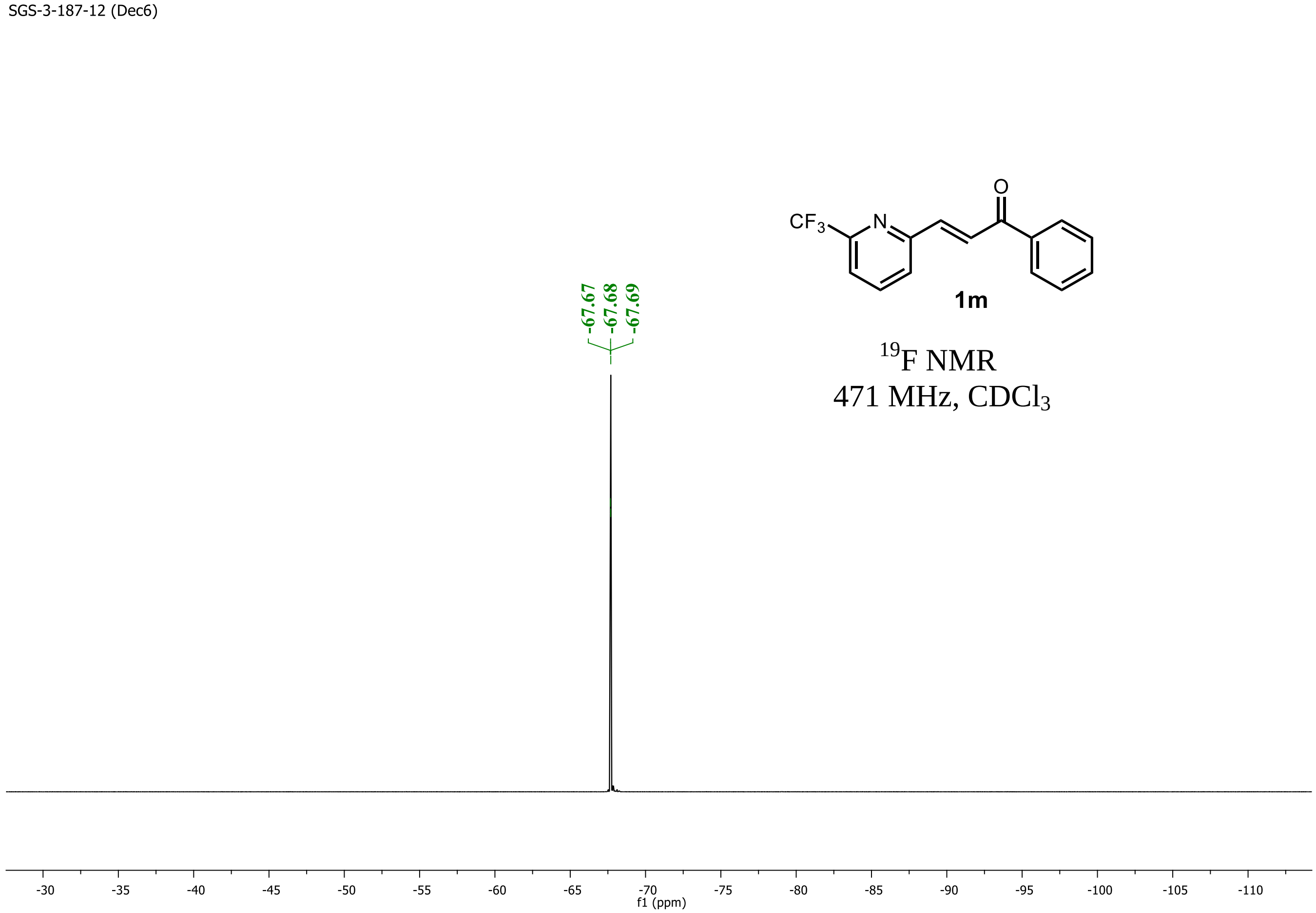
SGS-3-187-12 (Dec6)
CF3
N
O
1m
-67.67
-67.68
-67.69
19F NMR
471 MHz, CDCl3
-30 -35 -40 -45 -50 -55 -60 -65 -70 -75 -80 -85 -90 -95 -100 -105 -110
f1 (ppm)

SGS-3-191-B H

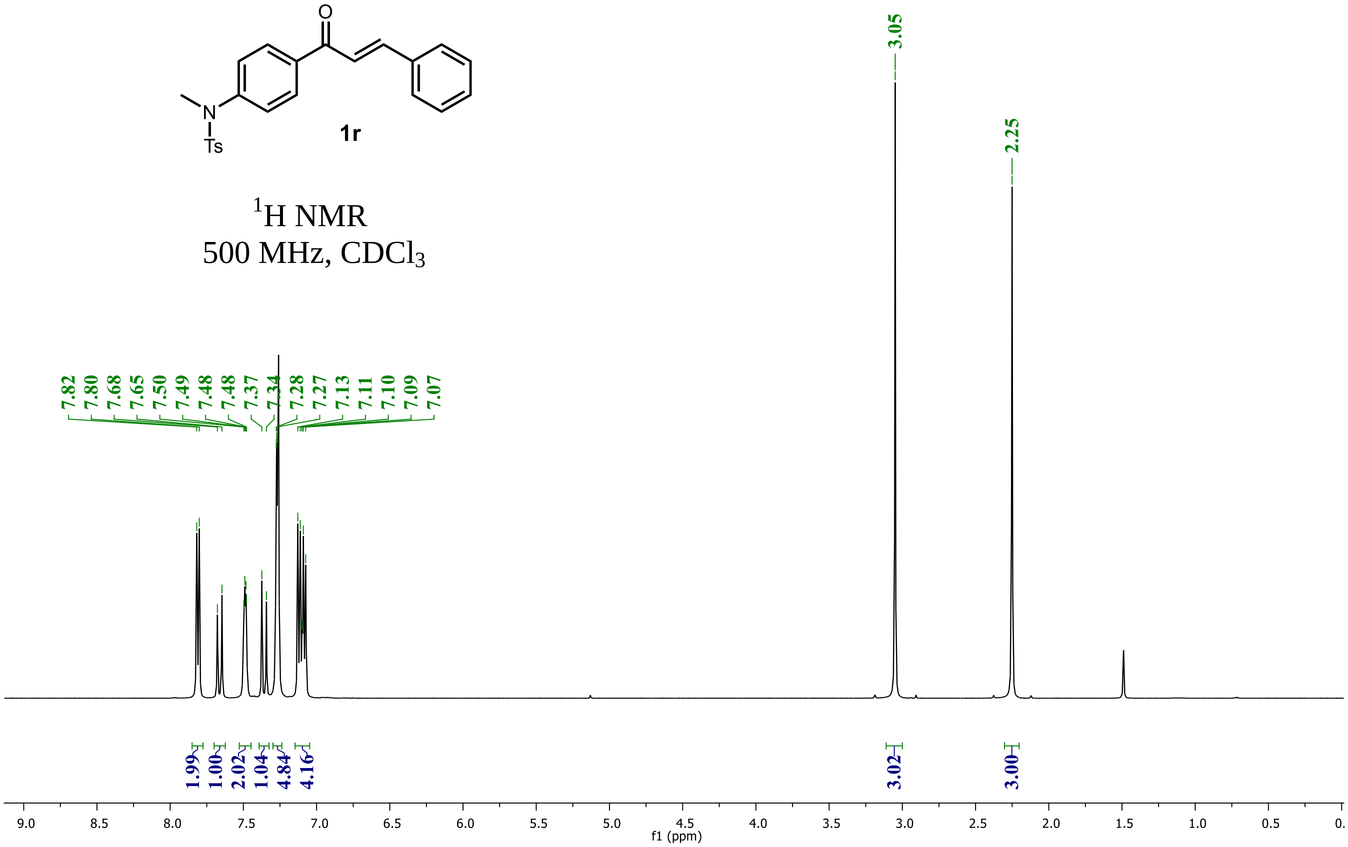

SGS-3-191-B C

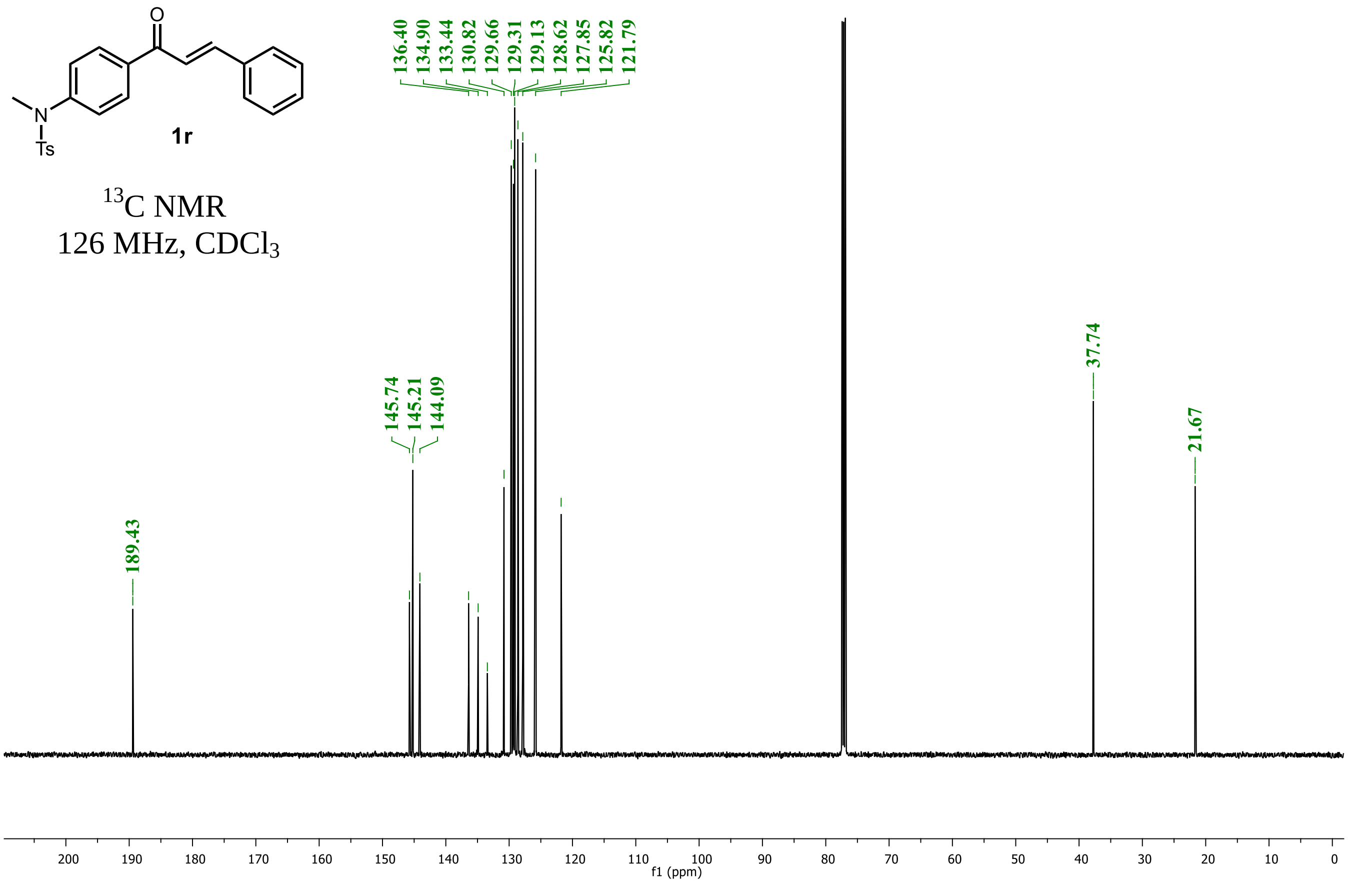

8. References

1. Nicholson, K.; Langer, T.; Thomas, S. P. Borane-Catalyzed, Chemoselective Reduction and Hydrofunctionalization of Enones Enabled by B–O Transborylation. *Org. Lett.* **2021,** *23,* 2498-2504.

2. Shibata, I.; Kano, T.; Kanazawa, N.; Fukuoka, S.; Baba, A., Generation of Organotantalum Reagents and Conjugate Addition to Enones. *Angewandte Chemie International Edition* **2002,** *41,* 1389-1392.

3. Wang, R.; Wang, Y.; Ding, R.; Staub, P. B.; Zhao, C. Z.; Liu, P.; Wang, Y.-M. Designed Iron Catalysts for Allylic C–H Functionalization of Propylene and Simple Olefins. *Angew. Chem., Int. Ed.* **2023**, *62,* e 202216309.

4. Bist, G.; Pun, N. T.; Magar, T. B. T.; Shrestha, A.; Oh, H. J.; Khakurel, A.; Park, P.-H.; Lee, E.-S. Inhibition of LPS-stimulated ROS production by fluorinated and hydroxylated chalcones in RAW 264.7 macrophages with structure-activity relationship study. *Bioorganic & Medicinal Chemistry Letters* **2017,** *27,* 1205-1209.

5. Hiraga, Y.; Kuwahara, R.; Hatta, T. Novel indolo[3,2,1-jk]carbazole-based bipolar host material for highly efficient thermally activated delayed-fluorescence organic light-emitting diodes. *Tetrahedron Lett.* **2021,** *94,* 132317.

6. Wang, Z.; Sun, Y.; Zhang, Q.; Pan, W.; Li, T.; Yin, Y. Bi(OTf)3-Catalyzed Alkyl-Intercepted Meyer–Schuster Rearrangement of Propargylic Alcohols for the Synthesis of 1,2,3,5-Tetrasubstituted Pentane-1,5-diones. *J. Org. Chem.* **2022,** *87,* 3329-3340.

7. Pál, K.; Kállay, M.; Kubinyi, M.; Bakó, P.; Makó, A. Circular dichroism spectra of trans-chalcone epoxides. *Tetrahedron: Asymmetry* **2007,** *18* (13), 1521-1528.

8. Patel, M. N.; Patidar, A. P.; Karia, P. S.; Vekariya, P. A. Cytotoxic, antibacterial and nucleic acid interaction studies of square planar palladium(II) complexes. *Inorganica Chimica Acta* **2014,** *419,* 45-54.

9. Shambhavi, C. N.; Jeganmohan, M. Rh(III)-Catalyzed Enone Carbonyl/Ketone-Directed Aerobic C–H Olefination of Aromatics with Unactivated Olefins. *J. Org. Chem.* **2022,** *87* (19), 13236-13258.

10. Zhang, Y.; Li, X.; Li, J.; Chen, J.; Meng, X.; Zhao, M.; Chen, B. CuO-Promoted Construction of N-2-Aryl-Substituted-1,2,3-Triazoles via Azide-Chalcone Oxidative Cycloaddition and Post-Triazole Arylation. *Org. Lett.* **2012,** *14* (1), 26-29.

11. Bowman, M. D.; Jacobson, M. M.; Blackwell, H. E. Discovery of Fluorescent Cyanopyridine and Deazalumazine Dyes Using Small Molecule Macroarrays. *Org. Lett.* **2006,** *8,* 1645-1648.

12. Rerkrachaneekorn, T.; Tankam, T.; Sukwattanasinitt, M.; Wacharasindhu, S. NaI-Mediated Oxidative Amidation of Benzyl Alcohols/Aromatic Aldehydes to Benzamides via Electrochemical Reaction. *Tetrahedron Lett.* **2021,** *70,* 153017.

13. Díaz-Álvarez, A. E.; Crochet, P.; Cadierno, V. A general route for the stereoselective synthesis of (E)-(1-propenyl)phenyl esters by catalytic CC bond isomerization. *Tetrahedron* **2012,** *68,* 2611-2620.

14. Wang, R., Wang, Y., Ding, R., Staub, P.B., Zhao, C.Z., Liu, P. and Wang, Y. Designed Iron Catalysts for Allylic C–H Functionalization of Propylene and Simple Olefins. *Angew. Chem. Int. Ed.* https://doi.org/10.1002/anie.202216309

15. Matos, J. L. M.; Vásquez-Céspedes, Suhelen.; Gu, J.; Oguma, T.; Shenvi, R. A. Branch-Selective Addition of Unactivated Olefins into Imines and Aldehydes. *J. Am. Chem. Soc.* **2018,** *140,* 16976-16981.

16. Chen, J.; Zhu, S. Nickel-Catalyzed Multicomponent Coupling: Synthesis of α-Chiral Ketones by Reductive Hydrocarbonylation of Alkenes. *J. Am. Chem. Soc.* **2021,** *143,* 14089-14096.

17. Lu, Z.; Hennis, O.; Gentry, J.; Xu B.; Hammond, G. B. Base-Promoted Radical Azofluoromethylation of Unactivated Alkenes. Org. Lett. **2020**, *22,* 4383-4388.

18. Yue, W.-J.; Day, C. S.; Brenes Rucinski, A. J.; Martin, R. Catalytic Hydrodifluoroalkylation of Unactivated Olefins. *Org. Lett.* **2022,** *24,* 5109-5114.

19. Chatterjee, A. K.; Choi, T.-L.; Sanders, D. P.; Grubbs, R. H. A General Model for Selectivity in Olefin Cross Metathesis. *J. Org. Chem. Soc.* **2003**, *125,* 11360-11370.

20. Eiden, F.; Eckle A. ZNS-wirksame 4-Phenylpyranderivate: Synthese und Reaktionen von 4-Phenyl-2-piperidino-dihydropyran-5-carbonsauree. *Arch. Pharm. (Weinheim)* **1989,** *322,* 617-627.
